# An Evaluation of Avian Influenza Virus Whole-Genome Sequencing Approaches Using Nanopore Technology

**DOI:** 10.3390/microorganisms11020529

**Published:** 2023-02-19

**Authors:** Hon S. Ip, Sarah Uhm, Mary Lea Killian, Mia K. Torchetti

**Affiliations:** 1 National Wildlife Health Center, U.S. Geological Survey, Department of the Interior, Madison, WI 53711, USA; 2 Department of Pathobiological Sciences, School of Veterinary Medicine, University of Wisconsin-Madison, Madison, WI 53706, USA; 3 National Veterinary Services Laboratories, U.S. Department of Agriculture, Ames, IA 50010, USA

**Keywords:** avian influenza, whole-genome sequencing, next-generation sequencing, nanopore sequencing

## Abstract

As exemplified by the global response to the SARS-CoV-2 pandemic, whole-genome sequencing played an important role in monitoring the evolution of novel viral variants and provided guidance on potential antiviral treatments. The recent rapid and extensive introduction and spread of highly pathogenic avian influenza virus in Europe, North America, and elsewhere raises the need for similarly rapid sequencing to aid in appropriate response and mitigation activities. To facilitate this objective, we investigate a next-generation sequencing platform that uses a portable nanopore sequencing device to generate and present data in real time. This platform offers the potential to extend in-house sequencing capacities to laboratories that may otherwise lack resources to adopt sequencing technologies requiring large benchtop instruments. We evaluate this platform for routine use in a diagnostic laboratory. In this study, we evaluate different primer sets for the whole genome amplification of influenza A virus and evaluate five different library preparation approaches for sequencing on the nanopore platform using the MinION flow cell. A limited amplification procedure and a rapid procedure are found to be best among the approaches taken.

## 1. Introduction

The emergence and global spread of SARS-CoV-2 has led to widespread use of whole-genome sequencing to guide therapeutic countermeasures for individuals, as well as for population-scale epidemiological monitoring. With over four million genomes sequenced, patterns of introduction, dissemination, and evolution of variants have been well-described [1], but one area where the state of the art might improve is the ability to sequence in real time or near real time [2]. In recent years, highly pathogenic avian influenza (HPAI) viruses have been introduced and have circulated in wild birds and poultry in Asia, Europe, and North America [3,4,5,6]. Whole-genome sequencing, in conjunction with epidemiological information, has often been used to elucidate routes of introduction and patterns of spread in HPAI outbreaks [7,8]. To date, however, there are no global programs for the large-scale sequencing and analysis of HPAI viral sequences analogous to that for SARS-CoV-2. For example, since 2021, in spite of nearly 2400 HPAI H5Nx outbreaks in poultry and over 2700 similar events in wild birds in Europe [9], plus another 673 detections in poultry [10] and 4362 detections in wild birds in the United States [11], as of 8 December 2022, only 3116 HPAI H5Nx genomes were deposited in the GenBank and Global Initiative on Sharing All Influenza Data (GISAID) databases.

Avian influenza viruses are members of the family Orthomyxoviridae [12]. The genome contains eight single-stranded RNA segments ranging in size from 890 to over 2280 nucleotides long. A conserved motif of 12 nucleotides is present at the 5′- and 3′-end of each RNA segment, and these conserved residues have been used as targets for cDNA synthesis and sequencing [13].

Reducing costs and turn-around times are among the key challenges that laboratories face when adopting next-generation sequencing (NGS) technology [14,15]. Illumina sequencing systems, such as the MiSeq, are characterized by relatively short reads (2 × 300 bp) and long sequencing times (36 h), but feature high sample throughput, high data density, and high sequence accuracy [14]. They additionally require investment to purchase and maintain a benchtop-scale sequencing instrument and involve complex workflows. In contrast, Oxford Nanopore Technology (ONT) MinION utilizes a low-cost, portable nanopore sequencing device to yield longer read lengths and data availability in real time, but it is limited by higher error rates and lower sensitivity [16]. Other NGS platforms exist, but Illumina and ONT were used to generate 90% (65% and 25%, respectively) of the 4.8 million SARS-CoV-2 genomes deposited in GISAID as of 5 November 2021 [16].

By producing readable sequencing data within 30 min, the MinION platform offers the ability to decrease the time for pathogen identification when speed is of the essence [17]. Additionally, the smaller capacity of the MinION may be well-suited to the number of avian influenza surveillance samples that need to be sequenced at any one time by an individual diagnostic laboratory. Several studies have described the whole-genome amplification and nanopore sequencing of avian influenza viruses from both wild and domestic bird samples [18,19,20]. Moreover, strategies to achieve >99% accuracy (often by increasing the depth of coverage, i.e., increasing the number of reads over each sequence location) in both SARS-CoV-2 and avian influenza virus sequencing have been published [19,20,21]. Although a variety of approaches and library preparation methods have been described for the sequencing of avian influenza viruses on the MinION platform (e.g., native barcoding kit (SQK-LSK109 [19], PCR barcoding kit (SQK-PBK004) [22], and rapid barcoding kit (SQK-RBK004) [20]), the performance differences between these methods have not yet been evaluated.

In this study, we evaluated the performance of RT-PCR primers for the conversion of avian influenza virus RNA to cDNA and the utility of different library preparation kits for the sequencing of the whole avian influenza genomes on the MinION platform.

## 2. Materials and Methods

### 2.1. Viruses Used in This Study

Three highly pathogenic H5N1 avian influenza virus-containing cloacal or tracheal swab samples collected from wild birds were chosen because not only do these samples represent the typical sample received in the laboratory for wild-bird surveillance testing, but they also span the cycle threshold (Ct) range typically seen in positive samples (Table 1).

Viral RNA was extracted using the MagMAX-96 AI/ND Viral RNA Isolation Kit (ThermoFisher) using a KingFisher magnetic particle processor and 90 µL elution volume.

### 2.2. RT-PCR

Reverse-transcription–polymerase chain reaction (RT-PCR) was performed using the primer sets listed in Table 2. Briefly, 5 μL of extracted RNA was amplified in a 25 μL reaction with Invitrogen SuperScript III RT-PCR mix (ThermoFisher, Waltham, MA, USA). Where referenced, the RT-PCR cycling conditions are as cited. For primer Set 2, the reaction was incubated as per Zhou and Wentworth [13].

cDNA was purified by diluting the reaction to 50 μL with nuclease-free water and adding 50 μL of AMPure beads (1:1 vol:vol) (Beckman Coulter, Brea, CA. USA). Following incubation at room temperature for 5 min, the tubes were placed on a magnetic stand for 2 min. The supernatant was discarded, and the pellets were washed with 500 μL of 80% ethanol twice, allowed to dry briefly, and eluted in 20 μL of nuclease-free water; 1 μL of the recovered cDNA was quantitated using the Qubit dsDNA HS (High Sensitivity) Assay Kit (ThermoFisher, Waltham, MA, USA).

### 2.3. Library Kit Comparison

The primer sets in Table 2 are designed to be used with different library preparation kits from Oxford Nanopore Technologies (London, UK) as some kits have their own specific requirements for purposes such as attachment or PCR amplification of barcodes (Supplementary Table S1). In Method A, libraries were constructed with cDNAs amplified with Set 1 primers using the native bar-coding kit SQK-LSK109. Method S used Set 2 primer-amplified cDNA and the PCR barcoding kit (SQK-PBK004). Method E constructed libraries with Primer Set 3-amplified cDNA and the rapid barcoding kit (SQK-RBK004). Method K used cDNAs amplified with Set 4 primers to prepare libraries with the rapid PCR barcoding kit (SQK-RPB004). One additional experiment was performed with Set 4-amplified cDNA using a newly described procedure for the whole-genome sequencing of avian influenza viruses with the SQK-LSK109 kit (Method N, [24]). The samples were barcoded, and library constructed as per manufacturer’s instructions except for Set 1, where equal volumes of New England Biolabs (Ipswitch, MA. USA) FFPE DNA repair mix were added to the end prep reaction.

### 2.4. MinION Runs

R9.4.1-version MinION flow cells were used in this study. Flow cells were loaded as per manufacturer’s instructions on an MK1c portable standalone sequencing unit. Runs were 48 h but were terminated early when little data continued to be generated, or when the Q score was below 10, depending on which came first.

### 2.5. Sequence Analysis

Base calls were exported as FASTQ files by the MK1c during the run, and the barcodes were deconvoluted. The FASTQ files were imported into the CLC Genomic Workbench (Qiagen, Hilden, Germany) where the sequences were mapped to a standard set of avian influenza reference sequences as described by Crossley et al. [19]. Mapped consensus sequences corresponding to each segment were compared to GenBank using BLAST, and closer homologs were identified. The sequences of the closer homologs were then used as new reference sequences for a new round of mapping. The process was repeated recursively until no closer GenBank entries were found. The final consensus sequences for each segment were then exported in FASTA format. The coding region for each segment was examined using MacVector (Apex, NC, USA), and if the expected coding region was interrupted, the MinION reads over the corresponding region were manually examined for possible sequencing or assembly errors, as per Delahaye and Nicolas [25], and the sequence manually edited. Sequence accuracy was evaluated between the various methods using T-Coffee multiple sequence alignment against the sequencing of the same RNA samples on a benchtop instrument (MiSeq, Illumina, San Diego, CA, USA).

## 3. Results

### 3.1. cDNA Generation by RT-PCR

**Figure 1:**
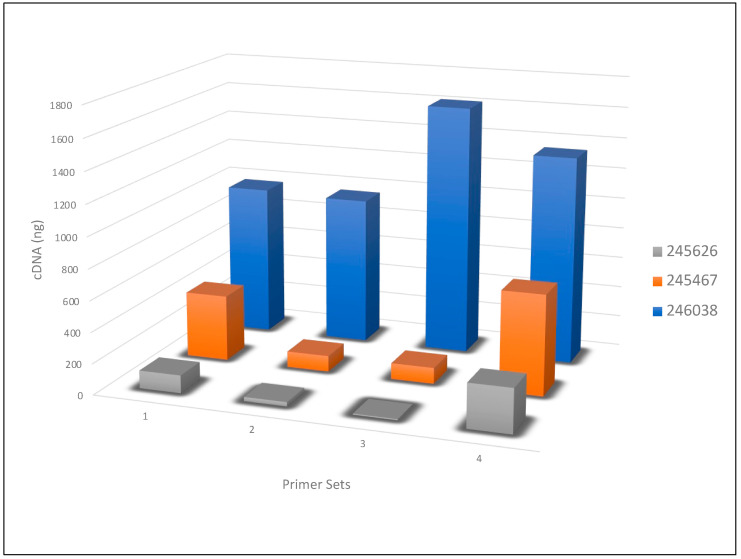
Net cDNA generated with four primer sets. The amount, in nanograms, of cDNA recovered following bead purification of the SuperScript III RT-PCR reaction on the three HPAI H5N1 swab samples is shown on the Y axis. Primer sets used (1–4, see Table 2) are listed on the X axis and the RNA samples used (from Table 1) are listed in the figure legend.

The amount of cDNA generated was calculated from the qubit quantitation value. Even though all primer sets essentially contain the same 12 or 13 nucleotide sequence homologous to the influenza RNA segments, the amount of products generated was variable (Figure 1). For example, the RNA from sample 245626 had a 38-fold difference among the four primer sets.

### 3.2. Library Construction Kits Evaluated

**Figure 2:**
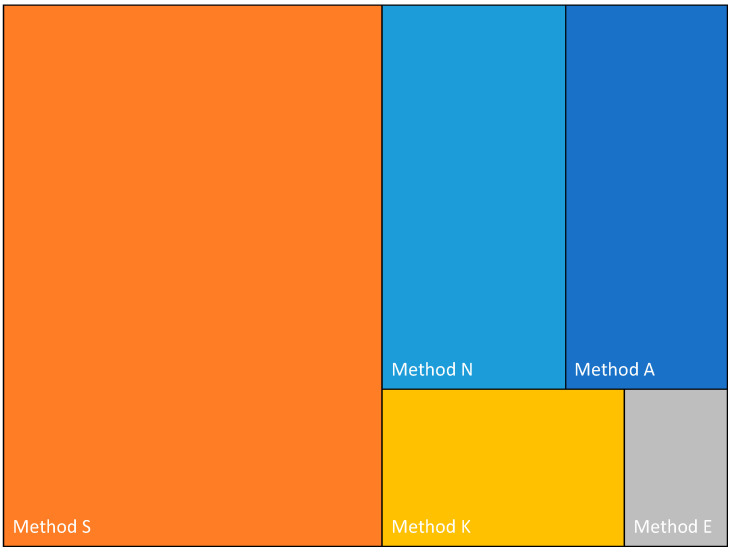
Treemap representation of the time required for the methods evaluated.

The major steps involved with each of the kits used in the methods evaluated in this study are summarized in Supplementary Table S1. SQK-LSK109 (Method A) is the kit often used for the barcoding of samples if additional amplification is not required. The other kits examined all involve some degree of PCR amplification, with Method S taking the longest time to complete. Of note is the number of AMPure bead purification steps required in the different protocols, which ranged from two (Methods E, K, and S) to four (Methods A and N). The total time required for a single sample (not including the RT-PCR step) until the sample is ready for loading onto the flow cell ranged from 18 min (Method E) to 228 min (Method S) (Figure 2).

### 3.3. Read Comparisons

A total of 24.4 million reads were generated among the kits evaluated, with average read lengths between 300 bp (Method E) to 1.07 kb (Method N). Together, 20.5 million reads mapped to the HPAI H5N1 genome. The number of reads generated using each kit varied widely and ranged from 1.7 million (Method N) to 9.4 million (Method S) (Table 3). Four of the methods had an average of 87–88% reads mapping to the H5N1 genome with Method E being an outlier with only 68% mapping to H5N1.

The total number of mapped reads was not a good measure of the kits’ performances, as some kits overamplified the ends of the viral RNA segments or failed to span the length of some of the segments. Using RNA 246038 as an example, between 10,758 (Method E) and about 3.6 million reads (Method S) were mapped to HPAI H5N1 by the five different methods, but only Methods A, K, and N were able to cover the entire genome (Table 4).

Several sequence variations were found in the consensus sequence generated from each kit. The number of variations for the three RNA samples is tabulated in Supplementary Table S2a–c, and the total number of sequence variations is given in Table 5.

The assembled consensus sequences from each method was used in a multiple sequence alignment against the reference sequences generated on the MiSeq (Supplementary Figures S1a–h, S2a–h, and S3a–h). Only one sequence variation was shared across the kits; the distribution of all other variations did not follow a discernable pattern. The one common variation was in the PB2 sequence of Sample 245626, where C19 was changed to A19 in the sequence assembled from all four methods with complete PB2 sequences. The change from CTA to ATA changes the amino acid encoded from leucine to isoleucine. The seven-nucleotide deletion in Method N was in the PB2 segment, where only 695 reads in total were mapped to this gene (Table 4, Supplementary Figure S2a). The deletion resulted in a frame shift and a premature termination after residue 731 rather than 760. Method E produced a large number of sequence mutations, the variations of which could be seen across all three RNA samples but especially in those with lower Ct values (Supplementary Figures S1a–h, S2a–h, and S3a–h).

## 4. Discussion

The sequencing of avian influenza viruses (AIV) was instrumental in defining the multiple introductions of highly pathogenic avian influenza viruses into North America in 2021–2022 [5,6]. Moreover, detailed genetic studies are useful in defining the relationships between outbreaks, identifying independent introductions, farm-to-farm spread, and superspreader events (e.g., [7,8]). In a typical year, the Centers for Disease Control and Prevention (CDC) sequences approximately 7000 influenza viruses from patient samples [26]; this is fewer than the number of SARS-CoV-2 viruses that are being sequenced [1]. Wider availability of rapid and preferably low-cost whole-genome sequencing methods for agents of emerging infectious diseases, including avian influenza viruses [27], could facilitate additional sequencing efforts. The nanopore-technology-based sequencing systems described in this manuscript met both criteria [28].

Although some publications have described the sequencing of influenza viruses on the MinION platform, few have described the selection and optimization that led to the final publication [19,20,23]. In this study, we used three wild-bird swab samples that were positive for the HPAI H5N1 virus to examine the role of oligonucleotide primers for whole-genome amplification and the use of different library preparation kits from ONT for their sequencing on the MinION platform. The RNA samples were chosen as they represented a typical range of samples that have been successfully sequenced on the Illumina MiSeq platform. The kits were chosen as their use has previously been published (Methods A, K, N, and S), or they had potential to offer useful features such as minimal handling steps and rapid time to first results (Method E).

The original Zhou and Wentworth primers (Set 1) generated much more cDNA than those using Sets 2 and 3 [13]. Set 4, which is a modification of the Mitchell et al. B primers [23], generated substantially more cDNA on each sample and particularly with the sample containing the lowest viral titer (Sample 245626). It is likely that secondary structural differences between the non-influenza A sequences in the primers might play a role in the efficiency of reverse transcriptase priming and cDNA synthesis. For example, primers in both Set 2 and Set 4 contain the same 3′ sequence as in the Zhou and Wentworth Set 1, as well as the same 5′ sequence (Table 2), which were added as anchor sequences for PCR amplification and barcoding with ONT’s SQK-PBK004 kit but differ in the sequence added as a spacer between the two domains. Based on our results, Set 4 primers were more appropriate for AIV whole-genome amplification, but additional analysis of secondary structures and thermodynamics of these and other primers could yield further optimization.

The five procedures (called “Methods” for the sake of simplicity in this paper) differ in terms of complexity and hands-on time (Supplementary Table S1). The differences are expected to be further heightened when more samples than the three used in this study are processed together in a multiplexed sequencing run.

The use of only three samples vastly underutilized the capacity of the MinION flow cell, so the total number of reads generated and the degree of coverage in this study is not expected to be typical during a production run in the laboratory (Table 3). However, even with only three samples, it was unexpected when some methods (E and S) did not result in the assembly of the complete genome from any of the samples, including from the sample with the higher viral titer (Sample 246038, Table 4). Care is warranted in interpreting the results because the effects seen with the ONT kits are compounded by the primer sets used in the RT-PCR step. Not all primer sets are compatible with every kit because some kits require specific sequences in the primers for purposes such as barcoding; thus, a direct comparison utilizing a common primer set could not be determined in the present study. The following are a few caveated observations. Method E, which has the least degree of PCR amplification, resulted in many sequence variations (Supplementary Table S2a–c). This method also had the lowest number of reads mapped with every sample tested and on every viral segment. Method S generated uneven read coverage, failing to completely sequence the genome from Sample 245626, which had the lowest viral titer. Method N is based on a recent protocol, and the overall procedure was similar to that in Method A as both methods use ONT’s SQK-LSK109 library construction kit. Method N was designed for the simultaneous sequencing of influenza A and B viruses from human samples, but we replaced the specified primers with Set 4, as that gave us superior amplification of avian influenza viruses. Method N generated complete genomes from all three RNA samples even though several segments (e.g., PB2) had very few mapped reads. In fact, across the five kits, Method N had the greatest number of viral segments with less than 10,000 reads per segment. The low number of reads in Method N, when much of the procedure is shared with Method A, indicates that optimization of the Method N procedure might result in improved generation of reads.

The principle of ONT’s nanopore sequencing is based on software interpretation of the changes in electrical voltage as a DNA molecule is passed through a synthetic protein pore in a membrane. This strategy is known to have issues of sequence accuracy of homopolymeric regions [25]. Indeed, several consensus sequences, particularly for the PB1 and PA segments, were found to have interrupted open reading frames using automated sequence assembly workflows (Table 4). When the reads in the region near the premature termination codon were examined, a frameshift caused by missing a nucleotide in a run of homopolymers was frequently found. For the MinION platform, 60-fold or higher read coverage has been found to result in a better than 99.95% sequencing accuracy [29,30]. In our study, Method K resulted in the identical sequence in two samples (245467 and 246038) when compared to the sequence generated from the same samples on the MiSeq (Supplementary Figures S1a–h and S3a–h). Two sequence differences were noted in Sample 245626, and this corresponds to an accuracy of 99.985% (Supplementary Figure S2a–h). A similar degree of sequence accuracy (>99%) between MinION and MiSeq was found by Wang et al. [31].

Both Method A and Method K were able to generate the complete genomes from all three test samples. Method A follows the classic process of RT-PCR followed by native barcoding using ONT’s SQK-LSK109 kit. This process has been previously described [16]. We found four sequence deviations from the MiSeq reference sequence using Method A. In addition to the one common sequence variation in PB2 as discussed above, three other departures were found in the PA gene for Sample 245626. None of these variations were found in the genomes assembled with the other kits. Method K (ONT SQK-RBP004) utilizes an abbreviated PCR step to amplify and barcode samples. The procedure only has two bead purification steps, only one of which is kit specific. Manual bead purification is not only labor intensive, but products are often lost during purification; so, minimizing the bead purification steps meant that Method K was the second fastest among the kits evaluated. This may have contributed to a more evenly mapped read coverage over each segment than those of the other kits (Supplementary Figures S2–S4a–h).

## 5. Conclusions

We evaluated four primer sets for the whole-genome amplification of avian influenza viruses and tested several library preparation kits for the multiplexed sequencing of the samples using a nanopore next-generation sequencing platform. Primer Set 4 and Methods A and K had the best performances, as indicated by the generation of more cDNA, increased number of reads, more even genome coverage, and fewer sequencing errors, with Method K requiring less hands-on time. Further studies on the optimization of these and other kits could help to improve the ability to rapidly characterize the genomes of avian influenza viruses for the study of virus phylogeny and to inform avian influenza outbreak response.

## Figures and Tables

**Table 1:** Highly pathogenic H5N1 avian influenza virus-containing samples used in this study.

Sample ID	Animal Information	State	Collection Date	Sample Type	Matrix Ct value	GISAID ID
245467	Neotropical Cormorant	Arizona	23/5/22	Cloacal Swab	25.18	EPI_ISL_16555203
245626	Eared Grebe	North Dakota	16/6/22	Tracheal Swab	30.51	EPI_ISL_16555204
246038	Canada Goose	California	5/7/22	Tracheal Swab	16.44	EPI_ISL_16555205

**Table 2:** Primer sets for avian influenza virus whole-genome amplification. Underlined sequences correspond to conserved sequences in influenza A viruses.

Primer name	Nucleotide Sequence	Reference	Used in Method
Set 1. 080304(Uni12/Inf1)	5’-GGGGGGAGCAAAAGCAGG-3’	[13]	A
Set 1. 080305(Uni12/Inf3)	5’-GGGGGGAGCGAAAGCAGG-3’	[13]	″
Set 1. 080306(Uni13/Inf1)	5’-GGGGTTATTAGTAGAAACAAGG-3’	[13]	″
Set 2. 080307(PSK004/Uni12)	5’-TTTCTGTTGGTGCTGATATTGCGATCAGCAAAAGCAGG-3’	This study	S
Set 2. 080308(PSK004/Uni12.4)	5’-TTTCTGTTGGTGCTGATATTGCGATCAGCGAAAGCAGG-3’	This study	″
Set 2. 080309(PSK004/Uni13)	5’-ACTTGCCTGTCGCTCTATCTTCGATCAGTAGAAACAAGG-3’	This study	″
Set 3. 969501(Pan-IVA-1F)	5’-TCCCAGTCACGACGTCGTAGCGAAAGCAGG-3’	[20]	E
Set 3. 969502(Pan-IVA-1F)	5’-GGAAACAGCTATGACCATGAGTAGAAACAAGG-3’	[20]	″
Set 4. 1071804(MitchellBUni12)	5’-TTTCTGTTGGTGCTGATATTGTTACGCGCCAGCAAAAGCAGG-3’	Modified after [23]	K, N
Set 4. 1071805(MitchellBUni12.4)	5’-TTTCTGTTGGTGCTGATATTGTTACGCGCCAGCGAAAGCAGG-3’	Modified after [23]	″
Set 4. 1071806(MitchellBUni13)	5’-ACTTGCCTGTCGCTCTATCTTCGTTACGCGCCAGTAGAAACAAGG-3’	Modified after [23]	″

**Table 3:** Overall reads generated by the ONT kits evaluated. The total number of reads generated between the three RNA samples, the number of reads mapped to the H5N1 genomes, and the percentage of reads mapped to H5N1 are presented.

Method	Method A	Method S	Method E	Method K	Method N
Total # reads	4,910,043	9,433,118	2,896,000	5,498,527	1,721,746
# mapped reads	3,899,374	8,275,317	1,982,488	4,854,536	1,525,701
% mapped reads	87.7%	87.7%	68.5%	88.3%	88.6%

**Table 4:** Summary of reads generated by each method with RNA sample 246038 that mapped to HPAI H5N1. AIV segments, Avian influenza virus (AIV) RNA segments; Methods A–N, the number of reads mapped to each of the viral RNA segments by each method; Total # reads, the total number of mapped viral reads generated for each method; CDS, whether the complete coding region was assembled automatically (Auto) by the bioinformatic pipeline, or Edited, if the complete coding region was obtained following manual editing, or Partial, if the coding region was incomplete.

AIV Segments	Method A	CDS	Method S	CDS	Method E	CDS	Method K	CDS	Method N	CDS
PB2	15,698	Edited	2,289,167	Partial	7476	Partial	142,151	Edited	695	Edited
PB1	63,842	Edited	1,101,735	Partial	551	Partial	26,142	Auto	734	Auto
PA	1489	Edited	94,586	Partial	2669	Partial	342,122	Edited	1064	Auto
H5	83,360	Auto	5775	Partial	16	Partial	344,541	Auto	5127	Auto
NP	32,937	Auto	45	Partial	7	Partial	127,815	Auto	2930	Auto
N1	46,701	Auto	3408	Auto	10	Partial	25,004	Auto	7144	Auto
MA	204,565	Auto	91,729	Auto	18	Partial	389,719	Auto	262,346	Auto
NS	435,686	Auto	67,187	Auto	12	Partial	473,247	Auto	101,586	Auto
Total # reads	884.278		3,653,632		10,759		1,870,741		381,626	

**Table 5:** The total number of sequence variations across the virus genome per kit. The single nucleotide variations generated by each kit when evaluated against the reference sequence generated on the Illumina MiSeq are listed; del7; a deletion of seven nucleotides.

Kits	No. Mutations
Method A	4
Method S	1
Method E	190
Method K	2
Method N	4 + del7

## Data Availability

Data associated with this project are available at: [32].

## Supplementary Materials

The following supporting information can be downloaded at https://www.mdpi.com/article/10.3390/microorganisms11020529/s1: Supplementary Figure S1a–h. Sequence variations generated by the different methods tested for Sample 245467; Supplementary Figure S2a–h. Sequence variations generated by the different methods tested for Sample 245627; Supplementary Figure S3a–h. Sequence variations generated by the different methods tested of Sample 246038; Supplementary Table S1: Summary of key steps in the methods evaluated; Supplementary Table S2a–c: The number of sequence variations in the final consensus sequence in each RNA segment by method.
