# Supplementary material for: An Evaluation of Avian Influenza Virus Whole-Genome Sequencing Approaches Using Nanopore Technology
Source: Microorganisms. 2023 Feb 19;11(2):529. doi: 10.3390/microorganisms11020529 (PMC9967579; doi:10.3390/microorganisms11020529)
Supplement: Supplementary file 1 [file microorganisms-11-00529-s001.zip › manuscript.v8 230219 Suppl Figures and Tables/Supplementary Table S2b.pdf]

245626	PB2	PB1	PA	H5	NP	N1	MA	NS
**Method A**	1	-	3	-	-	-	-	-
**Method S**	ND	ND	ND	ND	ND	ND	ND	ND
**Method E**	2	-	-	-	-	-	-	-
**Method K**	1	-	-	-	-	1	-	-
**Method N**	1	2	1	-	-	-	-	-
