# Supplementary material for: An Evaluation of Avian Influenza Virus Whole-Genome Sequencing Approaches Using Nanopore Technology
Source: Microorganisms. 2023 Feb 19;11(2):529. doi: 10.3390/microorganisms11020529 (PMC9967579; doi:10.3390/microorganisms11020529)
Supplement: Supplementary file 1 [file microorganisms-11-00529-s001.zip › manuscript.v8 230219 Suppl Figures and Tables/Supplementary Table S1.pdf]

	Method A	Method S	Method E	Method K	Method N
Description	Native barcoding	PCR barcoding	Rapid barcoding	Rapid PCR barcoding	Native barcoding
Kit name	SQK-LSK109	SQK-PBK004	SQK-RBK004	SQK-RBP004	SQK-LSK109 (lo-Cost)
**Step**	**RT-PCR**	**RT-PCR**	**RT-PCR**	**RT-PCR**	**RT-PCR**
Kit	SuperScript III Platinum	SuperScript III Platinum	SuperScript III Platinum	SuperScript III Platinum	SuperScript III Platinum
Primer sets used	Set 1	Set 2	Set 3	Set 4	Set 4
Cycling conditions	[13]	[13]	[20]	[28]	[13]
Time (min) incl bead purification	268	268	233	278	278
**Step**	**End Prep**				**End Prep**
Kit	NEBNext FFPE & Ultra II				NEBNext Ultra II
Time (min) incl bead purification	23				10
**Step**	**Barcoding**	**PCR Barcoding**	**Fragmentation and Barcoding**	**Rapid PCR Barcoding**	**Barcoding**
Kit	NEB Blunt/TA ligase	NEB LongAmp Hot Start Taq	ONT Fragmentation Mix	NEB LongAmp Hot Start Taq	NEB Blunt/TA ligase
Time (min) incl bead purification	23	223	13	37.5	45
**Step**	**Adaptor Ligation**	**RAP**	**RAP**	**RAP**	**Adaptor Ligation**
Kit	NEB Quick T4 DNA Ligase	ONT Rapid Adaptor	ONT Rapid Adaptor	ONT Rapid Adaptor	NEB Quick T4 DNA Ligase
Time (min) incl bead purification	23	5	5	5	23
**# Steps**	**4**	**3**	**3**	**3**	**4**
**# bead purifications**	**4**	**2**	**2**	**2**	**3**
**Total hands-on time (h)**	**1.2**	**3.8**	**0.3**	**0.7**	**1.3**
**Total prep time (h)**	**5.6**	**8.3**	**4.2**	**5.3**	**5.9**
