# Supplementary material for: An Evaluation of Avian Influenza Virus Whole-Genome Sequencing Approaches Using Nanopore Technology
Source: Microorganisms. 2023 Feb 19;11(2):529. doi: 10.3390/microorganisms11020529 (PMC9967579; doi:10.3390/microorganisms11020529)
Supplement: Supplementary file 1 [file microorganisms-11-00529-s001.zip › manuscript.v8 230219 Suppl Figures and Tables/Supplementary Figures S3a-h 246038/Supplementary Figure S3c PA.pdf]

Formatted Alignments

**PA 246038 MiSeq**	*1*	ATGGAAGACTTTGTGCGACAATGCTTCAATCCAATGATTGTCGAGCTTGCGGAAAAAGCA	*60*
**PA 246038 Method A**	*1*	ATGGAAGACTTTGTGCGACAATGCTTCAATCCAATGATTGTCGAGCTTGCGGAAAAAGCA	*60*
**PA 246038 Method S**	*1*	ATGGAAGACTTTGTGCGACAATGCTTCAATCCAATGATTGTCGAGCTTGCGGAAAAAGCA	*60*
**PA 246038 Method E**	*1*	ATGGAAGACTTTGTGCGACAATGCTTCAATCCAATGATTGTCGAGCTTGCGGAAAAAGCA	*60*
**PA 246038 Method K**	*1*	ATGGAAGACTTTGTGCGACAATGCTTCAATCCAATGATTGTCGAGCTTGCGGAAAAAGCA	*60*
**PA 246038 Method N**	*1*	ATGGAAGACTTTGTGCGACAATGCTTCAATCCAATGATTGTCGAGCTTGCGGAAAAAGCA	*60*

**PA 246038 MiSeq**	*61*	ATGAAAGAATATGGGGAAGATCCGAAAATCGAGACAAACAAATTTGCCGCAATATGCACA	*120*
**PA 246038 Method A**	*61*	ATGAAAGAATATGGGGAAGATCCGAAAATCGAGACAAACAAATTTGCCGCAATATGCACA	*120*
**PA 246038 Method S**	*61*	ATGAAAGAATATGGGGAAGATCCGAAAATCGAGACAAACAAATTTGCCGCAATATGCACA	*120*
**PA 246038 Method E**	*61*	ATGAAAGAATATGGGGAAGATCCGAAAATCGAGACAAACAAATTTGCCGCAATATGCACA	*120*
**PA 246038 Method K**	*61*	ATGAAAGAATATGGGGAAGATCCGAAAATCGAGACAAACAAATTTGCCGCAATATGCACA	*120*
**PA 246038 Method N**	*61*	ATGAAAGAATATGGGGAAGATCCGAAAATCGAGACAAACAAATTTGCCGCAATATGCACA	*120*

**PA 246038 MiSeq**	*121*	CACTTAGAAGTCTGTTTCATGTATTCGGATTTCCATTTTATTGATGAACGAGGCGAATCA	*180*
**PA 246038 Method A**	*121*	CACTTAGAAGTCTGTTTCATGTATTCGGATTTCCATTTTATTGATGAACGAGGCGAATCA	*180*
**PA 246038 Method S**	*121*	CACTTAGAAGTCTGTTTCATGTATTCGGATTTCCATTTTATTGATGAACGAGGCGAATCA	*180*
**PA 246038 Method E**	*121*	CACTTAGAAGTCTGTTTCATGTATTCGGATTTCCATTTTATTGATGAACGAGGCGAATCA	*180*
**PA 246038 Method K**	*121*	CACTTAGAAGTCTGTTTCATGTATTCGGATTTCCATTTTATTGATGAACGAGGCGAATCA	*180*
**PA 246038 Method N**	*121*	CACTTAGAAGTCTGTTTCATGTATTCGGATTTCCATTTTATTGATGAACGAGGCGAATCA	*180*

**PA 246038 MiSeq**	*181*	ATGATTGTAGAATCTGGGGATCCAAATGCATTATTGAAACACCGATTTGAGATAATCGAA	*240*
**PA 246038 Method A**	*181*	ATGATTGTAGAATCTGGGGATCCAAATGCATTATTGAAACACCGATTTGAGATAATCGAA	*240*
**PA 246038 Method S**	*181*	ATGATTGTAGAATCTGGGGATCCAAATGCATTATTGAAACACCGATTTGAGATAATCGAA	*240*
**PA 246038 Method E**	*181*	ATGATTGTAGAATCTGGGGATCCAAATGCATTATTGAAACACCGATTTGAGATAATCGAA	*240*
**PA 246038 Method K**	*181*	ATGATTGTAGAATCTGGGGATCCAAATGCATTATTGAAACACCGATTTGAGATAATCGAA	*240*
**PA 246038 Method N**	*181*	ATGATTGTAGAATCTGGGGATCCAAATGCATTATTGAAACACCGATTTGAGATAATCGAA	*240*

**PA 246038 MiSeq**	*241*	GGGAGAGACCGAGCAATGGCCTGGACAGTGGTGAATAGTATCTGCAACACCACAGGGGTC	*300*
**PA 246038 Method A**	*241*	GGGAGAGACCGAGCAATGGCCTGGACAGTGGTGAATAGTATCTGCAACACCACAGGGGTC	*300*
**PA 246038 Method S**	*241*	GGGAGAGACCGAGCAATGGCCTGGACAGTGGTGAATAGTATCTGCAACACCACAGGGGTC	*300*
**PA 246038 Method E**	*241*	GGGAGAGACCGAGCAATGGCCTGGACAGTGGTGAATAGTATCTGCAACACCACAGGGGTC	*300*
**PA 246038 Method K**	*241*	GGGAGAGACCGAGCAATGGCCTGGACAGTGGTGAATAGTATCTGCAACACCACAGGGGTC	*300*
**PA 246038 Method N**	*241*	GGGAGAGACCGAGCAATGGCCTGGACAGTGGTGAATAGTATCTGCAACACCACAGGGGTC	*300*

**PA 246038 MiSeq**	*301*	GAAAAGCCCAAATTCCTCCCTGATTTGTATGACTACAAAGAGAACCGATTCATTGAAATT	*360*
**PA 246038 Method A**	*301*	GAAAAGCCCAAATTCCTCCCTGATTTGTATGACTACAAAGAGAACCGATTCATTGAAATT	*360*
**PA 246038 Method S**	*301*	GAAAAGCCCAAATTCCTCCCTGATTTGTATGACTACAAAGAGAACCGATTCATTGAAATT	*360*
**PA 246038 Method E**	*301*	GAAAAGCCCAAATTCCTCCCTGATTTGTATGACTACAAAGAGAACCGATTCATTGAAATT	*360*
**PA 246038 Method K**	*301*	GAAAAGCCCAAATTCCTCCCTGATTTGTATGACTACAAAGAGAACCGATTCATTGAAATT	*360*
**PA 246038 Method N**	*301*	GAAAAGCCCAAATTCCTCCCTGATTTGTATGACTACAAAGAGAACCGATTCATTGAAATT	*360*

**PA 246038 MiSeq**	*361*	GGAGTAACGCGAAGGGAAGTTCACATATACTATTTAGAAAAAGCCAACAAGATAAAATCA	*420*
**PA 246038 Method A**	*361*	GGAGTAACGCGAAGGGAAGTTCACATATACTATTTAGAAAAAGCCAACAAGATAAAATCA	*420*
**PA 246038 Method S**	*361*	GGAGTAACGCGAAGGGAAGTTCACATATACTATTTAGAAAAAGCCAACAAGATAAAATCA	*420*
**PA 246038 Method E**	*361*	GGAGTAACGCGAAGGGAAGTTCACATATACTATTTAGAAAAAGCCAACAAGATAAAATCA	*420*
**PA 246038 Method K**	*361*	GGAGTAACGCGAAGGGAAGTTCACATATACTATTTAGAAAAAGCCAACAAGATAAAATCA	*420*
**PA 246038 Method N**	*361*	GGAGTAACGCGAAGGGAAGTTCACATATACTATTTAGAAAAAGCCAACAAGATAAAATCA	*420*

**PA 246038 MiSeq**	*421*	GAGAAAACACATATTCACATATTCTCATTCACTGGAGAGGAAATGGCCACCAAGGCGGAC	*480*
**PA 246038 Method A**	*421*	GAGAAAACACATATTCACATATTCTCATTCACTGGAGAGGAAATGGCCACCAAGGCGGAC	*480*
**PA 246038 Method S**	*421*	GAGAAAACACATATTCACATATTCTCATTCACTGGAGAGGAAATGGCCACCAAGGCGGAC	*480*
**PA 246038 Method E**	*421*	GAGAAAACACATATTCACATATTCTCATTCACTGGAGAGGAAATGGCCACCAAGGCGGAC	*480*
**PA 246038 Method K**	*421*	GAGAAAACACATATTCACATATTCTCATTCACTGGAGAGGAAATGGCCACCAAGGCGGAC	*480*
**PA 246038 Method N**	*421*	GAGAAAACACATATTCACATATTCTCATTCACTGGAGAGGAAATGGCCACCAAGGCGGAC	*480*

**PA 246038 MiSeq**	*481*	TACACCCTTGATGAAGAGAGCAGAGCAAGAATAAAAACCAGACTGTTCACTATAAGACAA	*540*
**PA 246038 Method A**	*481*	TACACCCTTGATGAAGAGAGCAGAGCAAGAATAAAAACCAGACTGTTCACTATAAGACAA	*540*
**PA 246038 Method S**	*481*	TACACCCTTGATGAAGAGAGCAGAGCAAGAATAAAAACCAGACTGTTCACTATAAGACAA	*540*
**PA 246038 Method E**	*481*	TACACCCTTGATGAAGAGAGCAGAGCAAGAATAAAAACCAGACTGTTCACTATAAGACAA	*540*
**PA 246038 Method K**	*481*	TACACCCTTGATGAAGAGAGCAGAGCAAGAATAAAAACCAGACTGTTCACTATAAGACAA	*540*
**PA 246038 Method N**	*481*	TACACCCTTGATGAAGAGAGCAGAGCAAGAATAAAAACCAGACTGTTCACTATAAGACAA	*540*

**PA 246038 MiSeq**	*541*	GAAATGGCCAGTAGAGGTCTATGGGATTCCTTTCGTCAGTCCGAGAGAGGCGAAGAGACA	*600*
**PA 246038 Method A**	*541*	GAAATGGCCAGTAGAGGTCTATGGGATTCCTTTCGTCAGTCCGAGAGAGGCGAAGAGACA	*600*
**PA 246038 Method S**	*541*	GAAATGGCCAGTAGAGGTCTATGGGATTCCTTTCGTCAGTCCGAGAGAGGCGAAGAGACA	*600*
**PA 246038 Method E**	*541*	GAAATGGCCAGTAGAGGTCTATGGGATTCCTTTCGTCAGTCCGAGAGAGGCGAAGAGACA	*600*
**PA 246038 Method K**	*541*	GAAATGGCCAGTAGAGGTCTATGGGATTCCTTTCGTCAGTCCGAGAGAGGCGAAGAGACA	*600*
**PA 246038 Method N**	*541*	GAAATGGCCAGTAGAGGTCTATGGGATTCCTTTCGTCAGTCCGAGAGAGGCGAAGAGACA	*600*

**PA 246038 MiSeq**	*601*	ATTGAAGAAAGATTTGAAATCACAGGAACCATGCGCAGGCTTGCCGACCAAAGTCTTCCA	*660*
**PA 246038 Method A**	*601*	ATTGAAGAAAGATTTGAAATCACAGGAACCATGCGCAGGCTTGCCGACCAAAGTCTTCCA	*660*
**PA 246038 Method S**	*601*	ATTGAAGAAAGATTTGAAATCACAGGAACCATGCGCAGGCTTGCCGACCAAAGTCTTCCA	*660*
**PA 246038 Method E**	*601*	ATTGAAGAAAGATTTGAAATCACAGGAACCATGCGCAGGCTTGCCGACCAAAGTCTTCCA	*660*
**PA 246038 Method K**	*601*	ATTGAAGAAAGATTTGAAATCACAGGAACCATGCGCAGGCTTGCCGACCAAAGTCTTCCA	*660*
**PA 246038 Method N**	*601*	ATTGAAGAAAGATTTGAAATCACAGGAACCATGCGCAGGCTTGCCGACCAAAGTCTTCCA	*660*

**PA 246038 MiSeq**	*661*	CCGAACTTCTCCAGCCTTGAAAACTTTAGAGCCTATGTGGATGGATTCGAACCGAACGGC	*720*
**PA 246038 Method A**	*661*	CCGAACTTCTCCAGCCTTGAAAACTTTAGAGCCTATGTGGATGGATTCGAACCGAACGGC	*720*
**PA 246038 Method S**	*661*	CCGAACTTCTCCAGCCTTGAAAACTTTAGAGCCTATGTGGATGGATTCGAACCGAACGGC	*720*
**PA 246038 Method E**	*661*	CCGAACTTCTCCAGCCTTGAAAACTTTAGAGCCTATGTGGATGGATTCGAACCGAACGGC	*720*
**PA 246038 Method K**	*661*	CCGAACTTCTCCAGCCTTGAAAACTTTAGAGCCTATGTGGATGGATTCGAACCGAACGGC	*720*
**PA 246038 Method N**	*661*	CCGAACTTCTCCAGCCTTGAAAACTTTAGAGCCTATGTGGATGGATTCGAACCGAACGGC	*720*

**PA 246038 MiSeq**	*721*	TGCATTGAGGGCAAACTTTCTCAAATGTCAAAAGAAGTGAACGCCAGAATTGAGCCATTT	*780*
**PA 246038 Method A**	*721*	TGCATTGAGGGCAAACTTTCTCAAATGTCAAAAGAAGTGAACGCCAGAATTGAGCCATTT	*780*
**PA 246038 Method S**	*721*	TGCATTGAGGGCAAACTTTCTCAAATGTCAAAAGAAGTGAACGCCAGAATTGAGCCATTT	*780*
**PA 246038 Method E**	*721*	TGCATTGAGGGCAAACTTTCTCAAATGTCAAAAGAAGTGAACGCCAGAATTGAGCCATTT	*780*
**PA 246038 Method K**	*721*	TGCATTGAGGGCAAACTTTCTCAAATGTCAAAAGAAGTGAACGCCAGAATTGAGCCATTT	*780*
**PA 246038 Method N**	*721*	TGCATTGAGGGCAAACTTTCTCAAATGTCAAAAGAAGTGAACGCCAGAATTGAGCCATTT	*780*

**PA 246038 MiSeq**	*781*	CTGAAGACAACACCACGCCCTCTCAGATTACCTGATGGGCCTCCCTGTTCTCAGCGGTCG	*840*
**PA 246038 Method A**	*781*	CTGAAGACAACACCACGCCCTCTCAGATTACCTGATGGGCCTCCCTGTTCTCAGCGGTCG	*840*
**PA 246038 Method S**	*781*	CTGAAGACAACACCACGCCCTCTCAGATTACCTGATGGGCCTCCCTGTTCTCAGCGGTCG	*840*
**PA 246038 Method E**	*781*	CTGAAGACAACACCACGCCCTCTCAGATTACCTGATGGGCCTCCCTGTTCTCAGCGGTCG	*840*
**PA 246038 Method K**	*781*	CTGAAGACAACACCACGCCCTCTCAGATTACCTGATGGGCCTCCCTGTTCTCAGCGGTCG	*840*
**PA 246038 Method N**	*781*	CTGAAGACAACACCACGCCCTCTCAGATTACCTGATGGGCCTCCCTGTTCTCAGCGGTCG	*840*

**PA 246038 MiSeq**	*841*	AAGTTCTTGCTGATGGATGCCCTTAAGTTGAGCATCGAAGACCCTAGTCATGAGGGGGAG	*900*
**PA 246038 Method A**	*841*	AAGTTCTTGCTGATGGATGCCCTTAAGTTGAGCATCGAAGACCCTAGTCATGAGGGGGAG	*900*
**PA 246038 Method S**	*841*	AAGTTCTTGCTGATGGATGCCCTTAAGTTGAGCATCGAAGACCCTAGTCATGAGGGGGAG	*900*
**PA 246038 Method E**	*841*	AAGTTCTTGCTGATGGATGCCCTTAAGTTGAGCATCGAAGACCCTAGTCATGAGGGGGAG	*900*
**PA 246038 Method K**	*841*	AAGTTCTTGCTGATGGATGCCCTTAAGTTGAGCATCGAAGACCCTAGTCATGAGGGGGAG	*900*
**PA 246038 Method N**	*841*	AAGTTCTTGCTGATGGATGCCCTTAAGTTGAGCATCGAAGACCCTAGTCATGAGGGGGAG	*900*

**PA 246038 MiSeq**	*901*	GGCATACCGCTGTATGATGCAATCAAATGCATGAAGACATTTTTTGGCTGGAAAGAGCCC	*960*
**PA 246038 Method A**	*901*	GGCATACCGCTGTATGATGCAATCAAATGCATGAAGACATTTTTTGGCTGGAAAGAGCCC	*960*
**PA 246038 Method S**	*901*	GGCATACCGCTGTATGATGCAATCAAATGCATGAAGACATTTTTTGGCTGGAAAGAGCCC	*960*
**PA 246038 Method E**	*901*	GGCATACCGCTGTATGATGCAATCAAATGCATGAAGACATTTTTTGGCTGGAAAGAGCCC	*960*
**PA 246038 Method K**	*901*	GGCATACCGCTGTATGATGCAATCAAATGCATGAAGACATTTTTTGGCTGGAAAGAGCCC	*960*
**PA 246038 Method N**	*901*	GGCATACCGCTGTATGATGCAATCAAATGCATGAAGACATTTTTTGGCTGGAAAGAGCCC	*960*

**PA 246038 MiSeq**	*961*	AACATCGTAAAGCCGCATGAGAAAGGCATAAACCCTAATTACCTCCTGGCTTGGAAGCAG	*1020*
**PA 246038 Method A**	*961*	AACATCGTAAAGCCGCATGAGAAAGGCATAAACCCTAATTACCTCCTGGCTTGGAAGCAG	*1020*
**PA 246038 Method S**	*961*	AACATCGTAAAGCCGCATGAGAAAGGCATAAACCCTAATTACCTCCTGGCTTGGAAGCAG	*1020*
**PA 246038 Method E**	*961*	AACATCGTAAAGCCGCATGAGAAAGGCATAAACCCTAATTACCTCCTGGCTTGGAAGCAG	*1020*
**PA 246038 Method K**	*961*	AACATCGTAAAGCCGCATGAGAAAGGCATAAACCCTAATTACCTCCTGGCTTGGAAGCAG	*1020*
**PA 246038 Method N**	*961*	AACATCGTAAAGCCGCATGAGAAAGGCATAAACCCTAATTACCTCCTGGCTTGGAAGCAG	*1020*

**PA 246038 MiSeq**	*1021*	GTGCTGGCAGAACTTCAAGACATTGAAAATGAGGAGAAAATTCCAAAAACAAAGAACATG	*1080*
**PA 246038 Method A**	*1021*	GTGCTGGCAGAACTTCAAGACATTGAAAATGAGGAGAAAATTCCAAAAACAAAGAACATG	*1080*
**PA 246038 Method S**	*1021*	GTGCTGGCAGAACTTCAAGACATTGAAAATGAGGAGAAAATTCCAAAAACAAAGAACATG	*1080*
**PA 246038 Method E**	*1021*	GTGCTGGCAGAACTTCAAGACATTGAAAATGAGGAGAAAATTCCAAAAACAAAGAACATG	*1080*
**PA 246038 Method K**	*1021*	GTGCTGGCAGAACTTCAAGACATTGAAAATGAGGAGAAAATTCCAAAAACAAAGAACATG	*1080*
**PA 246038 Method N**	*1021*	GTGCTGGCAGAACTTCAAGACATTGAAAATGAGGAGAAAATTCCAAAAACAAAGAACATG	*1080*

**PA 246038 MiSeq**	*1081*	AAGAAAACAAGCCAATTGAAGTGGGCACTTGGTGAGAACATGGCTCCAGAAAAAGTGGAC	*1140*
**PA 246038 Method A**	*1081*	AAGAAAACAAGCCAATTGAAGTGGGCACTTGGTGAGAACATGGCTCCAGAAAAAGTGGAC	*1140*
**PA 246038 Method S**	*1081*	AAGAAAACAAGCCAATTGAAGTGGGCACTTGGTGAGAACATGGCTCCAGAAAAAGTGGAC	*1140*
**PA 246038 Method E**	*1081*	AAGAAAACAAGCCAATTGAAGTGGGCACTTGGTGAGAACATGGCTCCAGAAAAAGTGGAC	*1140*
**PA 246038 Method K**	*1081*	AAGAAAACAAGCCAATTGAAGTGGGCACTTGGTGAGAACATGGCTCCAGAAAAAGTGGAC	*1140*
**PA 246038 Method N**	*1081*	AAGAAAACAAGCCAATTGAAGTGGGCACTTGGTGAGAACATGGCTCCAGAAAAAGTGGAC	*1140*

**PA 246038 MiSeq**	*1141*	TTTGAGGACTGCAAAGATGTTAGCGATCTAAGACAGTACGACAGTGACGAACCAGAGTCT	*1200*
**PA 246038 Method A**	*1141*	TTTGAGGACTGCAAAGATGTTAGCGATCTAAGACAGTACGACAGTGACGAACCAGAGTCT	*1200*
**PA 246038 Method S**	*1141*	TTTGAGGACTGCAAAGATGTTAGCGATCTAAGACAGTACGACAGTGACGAACCAGAGTCT	*1200*
**PA 246038 Method E**	*1141*	TTTGAGGACTGCAAAGATGTTAGCGATCTAAGACAGTACGACAGTGACGAACCAGAGTCT	*1200*
**PA 246038 Method K**	*1141*	TTTGAGGACTGCAAAGATGTTAGCGATCTAAGACAGTACGACAGTGACGAACCAGAGTCT	*1200*
**PA 246038 Method N**	*1141*	TTTGAGGACTGCAAAGATGTTAGCGATCTAAGACAGTACGACAGTGACGAACCAGAGTCT	*1200*

**PA 246038 MiSeq**	*1201*	AGATCACTAGCAAGCTGGATTCAGAGTGAATTCAACAAGGCATGCGAACTGACAGATTCG	*1260*
**PA 246038 Method A**	*1201*	AGATCACTAGCAAGCTGGATTCAGAGTGAATTCAACAAGGCATGCGAACTGACAGATTCG	*1260*
**PA 246038 Method S**	*1201*	AGATCACTAGCAAGCTGGATTCAGAGTGAATTCAACAAGGCATGCGAACTGACAGATTCG	*1260*
**PA 246038 Method E**	*1201*	AGATCACTAGCAAGCTGGATTCAGAGTGAATTCAACAAGGCATGCGAACTGACAGATTCG	*1260*
**PA 246038 Method K**	*1201*	AGATCACTAGCAAGCTGGATTCAGAGTGAATTCAACAAGGCATGCGAACTGACAGATTCG	*1260*
**PA 246038 Method N**	*1201*	AGATCACTAGCAAGCTGGATTCAGAGTGAATTCAACAAGGCATGCGAACTGACAGATTCG	*1260*

**PA 246038 MiSeq**	*1261*	AGTTGGATTGAACTTGATGAGATAGGGGAAGACGTTGCTCCAATCGAACACATTGCGAGT	*1320*
**PA 246038 Method A**	*1261*	AGTTGGATTGAACTTGATGAGATAGGGGAAGACGTTGCTCCAATCGAACACATTGCGAGT	*1320*
**PA 246038 Method S**	*1261*	AGTTGGATTGAACTTGATGAGATAGGGGAAGACGTTGCTCCAATCGAACACATTGCGAGT	*1320*
**PA 246038 Method E**	*1261*	AGTTGGATTGAACTTGATGAGATAGGGGAAGACGTTGCTCCAATCGAACACATTGCGAGT	*1320*
**PA 246038 Method K**	*1261*	AGTTGGATTGAACTTGATGAGATAGGGGAAGACGTTGCTCCAATCGAACACATTGCGAGT	*1320*
**PA 246038 Method N**	*1261*	AGTTGGATTGAACTTGATGAGATAGGGGAAGACGTTGCTCCAATCGAACACATTGCGAGT	*1320*

**PA 246038 MiSeq**	*1321*	GTGAGGAGGAACTATTTCACAGCAGAGGTATCCCATTGCAGGGCCACTGAATACATAATG	*1380*
**PA 246038 Method A**	*1321*	GTGAGGAGGAACTATTTCACAGCAGAGGTATCCCATTGCAGGGCCACTGAATACATAATG	*1380*
**PA 246038 Method S**	*1321*	GTGAGGAGGAACTATTTCACAGCAGAGGTATCCCATTGCAGGGCCACTGAATACATAATG	*1380*
**PA 246038 Method E**	*1321*	GTGAGGAGGAACTATTTCACAGCAGAGGTATCCCATTGCAGGGCCACTGAATACATAATG	*1380*
**PA 246038 Method K**	*1321*	GTGAGGAGGAACTATTTCACAGCAGAGGTATCCCATTGCAGGGCCACTGAATACATAATG	*1380*
**PA 246038 Method N**	*1321*	GTGAGGAGGAACTATTTCACAGCAGAGGTATCCCATTGCAGGGCCACTGAATACATAATG	*1380*

**PA 246038 MiSeq**	*1381*	AAGGGAGTATACATAAACACAGCCCTATTGAATGCATCCTGTGCAGCCATGGATGATTTC	*1440*
**PA 246038 Method A**	*1381*	AAGGGAGTATACATAAACACAGCCCTATTGAATGCATCCTGTGCAGCCATGGATGATTTC	*1440*
**PA 246038 Method S**	*1381*	AAGGGAGTATACATAAACACAGCCCTATTGAATGCATCCTGTGCAGCCATGGATGATTTC	*1440*
**PA 246038 Method E**	*1381*	AAGGGAGTATACATAAACACAGCCCTATTGAATGCATCCTGTGCAGCCATGGATGATTTC	*1440*
**PA 246038 Method K**	*1381*	AAGGGAGTATACATAAACACAGCCCTATTGAATGCATCCTGTGCAGCCATGGATGATTTC	*1440*
**PA 246038 Method N**	*1381*	AAGGGAGTATACATAAACACAGCCCTATTGAATGCATCCTGTGCAGCCATGGATGATTTC	*1440*

**PA 246038 MiSeq**	*1441*	CAACTGATTCCAATGATAAGTAAGTGCAGAACTAAAGAAGGAAGACGGAAGACAAATCTG	*1500*
**PA 246038 Method A**	*1441*	CAACTGATTCCAATGATAAGTAAGTGCAGAACTAAAGAAGGAAGACGGAAGACAAATCTG	*1500*
**PA 246038 Method S**	*1441*	CAACTGATTCCAATGATAAGTAAGTGCAGAACTAAAGAAGGAAGACGGAAGACAAATCTG	*1500*
**PA 246038 Method E**	*1441*	CAACTGATTCCAATGATAAGTAAGTGCAGAACTAAAGAAGGAAGACGGAAGACAAATCTG	*1500*
**PA 246038 Method K**	*1441*	CAACTGATTCCAATGATAAGTAAGTGCAGAACTAAAGAAGGAAGACGGAAGACAAATCTG	*1500*
**PA 246038 Method N**	*1441*	CAACTGATTCCAATGATAAGTAAGTGCAGAACTAAAGAAGGAAGACGGAAGACAAATCTG	*1500*

**PA 246038 MiSeq**	*1501*	TATGGATTCATTATAAAAGGAAGATCCCATTTGAGGAATGACACCGATGTGGTAAACTTT	*1560*
**PA 246038 Method A**	*1501*	TATGGATTCATTATAAAAGGAAGATCCCATTTGAGGAATGACACCGATGTGGTAAACTTT	*1560*
**PA 246038 Method S**	*1501*	TATGGATTCATTATAAAAGGAAGATCCCATTTGAGGAATGACACCGATGTGGTAAACTTT	*1560*
**PA 246038 Method E**	*1501*	TATGGATTCATTATAAAAGGAAGATCCCATTTGAGGAATGACACCGATGTGGTAAACTTT	*1560*
**PA 246038 Method K**	*1501*	TATGGATTCATTATAAAAGGAAGATCCCATTTGAGGAATGACACCGATGTGGTAAACTTT	*1560*
**PA 246038 Method N**	*1501*	TATGGATTCATTATAAAAGGAAGATCCCATTTGAGGAATGACACCGATGTGGTAAACTTT	*1560*

**PA 246038 MiSeq**	*1561*	GTGAGCATGGAATTCTCTCTAACTGACCCGAGGCTAGAGCCACACAAATGGGAAAAGTAC	*1620*
**PA 246038 Method A**	*1561*	GTGAGCATGGAATTCTCTCTAACTGACCCGAGGCTAGAGCCACACAAATGGGAAAAGTAC	*1620*
**PA 246038 Method S**	*1561*	GTGAGCATGGAATTCTCTCTAACTGACCCGAGGCTAGAGCCACACAAATGGGAAAAGTAC	*1620*
**PA 246038 Method E**	*1561*	GTGAGCATGGAATTCTCTCTAACTGACCCGAGGCTAGAGCCACACAAATGGGAAAAGTAC	*1620*
**PA 246038 Method K**	*1561*	GTGAGCATGGAATTCTCTCTAACTGACCCGAGGCTAGAGCCACACAAATGGGAAAAGTAC	*1620*
**PA 246038 Method N**	*1561*	GTGAGCATGGAATTCTCTCTAACTGACCCGAGGCTAGAGCCACACAAATGGGAAAAGTAC	*1620*

**PA 246038 MiSeq**	*1621*	TGTGTTCTTGAGATAGGAGACATGCTCCTACGGACTGCAATAGGCCAAGTGTCGAGGCCC	*1680*
**PA 246038 Method A**	*1621*	TGTGTTCTTGAGATAGGAGACATGCTCCTACGGACTGCAATAGGCCAAGTGTCGAGGCCC	*1680*
**PA 246038 Method S**	*1621*	TGTGTTCTTGAGATAGGAGACATGCTCCTACGGACTGCAATAGGCCAAGTGTCGAGGCCC	*1680*
**PA 246038 Method E**	*1621*	TGTGTTCTTGAGATAGGAGACATGCTCCTACGGACTGCAATAGGCCAAGTGTCGAGGCCC	*1680*
**PA 246038 Method K**	*1621*	TGTGTTCTTGAGATAGGAGACATGCTCCTACGGACTGCAATAGGCCAAGTGTCGAGGCCC	*1680*
**PA 246038 Method N**	*1621*	TGTGTTCTTGAGATAGGAGACATGCTCCTACGGACTGCAATAGGCCAAGTGTCGAGGCCC	*1680*

**PA 246038 MiSeq**	*1681*	ATGTTCCTGTATGTGAGAACCAATGGGACTTCCAAGATCAAAATGAAATGGGGCATGGAG	*1740*
**PA 246038 Method A**	*1681*	ATGTTCCTGTATGTGAGAACCAATGGGACTTCCAAGATCAAAATGAAATGGGGCATGGAG	*1740*
**PA 246038 Method S**	*1681*	ATGTTCCTGTATGTGAGAACCAATGGGACTTCCAAGATCAAAATGAAATGGGGCATGGAG	*1740*
**PA 246038 Method E**	*1681*	ATGTTCCTGTATGTGAGAACCAATGGGACTTCCAAGATCAAAATGAAATGGGGCATGGAG	*1740*
**PA 246038 Method K**	*1681*	ATGTTCCTGTATGTGAGAACCAATGGGACTTCCAAGATCAAAATGAAATGGGGCATGGAG	*1740*
**PA 246038 Method N**	*1681*	ATGTTCCTGTATGTGAGAACCAATGGGACTTCCAAGATCAAAATGAAATGGGGCATGGAG	*1740*

**PA 246038 MiSeq**	*1741*	ATGAGGCGATGCCTTCTTCAGTCCCTTCAACAAATTGAGAGCATGATTGAGGCCGAATCT	*1800*
**PA 246038 Method A**	*1741*	ATGAGGCGATGCCTTCTTCAGTCCCTTCAACAAATTGAGAGCATGATTGAGGCCGAATCT	*1800*
**PA 246038 Method S**	*1741*	ATGAGGCGATGCCTTCTTCAGTCCCTTCAACAAATTGAGAGCATGATTGAGGCCGAATCT	*1800*
**PA 246038 Method E**	*1741*	ATGAGGCGATGCCTTCTTCAGTCCCTTCAACAAATTGAGAGCATGATTGAGGCCGAATCT	*1800*
**PA 246038 Method K**	*1741*	ATGAGGCGATGCCTTCTTCAGTCCCTTCAACAAATTGAGAGCATGATTGAGGCCGAATCT	*1800*
**PA 246038 Method N**	*1741*	ATGAGGCGATGCCTTCTTCAGTCCCTTCAACAAATTGAGAGCATGATTGAGGCCGAATCT	*1800*

**PA 246038 MiSeq**	*1801*	TCTGTCAAAGAGAAGGACATGTCCAAGGAATTCTTTGAAAACAAATCAGAAACATGGCCA	*1860*
**PA 246038 Method A**	*1801*	TCTGTCAAAGAGAAGGACATGTCCAAGGAATTCTTTGAAAACAAATCAGAAACATGGCCA	*1860*
**PA 246038 Method S**	*1801*	TCTGTCAAAGAGAAGGACATGTCCAAGGAATTCTTTGAAAACAAATCAGAAACATGGCCA	*1860*
**PA 246038 Method E**	*1801*	TCTGTCAAAGAGAAGGACATGTCCAAGGAATTCTTTGAAAACAAATCAGAAACATGGCCA	*1860*
**PA 246038 Method K**	*1801*	TCTGTCAAAGAGAAGGACATGTCCAAGGAATTCTTTGAAAACAAATCAGAAACATGGCCA	*1860*
**PA 246038 Method N**	*1801*	TCTGTCAAAGAGAAGGACATGTCCAAGGAATTCTTTGAAAACAAATCAGAAACATGGCCA	*1860*

**PA 246038 MiSeq**	*1861*	ATTGGAGAATCACCCAAAGGGGTGGAGGAAGGCTCTATTGGGAAAGTATGCAGAACATTG	*1920*
**PA 246038 Method A**	*1861*	ATTGGAGAATCACCCAAAGGGGTGGAGGAAGGCTCTATTGGGAAAGTATGCAGAACATTG	*1920*
**PA 246038 Method S**	*1861*	ATTGGAGAATCACCCAAAGGGGTGGAGGAAGGCTCTATTGGGAAAGTATGCAGAACATTG	*1920*
**PA 246038 Method E**	*1861*	ATTGGAGAATCACCCAAAGGGGTGGAGGAAGGCTCTATTGGGAAAGTATGCAGAACATTG	*1920*
**PA 246038 Method K**	*1861*	ATTGGAGAATCACCCAAAGGGGTGGAGGAAGGCTCTATTGGGAAAGTATGCAGAACATTG	*1920*
**PA 246038 Method N**	*1861*	ATTGGAGAATCACCCAAAGGGGTGGAGGAAGGCTCTATTGGGAAAGTATGCAGAACATTG	*1920*

**PA 246038 MiSeq**	*1921*	CTAGCAAAGTCTGTGTTCAACAGCCTATATGCATCTCCACAACTCGAGGGGTTTTCAGCT	*1980*
**PA 246038 Method A**	*1921*	CTAGCAAAGTCTGTGTTCAACAGCCTATATGCATCTCCACAACTCGAGGGGTTTTCAGCT	*1980*
**PA 246038 Method S**	*1921*	CTAGCAAAGTCTGTGTTCAACAGCCTATATGCATCTCCACAACTCGAGGGGTTTTCAGCT	*1980*
**PA 246038 Method E**	*1921*	CTAGCAAAGTCTGTGTTCAACAGCCTATATGCATCTCCACAACTCGAGGGGTTTTCAGCT	*1980*
**PA 246038 Method K**	*1921*	CTAGCAAAGTCTGTGTTCAACAGCCTATATGCATCTCCACAACTCGAGGGGTTTTCAGCT	*1980*
**PA 246038 Method N**	*1921*	CTAGCAAAGTCTGTGTTCAACAGCCTATATGCATCTCCACAACTCGAGGGGTTTTCAGCT	*1980*
**PA 246038 MiSeq**	*1981*	GAATCAAGAAAATTGCTTCTCATTGTTCAGGCACTTAGGGACAACCTGGAACCTGGAACC	*2040*
**PA 246038 Method A**	*1981*	GAATCAAGAAAATTGCTTCTCATTGTTCAGGCACTTAGGGACAACCTGGAACCTGGAACC	*2040*
**PA 246038 Method S**	*1981*	GAATCAAGAAAATTGCTTCTCATTGTTCAGGCACTTAGGGACAACCTGGAACCTGGAACC	*2040*
**PA 246038 Method E**	*1981*	GAATCAAGAAAATTGCTTCTCATTGTTCAGGCACTTAGGGACAACCTGGAACCTGGAACC	*2040*
**PA 246038 Method K**	*1981*	GAATCAAGAAAATTGCTTCTCATTGTTCAGGCACTTAGGGACAACCTGGAACCTGGAACC	*2040*
**PA 246038 Method N**	*1981*	GAATCAAGAAAATTGCTTCTCATTGTTCAGGCACTTAGGGACAACCTGGAACCTGGAACC	*2040*
**PA 246038 MiSeq**	*2041*	TTCGATCTTGGGGGGCTATATGAAGCAATTGAGGAGTGCCTGATTAACGATCCCTGGGTT	*2100*
**PA 246038 Method A**	*2041*	TTCGATCTTGGGGGGCTATATGAAGCAATTGAGGAGTGCCTGATTAACGATCCCTGGGTT	*2100*
**PA 246038 Method S**	*2041*	TTCGATCTTGGGGGGCTATATGAAGCAATTGAGGAGTGCCTGATTAACGATCCCTGGGTT	*2100*
**PA 246038 Method E**	*2041*	TTCGATCTTGGGGGGCTATATGAAGCAATTGAGGAGTGCCTGATTAACGATCCCTGGGTT	*2100*
**PA 246038 Method K**	*2041*	TTCGATCTTGGGGGGCTATATGAAGCAATTGAGGAGTGCCTGATTAACGATCCCTGGGTT	*2100*
**PA 246038 Method N**	*2041*	TTCGATCTTGGGGGGCTATATGAAGCAATTGAGGAGTGCCTGATTAACGATCCCTGGGTT	*2100*
**PA 246038 MiSeq**	*2101*	TTGCTTAATGCGTCTTGGTTCAACTCCTTCCTCACACATGCACTGAAATAG	*2151*
**PA 246038 Method A**	*2101*	TTGCTTAATGCGTCTTGGTTCAACTCCTTCCTCACACATGCACTGAAATAG	*2151*
**PA 246038 Method S**	*2101*	TTGCTTAATGCGTCTTGGTTCAACTCCTTCCTCACACATGCACTGAAATAG	*2151*
**PA 246038 Method E**	*2101*	TTGCTTAATGCGTCTTGGTTCAACTCCTTCCTCACACATGCACTGAAATAG	*2151*
**PA 246038 Method K**	*2101*	TTGCTTAATGCGTCTTGGTTCAACTCCTTCCTCACACATGCACTGAAATAG	*2151*
**PA 246038 Method N**	*2101*	TTGCTTAATGCGTCTTGGTTCAACTCCTTCCTCACACATGCACTGAAATAG	*2151*
