# Supplementary material for: An Evaluation of Avian Influenza Virus Whole-Genome Sequencing Approaches Using Nanopore Technology
Source: Microorganisms. 2023 Feb 19;11(2):529. doi: 10.3390/microorganisms11020529 (PMC9967579; doi:10.3390/microorganisms11020529)
Supplement: Supplementary file 1 [file microorganisms-11-00529-s001.zip › manuscript.v8 230219 Suppl Figures and Tables/Supplementary Figures S3a-h 246038/Supplementary Figure S3e NP.pdf]

Formatted Alignments

**NP 246038 MiSeq**	*1*	ATGGCGTCTCAAGGCACCAAACGATCCTATGAACAAATGGAAACTGGTGGGGAACGCCAG	*60*
**NP 246038 Method A**	*1*	ATGGCGTCTCAAGGCACCAAACGATCCTATGAACAAATGGAAACTGGTGGGGAACGCCAG	*60*
**NP 246038 Method S**	*1*	ATGGCGTCTCAAGGCACCAAACGATCCTATGAACAAATGGAAACTGGTGGGGAACGCCAG	*60*
**NP 246038 Method E**	*1*	ATGGCGTCTCAAGGCACCAAACGATCCTATGAACAAATGGAAACTGGTGGGGAACGCCAG	*60*
**NP 246038 Method K**	*1*	ATGGCGTCTCAAGGCACCAAACGATCCTATGAACAAATGGAAACTGGTGGGGAACGCCAG	*60*
**NP 246038 Method N**	*1*	ATGGCGTCTCAAGGCACCAAACGATCCTATGAACAAATGGAAACTGGTGGGGAACGCCAG	*60*

**NP 246038 MiSeq**	*61*	AATGCCACTGAAATCAGAGCATCTGTTGGAAGAATGGTTGGCGGAATCGGGAGATTCTAC	*120*
**NP 246038 Method A**	*61*	AATGCCACTGAAATCAGAGCATCTGTTGGAAGAATGGTTGGCGGAATCGGGAGATTCTAC	*120*
**NP 246038 Method S**	*61*	AATGCCACTGAAATCAGAGCATCTGTTGGAAGAATGGTTGGCGGAATCGGGAGATTCTAC	*120*
**NP 246038 Method E**	*61*	AATGCCACTGAAATCAGAGCATCTGTTGGAAGAATGGTTGGCGGAATCGGGAGATTCTAC	*120*
**NP 246038 Method K**	*61*	AATGCCACTGAAATCAGAGCATCTGTTGGAAGAATGGTTGGCGGAATCGGGAGATTCTAC	*120*
**NP 246038 Method N**	*61*	AATGCCACTGAAATCAGAGCATCTGTTGGAAGAATGGTTGGCGGAATCGGGAGATTCTAC	*120*

**NP 246038 MiSeq**	*121*	ATACAGATGTGCACTGAGCTCAAACTCAGTGATTACGAAGGGAGGCTGATCCAAAACAGC	*180*
**NP 246038 Method A**	*121*	ATACAGATGTGCACTGAGCTCAAACTCAGTGATTACGAAGGGAGGCTGATCCAAAACAGC	*180*
**NP 246038 Method S**	*121*	ATACAGATGTGCACTGAGCTCAAACTCAGTGATTACGAAGGGAGGCTGATCCAAAACAGC	*180*
**NP 246038 Method E**	*121*	ATACAGATGTGCACTGAGCTCAAACTCAGTGATTACGAAGGGAGGCTGATCCAAAACAGC	*180*
**NP 246038 Method K**	*121*	ATACAGATGTGCACTGAGCTCAAACTCAGTGATTACGAAGGGAGGCTGATCCAAAACAGC	*180*
**NP 246038 Method N**	*121*	ATACAGATGTGCACTGAGCTCAAACTCAGTGATTACGAAGGGAGGCTGATCCAAAACAGC	*180*

**NP 246038 MiSeq**	*181*	ATAACCATAGAAAGGATGGTTCTCTCGGCATTTGATGAGAGGAGGAACAAGTATCTGGAG	*240*
**NP 246038 Method A**	*181*	ATAACCATAGAAAGGATGGTTCTCTCGGCATTTGATGAGAGGAGGAACAAGTATCTGGAG	*240*
**NP 246038 Method S**	*181*	ATAACCATAGAAAGGATGGTTCTCTCGGCATTTGATGAGAGGAGGAACAAGTATCTGGAG	*240*
**NP 246038 Method E**	*181*	ATAACCATAGAAAGGATGGTTCTCTCGGCATTTGATGAGAGGAGGAACAAGTATCTGGAG	*240*
**NP 246038 Method K**	*181*	ATAACCATAGAAAGGATGGTTCTCTCGGCATTTGATGAGAGGAGGAACAAGTATCTGGAG	*240*
**NP 246038 Method N**	*181*	ATAACCATAGAAAGGATGGTTCTCTCGGCATTTGATGAGAGGAGGAACAAGTATCTGGAG	*240*

**NP 246038 MiSeq**	*241*	GAACATCCCAGTGCTGGGAAGGATCCCAAGAAGACTGGAGGTCCAATCTACAGGAGGAGA	*300*
**NP 246038 Method A**	*241*	GAACATCCCAGTGCTGGGAAGGATCCCAAGAAGACTGGAGGTCCAATCTACAGGAGGAGA	*300*
**NP 246038 Method S**	*241*	GAACATCCCAGTGCTGGGAAGGATCCCAAGAAGACTGGAGGTCCAATCTACAGGAGGAGA	*300*
**NP 246038 Method E**	*241*	GAACATCCCAGTGCTGGGAAGGATCCCAAGAAGACTGGAGGTCCAATCTACAGGAGGAGA	*300*
**NP 246038 Method K**	*241*	GAACATCCCAGTGCTGGGAAGGATCCCAAGAAGACTGGAGGTCCAATCTACAGGAGGAGA	*300*
**NP 246038 Method N**	*241*	GAACATCCCAGTGCTGGGAAGGATCCCAAGAAGACTGGAGGTCCAATCTACAGGAGGAGA	*300*

**NP 246038 MiSeq**	*301*	GATGGCAAATGGATGAGAGAGTTGATCCTTTACGACAAAGAAGAAATCAGAAGAATTTGG	*360*
**NP 246038 Method A**	*301*	GATGGCAAATGGATGAGAGAGTTGATCCTTTACGACAAAGAAGAAATCAGAAGAATTTGG	*360*
**NP 246038 Method S**	*301*	GATGGCAAATGGATGAGAGAGTTGATCCTTTACGACAAAGAAGAAATCAGAAGAATTTGG	*360*
**NP 246038 Method E**	*301*	GATGGCAAATGGATGAGAGAGTTGATCCTTTACGACAAAGAAGAAATCAGAAGAATTTGG	*360*
**NP 246038 Method K**	*301*	GATGGCAAATGGATGAGAGAGTTGATCCTTTACGACAAAGAAGAAATCAGAAGAATTTGG	*360*
**NP 246038 Method N**	*301*	GATGGCAAATGGATGAGAGAGTTGATCCTTTACGACAAAGAAGAAATCAGAAGAATTTGG	*360*

**NP 246038 MiSeq**	*361*	CGTCAAGCTAATAATGGAGAGGATGCCACTGCTGGTCTCACTCATTTGATGATTTGGCAT	*420*
**NP 246038 Method A**	*361*	CGTCAAGCTAATAATGGAGAGGATGCCACTGCTGGTCTCACTCATTTGATGATTTGGCAT	*420*
**NP 246038 Method S**	*361*	CGTCAAGCTAATAATGGAGAGGATGCCACTGCTGGTCTCACTCATTTGATGATTTGGCAT	*420*
**NP 246038 Method E**	*361*	CGTCAAGCTAATAATGGAGAGGATGCCACTGCTGGTCTCACTCATTTGATGATTTGGCAT	*420*
**NP 246038 Method K**	*361*	CGTCAAGCTAATAATGGAGAGGATGCCACTGCTGGTCTCACTCATTTGATGATTTGGCAT	*420*
**NP 246038 Method N**	*361*	CGTCAAGCTAATAATGGAGAGGATGCCACTGCTGGTCTCACTCATTTGATGATTTGGCAT	*420*

**NP 246038 MiSeq**	*421*	TCCAATCTGAATGATGCCACATACCAGAGAACAAGGGCACTTGTGCGTACTGGAATGGAC	*480*
**NP 246038 Method A**	*421*	TCCAATCTGAATGATGCCACATACCAGAGAACAAGGGCACTTGTGCGTACTGGAATGGAC	*480*
**NP 246038 Method S**	*421*	TCCAATCTGAATGATGCCACATACCAGAGAACAAGGGCACTTGTGCGTACTGGAATGGAC	*480*
**NP 246038 Method E**	*421*	TCCAATCTGAATGATGCCACATACCAGAGAACAAGGGCACTTGTGCGTACTGGAATGGAC	*480*
**NP 246038 Method K**	*421*	TCCAATCTGAATGATGCCACATACCAGAGAACAAGGGCACTTGTGCGTACTGGAATGGAC	*480*
**NP 246038 Method N**	*421*	TCCAATCTGAATGATGCCACATACCAGAGAACAAGGGCACTTGTGCGTACTGGAATGGAC	*480*

**NP 246038 MiSeq**	*481*	CCTAGGATGTGCTCTCTGATGCAAGGATCAACCCTCCCTAGGAGATCCGGGGCTGCTGGA	*540*
**NP 246038 Method A**	*481*	CCTAGGATGTGCTCTCTGATGCAAGGATCAACCCTCCCTAGGAGATCCGGGGCTGCTGGA	*540*
**NP 246038 Method S**	*481*	CCTAGGATGTGCTCTCTGATGCAAGGATCAACCCTCCCTAGGAGATCCGGGGCTGCTGGA	*540*
**NP 246038 Method E**	*481*	CCTAGGATGTGCTCTCTGATGCAAGGATCAACCCTCCCTAGGAGATCCGGGGCTGCTGGA	*540*
**NP 246038 Method K**	*481*	CCTAGGATGTGCTCTCTGATGCAAGGATCAACCCTCCCTAGGAGATCCGGGGCTGCTGGA	*540*
**NP 246038 Method N**	*481*	CCTAGGATGTGCTCTCTGATGCAAGGATCAACCCTCCCTAGGAGATCCGGGGCTGCTGGA	*540*
**NP 246038 MiSeq**	*541*	GCAGCAGTGAAAGGAGTTGGAACAATGGTGATGGAATTGATTCGGATGATCAAACGAGGG	*600*
**NP 246038 Method A**	*541*	GCAGCAGTGAAAGGAGTTGGAACAATGGTGATGGAATTGATTCGGATGATCAAACGAGGG	*600*
**NP 246038 Method S**	*541*	GCAGCAGTGAAAGGAGTTGGAACAATGGTGATGGAATTGATTCGGATGATCAAACGAGGG	*600*
**NP 246038 Method E**	*541*	GCAGCAGTGAAAGGAGTTGGAACAATGGTGATGGAATTGATTCGGATGATCAAACGAGGG	*600*
**NP 246038 Method K**	*541*	GCAGCAGTGAAAGGAGTTGGAACAATGGTGATGGAATTGATTCGGATGATCAAACGAGGG	*600*
**NP 246038 Method N**	*541*	GCAGCAGTGAAAGGAGTTGGAACAATGGTGATGGAATTGATTCGGATGATCAAACGAGGG	*600*
**NP 246038 MiSeq**	*601*	ATCAATGATCGGAATTTCTGGAGAGGCGAAAATGGACGGAGAACCAGGATTGCCTACGAG	*660*
**NP 246038 Method A**	*601*	ATCAATGATCGGAATTTCTGGAGAGGCGAAAATGGACGGAGAACCAGGATTGCCTACGAG	*660*
**NP 246038 Method S**	*601*	ATCAATGATCGGAATTTCTGGAGAGGCGAAAATGGACGGAGAACCAGGATTGCCTACGAG	*660*
**NP 246038 Method E**	*601*	ATCAATGATCGGAATTTCTGGAGAGGCGAAAATGGACGGAGAACCAGGATTGCCTACGAG	*660*
**NP 246038 Method K**	*601*	ATCAATGATCGGAATTTCTGGAGAGGCGAAAATGGACGGAGAACCAGGATTGCCTACGAG	*660*
**NP 246038 Method N**	*601*	ATCAATGATCGGAATTTCTGGAGAGGCGAAAATGGACGGAGAACCAGGATTGCCTACGAG	*660*
**NP 246038 MiSeq**	*661*	AGAATGTGCAACATCCTCAAGGGAAAGTTCCAAACAGCAGCACAACGAGCAATGATGGAT	*720*
**NP 246038 Method A**	*661*	AGAATGTGCAACATCCTCAAGGGAAAGTTCCAAACAGCAGCACAACGAGCAATGATGGAT	*720*
**NP 246038 Method S**	*661*	AGAATGTGCAACATCCTCAAGGGAAAGTTCCAAACAGCAGCACAACGAGCAATGATGGAT	*720*
**NP 246038 Method E**	*661*	AGAATGTGCAACATCCTCAAGGGAAAGTTCCAAACAGCAGCACAACGAGCAATGATGGAT	*720*
**NP 246038 Method K**	*661*	AGAATGTGCAACATCCTCAAGGGAAAGTTCCAAACAGCAGCACAACGAGCAATGATGGAT	*720*
**NP 246038 Method N**	*661*	AGAATGTGCAACATCCTCAAGGGAAAGTTCCAAACAGCAGCACAACGAGCAATGATGGAT	*720*

**NP 246038 MiSeq**	*721*	CAAGTGAGGGAAAGCCGGAATCCTGGAAATGCTGAGATTGAAGATCTCATCTTTCTCGCA	*780*
**NP 246038 Method A**	*721*	CAAGTGAGGGAAAGCCGGAATCCTGGAAATGCTGAGATTGAAGATCTCATCTTTCTCGCA	*780*
**NP 246038 Method S**	*721*	CAAGTGAGGGAAAGCCGGAATCCTGGAAATGCTGAGATTGAAGATCTCATCTTTCTCGCA	*780*
**NP 246038 Method E**	*721*	CAAGTGAGGGAAAGCCGGAATCCTGGAAATGCTGAGATTGAAGATCTCATCTTTCTCGCA	*780*
**NP 246038 Method K**	*721*	CAAGTGAGGGAAAGCCGGAATCCTGGAAATGCTGAGATTGAAGATCTCATCTTTCTCGCA	*780*
**NP 246038 Method N**	*721*	CAAGTGAGGGAAAGCCGGAATCCTGGAAATGCTGAGATTGAAGATCTCATCTTTCTCGCA	*780*

**NP 246038 MiSeq**	*781*	CGATCTGCTCTCATCCTGAGGGGATCAGTGGCTCATAAGTCCTGTCTGCCTGCTTGCGTG	*840*
**NP 246038 Method A**	*781*	CGATCTGCTCTCATCCTGAGGGGATCAGTGGCTCATAAGTCCTGTCTGCCTGCTTGCGTG	*840*
**NP 246038 Method S**	*781*	CGATCTGCTCTCATCCTGAGGGGATCAGTGGCTCATAAGTCCTGTCTGCCTGCTTGCGTG	*840*
**NP 246038 Method E**	*781*	CGATCTGCTCTCATCCTGAGGGGATCAGTGGCTCATAAGTCCTGTCTGCCTGCTTGCGTG	*840*
**NP 246038 Method K**	*781*	CGATCTGCTCTCATCCTGAGGGGATCAGTGGCTCATAAGTCCTGTCTGCCTGCTTGCGTG	*840*
**NP 246038 Method N**	*781*	CGATCTGCTCTCATCCTGAGGGGATCAGTGGCTCATAAGTCCTGTCTGCCTGCTTGCGTG	*840*

**NP 246038 MiSeq**	*841*	TATGGACTTGCTGTAGCCAGTGGATATGACTTTGAAAGAGAAGGATACTCTCTAGTCGGA	*900*
**NP 246038 Method A**	*841*	TATGGACTTGCTGTAGCCAGTGGATATGACTTTGAAAGAGAAGGATACTCTCTAGTCGGA	*900*
**NP 246038 Method S**	*841*	TATGGACTTGCTGTAGCCAGTGGATATGACTTTGAAAGAGAAGGATACTCTCTAGTCGGA	*900*
**NP 246038 Method E**	*841*	TATGGACTTGCTGTAGCCAGTGGATATGACTTTGAAAGAGAAGGATACTCTCTAGTCGGA	*900*
**NP 246038 Method K**	*841*	TATGGACTTGCTGTAGCCAGTGGATATGACTTTGAAAGAGAAGGATACTCTCTAGTCGGA	*900*
**NP 246038 Method N**	*841*	TATGGACTTGCTGTAGCCAGTGGATATGACTTTGAAAGAGAAGGATACTCTCTAGTCGGA	*900*

**NP 246038 MiSeq**	*901*	ATTGATCCTTTCCGTCTGCTCCAGAACAGTCAAGTCTTCAGTCTCATCAGACCGAACGAA	*960*
**NP 246038 Method A**	*901*	ATTGATCCTTTCCGTCTGCTCCAGAACAGTCAAGTCTTCAGTCTCATCAGACCGAACGAA	*960*
**NP 246038 Method S**	*901*	ATTGATCCTTTCCGTCTGCTCCAGAACAGTCAAGTCTTCAGTCTCATCAGACCGAACGAA	*960*
**NP 246038 Method E**	*901*	ATTGATCCTTTCCGTCTGCTCCAGAACAGTCAAGTCTTCAGTCTCATCAGACCGAACGAA	*960*
**NP 246038 Method K**	*901*	ATTGATCCTTTCCGTCTGCTCCAGAACAGTCAAGTCTTCAGTCTCATCAGACCGAACGAA	*960*
**NP 246038 Method N**	*901*	ATTGATCCTTTCCGTCTGCTCCAGAACAGTCAAGTCTTCAGTCTCATCAGACCGAACGAA	*960*

**NP 246038 MiSeq**	*961*	AATCCAGCTCATAAAAGTCAGCTGATATGGATGGCATGTCACTCTGCGGCATTTGAGGAT	*1020*
**NP 246038 Method A**	*961*	AATCCAGCTCATAAAAGTCAGCTGATATGGATGGCATGTCACTCTGCGGCATTTGAGGAT	*1020*
**NP 246038 Method S**	*961*	AATCCAGCTCATAAAAGTCAGCTGATATGGATGGCATGTCACTCTGCGGCATTTGAGGAT	*1020*
**NP 246038 Method E**	*961*	AATCCAGCTCATAAAAGTCAGCTGATATGGATGGCATGTCACTCTGCGGCATTTGAGGAT	*1020*
**NP 246038 Method K**	*961*	AATCCAGCTCATAAAAGTCAGCTGATATGGATGGCATGTCACTCTGCGGCATTTGAGGAT	*1020*
**NP 246038 Method N**	*961*	AATCCAGCTCATAAAAGTCAGCTGATATGGATGGCATGTCACTCTGCGGCATTTGAGGAT	*1020*

**NP 246038 MiSeq**	*1021*	CTGAGAGTGTCAAGCTTCATCAGAGGGACAAGAGTAGTCCCAAGAGGACAACTGTCCACC	*1080*
**NP 246038 Method A**	*1021*	CTGAGAGTGTCAAGCTTCATCAGAGGGACAAGAGTAGTCCCAAGAGGACAACTGTCCACC	*1080*
**NP 246038 Method S**	*1021*	CTGAGAGTGTCAAGCTTCATCAGAGGGACAAGAGTAGTCCCAAGAGGACAACTGTCCACC	*1080*
**NP 246038 Method E**	*1021*	CTGAGAGTGTCAAGCTTCATCAGAGGGACAAGAGTAGTCCCAAGAGGACAACTGTCCACC	*1080*
**NP 246038 Method K**	*1021*	CTGAGAGTGTCAAGCTTCATCAGAGGGACAAGAGTAGTCCCAAGAGGACAACTGTCCACC	*1080*
**NP 246038 Method N**	*1021*	CTGAGAGTGTCAAGCTTCATCAGAGGGACAAGAGTAGTCCCAAGAGGACAACTGTCCACC	*1080*

**NP 246038 MiSeq**	*1081*	AGAGGAGTCCAGATTGCTTCAAATGAAAACATGGAGACAATGGACTCCAGTACTCTTGAA	*1140*
**NP 246038 Method A**	*1081*	AGAGGAGTCCAGATTGCTTCAAATGAAAACATGGAGACAATGGACTCCAGTACTCTTGAA	*1140*
**NP 246038 Method S**	*1081*	AGAGGAGTCCAGATTGCTTCAAATGAAAACATGGAGACAATGGACTCCAGTACTCTTGAA	*1140*
**NP 246038 Method E**	*1081*	AGAGGAGTCCAGATTGCTTCAAATGAAAACATGGAGACAATGGACTCCAGTACTCTTGAA	*1140*
**NP 246038 Method K**	*1081*	AGAGGAGTCCAGATTGCTTCAAATGAAAACATGGAGACAATGGACTCCAGTACTCTTGAA	*1140*
**NP 246038 Method N**	*1081*	AGAGGAGTCCAGATTGCTTCAAATGAAAACATGGAGACAATGGACTCCAGTACTCTTGAA	*1140*

**NP 246038 MiSeq**	*1141*	CTGAGGAGCAGATACTGGGCTATAAGAACAAGAAGTGGAGGAAACACTAACCAACAGAGA	*1200*
**NP 246038 Method A**	*1141*	CTGAGGAGCAGATACTGGGCTATAAGAACAAGAAGTGGAGGAAACACTAACCAACAGAGA	*1200*
**NP 246038 Method S**	*1141*	CTGAGGAGCAGATACTGGGCTATAAGAACAAGAAGTGGAGGAAACACTAACCAACAGAGA	*1200*
**NP 246038 Method E**	*1141*	CTGAGGAGCAGATACTGGGCTATAAGAACAAGAAGTGGAGGAAACACTAACCAACAGAGA	*1200*
**NP 246038 Method K**	*1141*	CTGAGGAGCAGATACTGGGCTATAAGAACAAGAAGTGGAGGAAACACTAACCAACAGAGA	*1200*
**NP 246038 Method N**	*1141*	CTGAGGAGCAGATACTGGGCTATAAGAACAAGAAGTGGAGGAAACACTAACCAACAGAGA	*1200*

**NP 246038 MiSeq**	*1201*	GCATCTGCAGGACAAATCAGCGTACAGCCCACATTCTCTGTGCAGAGAAACCTCCCATTC	*1260*
**NP 246038 Method A**	*1201*	GCATCTGCAGGACAAATCAGCGTACAGCCCACATTCTCTGTGCAGAGAAACCTCCCATTC	*1260*
**NP 246038 Method S**	*1201*	GCATCTGCAGGACAAATCAGCGTACAGCCCACATTCTCTGTGCAGAGAAACCTCCCATTC	*1260*
**NP 246038 Method E**	*1201*	GCATCTGCAGGACAAATCAGCGTACAGCCCACATTCTCTGTGCAGAGAAACCTCCCATTC	*1260*
**NP 246038 Method K**	*1201*	GCATCTGCAGGACAAATCAGCGTACAGCCCACATTCTCTGTGCAGAGAAACCTCCCATTC	*1260*
**NP 246038 Method N**	*1201*	GCATCTGCAGGACAAATCAGCGTACAGCCCACATTCTCTGTGCAGAGAAACCTCCCATTC	*1260*

**NP 246038 MiSeq**	*1261*	GAGAGAGCAACCATCATGGCAGCATTTACGGGAAACACTGAAGGCAGAACTTCAGACATG	*1320*
**NP 246038 Method A**	*1261*	GAGAGAGCAACCATCATGGCAGCATTTACGGGAAACACTGAAGGCAGAACTTCAGACATG	*1320*
**NP 246038 Method S**	*1261*	GAGAGAGCAACCATCATGGCAGCATTTACGGGAAACACTGAAGGCAGAACTTCAGACATG	*1320*
**NP 246038 Method E**	*1261*	GAGAGAGCAACCATCATGGCAGCATTTACGGGAAACACTGAAGGCAGAACTTCAGACATG	*1320*
**NP 246038 Method K**	*1261*	GAGAGAGCAACCATCATGGCAGCATTTACGGGAAACACTGAAGGCAGAACTTCAGACATG	*1320*
**NP 246038 Method N**	*1261*	GAGAGAGCAACCATCATGGCAGCATTTACGGGAAACACTGAAGGCAGAACTTCAGACATG	*1320*

**NP 246038 MiSeq**	*1321*	AGAACTGAGATCATAAGGATGATGGAAAATGCCAGACCTGAAGATGTGTCTTTCCAGGGG	*1380*
**NP 246038 Method A**	*1321*	AGAACTGAGATCATAAGGATGATGGAAAATGCCAGACCTGAAGATGTGTCTTTCCAGGGG	*1380*
**NP 246038 Method S**	*1321*	AGAACTGAGATCATAAGGATGATGGAAAATGCCAGACCTGAAGATGTGTCTTTCCAGGGG	*1380*
**NP 246038 Method E**	*1321*	AGAACTGAGATCATAAGGATGATGGAAAATGCCAGACCTGAAGATGTGTCTTTCCAGGGG	*1380*
**NP 246038 Method K**	*1321*	AGAACTGAGATCATAAGGATGATGGAAAATGCCAGACCTGAAGATGTGTCTTTCCAGGGG	*1380*
**NP 246038 Method N**	*1321*	AGAACTGAGATCATAAGGATGATGGAAAATGCCAGACCTGAAGATGTGTCTTTCCAGGGG	*1380*

**NP 246038 MiSeq**	*1381*	CGGGGAGTCTTCGAGCTCTCGGACGAAAAGGCAACGAACCCGATCGTGCCTTCCTTTGAC	*1440*
**NP 246038 Method A**	*1381*	CGGGGAGTCTTCGAGCTCTCGGACGAAAAGGCAACGAACCCGATCGTGCCTTCCTTTGAC	*1440*
**NP 246038 Method S**	*1381*	CGGGGAGTCTTCGAGCTCTCGGACGAAAAGGCAACGAACCCGATCGTGCCTTCCTTTGAC	*1440*
**NP 246038 Method E**	*1381*	CGGGGAGTCTTCGAGCTCTCGGACGAAAAGGCAACGAACCCGATCGTGCCTTCCTTTGAC	*1440*
**NP 246038 Method K**	*1381*	CGGGGAGTCTTCGAGCTCTCGGACGAAAAGGCAACGAACCCGATCGTGCCTTCCTTTGAC	*1440*
**NP 246038 Method N**	*1381*	CGGGGAGTCTTCGAGCTCTCGGACGAAAAGGCAACGAACCCGATCGTGCCTTCCTTTGAC	*1440*

**NP 246038 MiSeq**	*1441*	ATGAGCAATGAAGGATCTTATTTCTTCGGAGACAATGCAGAGGAGTATGACAATTAA	*1497*
**NP 246038 Method A**	*1441*	ATGAGCAATGAAGGATCTTATTTCTTCGGAGACAATGCAGAGGAGTATGACAATTAA	*1497*
**NP 246038 Method S**	*1441*	ATGAGCAATGAAGGATCTTATTTCTTCGGAGACAATGCAGAGGAGTATGACAATTAA	*1497*
**NP 246038 Method E**	*1441*	ATGAGCAATGAAGGATCTTATTTCTTCGGAGACAATGCAGAGGAGTATGACAATTAA	*1497*
**NP 246038 Method K**	*1441*	ATGAGCAATGAAGGATCTTATTTCTTCGGAGACAATGCAGAGGAGTATGACAATTAA	*1497*
**NP 246038 Method N**	*1441*	ATGAGCAATGAAGGATCTTATTTCTTCGGAGACAATGCAGAGGAGTATGACAATTAA	*1497*
