# Supplementary material for: An Evaluation of Avian Influenza Virus Whole-Genome Sequencing Approaches Using Nanopore Technology
Source: Microorganisms. 2023 Feb 19;11(2):529. doi: 10.3390/microorganisms11020529 (PMC9967579; doi:10.3390/microorganisms11020529)
Supplement: Supplementary file 1 [file microorganisms-11-00529-s001.zip › manuscript.v8 230219 Suppl Figures and Tables/Supplementary Figures S3a-h 246038/Supplementary Figure S3d H5.pdf]

Formatted Alignments

H5 246038 MiSeq	1	ATGGAGAACATAGTACTACTTCTTGCAATAGTTAGCCTTGTTAAAAGTGATCAGATTTGC	60
H5 246038 Method A	1	ATGGAGAACATAGTACTACTTCTTGCAATAGTTAGCCTTGTTAAAAGTGATCAGATTTGC	60
H5 246038 Method S	1	ATGGAGAACATAGTACTACTTCTTGCAATAGTTAGCCTTGTTAAAAGTGATCAGATTTGC	60
H5 246038 Method E	1	ATGGAGAACATAGTACTACTTCTTGCAATAGTTAGCCTTGTTAAAAGTGATCAGATTTGC	60
H5 246038 Method K	1	ATGGAGAACATAGTACTACTTCTTGCAATAGTTAGCCTTGTTAAAAGTGATCAGATTTGC	60
H5 246038 Method N	1	ATGGAGAACATAGTACTACTTCTTGCAATAGTTAGCCTTGTTAAAAGTGATCAGATTTGC	60

H5 246038 MiSeq	61	ATTGGTTACCATGCAAACAATTCGACAGAGCAAGTTGACACGATAATGGAAAAGAACGTC	120
H5 246038 Method A	61	ATTGGTTACCATGCAAACAATTCGACAGAGCAAGTTGACACGATAATGGAAAAGAACGTC	120
H5 246038 Method S	61	ATTGGTTACCATGCAAACAATTCGACAGAGCAAGTTGACACGATAATGGAAAAGAACGTC	120
H5 246038 Method E	61	ATTGGTTACCATGCAAACAATTCGACAGAGCAAGTTGACACGATAATGGAAAAGAACGTC	120
H5 246038 Method K	61	ATTGGTTACCATGCAAACAATTCGACAGAGCAAGTTGACACGATAATGGAAAAGAACGTC	120
H5 246038 Method N	61	ATTGGTTACCATGCAAACAATTCGACAGAGCAAGTTGACACGATAATGGAAAAGAACGTC	120

H5 246038 MiSeq	121	ACTGTTACACATGCCCAAGACATACTGGAAAAAACACACAACGGGAAGCTCTGTGATCTA	180
H5 246038 Method A	121	ACTGTTACACATGCCCAAGACATACTGGAAAAAACACACAACGGGAAGCTCTGTGATCTA	180
H5 246038 Method S	121	ACTGTTACACATGCCCAAGACATACTGGAAAAAACACACAACGGGAAGCTCTGTGATCTA	180
H5 246038 Method E	121	ACTGTTACACATGCCCAAGACATACTGGAAAAAACACACAACGGGAAGCTCTGTGATCTA	180
H5 246038 Method K	121	ACTGTTACACATGCCCAAGACATACTGGAAAAAACACACAACGGGAAGCTCTGTGATCTA	180
H5 246038 Method N	121	ACTGTTACACATGCCCAAGACATACTGGAAAAAACACACAACGGGAAGCTCTGTGATCTA	180

H5 246038 MiSeq	181	AATGGGGTGAAGCCTCTGATTTTAAAGGATTGTAGTGTAGCTGGATGGCTCCTCGGAAAT	240
H5 246038 Method A	181	AATGGGGTGAAGCCTCTGATTTTAAAGGATTGTAGTGTAGCTGGATGGCTCCTCGGAAAT	240
H5 246038 Method S	181	AATGGGGTGAAGCCTCTGATTTTAAAGGATTGTAGTGTAGCTGGATGGCTCCTCGGAAAT	240
H5 246038 Method E	181	AATGGGGTGAAGCCTCTGATTTTAAAGGATTGTAGTGTAGCTGGATGGCTCCTCGGAAAT	240
H5 246038 Method K	181	AATGGGGTGAAGCCTCTGATTTTAAAGGATTGTAGTGTAGCTGGATGGCTCCTCGGAAAT	240
H5 246038 Method N	181	AATGGGGTGAAGCCTCTGATTTTAAAGGATTGTAGTGTAGCTGGATGGCTCCTCGGAAAT	240

**H5 246038 MiSeq**	*241*	CCAATGTGCGACGAATTCATCAGAGTGCCGGAATGGTCCTACATAGTGGAGCGGGCTAAC	*300*
**H5 246038 Method A**	*241*	CCAATGTGCGACGAATTCATCAGAGTGCCGGAATGGTCCTACATAGTGGAGCGGGCTAAC	*300*
**H5 246038 Method S**	*241*	CCAATGTGCGACGAATTCATCAGAGTGCCGGAATGGTCCTACATAGTGGAGCGGGCTAAC	*300*
**H5 246038 Method E**	*241*	CCAATGTGCGACGAATTCATCAGAGTGCCGGAATGGTCCTACATAGTGGAGCGGGCTAAC	*300*
**H5 246038 Method K**	*241*	CCAATGTGCGACGAATTCATCAGAGTGCCGGAATGGTCCTACATAGTGGAGCGGGCTAAC	*300*
**H5 246038 Method N**	*241*	CCAATGTGCGACGAATTCATCAGAGTGCCGGAATGGTCCTACATAGTGGAGCGGGCTAAC	*300*
**H5 246038 MiSeq**	*301*	CCAGCTAATGACCTCTGTTACCCAGGGAGCCTCAATGACTATGAAGAACTGAAACACATG	*360*
**H5 246038 Method A**	*301*	CCAGCTAATGACCTCTGTTACCCAGGGAGCCTCAATGACTATGAAGAACTGAAACACATG	*360*
**H5 246038 Method S**	*301*	CCAGCTAATGACCTCTGTTACCCAGGGAGCCTCAATGACTATGAAGAACTGAAACACATG	*360*
**H5 246038 Method E**	*301*	CCAGCTAATGACCTCTGTTACCCAGGGAGCCTCAATGACTATGAAGAACTGAAACACATG	*360*
**H5 246038 Method K**	*301*	CCAGCTAATGACCTCTGTTACCCAGGGAGCCTCAATGACTATGAAGAACTGAAACACATG	*360*
**H5 246038 Method N**	*301*	CCAGCTAATGACCTCTGTTACCCAGGGAGCCTCAATGACTATGAAGAACTGAAACACATG	*360*
**H5 246038 MiSeq**	*361*	TTGAGCAGAATAAATCATTTTGAGAAGATTCTGATCATCCCCAAGAGTTCCTGGCCAAAT	*420*
**H5 246038 Method A**	*361*	TTGAGCAGAATAAATCATTTTGAGAAGATTCTGATCATCCCCAAGAGTTCCTGGCCAAAT	*420*
**H5 246038 Method S**	*361*	TTGAGCAGAATAAATCATTTTGAGAAGATTCTGATCATCCCCAAGAGTTCCTGGCCAAAT	*420*
**H5 246038 Method E**	*361*	TTGAGCAGAATAAATCATTTTGAGAAGATTCTGATCATCCCCAAGAGTTCCTGGCCAAAT	*420*
**H5 246038 Method K**	*361*	TTGAGCAGAATAAATCATTTTGAGAAGATTCTGATCATCCCCAAGAGTTCCTGGCCAAAT	*420*
**H5 246038 Method N**	*361*	TTGAGCAGAATAAATCATTTTGAGAAGATTCTGATCATCCCCAAGAGTTCCTGGCCAAAT	*420*
**H5 246038 MiSeq**	*421*	CATGAAACATCACTAGGGGTGAGCGCAGCTTGTCCATACCAGGGAGCGCCCTCCTTTTTC	*480*
**H5 246038 Method A**	*421*	CATGAAACATCACTAGGGGTGAGCGCAGCTTGTCCATACCAGGGAGCGCCCTCCTTTTTC	*480*
**H5 246038 Method S**	*421*	CATGAAACATCACTAGGGGTGAGCGCAGCTTGTCCATACCAGGGAGCGCCCTCCTTTTTC	*480*
**H5 246038 Method E**	*421*	CATGAAACATCACTAGGGGTGAGCGCAGCTTGTCCATACCAGGGAGCGCCCTCCTTTTTC	*480*
**H5 246038 Method K**	*421*	CATGAAACATCACTAGGGGTGAGCGCAGCTTGTCCATACCAGGGAGCGCCCTCCTTTTTC	*480*
**H5 246038 Method N**	*421*	CATGAAACATCACTAGGGGTGAGCGCAGCTTGTCCATACCAGGGAGCGCCCTCCTTTTTC	*480*

**H5 246038 MiSeq**	*481*	AGAAATGTGGTGTGGCTTATCAAAAAGAACGATGCATACCCAACAATAAAGATAAGCTAC	*540*
**H5 246038 Method A**	*481*	AGAAATGTGGTGTGGCTTATCAAAAAGAACGATGCATACCCAACAATAAAGATAAGCTAC	*540*
**H5 246038 Method S**	*481*	AGAAATGTGGTGTGGCTTATCAAAAAGAACGATGCATACCCAACAATAAAGATAAGCTAC	*540*
**H5 246038 Method E**	*481*	AGAAATGTGGTGTGGCTTATCAAAAAGAACGATGCATACCCAACAATAAAGATAAGCTAC	*540*
**H5 246038 Method K**	*481*	AGAAATGTGGTGTGGCTTATCAAAAAGAACGATGCATACCCAACAATAAAGATAAGCTAC	*540*
**H5 246038 Method N**	*481*	AGAAATGTGGTGTGGCTTATCAAAAAGAACGATGCATACCCAACAATAAAGATAAGCTAC	*540*

**H5 246038 MiSeq**	*541*	AATAATACCAATCGGGAAGATCTCTTGATACTGTGGGGGATTCATCATTCCAACAATGCA	*600*
**H5 246038 Method A**	*541*	AATAATACCAATCGGGAAGATCTCTTGATACTGTGGGGGATTCATCATTCCAACAATGCA	*600*
**H5 246038 Method S**	*541*	AATAATACCAATCGGGAAGATCTCTTGATACTGTGGGGGATTCATCATTCCAACAATGCA	*600*
**H5 246038 Method E**	*541*	AATAATACCAATCGGGAAGATCTCTTGATACTGTGGGGGATTCATCATTCCAACAATGCA	*600*
**H5 246038 Method K**	*541*	AATAATACCAATCGGGAAGATCTCTTGATACTGTGGGGGATTCATCATTCCAACAATGCA	*600*
**H5 246038 Method N**	*541*	AATAATACCAATCGGGAAGATCTCTTGATACTGTGGGGGATTCATCATTCCAACAATGCA	*600*

**H5 246038 MiSeq**	*601*	GAAGAGCAGACAAATCTCTACAAAAACCCAACCACCTACATTTCAGTTGGAACATCAACT	*660*
**H5 246038 Method A**	*601*	GAAGAGCAGACAAATCTCTACAAAAACCCAACCACCTACATTTCAGTTGGAACATCAACT	*660*
**H5 246038 Method S**	*601*	GAAGAGCAGACAAATCTCTACAAAAACCCAACCACCTACATTTCAGTTGGAACATCAACT	*660*
**H5 246038 Method E**	*601*	GAAGAGCAGACAAATCTCTACAAAAACCCAACCACCTACATTTCAGTTGGAACATCAACT	*660*
**H5 246038 Method K**	*601*	GAAGAGCAGACAAATCTCTACAAAAACCCAACCACCTACATTTCAGTTGGAACATCAACT	*660*
**H5 246038 Method N**	*601*	GAAGAGCAGACAAATCTCTACAAAAACCCAACCACCTACATTTCAGTTGGAACATCAACT	*660*

**H5 246038 MiSeq**	*661*	TTAAACCAGAGGTTGGCACCAAAAATAGCTACTAGATCCCAAGTAAACGGGCAACGTGGA	*720*
**H5 246038 Method A**	*661*	TTAAACCAGAGGTTGGCACCAAAAATAGCTACTAGATCCCAAGTAAACGGGCAACGTGGA	*720*
**H5 246038 Method S**	*661*	TTAAACCAGAGGTTGGCACCAAAAATAGCTACTAGATCCCAAGTAAACGGGCAACGTGGA	*720*
**H5 246038 Method E**	*661*	TTAAACCAGAGGTTGGCACCAAAAATAGCTACTAGATCCCAAGTAAACGGGCAACGTGGA	*720*
**H5 246038 Method K**	*661*	TTAAACCAGAGGTTGGCACCAAAAATAGCTACTAGATCCCAAGTAAACGGGCAACGTGGA	*720*
**H5 246038 Method N**	*661*	TTAAACCAGAGGTTGGCACCAAAAATAGCTACTAGATCCCAAGTAAACGGGCAACGTGGA	*720*

**H5 246038 MiSeq**	*721*	AGAATGGACTTCTTCTGGACAATCTTAAAACCAGATGATGCAATCCATTTCGAGAGTAAT	*780*
**H5 246038 Method A**	*721*	AGAATGGACTTCTTCTGGACAATCTTAAAACCAGATGATGCAATCCATTTCGAGAGTAAT	*780*
**H5 246038 Method S**	*721*	AGAATGGACTTCTTCTGGACAATCTTAAAACCAGATGATGCAATCCATTTCGAGAGTAAT	*780*
**H5 246038 Method E**	*721*	AGAATGGACTTCTTCTGGACAATCTTAAAACCAGATGATGCAATCCATTTCGAGAGTAAT	*780*
**H5 246038 Method K**	*721*	AGAATGGACTTCTTCTGGACAATCTTAAAACCAGATGATGCAATCCATTTCGAGAGTAAT	*780*
**H5 246038 Method N**	*721*	AGAATGGACTTCTTCTGGACAATCTTAAAACCAGATGATGCAATCCATTTCGAGAGTAAT	*780*

**H5 246038 MiSeq**	*781*	GGAAATTTCATTGCTCCAGAATATGCATACAAAATTGTCAAGAAAGGGGACTCAACAATT	*840*
**H5 246038 Method A**	*781*	GGAAATTTCATTGCTCCAGAATATGCATACAAAATTGTCAAGAAAGGGGACTCAACAATT	*840*
**H5 246038 Method S**	*781*	GGAAATTTCATTGCTCCAGAATATGCATACAAAATTGTCAAGAAAGGGGACTCAACAATT	*840*
**H5 246038 Method E**	*781*	GGAAATTTCATTGCTCCAGAATATGCATACAAAATTGTCAAGAAAGGGGACTCAACAATT	*840*
**H5 246038 Method K**	*781*	GGAAATTTCATTGCTCCAGAATATGCATACAAAATTGTCAAGAAAGGGGACTCAACAATT	*840*
**H5 246038 Method N**	*781*	GGAAATTTCATTGCTCCAGAATATGCATACAAAATTGTCAAGAAAGGGGACTCAACAATT	*840*

**H5 246038 MiSeq**	*841*	ATGAAAAGTGGAGTGGAATATGGCCACTGCAACACCAAATGTCAAACCCCAGTAGGTGCG	*900*
**H5 246038 Method A**	*841*	ATGAAAAGTGGAGTGGAATATGGCCACTGCAACACCAAATGTCAAACCCCAGTAGGTGCG	*900*
**H5 246038 Method S**	*841*	ATGAAAAGTGGAGTGGAATATGGCCACTGCAACACCAAATGTCAAACCCCAGTAGGTGCG	*900*
**H5 246038 Method E**	*841*	ATGAAAAGTGGAGTGGAATATGGCCACTGCAACACCAAATGTCAAACCCCAGTAGGTGCG	*900*
**H5 246038 Method K**	*841*	ATGAAAAGTGGAGTGGAATATGGCCACTGCAACACCAAATGTCAAACCCCAGTAGGTGCG	*900*
**H5 246038 Method N**	*841*	ATGAAAAGTGGAGTGGAATATGGCCACTGCAACACCAAATGTCAAACCCCAGTAGGTGCG	*900*

**H5 246038 MiSeq**	*901*	ATAAATTCTAGTATGCCATTCCACAACATACATCCTCTCACCATTGGGGAATGCCCCAAA	*960*
**H5 246038 Method A**	*901*	ATAAATTCTAGTATGCCATTCCACAACATACATCCTCTCACCATTGGGGAATGCCCCAAA	*960*
**H5 246038 Method S**	*901*	ATAAATTCTAGTATGCCATTCCACAACATACATCCTCTCACCATTGGGGAATGCCCCAAA	*960*
**H5 246038 Method E**	*901*	ATAAATTCTAGTATGCCATTCCACAACATACATCCTCTCACCATTGGGGAATGCCCCAAA	*960*
**H5 246038 Method K**	*901*	ATAAATTCTAGTATGCCATTCCACAACATACATCCTCTCACCATTGGGGAATGCCCCAAA	*960*
**H5 246038 Method N**	*901*	ATAAATTCTAGTATGCCATTCCACAACATACATCCTCTCACCATTGGGGAATGCCCCAAA	*960*

**H5 246038 MiSeq**	*961*	TACGTGAAGTCAAACAAGTTGGTCCTTGCGACTGGGCTCAGAAATAGTCCTCTAAGAGAA	*1020*
**H5 246038 Method A**	*961*	TACGTGAAGTCAAACAAGTTGGTCCTTGCGACTGGGCTCAGAAATAGTCCTCTAAGAGAA	*1020*
**H5 246038 Method S**	*961*	TACGTGAAGTCAAACAAGTTGGTCCTTGCGACTGGGCTCAGAAATAGTCCTCTAAGAGAA	*1020*
**H5 246038 Method E**	*961*	TACGTGAAGTCAAACAAGTTGGTCCTTGCGACTGGGCTCAGAAATAGTCCTCTAAGAGAA	*1020*
**H5 246038 Method K**	*961*	TACGTGAAGTCAAACAAGTTGGTCCTTGCGACTGGGCTCAGAAATAGTCCTCTAAGAGAA	*1020*
**H5 246038 Method N**	*961*	TACGTGAAGTCAAACAAGTTGGTCCTTGCGACTGGGCTCAGAAATAGTCCTCTAAGAGAA	*1020*

**H5 246038 MiSeq**	*1021*	AAGAGAAGAAAAAGGGGCCTGTTTGGGGCGATAGCAGGGTTTATAGAGGGAGGATGGCAG	*1080*
**H5 246038 Method A**	*1021*	AAGAGAAGAAAAAGGGGCCTGTTTGGGGCGATAGCAGGGTTTATAGAGGGAGGATGGCAG	*1080*
**H5 246038 Method S**	*1021*	AAGAGAAGAAAAAGGGGCCTGTTTGGGGCGATAGCAGGGTTTATAGAGGGAGGATGGCAG	*1080*
**H5 246038 Method E**	*1021*	AAGAGAAGAAAAAGGGGCCTGTTTGGGGCGATAGCAGGGTTTATAGAGGGAGGATGGCAG	*1080*
**H5 246038 Method K**	*1021*	AAGAGAAGAAAAAGGGGCCTGTTTGGGGCGATAGCAGGGTTTATAGAGGGAGGATGGCAG	*1080*
**H5 246038 Method N**	*1021*	AAGAGAAGAAAAAGGGGCCTGTTTGGGGCGATAGCAGGGTTTATAGAGGGAGGATGGCAG	*1080*

**H5 246038 MiSeq**	*1081*	GGAATGGTTGATGGTTGGTATGGGTACCATCATAGCAATGAGCAGGGGAGTGGATACGCT	*1140*
**H5 246038 Method A**	*1081*	GGAATGGTTGATGGTTGGTATGGGTACCATCATAGCAATGAGCAGGGGAGTGGATACGCT	*1140*
**H5 246038 Method S**	*1081*	GGAATGGTTGATGGTTGGTATGGGTACCATCATAGCAATGAGCAGGGGAGTGGATACGCT	*1140*
**H5 246038 Method E**	*1081*	GGAATGGTTGATGGTTGGTATGGGTACCATCATAGCAATGAGCAGGGGAGTGGATACGCT	*1140*
**H5 246038 Method K**	*1081*	GGAATGGTTGATGGTTGGTATGGGTACCATCATAGCAATGAGCAGGGGAGTGGATACGCT	*1140*
**H5 246038 Method N**	*1081*	GGAATGGTTGATGGTTGGTATGGGTACCATCATAGCAATGAGCAGGGGAGTGGATACGCT	*1140*

**H5 246038 MiSeq**	*1141*	GCGGACAAAGAATCCACCCAAAAGGCAATAGATGGAGTTACCAATAAGGTCAACTCAATC	*1200*
**H5 246038 Method A**	*1141*	GCGGACAAAGAATCCACCCAAAAGGCAATAGATGGAGTTACCAATAAGGTCAACTCAATC	*1200*
**H5 246038 Method S**	*1141*	GCGGACAAAGAATCCACCCAAAAGGCAATAGATGGAGTTACCAATAAGGTCAACTCAATC	*1200*
**H5 246038 Method E**	*1141*	GCGGACAAAGAATCCACCCAAAAGGCAATAGATGGAGTTACCAATAAGGTCAACTCAATC	*1200*
**H5 246038 Method K**	*1141*	GCGGACAAAGAATCCACCCAAAAGGCAATAGATGGAGTTACCAATAAGGTCAACTCAATC	*1200*
**H5 246038 Method N**	*1141*	GCGGACAAAGAATCCACCCAAAAGGCAATAGATGGAGTTACCAATAAGGTCAACTCAATC	*1200*

**H5 246038 MiSeq**	*1201*	ATTGACAAAATGAACACTCAATTTGAGGCAGTTGGAAGGGAGTTTAATAACTTAGAAAGG	*1260*
**H5 246038 Method A**	*1201*	ATTGACAAAATGAACACTCAATTTGAGGCAGTTGGAAGGGAGTTTAATAACTTAGAAAGG	*1260*
**H5 246038 Method S**	*1201*	ATTGACAAAATGAACACTCAATTTGAGGCAGTTGGAAGGGAGTTTAATAACTTAGAAAGG	*1260*
**H5 246038 Method E**	*1201*	ATTGACAAAATGAACACTCAATTTGAGGCAGTTGGAAGGGAGTTTAATAACTTAGAAAGG	*1260*
**H5 246038 Method K**	*1201*	ATTGACAAAATGAACACTCAATTTGAGGCAGTTGGAAGGGAGTTTAATAACTTAGAAAGG	*1260*
**H5 246038 Method N**	*1201*	ATTGACAAAATGAACACTCAATTTGAGGCAGTTGGAAGGGAGTTTAATAACTTAGAAAGG	*1260*

**H5 246038 MiSeq**	*1261*	AGGATAGAGAATTTGAACAAGAAAATGGAAGACGGATTCCTAGATGTCTGGACCTATAAT	*1320*
**H5 246038 Method A**	*1261*	AGGATAGAGAATTTGAACAAGAAAATGGAAGACGGATTCCTAGATGTCTGGACCTATAAT	*1320*
**H5 246038 Method S**	*1261*	AGGATAGAGAATTTGAACAAGAAAATGGAAGACGGATTCCTAGATGTCTGGACCTATAAT	*1320*
**H5 246038 Method E**	*1261*	AGGATAGAGAATTTGAACAAGAAAATGGAAGACGGATTCCTAGATGTCTGGACCTATAAT	*1320*
**H5 246038 Method K**	*1261*	AGGATAGAGAATTTGAACAAGAAAATGGAAGACGGATTCCTAGATGTCTGGACCTATAAT	*1320*
**H5 246038 Method N**	*1261*	AGGATAGAGAATTTGAACAAGAAAATGGAAGACGGATTCCTAGATGTCTGGACCTATAAT	*1320*

**H5 246038 MiSeq**	*1321*	GCTGAACTTCTAGTTCTCATGGAAAACGAGAGGACTCTAGATTTCCATGATTCAAATGTC	*1380*
**H5 246038 Method A**	*1321*	GCTGAACTTCTAGTTCTCATGGAAAACGAGAGGACTCTAGATTTCCATGATTCAAATGTC	*1380*
**H5 246038 Method S**	*1321*	GCTGAACTTCTAGTTCTCATGGAAAACGAGAGGACTCTAGATTTCCATGATTCAAATGTC	*1380*
**H5 246038 Method E**	*1321*	GCTGAACTTCTAGTTCTCATGGAAAACGAGAGGACTCTAGATTTCCATGATTCAAATGTC	*1380*
**H5 246038 Method K**	*1321*	GCTGAACTTCTAGTTCTCATGGAAAACGAGAGGACTCTAGATTTCCATGATTCAAATGTC	*1380*
**H5 246038 Method N**	*1321*	GCTGAACTTCTAGTTCTCATGGAAAACGAGAGGACTCTAGATTTCCATGATTCAAATGTC	*1380*

**H5 246038 MiSeq**	*1381*	AAGAACCTTTACGACAAAGTCAGATTACAGCTTAGGGATAATGCAAAGGAGCTGGGTAAC	*1440*
**H5 246038 Method A**	*1381*	AAGAACCTTTACGACAAAGTCAGATTACAGCTTAGGGATAATGCAAAGGAGCTGGGTAAC	*1440*
**H5 246038 Method S**	*1381*	AAGAACCTTTACGACAAAGTCAGATTACAGCTTAGGGATAATGCAAAGGAGCTGGGTAAC	*1440*
**H5 246038 Method E**	*1381*	AAGAACCTTTACGACAAAGTCAGATTACAGCTTAGGGATAATGCAAAGGAGCTGGGTAAC	*1440*
**H5 246038 Method K**	*1381*	AAGAACCTTTACGACAAAGTCAGATTACAGCTTAGGGATAATGCAAAGGAGCTGGGTAAC	*1440*
**H5 246038 Method N**	*1381*	AAGAACCTTTACGACAAAGTCAGATTACAGCTTAGGGATAATGCAAAGGAGCTGGGTAAC	*1440*

H5 246038 MiSeq	1441	GGCTGTTTCGAATTCTATCACAAATGTGATAATGAATGTATGGAAAGTGTGAAAAATGGG	1500
H5 246038 Method A	1441	GGCTGTTTCGAATTCTATCACAAATGTGATAATGAATGTATGGAAAGTGTGAAAAATGGG	1500
H5 246038 Method S	1441	GGCTGTTTCGAATTCTATCACAAATGTGATAATGAATGTATGGAAAGTGTGAAAAATGGG	1500
H5 246038 Method E	1441	GGCTGTTTCGAATTCTATCACAAATGTGATAATGAATGTATGGAAAGTGTGAAAAATGGG	1500
H5 246038 Method K	1441	GGCTGTTTCGAATTCTATCACAAATGTGATAATGAATGTATGGAAAGTGTGAAAAATGGG	1500
H5 246038 Method N	1441	GGCTGTTTCGAATTCTATCACAAATGTGATAATGAATGTATGGAAAGTGTGAAAAATGGG	1500

H5 246038 MiSeq	1501	ACGTATGACTACCCTCAGTATTCAGAAGAAGCAAGATTAAAAAGAGAAGAAATAAGCGGA	1560
H5 246038 Method A	1501	ACGTATGACTACCCTCAGTATTCAGAAGAAGCAAGATTAAAAAGAGAAGAAATAAGCGGA	1560
H5 246038 Method S	1501	ACGTATGACTACCCTCAGTATTCAGAAGAAGCAAGATTAAAAAGAGAAGAAATAAGCGGA	1560
H5 246038 Method E	1501	ACGTATGACTACCCTCAGTATTCAGAAGAAGCAAGATTAAAAAGAGAAGAAATAAGCGGA	1560
H5 246038 Method K	1501	ACGTATGACTACCCTCAGTATTCAGAAGAAGCAAGATTAAAAAGAGAAGAAATAAGCGGA	1560
H5 246038 Method N	1501	ACGTATGACTACCCTCAGTATTCAGAAGAAGCAAGATTAAAAAGAGAAGAAATAAGCGGA	1560

H5 246038 MiSeq	1561	GTGAAATTAGAATCAGTAGGAACTTACCAGATACTGTCAATTTATTCAACAGCGGCAAGT	1620
H5 246038 Method A	1561	GTGAAATTAGAATCAGTAGGAACTTACCAGATACTGTCAATTTATTCAACAGCGGCAAGT	1620
H5 246038 Method S	1561	GTGAAATTAGAATCAGTAGGAACTTACCAGATACTGTCAATTTATTCAACAGCGGCAAGT	1620
H5 246038 Method E	1561	GTGAAATTAGAATCAGTAGGAACTTACCAGATACTGTCAATTTATTCAACAGCGGCAAGT	1620
H5 246038 Method K	1561	GTGAAATTAGAATCAGTAGGAACTTACCAGATACTGTCAATTTATTCAACAGCGGCAAGT	1620
H5 246038 Method N	1561	GTGAAATTAGAATCAGTAGGAACTTACCAGATACTGTCAATTTATTCAACAGCGGCAAGT	1620

H5 246038 MiSeq	1621	TCCCTAGCACTGGCAATCATGATGGCTGGTCTATCTTTATGGATGTGCTCCAATGGGTCG	1680
H5 246038 Method A	1621	TCCCTAGCACTGGCAATCATGATGGCTGGTCTATCTTTATGGATGTGCTCCAATGGGTCG	1680
H5 246038 Method S	1621	TCCCTAGCACTGGCAATCATGATGGCTGGTCTATCTTTATGGATGTGCTCCAATGGGTCG	1680
H5 246038 Method E	1621	TCCCTAGCACTGGCAATCATGATGGCTGGTCTATCTTTATGGATGTGCTCCAATGGGTCG	1680
H5 246038 Method K	1621	TCCCTAGCACTGGCAATCATGATGGCTGGTCTATCTTTATGGATGTGCTCCAATGGGTCG	1680
H5 246038 Method N	1621	TCCCTAGCACTGGCAATCATGATGGCTGGTCTATCTTTATGGATGTGCTCCAATGGGTCG	1680

**H5 246038 MiSeq**	*1681*	T T A C A G T G C A G A A T T T G C A T T T A G	*1704*
**H5 246038 Method A**	*1681*	T T A C A G T G C A G A A T T T G C A T T T A G	*1704*
**H5 246038 Method S**	*1681*	T T A C A G T G C A G A A T T T G C A T T T A G	*1704*
**H5 246038 Method E**	*1681*	T T A C A G T G C A G A A T T T G C A T T T A G	*1704*
**H5 246038 Method K**	*1681*	T T A C A G T G C A G A A T T T G C A T T T A G	*1704*
**H5 246038 Method N**	*1681*	T T A C A G T G C A G A A T T T G C A T T T A G	*1704*
