# Supplementary material for: An Evaluation of Avian Influenza Virus Whole-Genome Sequencing Approaches Using Nanopore Technology
Source: Microorganisms. 2023 Feb 19;11(2):529. doi: 10.3390/microorganisms11020529 (PMC9967579; doi:10.3390/microorganisms11020529)
Supplement: Supplementary file 1 [file microorganisms-11-00529-s001.zip › manuscript.v8 230219 Suppl Figures and Tables/Supplementary Figures S3a-h 246038/Supplementary Figure S3b PB1.pdf]

Formatted Alignments

PB1 246038 MiSeq	1	ATGGATGTCAATCCGACTTTACTTTTCTTAAAAGTGCCAGCGCAAAATGCCATAAGTACC	60
PB1 246038 Method A	1	ATGGATGTCAATCCGACTTTACTTTTCTTAAAAGTGCCAGCGCAAAATGCCATAAGTACC	60
PB1 246038 Method S	1	ATGGATGTCAATCCGACTTTACTTTTCTTAAAAGTGCCAGCGCAAAATGCCATAAGTACC	60
PB1 246038 Method E	1	ATGGATGTCAATCCGACTTTACTTTTCTTAAAAGTGCCAGCGCAAAATGCCATAAGTACC	60
PB1 246038 Method K	1	ATGGATGTCAATCCGACTTTACTTTTCTTAAAAGTGCCAGCGCAAAATGCCATAAGTACC	60
PB1 246038 Method N	1	ATGGATGTCAATCCGACTTTACTTTTCTTAAAAGTGCCAGCGCAAAATGCCATAAGTACC	60

PB1 246038 MiSeq	61	ACATTCCCTTATACTGGAGATCCTCCATACAGCCATGGAACAGGGACAGGATACACAATG	120
PB1 246038 Method A	61	ACATTCCCTTATACTGGAGATCCTCCATACAGCCATGGAACAGGGACAGGATACACAATG	120
PB1 246038 Method S	61	ACATTCCCTTATACTGGAGATCCTCCATACAGCCATGGAACAGGGACAGGATACACAATG	120
PB1 246038 Method E	61	ACATTCCCTTATACTGGAGATCCTCCATACAGCCATGGAACAGGGACAGGATACACAATG	120
PB1 246038 Method K	61	ACATTCCCTTATACTGGAGATCCTCCATACAGCCATGGAACAGGGACAGGATACACAATG	120
PB1 246038 Method N	61	ACATTCCCTTATACTGGAGATCCTCCATACAGCCATGGAACAGGGACAGGATACACAATG	120

PB1 246038 MiSeq	121	GACACAGTCAACAGAACACATCAATACTCAGAGAAGGGAAAATGGACAACAAACACAGAA	180
PB1 246038 Method A	121	GACACAGTCAACAGAACACATCAATACTCAGAGAAGGGAAAATGGACAACAAACACAGAA	180
PB1 246038 Method S	121	GACACAGTCAACAGAACACATCAATACTCAGAGAAGGGAAAATGGACAACAAACACAGAA	180
PB1 246038 Method E	121	GACACAGTCAACAGAACACATCAATACTCAGAGAAGGGAAAATGGACAACAAACACAGAA	180
PB1 246038 Method K	121	GACACAGTCAACAGAACACATCAATACTCAGAGAAGGGAAAATGGACAACAAACACAGAA	180
PB1 246038 Method N	121	GACACAGTCAACAGAACACATCAATACTCAGAGAAGGGAAAATGGACAACAAACACAGAA	180

PB1 246038 MiSeq	181	ACCGGAGCACCTCAACTCAACCCAATTGATGGACCACTACCTGGGGACAACGAACCGAGC	240
PB1 246038 Method A	181	ACCGGAGCACCTCAACTCAACCCAATTGATGGACCACTACCTGGGGACAACGAACCGAGC	240
PB1 246038 Method S	181	ACCGGAGCACCTCAACTCAACCCAATTGATGGACCACTACCTGGGGACAACGAACCGAGC	240
PB1 246038 Method E	181	ACCGGAGCACCTCAACTCAACCCAATTGATGGACCACTACCTGGGGACAACGAACCGAGC	240
PB1 246038 Method K	181	ACCGGAGCACCTCAACTCAACCCAATTGATGGACCACTACCTGGGGACAACGAACCGAGC	240
PB1 246038 Method N	181	ACCGGAGCACCTCAACTCAACCCAATTGATGGACCACTACCTGGGGACAACGAACCGAGC	240

**PB1 246038 MiSeq**	*241*	GGATATGCACAAACAGATTGCGTGTTGGAAGCAATGGCTTTCCTTGAAGAGTCCCACCCA	*300*
**PB1 246038 Method A**	*241*	GGATATGCACAAACAGATTGCGTGTTGGAAGCAATGGCTTTCCTTGAAGAGTCCCACCCA	*300*
**PB1 246038 Method S**	*241*	GGATATGCACAAACAGATTGCGTGTTGGAAGCAATGGCTTTCCTTGAAGAGTCCCACCCA	*300*
**PB1 246038 Method E**	*241*	GGATATGCACAAACAGATTGCGTGTTGGAAGCAATGGCTTTCCTTGAAGAGTCCCACCCA	*300*
**PB1 246038 Method K**	*241*	GGATATGCACAAACAGATTGCGTGTTGGAAGCAATGGCTTTCCTTGAAGAGTCCCACCCA	*300*
**PB1 246038 Method N**	*241*	GGATATGCACAAACAGATTGCGTGTTGGAAGCAATGGCTTTCCTTGAAGAGTCCCACCCA	*300*

**PB1 246038 MiSeq**	*301*	GGGATCTTTGGAAACTCTTGTCTTGAAACGATGGAAGTCGTTCAGCAAACAAGAGTGGAC	*360*
**PB1 246038 Method A**	*301*	GGGATCTTTGGAAACTCTTGTCTTGAAACGATGGAAGTCGTTCAGCAAACAAGAGTGGAC	*360*
**PB1 246038 Method S**	*301*	GGGATCTTTGGAAACTCTTGTCTTGAAACGATGGAAGTCGTTCAGCAAACAAGAGTGGAC	*360*
**PB1 246038 Method E**	*301*	GGGATCTTTGGAAACTCTTGTCTTGAAACGATGGAAGTCGTTCAGCAAACAAGAGTGGAC	*360*
**PB1 246038 Method K**	*301*	GGGATCTTTGGAAACTCTTGTCTTGAAACGATGGAAGTCGTTCAGCAAACAAGAGTGGAC	*360*
**PB1 246038 Method N**	*301*	GGGATCTTTGGAAACTCTTGTCTTGAAACGATGGAAGTCGTTCAGCAAACAAGAGTGGAC	*360*

**PB1 246038 MiSeq**	*361*	AAACTAACTCAAGGTCGCCAGACTTATGACTGGACACTGAATAGAAACCAACCAGCTGCA	*420*
**PB1 246038 Method A**	*361*	AAACTAACTCAAGGTCGCCAGACTTATGACTGGACACTGAATAGAAACCAACCAGCTGCA	*420*
**PB1 246038 Method S**	*361*	AAACTAACTCAAGGTCGCCAGACTTATGACTGGACACTGAATAGAAACCAACCAGCTGCA	*420*
**PB1 246038 Method E**	*361*	AAACTAACTCAAGGTCGCCAGACTTATGACTGGACACTGAATAGAAACCAACCAGCTGCA	*420*
**PB1 246038 Method K**	*361*	AAACTAACTCAAGGTCGCCAGACTTATGACTGGACACTGAATAGAAACCAACCAGCTGCA	*420*
**PB1 246038 Method N**	*361*	AAACTAACTCAAGGTCGCCAGACTTATGACTGGACACTGAATAGAAACCAACCAGCTGCA	*420*

**PB1 246038 MiSeq**	*421*	ACTGCCCTGGCCAACACTATAGAGGTCTTCAGATCAAACGGTCTAACAGCCAATGAATCG	*480*
**PB1 246038 Method A**	*421*	ACTGCCCTGGCCAACACTATAGAGGTCTTCAGATCAAACGGTCTAACAGCCAATGAATCG	*480*
**PB1 246038 Method S**	*421*	ACTGCCCTGGCCAACACTATAGAGGTCTTCAGATCAAACGGTCTAACAGCCAATGAATCG	*480*
**PB1 246038 Method E**	*421*	ACTGCCCTGGCCAACACTATAGAGGTCTTCAGATCAAACGGTCTAACAGCCAATGAATCG	*480*
**PB1 246038 Method K**	*421*	ACTGCCCTGGCCAACACTATAGAGGTCTTCAGATCAAACGGTCTAACAGCCAATGAATCG	*480*
**PB1 246038 Method N**	*421*	ACTGCCCTGGCCAACACTATAGAGGTCTTCAGATCAAACGGTCTAACAGCCAATGAATCG	*480*

**PB1 246038 MiSeq**	*481*	GGGAGACTAATAGATTTCCTCAAGGACGTGATGGACTCAATGGATACAGAAGAAATGGAA	*540*
**PB1 246038 Method A**	*481*	GGGAGACTAATAGATTTCCTCAAGGACGTGATGGACTCAATGGATACAGAAGAAATGGAA	*540*
**PB1 246038 Method S**	*481*	GGGAGACTAATAGATTTCCTCAAGGACGTGATGGACTCAATGGATACAGAAGAAATGGAA	*540*
**PB1 246038 Method E**	*481*	GGGAGACTAATAGATTTCCTCAAGGACGTGATGGACTCAATGGATACAGAAGAAATGGAA	*540*
**PB1 246038 Method K**	*481*	GGGAGACTAATAGATTTCCTCAAGGACGTGATGGACTCAATGGATACAGAAGAAATGGAA	*540*
**PB1 246038 Method N**	*481*	GGGAGACTAATAGATTTCCTCAAGGACGTGATGGACTCAATGGATACAGAAGAAATGGAA	*540*

```
PB1 246038 MiSeq      541  ATAACAACACATTTCCAGAGAAAGAGAAGAGTAAGGGACAACATGACCAAGAAAATGGTC  600
PB1 246038 Method A   541  ATAACAACACATTTCCAGAGAAAGAGAAGAGTAAGGGACAACATGACCAAGAAAATGGTC  600
PB1 246038 Method S   541  ATAACAACACATTTCCAGAGAAAGAGAAGAGTAAGGGACAACATGACCAAGAAAATGGTC  600
PB1 246038 Method E   541  ATAACAACACATTTCCAGAGAAAGAGAAGAGTAAGGGACAACATGACCAAGAAAATGGTC  600
PB1 246038 Method K   541  ATAACAACACATTTCCAGAGAAAGAGAAGAGTAAGGGACAACATGACCAAGAAAATGGTC  600
PB1 246038 Method N   541  ATAACAACACATTTCCAGAGAAAGAGAAGAGTAAGGGACAACATGACCAAGAAAATGGTC  600

PB1 246038 MiSeq      601  ACACAAAGAACAATAGGAAAGAAGAAACAAAGGCTAAACAAGAGGAGCTACTTAATAAGA  660
PB1 246038 Method A   601  ACACAAAGAACAATAGGAAAGAAGAAACAAAGGCTAAACAAGAGGAGCTACTTAATAAGA  660
PB1 246038 Method S   601  ACACAAAGAACAATAGGAAAGAAGAAACAAAGGCTAAACAAGAGGAGCTACTTAATAAGA  660
PB1 246038 Method E   601  ACACAAAGAACAATAGGAAAGAAGAAACAAAGGCTAAACAAGAGGAGCTACTTAATAAGA  660
PB1 246038 Method K   601  ACACAAAGAACAATAGGAAAGAAGAAACAAAGGCTAAACAAGAGGAGCTACTTAATAAGA  660
PB1 246038 Method N   601  ACACAAAGAACAATAGGAAAGAAGAAACAAAGGCTAAACAAGAGGAGCTACTTAATAAGA  660

PB1 246038 MiSeq      661  GCACTGACACTGAATACAATGACAAAAGATGCAGAAAGAGGCAAATTGAAGAGACGGGCG  720
PB1 246038 Method A   661  GCACTGACACTGAATACAATGACAAAAGATGCAGAAAGAGGCAAATTGAAGAGACGGGCG  720
PB1 246038 Method S   661  GCACTGACACTGAATACAATGACAAAAGATGCAGAAAGAGGCAAATTGAAGAGACGGGCG  720
PB1 246038 Method E   661  GCACTGACACTGAATACAATGACAAAAGATGCAGAAAGAGGCAAATTGAAGAGACGGGCG  720
PB1 246038 Method K   661  GCACTGACACTGAATACAATGACAAAAGATGCAGAAAGAGGCAAATTGAAGAGACGGGCG  720
PB1 246038 Method N   661  GCACTGACACTGAATACAATGACAAAAGATGCAGAAAGAGGCAAATTGAAGAGACGGGCG  720

PB1 246038 MiSeq      721  ATTGCAACACCAGGGATGCAGATTAGAGGATTTGTGTACTTTGTCGAAACACTGGCAAGG  780
PB1 246038 Method A   721  ATTGCAACACCAGGGATGCAGATTAGAGGATTTGTGTACTTTGTCGAAACACTGGCAAGG  780
PB1 246038 Method S   721  ATTGCAACACCAGGGATGCAGATTAGAGGATTTGTGTACTTTGTCGAAACACTGGCAAGG  780
PB1 246038 Method E   721  ATTGCAACACCAGGGATGCAGATTAGAGGATTTGTGTACTTTGTCGAAACACTGGCAAGG  780
PB1 246038 Method K   721  ATTGCAACACCAGGGATGCAGATTAGAGGATTTGTGTACTTTGTCGAAACACTGGCAAGG  780
PB1 246038 Method N   721  ATTGCAACACCAGGGATGCAGATTAGAGGATTTGTGTACTTTGTCGAAACACTGGCAAGG  780

PB1 246038 MiSeq      781  AGCATCTGTGAAAAACTTGAGCAATCTGGACTCCCCGTTGGAGGAAATGAGAAGAAGGCT  840
PB1 246038 Method A   781  AGCATCTGTGAAAAACTTGAGCAATCTGGACTCCCCGTTGGAGGAAATGAGAAGAAGGCT  840
PB1 246038 Method S   781  AGCATCTGTGAAAAACTTGAGCAATCTGGACTCCCCGTTGGAGGAAATGAGAAGAAGGCT  840
PB1 246038 Method E   781  AGCATCTGTGAAAAACTTGAGCAATCTGGACTCCCCGTTGGAGGAAATGAGAAGAAGGCT  840
PB1 246038 Method K   781  AGCATCTGTGAAAAACTTGAGCAATCTGGACTCCCCGTTGGAGGAAATGAGAAGAAGGCT  840
PB1 246038 Method N   781  AGCATCTGTGAAAAACTTGAGCAATCTGGACTCCCCGTTGGAGGAAATGAGAAGAAGGCT  840
```

PB1 246038 MiSeq	841	AAATTGGCAAATGTCGTGAGGAAAATGATGACTAACTCACAAGATACAGAGCTCTCCTTC	900
PB1 246038 Method A	841	AAATTGGCAAATGTCGTGAGGAAAATGATGACTAACTCACAAGATACAGAGCTCTCCTTC	900
PB1 246038 Method S	841	AAATTGGCAAATGTCGTGAGGAAAATGATGACTAACTCACAAGATACAGAGCTCTCCTTC	900
PB1 246038 Method E	841	AAATTGGCAAATGTCGTGAGGAAAATGATGACTAACTCACAAGATACAGAGCTCTCCTTC	900
PB1 246038 Method K	841	AAATTGGCAAATGTCGTGAGGAAAATGATGACTAACTCACAAGATACAGAGCTCTCCTTC	900
PB1 246038 Method N	841	AAATTGGCAAATGTCGTGAGGAAAATGATGACTAACTCACAAGATACAGAGCTCTCCTTC	900
PB1 246038 MiSeq	901	ACAATTACTGGAGATAACACAAAATGGAATGAGAATCAAAATCCTCGGATGTTTCTGGCA	960
PB1 246038 Method A	901	ACAATTACTGGAGATAACACAAAATGGAATGAGAATCAAAATCCTCGGATGTTTCTGGCA	960
PB1 246038 Method S	901	ACAATTACTGGAGATAACACAAAATGGAATGAGAATCAAAATCCTCGGATGTTTCTGGCA	960
PB1 246038 Method E	901	ACAATTACTGGAGATAACACAAAATGGAATGAGAATCAAAATCCTCGGATGTTTCTGGCA	960
PB1 246038 Method K	901	ACAATTACTGGAGATAACACAAAATGGAATGAGAATCAAAATCCTCGGATGTTTCTGGCA	960
PB1 246038 Method N	901	ACAATTACTGGAGATAACACAAAATGGAATGAGAATCAAAATCCTCGGATGTTTCTGGCA	960
PB1 246038 MiSeq	961	ATGATAACGTACATTACAAGAAACCAACCTGAATGGTTTAGAAATGTCTTGAGTATTGCC	1020
PB1 246038 Method A	961	ATGATAACGTACATTACAAGAAACCAACCTGAATGGTTTAGAAATGTCTTGAGTATTGCC	1020
PB1 246038 Method S	961	ATGATAACGTACATTACAAGAAACCAACCTGAATGGTTTAGAAATGTCTTGAGTATTGCC	1020
PB1 246038 Method E	961	ATGATAACGTACATTACAAGAAACCAACCTGAATGGTTTAGAAATGTCTTGAGTATTGCC	1020
PB1 246038 Method K	961	ATGATAACGTACATTACAAGAAACCAACCTGAATGGTTTAGAAATGTCTTGAGTATTGCC	1020
PB1 246038 Method N	961	ATGATAACGTACATTACAAGAAACCAACCTGAATGGTTTAGAAATGTCTTGAGTATTGCC	1020
PB1 246038 MiSeq	1021	CCTATAATGTTCTCGAACAAAATGGCGAGATTGGGAAAAGGGTACATGTTTGAAAGTAAG	1080
PB1 246038 Method A	1021	CCTATAATGTTCTCGAACAAAATGGCGAGATTGGGAAAAGGGTACATGTTTGAAAGTAAG	1080
PB1 246038 Method S	1021	CCTATAATGTTCTCGAACAAAATGGCGAGATTGGGAAAAGGGTACATGTTTGAAAGTAAG	1080
PB1 246038 Method E	1021	CCTATAATGTTCTCGAACAAAATGGCGAGATTGGGAAAAGGGTACATGTTTGAAAGTAAG	1080
PB1 246038 Method K	1021	CCTATAATGTTCTCGAACAAAATGGCGAGATTGGGAAAAGGGTACATGTTTGAAAGTAAG	1080
PB1 246038 Method N	1021	CCTATAATGTTCTCGAACAAAATGGCGAGATTGGGAAAAGGGTACATGTTTGAAAGTAAG	1080
PB1 246038 MiSeq	1081	AGCATGAAGTTACGGACACAAATACCTGCAGAAATACTTGCAAACATTGACTTAAAATAC	1140
PB1 246038 Method A	1081	AGCATGAAGTTACGGACACAAATACCTGCAGAAATACTTGCAAACATTGACTTAAAATAC	1140
PB1 246038 Method S	1081	AGCATGAAGTTACGGACACAAATACCTGCAGAAATACTTGCAAACATTGACTTAAAATAC	1140
PB1 246038 Method E	1081	AGCATGAAGTTACGGACACAAATACCTGCAGAAATACTTGCAAACATTGACTTAAAATAC	1140
PB1 246038 Method K	1081	AGCATGAAGTTACGGACACAAATACCTGCAGAAATACTTGCAAACATTGACTTAAAATAC	1140
PB1 246038 Method N	1081	AGCATGAAGTTACGGACACAAATACCTGCAGAAATACTTGCAAACATTGACTTAAAATAC	1140

**PB1 246038 MiSeq**	*1141*	TTCAATGAATCAACAAGAAAGAAAATCGAAAAAATAAGGCCTCTACTAATAGATGGCACT	*1200*
**PB1 246038 Method A**	*1141*	TTCAATGAATCAACAAGAAAGAAAATCGAAAAAATAAGGCCTCTACTAATAGATGGCACT	*1200*
**PB1 246038 Method S**	*1141*	TTCAATGAATCAACAAGAAAGAAAATCGAAAAAATAAGGCCTCTACTAATAGATGGCACT	*1200*
**PB1 246038 Method E**	*1141*	TTCAATGAATCAACAAGAAAGAAAATCGAAAAAATAAGGCCTCTACTAATAGATGGCACT	*1200*
**PB1 246038 Method K**	*1141*	TTCAATGAATCAACAAGAAAGAAAATCGAAAAAATAAGGCCTCTACTAATAGATGGCACT	*1200*
**PB1 246038 Method N**	*1141*	TTCAATGAATCAACAAGAAAGAAAATCGAAAAAATAAGGCCTCTACTAATAGATGGCACT	*1200*

**PB1 246038 MiSeq**	*1201*	GCCTCATTGAGTCCTGGAATGATGATGGGCATGTTCAACATGCTGAGTACAGTATTAGGA	*1260*
**PB1 246038 Method A**	*1201*	GCCTCATTGAGTCCTGGAATGATGATGGGCATGTTCAACATGCTGAGTACAGTATTAGGA	*1260*
**PB1 246038 Method S**	*1201*	GCCTCATTGAGTCCTGGAATGATGATGGGCATGTTCAACATGCTGAGTACAGTATTAGGA	*1260*
**PB1 246038 Method E**	*1201*	GCCTCATTGAGTCCTGGAATGATGATGGGCATGTTCAACATGCTGAGTACAGTATTAGGA	*1260*
**PB1 246038 Method K**	*1201*	GCCTCATTGAGTCCTGGAATGATGATGGGCATGTTCAACATGCTGAGTACAGTATTAGGA	*1260*
**PB1 246038 Method N**	*1201*	GCCTCATTGAGTCCTGGAATGATGATGGGCATGTTCAACATGCTGAGTACAGTATTAGGA	*1260*

**PB1 246038 MiSeq**	*1261*	GTTTCAATCCTAAATCTTGGGCAAAAGAGGTACACCAAAACCACATACTGGTGGGATGGA	*1320*
**PB1 246038 Method A**	*1261*	GTTTCAATCCTAAATCTTGGGCAAAAGAGGTACACCAAAACCACATACTGGTGGGATGGA	*1320*
**PB1 246038 Method S**	*1261*	GTTTCAATCCTAAATCTTGGGCAAAAGAGGTACACCAAAACCACATACTGGTGGGATGGA	*1320*
**PB1 246038 Method E**	*1261*	GTTTCAATCCTAAATCTTGGGCAAAAGAGGTACACCAAAACCACATACTGGTGGGATGGA	*1320*
**PB1 246038 Method K**	*1261*	GTTTCAATCCTAAATCTTGGGCAAAAGAGGTACACCAAAACCACATACTGGTGGGATGGA	*1320*
**PB1 246038 Method N**	*1261*	GTTTCAATCCTAAATCTTGGGCAAAAGAGGTACACCAAAACCACATACTGGTGGGATGGA	*1320*

**PB1 246038 MiSeq**	*1321*	CTCCAATCCTCTGATGATTTCGCCCTCATAGTAAATGCACCTAATCATGAGGGAATACAA	*1380*
**PB1 246038 Method A**	*1321*	CTCCAATCCTCTGATGATTTCGCCCTCATAGTAAATGCACCTAATCATGAGGGAATACAA	*1380*
**PB1 246038 Method S**	*1321*	CTCCAATCCTCTGATGATTTCGCCCTCATAGTAAATGCACCTAATCATGAGGGAATACAA	*1380*
**PB1 246038 Method E**	*1321*	CTCCAATCCTCTGATGATTTCGCCCTCATAGTAAATGCACCTAATCATGAGGGAATACAA	*1380*
**PB1 246038 Method K**	*1321*	CTCCAATCCTCTGATGATTTCGCCCTCATAGTAAATGCACCTAATCATGAGGGAATACAA	*1380*
**PB1 246038 Method N**	*1321*	CTCCAATCCTCTGATGATTTCGCCCTCATAGTAAATGCACCTAATCATGAGGGAATACAA	*1380*

**PB1 246038 MiSeq**	*1381*	GCAGGAGTGGATAGGTTCTATAGGACCTGCAAACTGGTCGGGATCAATATGAGCAAAAAG	*1440*
**PB1 246038 Method A**	*1381*	GCAGGAGTGGATAGGTTCTATAGGACCTGCAAACTGGTCGGGATCAATATGAGCAAAAAG	*1440*
**PB1 246038 Method S**	*1381*	GCAGGAGTGGATAGGTTCTATAGGACCTGCAAACTGGTCGGGATCAATATGAGCAAAAAG	*1440*
**PB1 246038 Method E**	*1381*	GCAGGAGTGGATAGGTTCTATAGGACCTGCAAACTGGTCGGGATCAATATGAGCAAAAAG	*1440*
**PB1 246038 Method K**	*1381*	GCAGGAGTGGATAGGTTCTATAGGACCTGCAAACTGGTCGGGATCAATATGAGCAAAAAG	*1440*
**PB1 246038 Method N**	*1381*	GCAGGAGTGGATAGGTTCTATAGGACCTGCAAACTGGTCGGGATCAATATGAGCAAAAAG	*1440*

**PB1 246038 MiSeq**	*1441*	AAGTCTTACATAAACCGGACTGGAACATTTGAGTTCACAAGCTTTTTCTATCGCTATGGA	*1500*
**PB1 246038 Method A**	*1441*	AAGTCTTACATAAACCGGACTGGAACATTTGAGTTCACAAGCTTTTTCTATCGCTATGGA	*1500*
**PB1 246038 Method S**	*1441*	AAGTCTTACATAAACCGGACTGGAACATTTGAGTTCACAAGCTTTTTCTATCGCTATGGA	*1500*
**PB1 246038 Method E**	*1441*	AAGTCTTACATAAACCGGACTGGAACATTTGAGTTCACAAGCTTTTTCTATCGCTATGGA	*1500*
**PB1 246038 Method K**	*1441*	AAGTCTTACATAAACCGGACTGGAACATTTGAGTTCACAAGCTTTTTCTATCGCTATGGA	*1500*
**PB1 246038 Method N**	*1441*	AAGTCTTACATAAACCGGACTGGAACATTTGAGTTCACAAGCTTTTTCTATCGCTATGGA	*1500*

**PB1 246038 MiSeq**	*1501*	TTTGTGGCTAACTTCAGTATGGAGCTGCCCAGCTTTGGAGTTTCTGGGATCAATGAATCA	*1560*
**PB1 246038 Method A**	*1501*	TTTGTGGCTAACTTCAGTATGGAGCTGCCCAGCTTTGGAGTTTCTGGGATCAATGAATCA	*1560*
**PB1 246038 Method S**	*1501*	TTTGTGGCTAACTTCAGTATGGAGCTGCCCAGCTTTGGAGTTTCTGGGATCAATGAATCA	*1560*
**PB1 246038 Method E**	*1501*	TTTGTGGCTAACTTCAGTATGGAGCTGCCCAGCTTTGGAGTTTCTGGGATCAATGAATCA	*1560*
**PB1 246038 Method K**	*1501*	TTTGTGGCTAACTTCAGTATGGAGCTGCCCAGCTTTGGAGTTTCTGGGATCAATGAATCA	*1560*
**PB1 246038 Method N**	*1501*	TTTGTGGCTAACTTCAGTATGGAGCTGCCCAGCTTTGGAGTTTCTGGGATCAATGAATCA	*1560*

**PB1 246038 MiSeq**	*1561*	GCTGACATGAGCATTGGCGTCACAGTGATAAAGAACAACATGATAAACAATGACCTTGGT	*1620*
**PB1 246038 Method A**	*1561*	GCTGACATGAGCATTGGCGTCACAGTGATAAAGAACAACATGATAAACAATGACCTTGGT	*1620*
**PB1 246038 Method S**	*1561*	GCTGACATGAGCATTGGCGTCACAGTGATAAAGAACAACATGATAAACAATGACCTTGGT	*1620*
**PB1 246038 Method E**	*1561*	GCTGACATGAGCATTGGCGTCACAGTGATAAAGAACAACATGATAAACAATGACCTTGGT	*1620*
**PB1 246038 Method K**	*1561*	GCTGACATGAGCATTGGCGTCACAGTGATAAAGAACAACATGATAAACAATGACCTTGGT	*1620*
**PB1 246038 Method N**	*1561*	GCTGACATGAGCATTGGCGTCACAGTGATAAAGAACAACATGATAAACAATGACCTTGGT	*1620*

**PB1 246038 MiSeq**	*1621*	CCAGCAACAGCTCAAATGGCCCTTCAACTATTCATCAAAGATTACAGGTACACGTACCGA	*1680*
**PB1 246038 Method A**	*1621*	CCAGCAACAGCTCAAATGGCCCTTCAACTATTCATCAAAGATTACAGGTACACGTACCGA	*1680*
**PB1 246038 Method S**	*1621*	CCAGCAACAGCTCAAATGGCCCTTCAACTATTCATCAAAGATTACAGGTACACGTACCGA	*1680*
**PB1 246038 Method E**	*1621*	CCAGCAACAGCTCAAATGGCCCTTCAACTATTCATCAAAGATTACAGGTACACGTACCGA	*1680*
**PB1 246038 Method K**	*1621*	CCAGCAACAGCTCAAATGGCCCTTCAACTATTCATCAAAGATTACAGGTACACGTACCGA	*1680*
**PB1 246038 Method N**	*1621*	CCAGCAACAGCTCAAATGGCCCTTCAACTATTCATCAAAGATTACAGGTACACGTACCGA	*1680*

**PB1 246038 MiSeq**	*1681*	TGCCACAGAGGTGACACACAAATTCAAACGAGGAGATCATTCGAGCTGAAGAAGCTGTGG	*1740*
**PB1 246038 Method A**	*1681*	TGCCACAGAGGTGACACACAAATTCAAACGAGGAGATCATTCGAGCTGAAGAAGCTGTGG	*1740*
**PB1 246038 Method S**	*1681*	TGCCACAGAGGTGACACACAAATTCAAACGAGGAGATCATTCGAGCTGAAGAAGCTGTGG	*1740*
**PB1 246038 Method E**	*1681*	TGCCACAGAGGTGACACACAAATTCAAACGAGGAGATCATTCGAGCTGAAGAAGCTGTGG	*1740*
**PB1 246038 Method K**	*1681*	TGCCACAGAGGTGACACACAAATTCAAACGAGGAGATCATTCGAGCTGAAGAAGCTGTGG	*1740*
**PB1 246038 Method N**	*1681*	TGCCACAGAGGTGACACACAAATTCAAACGAGGAGATCATTCGAGCTGAAGAAGCTGTGG	*1740*

**PB1 246038 MiSeq**	*1741*	GAACAGACCCGTTCAAAGGCAGGACTGTTGGTGTCAGATGGAGGACCAAATCTATACAAC	*1800*
**PB1 246038 Method A**	*1741*	GAACAGACCCGTTCAAAGGCAGGACTGTTGGTGTCAGATGGAGGACCAAATCTATACAAC	*1800*
**PB1 246038 Method S**	*1741*	GAACAGACCCGTTCAAAGGCAGGACTGTTGGTGTCAGATGGAGGACCAAATCTATACAAC	*1800*
**PB1 246038 Method E**	*1741*	GAACAGACCCGTTCAAAGGCAGGACTGTTGGTGTCAGATGGAGGACCAAATCTATACAAC	*1800*
**PB1 246038 Method K**	*1741*	GAACAGACCCGTTCAAAGGCAGGACTGTTGGTGTCAGATGGAGGACCAAATCTATACAAC	*1800*
**PB1 246038 Method N**	*1741*	GAACAGACCCGTTCAAAGGCAGGACTGTTGGTGTCAGATGGAGGACCAAATCTATACAAC	*1800*

**PB1 246038 MiSeq**	*1801*	ATTCGGAATCTCCATATCCCAGAGGTCTGCTTGAAGTGGGAGCTGATGGACGAAGATTAC	*1860*
**PB1 246038 Method A**	*1801*	ATTCGGAATCTCCATATCCCAGAGGTCTGCTTGAAGTGGGAGCTGATGGACGAAGATTAC	*1860*
**PB1 246038 Method S**	*1801*	ATTCGGAATCTCCATATCCCAGAGGTCTGCTTGAAGTGGGAGCTGATGGACGAAGATTAC	*1860*
**PB1 246038 Method E**	*1801*	ATTCGGAATCTCCATATCCCAGAGGTCTGCTTGAAGTGGGAGCTGATGGACGAAGATTAC	*1860*
**PB1 246038 Method K**	*1801*	ATTCGGAATCTCCATATCCCAGAGGTCTGCTTGAAGTGGGAGCTGATGGACGAAGATTAC	*1860*
**PB1 246038 Method N**	*1801*	ATTCGGAATCTCCATATCCCAGAGGTCTGCTTGAAGTGGGAGCTGATGGACGAAGATTAC	*1860*

**PB1 246038 MiSeq**	*1861*	CAGGGCAGGTTGTGTAATCCTCTGAACCCATTTGTCAGTCATAAAGAAATTGAGTCCGTA	*1920*
**PB1 246038 Method A**	*1861*	CAGGGCAGGTTGTGTAATCCTCTGAACCCATTTGTCAGTCATAAAGAAATTGAGTCCGTA	*1920*
**PB1 246038 Method S**	*1861*	CAGGGCAGGTTGTGTAATCCTCTGAACCCATTTGTCAGTCATAAAGAAATTGAGTCCGTA	*1920*
**PB1 246038 Method E**	*1861*	CAGGGCAGGTTGTGTAATCCTCTGAACCCATTTGTCAGTCATAAAGAAATTGAGTCCGTA	*1920*
**PB1 246038 Method K**	*1861*	CAGGGCAGGTTGTGTAATCCTCTGAACCCATTTGTCAGTCATAAAGAAATTGAGTCCGTA	*1920*
**PB1 246038 Method N**	*1861*	CAGGGCAGGTTGTGTAATCCTCTGAACCCATTTGTCAGTCATAAAGAAATTGAGTCCGTA	*1920*

**PB1 246038 MiSeq**	*1921*	AACAATGCTGTGGTGATGCCAGCACACGGTCCAGCCAAAAGCATGGAATATGATGCCATT	*1980*
**PB1 246038 Method A**	*1921*	AACAATGCTGTGGTGATGCCAGCACACGGTCCAGCCAAAAGCATGGAATATGATGCCATT	*1980*
**PB1 246038 Method S**	*1921*	AACAATGCTGTGGTGATGCCAGCACACGGTCCAGCCAAAAGCATGGAATATGATGCCATT	*1980*
**PB1 246038 Method E**	*1921*	AACAATGCTGTGGTGATGCCAGCACACGGTCCAGCCAAAAGCATGGAATATGATGCCATT	*1980*
**PB1 246038 Method K**	*1921*	AACAATGCTGTGGTGATGCCAGCACACGGTCCAGCCAAAAGCATGGAATATGATGCCATT	*1980*
**PB1 246038 Method N**	*1921*	AACAATGCTGTGGTGATGCCAGCACACGGTCCAGCCAAAAGCATGGAATATGATGCCATT	*1980*

**PB1 246038 MiSeq**	*1981*	GCGACTACACACTCATGGATTCCTAAAAGGAATCGTTCCATTCTCAATACCAGTCAAAGG	*2040*
**PB1 246038 Method A**	*1981*	GCGACTACACACTCATGGATTCCTAAAAGGAATCGTTCCATTCTCAATACCAGTCAAAGG	*2040*
**PB1 246038 Method S**	*1981*	GCGACTACACACTCATGGATTCCTAAAAGGAATCGTTCCATTCTCAATACCAGTCAAAGG	*2040*
**PB1 246038 Method E**	*1981*	GCGACTACACACTCATGGATTCCTAAAAGGAATCGTTCCATTCTCAATACCAGTCAAAGG	*2040*
**PB1 246038 Method K**	*1981*	GCGACTACACACTCATGGATTCCTAAAAGGAATCGTTCCATTCTCAATACCAGTCAAAGG	*2040*
**PB1 246038 Method N**	*1981*	GCGACTACACACTCATGGATTCCTAAAAGGAATCGTTCCATTCTCAATACCAGTCAAAGG	*2040*

**PB1 246038 MiSeq**	*2041*	GGAATTCTTGAGGATGAACAGATGTACCAGAAATGCTGCAGTCTATTCGAAAAATTTTTT	*2100*
**PB1 246038 Method A**	*2041*	GGAATTCTTGAGGATGAACAGATGTACCAGAAATGCTGCAGTCTATTCGAAAAATTTTTT	*2100*
**PB1 246038 Method S**	*2041*	GGAATTCTTGAGGATGAACAGATGTACCAGAAATGCTGCAGTCTATTCGAAAAATTTTTT	*2100*
**PB1 246038 Method E**	*2041*	GGAATTCTTGAGGATGAACAGATGTACCAGAAATGCTGCAGTCTATTCGAAAAATTTTTT	*2100*
**PB1 246038 Method K**	*2041*	GGAATTCTTGAGGATGAACAGATGTACCAGAAATGCTGCAGTCTATTCGAAAAATTTTTT	*2100*
**PB1 246038 Method N**	*2041*	GGAATTCTTGAGGATGAACAGATGTACCAGAAATGCTGCAGTCTATTCGAAAAATTTTTT	*2100*

**PB1 246038 MiSeq**	*2101*	CCCAGTAGTTCATACAGGAGACCAGTTGGAATTTCCAGCATGGTGGAGGCCATGGTGTCT	*2160*
**PB1 246038 Method A**	*2101*	CCCAGTAGTTCATACAGGAGACCAGTTGGAATTTCCAGCATGGTGGAGGCCATGGTGTCT	*2160*
**PB1 246038 Method S**	*2101*	CCCAGTAGTTCATACAGGAGACCAGTTGGAATTTCCAGCATGGTGGAGGCCATGGTGTCT	*2160*
**PB1 246038 Method E**	*2101*	CCCAGTAGTTCATACAGGAGACCAGTTGGAATTTCCAGCATGGTGGAGGCCATGGTGTCT	*2160*
**PB1 246038 Method K**	*2101*	CCCAGTAGTTCATACAGGAGACCAGTTGGAATTTCCAGCATGGTGGAGGCCATGGTGTCT	*2160*
**PB1 246038 Method N**	*2101*	CCCAGTAGTTCATACAGGAGACCAGTTGGAATTTCCAGCATGGTGGAGGCCATGGTGTCT	*2160*

**PB1 246038 MiSeq**	*2161*	AGGGCCCGAATCGATGCACGCATTGATTTCGAATCTGGAAGGATCAAGAAGGGAGAGTTT	*2220*
**PB1 246038 Method A**	*2161*	AGGGCCCGAATCGATGCACGCATTGATTTCGAATCTGGAAGGATCAAGAAGGGAGAGTTT	*2220*
**PB1 246038 Method S**	*2161*	AGGGCCCGAATCGATGCACGCATTGATTTCGAATCTGGAAGGATCAAGAAGGGAGAGTTT	*2220*
**PB1 246038 Method E**	*2161*	AGGGCCCGAATCGATGCACGCATTGATTTCGAATCTGGAAGGATCAAGAAGGGAGAGTTT	*2220*
**PB1 246038 Method K**	*2161*	AGGGCCCGAATCGATGCACGCATTGATTTCGAATCTGGAAGGATCAAGAAGGGAGAGTTT	*2220*
**PB1 246038 Method N**	*2161*	AGGGCCCGAATCGATGCACGCATTGATTTCGAATCTGGAAGGATCAAGAAGGGAGAGTTT	*2220*

**PB1 246038 MiSeq**	*2221*	TCTGAGATCATGAAGATCTGTTCCACCATTGAAGAGCTCAGACGGCAAAAATAG	*2274*
**PB1 246038 Method A**	*2221*	TCTGAGATCATGAAGATCTGTTCCACCATTGAAGAGCTCAGACGGCAAAAATAG	*2274*
**PB1 246038 Method S**	*2221*	TCTGAGATCATGAAGATCTGTTCCACCATTGAAGAGCTCAGACGGCAAAAATAG	*2274*
**PB1 246038 Method E**	*2221*	TCTGAGATCATGAAGATCTGTTCCACCATTGAAGAGCTCAGACGGCAAAAATAG	*2274*
**PB1 246038 Method K**	*2221*	TCTGAGATCATGAAGATCTGTTCCACCATTGAAGAGCTCAGACGGCAAAAATAG	*2274*
**PB1 246038 Method N**	*2221*	TCTGAGATCATGAAGATCTGTTCCACCATTGAAGAGCTCAGACGGCAAAAATAG	*2274*
