# Supplementary material for: An Evaluation of Avian Influenza Virus Whole-Genome Sequencing Approaches Using Nanopore Technology
Source: Microorganisms. 2023 Feb 19;11(2):529. doi: 10.3390/microorganisms11020529 (PMC9967579; doi:10.3390/microorganisms11020529)
Supplement: Supplementary file 1 [file microorganisms-11-00529-s001.zip › manuscript.v8 230219 Suppl Figures and Tables/Supplementary Figures S3a-h 246038/Supplementary Figure S3a PB2.pdf]

Formatted Alignments

**PB2 246038 MiSeq**	*1*	ATGGAGAGAATAAAAGAACTGATAGATCTAATGTCACAGTCTCGCACTCGCGAGATACTA	*60*
**PB2 246038 Method A**	*1*	ATGGAGAGAATAAAAGAACTGATAGATCTAATGTCACAGTCTCGCACTCGCGAGATACTA	*60*
**PB2 246038 Method S**	*1*	ATGGAGAGAATAAAAGAACTGATAGATCTAATGTCACAGTCTCGCACTCGCGAGATACTA	*60*
**PB2 246038 Method E**	*1*	ATGGAGAGAATAAAAGAACTGATAGATCTAATGTCACAGTCTCGCACTCGCGAGATACTA	*60*
**PB2 246038 Method K**	*1*	ATGGAGAGAATAAAAGAACTGATAGATCTAATGTCACAGTCTCGCACTCGCGAGATACTA	*60*
**PB2 246038 Method N**	*1*	ATGGAGAGAATAAAAGAACTGATAGATCTAATGTCACAGTCTCGCACTCGCGAGATACTA	*60*

**PB2 246038 MiSeq**	*61*	ACCAAAACCACTGTTGACCACATGGCCATAATCAAGAAGTACACATCAGGAAGACAAGAA	*120*
**PB2 246038 Method A**	*61*	ACCAAAACCACTGTTGACCACATGGCCATAATCAAGAAGTACACATCAGGAAGACAAGAA	*120*
**PB2 246038 Method S**	*61*	ACCAAAACCACTGTTGACCACATGGCCATAATCAAGAAGTACACATCAGGAAGACAAGAA	*120*
**PB2 246038 Method E**	*61*	ACCAAAACCACTGTTGACCACATGGCCATAATCAAGAAGTACACATCAGGAAGACAAGAA	*120*
**PB2 246038 Method K**	*61*	ACCAAAACCACTGTTGACCACATGGCCATAATCAAGAAGTACACATCAGGAAGACAAGAA	*120*
**PB2 246038 Method N**	*61*	ACCAAAACCACTGTTGACCACATGGCCATAATCAAGAAGTACACATCAGGAAGACAAGAA	*120*

**PB2 246038 MiSeq**	*121*	AAGAACCCTGCACTCAGAATGAAATGGATGATGGCAATGAAATATCCAATCACAGCAGAC	*180*
**PB2 246038 Method A**	*121*	AAGAACCCTGCACTCAGAATGAAATGGATGATGGCAATGAAATATCCAATCACAGCAGAC	*180*
**PB2 246038 Method S**	*121*	AAGAACCCTGCACTCAGAATGAAATGGATGATGGCAATGAAATATCCAATCACAGCAGAC	*180*
**PB2 246038 Method E**	*121*	AAGAACCCTGCACTCAGAATGAAATGGATGATGGCAATGAAATATCCAATCACAGCAGAC	*180*
**PB2 246038 Method K**	*121*	AAGAACCCTGCACTCAGAATGAAATGGATGATGGCAATGAAATATCCAATCACAGCAGAC	*180*
**PB2 246038 Method N**	*121*	AAGAACCCTGCACTCAGAATGAAATGGATGATGGCAATGAAATATCCAATCACAGCAGAC	*180*

**PB2 246038 MiSeq**	*181*	AAGCGAATAATGGAAATGATCCCTGAAAGGAATGAACAAGGACAAACCCTCTGGAGCAAG	*240*
**PB2 246038 Method A**	*181*	AAGCGAATAATGGAAATGATCCCTGAAAGGAATGAACAAGGACAAACCCTCTGGAGCAAG	*240*
**PB2 246038 Method S**	*181*	AAGCGAATAATGGAAATGATCCCTGAAAGGAATGAACAAGGACAAACCCTCTGGAGCAAG	*240*
**PB2 246038 Method E**	*181*	AAGCGAATAATGGAAATGATCCCTGAAAGGAATGAACAAGGACAAACCCTCTGGAGCAAG	*240*
**PB2 246038 Method K**	*181*	AAGCGAATAATGGAAATGATCCCTGAAAGGAATGAACAAGGACAAACCCTCTGGAGCAAG	*240*
**PB2 246038 Method N**	*181*	AAGCGAATAATGGAAATGATCCCTGAAAGGAATGAACAAGGACAAACCCTCTGGAGCAAG	*240*

**PB2 246038 MiSeq**	*241*	ACAAATGATGCCGGATCAGATCGAGTGATGGTATCACCCCTGGCTGTGACATGGTGGAAT	*300*
**PB2 246038 Method A**	*241*	ACAAATGATGCCGGATCAGATCGAGTGATGGTATCACCCCTGGCTGTGACATGGTGGAAT	*300*
**PB2 246038 Method S**	*241*	ACAAATGATGCCGGATCAGATCGAGTGATGGTATCACCCCTGGCTGTGACATGGTGGAAT	*300*
**PB2 246038 Method E**	*241*	ACAAATGATGCCGGATCAGATCGAGTGATGGTATCACCCCTGGCTGTGACATGGTGGAAT	*300*
**PB2 246038 Method K**	*241*	ACAAATGATGCCGGATCAGATCGAGTGATGGTATCACCCCTGGCTGTGACATGGTGGAAT	*300*
**PB2 246038 Method N**	*241*	ACAAATGATGCCGGATCAGATCGAGTGATGGTATCACCCCTGGCTGTGACATGGTGGAAT	*300*

**PB2 246038 MiSeq**	*301*	AGGAGTGGACCAACAACAAGTACAGTTCACTATCCAAAGGTATACAAAACTTATTTTGAA	*360*
**PB2 246038 Method A**	*301*	AGGAGTGGACCAACAACAAGTACAGTTCACTATCCAAAGGTATACAAAACTTATTTTGAA	*360*
**PB2 246038 Method S**	*301*	AGGAGTGGACCAACAACAAGTACAGTTCACTATCCAAAGGTATACAAAACTTATTTTGAA	*360*
**PB2 246038 Method E**	*301*	AGGAGTGGACCAACAACAAGTACAGTTCACTATCCAAAGGTATACAAAACTTATTTTGAA	*360*
**PB2 246038 Method K**	*301*	AGGAGTGGACCAACAACAAGTACAGTTCACTATCCAAAGGTATACAAAACTTATTTTGAA	*360*
**PB2 246038 Method N**	*301*	AGGAGTGGACCAACAACAAGTACAGTTCACTATCCAAAGGTATACAAAACTTATTTTGAA	*360*

**PB2 246038 MiSeq**	*361*	AAAGTTGAAAGGTTGAAACACGGGACCTTTGGCCCTGTACACTTCAGAAACCAAGTTAAG	*420*
**PB2 246038 Method A**	*361*	AAAGTTGAAAGGTTGAAACACGGGACCTTTGGCCCTGTACACTTCAGAAACCAAGTTAAG	*420*
**PB2 246038 Method S**	*361*	AAAGTTGAAAGGTTGAAACACGGGACCTTTGGCCCTGTACACTTCAGAAACCAAGTTAAG	*420*
**PB2 246038 Method E**	*361*	AAAGTTGAAAGGTTGAAACACGGGACCTTTGGCCCTGTACACTTCAGAAACCAAGTTAAG	*420*
**PB2 246038 Method K**	*361*	AAAGTTGAAAGGTTGAAACACGGGACCTTTGGCCCTGTACACTTCAGAAACCAAGTTAAG	*420*
**PB2 246038 Method N**	*361*	AAAGTTGAAAGGTTGAAACACGGGACCTTTGGCCCTGTACACTTCAGAAACCAAGTTAAG	*420*

**PB2 246038 MiSeq**	*421*	ATAAGACGGAGGGTCGACATAAACCCGGGCCATGCTGACCTCAGCGCCAAAGAGGCGCAG	*480*
**PB2 246038 Method A**	*421*	ATAAGACGGAGGGTCGACATAAACCCGGGCCATGCTGACCTCAGCGCCAAAGAGGCGCAG	*480*
**PB2 246038 Method S**	*421*	ATAAGACGGAGGGTCGACATAAACCCGGGCCATGCTGACCTCAGCGCCAAAGAGGCGCAG	*480*
**PB2 246038 Method E**	*421*	ATAAGACGGAGGGTCGACATAAACCCGGGCCATGCTGACCTCAGCGCCAAAGAGGCGCAG	*480*
**PB2 246038 Method K**	*421*	ATAAGACGGAGGGTCGACATAAACCCGGGCCATGCTGACCTCAGCGCCAAAGAGGCGCAG	*480*
**PB2 246038 Method N**	*421*	ATAAGACGGAGGGTCGACATAAACCCGGGCCATGCTGACCTCAGCGCCAAAGAGGCGCAG	*480*

**PB2 246038 MiSeq**	*481*	GACGTAATCATGGAAGTTGTCTTTCCAAATGAAGTGGGAGCGAGAATACTGACGTCGGAG	*540*
**PB2 246038 Method A**	*481*	GACGTAATCATGGAAGTTGTCTTTCCAAATGAAGTGGGAGCGAGAATACTGACGTCGGAG	*540*
**PB2 246038 Method S**	*481*	GACGTAATCATGGAAGTTGTCTTTCCAAATGAAGTGGGAGCGAGAATACTGACGTC**A**GAG	*540*
**PB2 246038 Method E**	*481*	GACGTAATCATGGAAGTTGTCTTTCCAAATGAAGTGGGAGCGAGAATACTGACGTCGGAG	*540*
**PB2 246038 Method K**	*481*	GACGTAATCATGGAAGTTGTCTTTCCAAATGAAGTGGGAGCGAGAATACTGACGTCGGAG	*540*
**PB2 246038 Method N**	*481*	GACGTAATCATGGAAGTTGTCTTTCCAAATGAAGTGGGAGCGAGAATACTGACGTCGGAG	*540*

**PB2 246038 MiSeq**	*541*	TCACAGTTGACAATAACAAAGGAAAAGAAAGAAGAACTCCAGGACTGCAAAATCGCCCCT	*600*
**PB2 246038 Method A**	*541*	TCACAGTTGACAATAACAAAGGAAAAGAAAGAAGAACTCCAGGACTGCAAAATCGCCCCT	*600*
**PB2 246038 Method S**	*541*	TCACAGTTGACAATAACAAAGGAAAAGAAAGAAGAACTCCAGGACTGCAAAATCGCCCCT	*600*
**PB2 246038 Method E**	*541*	TCACAGTTGACAATAACAAAGGAAAAGAAAGAAGAACTCCAGGACTGCAAAATCGCCCCT	*600*
**PB2 246038 Method K**	*541*	TCACAGTTGACAATAACAAAGGAAAAGAAAGAAGAACTCCAGGACTGCAAAATCGCCCCT	*600*
**PB2 246038 Method N**	*541*	TCACAGTTGACAATAACAAAGGAAAAGAAAGAAGAACTCCAGGACTGCAAAATCGCCCCT	*600*

**PB2 246038 MiSeq**	*601*	CTGATGGTTGCATACATGCTAGAAAGAGAGCTGGTCCGCAAGACAAGGTTCCTCCCAGTG	*660*
**PB2 246038 Method A**	*601*	CTGATGGTTGCATACATGCTAGAAAGAGAGCTGGTCCGCAAGACAAGGTTCCTCCCAGTG	*660*
**PB2 246038 Method S**	*601*	CTGATGGTTGCATACATGCTAGAAAGAGAGCTGGTCCGCAAGACAAGGTTCCTCCCAGTG	*660*
**PB2 246038 Method E**	*601*	CTGATGGTTGCATACATGCTAGAAAGAGAGCTGGTCCGCAAGACAAGGTTCCTCCCAGTG	*660*
**PB2 246038 Method K**	*601*	CTGATGGTTGCATACATGCTAGAAAGAGAGCTGGTCCGCAAGACAAGGTTCCTCCCAGTG	*660*
**PB2 246038 Method N**	*601*	CTGATGGTTGCATACATGCTAGAAAGAGAGCTGGTCCGCAAGACAAGGTTCCTCCCAGTG	*660*

**PB2 246038 MiSeq**	*661*	GCTGGTGGAACAAGCAGTGTCTACATTGAGGTGCTGCATTTGACCCAGGGAACATGCTGG	*720*
**PB2 246038 Method A**	*661*	GCTGGTGGAACAAGCAGTGTCTACATTGAGGTGCTGCATTTGACCCAGGGAACATGCTGG	*720*
**PB2 246038 Method S**	*661*	GCTGGTGGAACAAGCAGTGTCTACATTGAGGTGCTGCATTTGACCCAGGGAACATGCTGG	*720*
**PB2 246038 Method E**	*661*	GCTGGTGGAACAAGCAGTGTCTACATTGAGGTGCTGCATTTGACCCAGGGAACATGCTGG	*720*
**PB2 246038 Method K**	*661*	GCTGGTGGAACAAGCAGTGTCTACATTGAGGTGCTGCATTTGACCCAGGGAACATGCTGG	*720*
**PB2 246038 Method N**	*661*	GCTGGTGGAACAAGCAGTGTCTACATTGAGGTGCTGCATTTGACCCAGGGAACATGCTGG	*720*

**PB2 246038 MiSeq**	*721*	GAGCAGATGTATACTCCAGGAGGAGAAGTGAGAAACGATGATGTAGACCAGAGCTTAATC	*780*
**PB2 246038 Method A**	*721*	GAGCAGATGTATACTCCAGGAGGAGAAGTGAGAAACGATGATGTAGACCAGAGCTTAATC	*780*
**PB2 246038 Method S**	*721*	GAGCAGATGTATACTCCAGGAGGAGAAGTGAGAAACGATGATGTAGACCAGAGCTTAATC	*780*
**PB2 246038 Method E**	*721*	GAGCAGATGTATACTCCAGGAGGAGAAGTGAGAAACGATGATGTAGACCAGAGCTTAATC	*780*
**PB2 246038 Method K**	*721*	GAGCAGATGTATACTCCAGGAGGAGAAGTGAGAAACGATGATGTAGACCAGAGCTTAATC	*780*
**PB2 246038 Method N**	*721*	GAGCAGATGTATACTCCAGGAGGAGAAGTGAGAAACGATGATGTAGACCAGAGCTTAATC	*780*

**PB2 246038 MiSeq**	*781*	ATTGCTGCCCGGAATATAGTAAGAAGAGCAACAGTGTCAGCAGACCCATTAGCATCTCTA	*840*
**PB2 246038 Method A**	*781*	ATTGCTGCCCGGAATATAGTAAGAAGAGCAACAGTGTCAGCAGACCCATTAGCATCTCTA	*840*
**PB2 246038 Method S**	*781*	ATTGCTGCCCGGAATATAGTAAGAAGAGCAACAGTGTCAGCAGACCCATTAGCATCTCTA	*840*
**PB2 246038 Method E**	*781*	ATTGCTGCCCGGAATATAGTAAGAAGAGCAACAGTGTCAGCAGACCCATTAGCATCTCTA	*840*
**PB2 246038 Method K**	*781*	ATTGCTGCCCGGAATATAGTAAGAAGAGCAACAGTGTCAGCAGACCCATTAGCATCTCTA	*840*
**PB2 246038 Method N**	*781*	ATTGCTGCCCGGAATATAGTAAGAAGAGCAACAGTGTCAGCAGACCCATTAGCATCTCTA	*840*

**PB2 246038 MiSeq**	*841*	TTGGAGATGTGCCACAGCACACAAATTGGAGGAATAAGGATGGTAGACATTCTTCGGCAA	*900*
**PB2 246038 Method A**	*841*	TTGGAGATGTGCCACAGCACACAAATTGGAGGAATAAGGATGGTAGACATTCTTCGGCAA	*900*
**PB2 246038 Method S**	*841*	TTGGAGATGTGCCACAGCACACAAATTGGAGGAATAAGGATGGTAGACATTCTTCGGCAA	*900*
**PB2 246038 Method E**	*841*	TTGGAGATGTGCCACAGCACACAAATTGGAGGAATAAGGATGGTAGACATTCTTCGGCAA	*900*
**PB2 246038 Method K**	*841*	TTGGAGATGTGCCACAGCACACAAATTGGAGGAATAAGGATGGTAGACATTCTTCGGCAA	*900*
**PB2 246038 Method N**	*841*	TTGGAGATGTGCCACAGCACACAAATTGGAGGAATAAGGATGGTAGACATTCTTCGGCAA	*900*

**PB2 246038 MiSeq**	*901*	AATCCAACGGAGGAACAAGCCGTGGACATATGCAAGGCAGCAATGGGCTTGAGGATTAGC	*960*
**PB2 246038 Method A**	*901*	AATCCAACGGAGGAACAAGCCGTGGACATATGCAAGGCAGCAATGGGCTTGAGGATTAGC	*960*
**PB2 246038 Method S**	*901*	AATCCAACGGAGGAACAAGCCGTGGACATATGCAAGGCAGCAATGGGCTTGAGGATTAGC	*960*
**PB2 246038 Method E**	*901*	AATCCAACGGAGGAACAAGCCGTGGACATATGCAAGGCAGCAATGGGCTTGAGGATTAGC	*960*
**PB2 246038 Method K**	*901*	AATCCAACGGAGGAACAAGCCGTGGACATATGCAAGGCAGCAATGGGCTTGAGGATTAGC	*960*
**PB2 246038 Method N**	*901*	AATCCAACGGAGGAACAAGCCGTGGACATATGCAAGGCAGCAATGGGCTTGAGGATTAGC	*960*

**PB2 246038 MiSeq**	*961*	TCATCGTTCAGCTTTGGTGGATTCACTTTTAAAAGAACAAGTGGATCGTCAATCAAAAGG	*1020*
**PB2 246038 Method A**	*961*	TCATCGTTCAGCTTTGGTGGATTCACTTTTAAAAGAACAAGTGGATCGTCAATCAAAAGG	*1020*
**PB2 246038 Method S**	*961*	TCATCGTTCAGCTTTGGTGGATTCACTTTTAAAAGAACAAGTGGATCGTCAATCAAAAGG	*1020*
**PB2 246038 Method E**	*961*	TCATCGTTCAGCTTTGGTGGATTCACTTTTAAAAGAACAAGTGGATCGTCAATCAAAAGG	*1020*
**PB2 246038 Method K**	*961*	TCATCGTTCAGCTTTGGTGGATTCACTTTTAAAAGAACAAGTGGATCGTCAATCAAAAGG	*1020*
**PB2 246038 Method N**	*961*	TCATCGTTCAGCTTTGGTGGATTCACTTTTAAAAGAACAAGTGGATCGTCAATCAAAAGG	*1020*

**PB2 246038 MiSeq**	*1021*	GAAGAAGAGGTGCTTACGGGCAACCTTCAAACATTGAAAATAAGAGTACATGAGGGGTAT	*1080*
**PB2 246038 Method A**	*1021*	GAAGAAGAGGTGCTTACGGGCAACCTTCAAACATTGAAAATAAGAGTACATGAGGGGTAT	*1080*
**PB2 246038 Method S**	*1021*	GAAGAAGAGGTGCTTACGGGCAACCTTCAAACATTGAAAATAAGAGTACATGAGGGGTAT	*1080*
**PB2 246038 Method E**	*1021*	GAAGAAGAGGTGCTTACGGGCAACCTTCAAACATTGAAAATAAGAGTACATGAGGGGTAT	*1080*
**PB2 246038 Method K**	*1021*	GAAGAAGAGGTGCTTACGGGCAACCTTCAAACATTGAAAATAAGAGTACATGAGGGGTAT	*1080*
**PB2 246038 Method N**	*1021*	GAAGAAGAGGTGCTTACGGGCAACCTTCAAACATTGAAAATAAGAGTACATGAGGGGTAT	*1080*

**PB2 246038 MiSeq**	*1081*	GAAGAGTTCACTATGGTTGGAAGAAGAGCAACGGCCATCCTCAGGAAAGCAACCAGAAGG	*1140*
**PB2 246038 Method A**	*1081*	GAAGAGTTCACTATGGTTGGAAGAAGAGCAACGGCCATCCTCAGGAAAGCAACCAGAAGG	*1140*
**PB2 246038 Method S**	*1081*	GAAGAGTTCACTATGGTTGGAAGAAGAGCAACGGCCATCCTCAGGAAAGCAACCAGAAGG	*1140*
**PB2 246038 Method E**	*1081*	GAAGAGTTCACTATGGTTGGAAGAAGAGCAACGGCCATCCTCAGGAAAGCAACCAGAAGG	*1140*
**PB2 246038 Method K**	*1081*	GAAGAGTTCACTATGGTTGGAAGAAGAGCAACGGCCATCCTCAGGAAAGCAACCAGAAGG	*1140*
**PB2 246038 Method N**	*1081*	GAAGAGTTCACTATGGTTGGAAGAAGAGCAACGGCCATCCTCAGGAAAGCAACCAGAAGG	*1140*

**PB2 246038 MiSeq**	*1141*	CTGATCCAGCTAATAGTAAGTGGAAGGGACGAACAGTCAATTGCTGAAGCAATAATCGTG	*1200*
**PB2 246038 Method A**	*1141*	CTGATCCAGCTAATAGTAAGTGGAAGGGACGAACAGTCAATTGCTGAAGCAATAATCGTG	*1200*
**PB2 246038 Method S**	*1141*	CTGATCCAGCTAATAGTAAGTGGAAGGGACGAACAGTCAATTGCTGAAGCAATAATCGTG	*1200*
**PB2 246038 Method E**	*1141*	CTGATCCAGCTAATAGTAAGTGGAAGGGACGAACAGTCAATTGCTGAAGCAATAATCGTG	*1200*
**PB2 246038 Method K**	*1141*	CTGATCCAGCTAATAGTAAGTGGAAGGGACGAACAGTCAATTGCTGAAGCAATAATCGTG	*1200*
**PB2 246038 Method N**	*1141*	CTGATCCAGCTAATAGTAAGTGGAAGGGACGAACAGTCAATTGCTGAAGCAATAATCGTG	*1200*

**PB2 246038 MiSeq**	*1201*	GCCATGGTATTCTCACAAGAGGACTGCATGATAAAGGCAGTTCGAGGTGATCTGAACTTT	*1260*
**PB2 246038 Method A**	*1201*	GCCATGGTATTCTCACAAGAGGACTGCATGATAAAGGCAGTTCGAGGTGATCTGAACTTT	*1260*
**PB2 246038 Method S**	*1201*	GCCATGGTATTCTCACAAGAGGACTGCATGATAAAGGCAGTTCGAGGTGATCTGAACTTT	*1260*
**PB2 246038 Method E**	*1201*	GCCATGGTATTCTCACAAGAGGACTGCATGATAAAGGCAGTTCGAGGTGATCTGAACTTT	*1260*
**PB2 246038 Method K**	*1201*	GCCATGGTATTCTCACAAGAGGACTGCATGATAAAGGCAGTTCGAGGTGATCTGAACTTT	*1260*
**PB2 246038 Method N**	*1201*	GCCATGGTATTCTCACAAGAGGACTGCATGATAAAGGCAGTTCGAGGTGATCTGAACTTT	*1260*

**PB2 246038 MiSeq**	*1261*	GTCAATAGAGCGAATCAGCGGCTGAATCCAATGCATCAGCTCTTGAGACACTTCCAAAAG	*1320*
**PB2 246038 Method A**	*1261*	GTCAATAGAGCGAATCAGCGGCTGAATCCAATGCATCAGCTCTTGAGACACTTCCAAAAG	*1320*
**PB2 246038 Method S**	*1261*	GTCAATAGAGCGAATCAGCGGCTGAATCCAATGCATCAGCTCTTGAGACACTTCCAAAAG	*1320*
**PB2 246038 Method E**	*1261*	GTCAATAGAGCGAATCAGCGGCTGAATCCAATGCATCAGCTCTTGAGACACTTCCAAAAG	*1320*
**PB2 246038 Method K**	*1261*	GTCAATAGAGCGAATCAGCGGCTGAATCCAATGCATCAGCTCTTGAGACACTTCCAAAAG	*1320*
**PB2 246038 Method N**	*1261*	GTCAATAGAGCGAATCAGCGGCTGAATCCAATGCATCAGCTCTTGAGACACTTCCAAAAG	*1320*

**PB2 246038 MiSeq**	*1321*	GATGCAAAAGTGCTTTTCCAAAATTGGGGAATTGAGCCCATTGACAATGTGATGGGAATG	*1380*
**PB2 246038 Method A**	*1321*	GATGCAAAAGTGCTTTTCCAAAATTGGGGAATTGAGCCCATTGACAATGTGATGGGAATG	*1380*
**PB2 246038 Method S**	*1321*	GATGCAAAAGTGCTTTTCCAAAATTGGGGAATTGAGCCCATTGACAATGTGATGGGAATG	*1380*
**PB2 246038 Method E**	*1321*	GATGCAAAAGTGCTTTTCCAAAATTGGGGAATTGAGCCCATTGACAATGTGATGGGAATG	*1380*
**PB2 246038 Method K**	*1321*	GATGCAAAAGTGCTTTTCCAAAATTGGGGAATTGAGCCCATTGACAATGTGATGGGAATG	*1380*
**PB2 246038 Method N**	*1321*	GATGCAAAAGTGCTTTTCCAAAATTGGGGAATTGAGCCCATTGACAATGTGATGGGAATG	*1380*

**PB2 246038 MiSeq**	*1381*	ATCGGGATATTGCCTGACATGACTCCAAGTACTGAGATGTCACTGAGGGGAATAAGAGTC	*1440*
**PB2 246038 Method A**	*1381*	ATCGGGATATTGCCTGACATGACTCCAAGTACTGAGATGTCACTGAGGGGAATAAGAGTC	*1440*
**PB2 246038 Method S**	*1381*	ATCGGGATATTGCCTGACATGACTCCAAGTACTGAGATGTCACTGAGGGGAATAAGAGTC	*1440*
**PB2 246038 Method E**	*1381*	ATCGGGATATTGCCTGACATGACTCCAAGTACTGAGATGTCACTGAGGGGAATAAGAGTC	*1440*
**PB2 246038 Method K**	*1381*	ATCGGGATATTGCCTGACATGACTCCAAGTACTGAGATGTCACTGAGGGGAATAAGAGTC	*1440*
**PB2 246038 Method N**	*1381*	ATCGGGATATTGCCTGACATGACTCCAAGTACTGAGATGTCACTGAGGGGAATAAGAGTC	*1440*

**PB2 246038 MiSeq**	*1441*	AGTAAGATGGGAGTAGATGAATACTCCAGTACAGAGCGGGTAGTAGTAAGCATCGATCGA	*1500*
**PB2 246038 Method A**	*1441*	AGTAAGATGGGAGTAGATGAATACTCCAGTACAGAGCGGGTAGTAGTAAGCATCGATCGA	*1500*
**PB2 246038 Method S**	*1441*	AGTAAGATGGGAGTAGATGAATACTCCAGTACAGAGCGGGTAGTAGTAAGCATCGATCGA	*1500*
**PB2 246038 Method E**	*1441*	AGTAAGATGGGAGTAGATGAATACTCCAGTACAGAGCGGGTAGTAGTAAGCATCGATCGA	*1500*
**PB2 246038 Method K**	*1441*	AGTAAGATGGGAGTAGATGAATACTCCAGTACAGAGCGGGTAGTAGTAAGCATCGATCGA	*1500*
**PB2 246038 Method N**	*1441*	AGTAAGATGGGAGTAGATGAATACTCCAGTACAGAGCGGGTAGTAGTAAGCATCGATCGA	*1500*

**PB2 246038 MiSeq**	*1501*	TTTTTAAGAGTTCGAGACCAACGGGGGAGCGTACTACTGTCACCCGAAGAAGTCAGCGAG	*1560*
**PB2 246038 Method A**	*1501*	TTTTTAAGAGTTCGAGACCAACGGGGGAGCGTACTACTGTCACCCGAAGAAGTCAGCGAG	*1560*
**PB2 246038 Method S**	*1501*	TTTTTAAGAGTTCGAGACCAACGGGGGAGCGTACTACTGTCACCCGAAGAAGTCAGCGAG	*1560*
**PB2 246038 Method E**	*1501*	TTTTTAAGAGTTCGAGACCAACGGGGGAGCGTACTACTGTCACCCGAAGAAGTCAGCGAG	*1560*
**PB2 246038 Method K**	*1501*	TTTTTAAGAGTTCGAGACCAACGGGGGAGCGTACTACTGTCACCCGAAGAAGTCAGCGAG	*1560*
**PB2 246038 Method N**	*1501*	TTTTTAAGAGTTCGAGACCAACGGGGGAGCGTACTACTGTCACCCGAAGAAGTCAGCGAG	*1560*

**PB2 246038 MiSeq**	*1561*	ACACAAGGAACAGAGAAATTGACAATCACTTATTCGTCATCAATGATGTGGGAGATCAAT	*1620*
**PB2 246038 Method A**	*1561*	ACACAAGGAACAGAGAAATTGACAATCACTTATTCGTCATCAATGATGTGGGAGATCAAT	*1620*
**PB2 246038 Method S**	*1561*	ACACAAGGAACAGAGAAATTGACAATCACTTATTCGTCATCAATGATGTGGGAGATCAAT	*1620*
**PB2 246038 Method E**	*1561*	ACACAAGGAACAGAGAAATTGACAATCACTTATTCGTCATCAATGATGTGGGAGATCAAT	*1620*
**PB2 246038 Method K**	*1561*	ACACAAGGAACAGAGAAATTGACAATCACTTATTCGTCATCAATGATGTGGGAGATCAAT	*1620*
**PB2 246038 Method N**	*1561*	ACACAAGGAACAGAGAAATTGACAATCACTTATTCGTCATCAATGATGTGGGAGATCAAT	*1620*

**PB2 246038 MiSeq**	*1621*	GGTCCTGAGTCGGTGTTGGTCAATACTTATCAGTGGATAATCAGAAACTGGGAAACAGTA	*1680*
**PB2 246038 Method A**	*1621*	GGTCCTGAGTCGGTGTTGGTCAATACTTATCAGTGGATAATCAGAAACTGGGAAACAGTA	*1680*
**PB2 246038 Method S**	*1621*	GGTCCTGAGTCGGTGTTGGTCAATACTTATCAGTGGATAATCAGAAACTGGGAAACAGTA	*1680*
**PB2 246038 Method E**	*1621*	GGTCCTGAGTCGGTGTTGGTCAATACTTATCAGTGGATAATCAGAAACTGGGAAACAGTA	*1680*
**PB2 246038 Method K**	*1621*	GGTCCTGAGTCGGTGTTGGTCAATACTTATCAGTGGATAATCAGAAACTGGGAAACAGTA	*1680*
**PB2 246038 Method N**	*1621*	GGTCCTGAGTCGGTGTTGGTCAATACTTATCAGTGGATAATCAGAAACTGGGAAACAGTA	*1680*

**PB2 246038 MiSeq**	*1681*	AAAATTCAATGGTCACAAGATCCCACAATGTTGTATAATAAGATGGAGTTCGAGCCATTC	*1740*
**PB2 246038 Method A**	*1681*	AAAATTCAATGGTCACAAGATCCCACAATGTTGTATAATAAGATGGAGTTCGAGCCATTC	*1740*
**PB2 246038 Method S**	*1681*	AAAATTCAATGGTCACAAGATCCCACAATGTTGTATAATAAGATGGAGTTCGAGCCATTC	*1740*
**PB2 246038 Method E**	*1681*	AAAATTCAATGGTCACAAGATCCCACAATGTTGTATAATAAGATGGAGTTCGAGCCATTC	*1740*
**PB2 246038 Method K**	*1681*	AAAATTCAATGGTCACAAGATCCCACAATGTTGTATAATAAGATGGAGTTCGAGCCATTC	*1740*
**PB2 246038 Method N**	*1681*	AAAATTCAATGGTCACAAGATCCCACAATGTTGTATAATAAGATGGAGTTCGAGCCATTC	*1740*

**PB2 246038 MiSeq**	*1741*	CAGTCTCTGGTCCCTAAGGCAGCCAGGGGTCAATACAGTGGGTTCGTGAGGACACTATTT	*1800*
**PB2 246038 Method A**	*1741*	CAGTCTCTGGTCCCTAAGGCAGCCAGGGGTCAATACAGTGGGTTCGTGAGGACACTATTT	*1800*
**PB2 246038 Method S**	*1741*	CAGTCTCTGGTCCCTAAGGCAGCCAGGGGTCAATACAGTGGGTTCGTGAGGACACTATTT	*1800*
**PB2 246038 Method E**	*1741*	CAGTCTCTGGTCCCTAAGGCAGCCAGGGGTCAATACAGTGGGTTCGTGAGGACACTATTT	*1800*
**PB2 246038 Method K**	*1741*	CAGTCTCTGGTCCCTAAGGCAGCCAGGGGTCAATACAGTGGGTTCGTGAGGACACTATTT	*1800*
**PB2 246038 Method N**	*1741*	CAGTCTCTGGTCCCTAAGGCAGCCAGGGGTCAATACAGTGGGTTCGTGAGGACACTATTT	*1800*

**PB2 246038 MiSeq**	*1801*	CAGCAAATGCGAGATGTGCTTGGAACATTTGACACTGTTCAGATAATAAAACTTCTCCCC	*1860*
**PB2 246038 Method A**	*1801*	CAGCAAATGCGAGATGTGCTTGGAACATTTGACACTGTTCAGATAATAAAACTTCTCCCC	*1860*
**PB2 246038 Method S**	*1801*	CAGCAAATGCGAGATGTGCTTGGAACATTTGACACTGTTCAGATAATAAAACTTCTCCCC	*1860*
**PB2 246038 Method E**	*1801*	CAGCAAATGCGAGATGTGCTTGGAACATTTGACACTGTTCAGATAATAAAACTTCTCCCC	*1860*
**PB2 246038 Method K**	*1801*	CAGCAAATGCGAGATGTGCTTGGAACATTTGACACTGTTCAGATAATAAAACTTCTCCCC	*1860*
**PB2 246038 Method N**	*1801*	CAGCAAATGCGAGATGTGCTTGGAACATTTGACACTGTTCAGATAATAAAACTTCTCCCC	*1860*

**PB2 246038 MiSeq**	*1861*	TTTGCTGCTGCCCCACCGGAACAAAGTAGGATGCAATTCTCCTCTCTGACTGTGAATGTG	*1920*
**PB2 246038 Method A**	*1861*	TTTGCTGCTGCCCCACCGGAACAAAGTAGGATGCAATTCTCCTCTCTGACTGTGAATGTG	*1920*
**PB2 246038 Method S**	*1861*	TTTGCTGCTGCCCCACCGGAACAAAGTAGGATGCAATTCTCCTCTCTGACTGTGAATGTG	*1920*
**PB2 246038 Method E**	*1861*	TTTGCTGCTGCCCCACCGGAACAAAGTAGGATGCAATTCTCCTCTCTGACTGTGAATGTG	*1920*
**PB2 246038 Method K**	*1861*	TTTGCTGCTGCCCCACCGGAACAAAGTAGGATGCAATTCTCCTCTCTGACTGTGAATGTG	*1920*
**PB2 246038 Method N**	*1861*	TTTGCTGCTGCCCCACCGGAACAAAGTAGGATGCAATTCTCCTCTCTGACTGTGAATGTG	*1920*

**PB2 246038 MiSeq**	*1921*	AGAGGATCAGGAATGAGAATACTGGTAAGAGGCAATTCTCCAGTGTTCAATTACAACAAG	*1980*
**PB2 246038 Method A**	*1921*	AGAGGATCAGGAATGAGAATACTGGTAAGAGGCAATTCTCCAGTGTTCAATTACAACAAG	*1980*
**PB2 246038 Method S**	*1921*	AGAGGATCAGGAATGAGAATACTGGTAAGAGGCAATTCTCCAGTGTTCAATTACAACAAG	*1980*
**PB2 246038 Method E**	*1921*	AGAGGATCAGGAATGAGAATACTGGTAAGAGGCAATTCTCCAGTGTTCAATTACAACAAG	*1980*
**PB2 246038 Method K**	*1921*	AGAGGATCAGGAATGAGAATACTGGTAAGAGGCAATTCTCCAGTGTTCAATTACAACAAG	*1980*
**PB2 246038 Method N**	*1921*	AGAGGATCAGGAATGAGAATACTGGTAAGAGGCAATTCTCCAGTGTTCAATTACAACAAG	*1980*

**PB2 246038 MiSeq**	*1981*	GCCACCAAGAGGCTCACAATTCTCGGGAAAGATGCAGGTGCATTGACCGAAGACCCAGAT	*2040*
**PB2 246038 Method A**	*1981*	GCCACCAAGAGGCTCACAATTCTCGGGAAAGATGCAGGTGCATTGACCGAAGACCCAGAT	*2040*
**PB2 246038 Method S**	*1981*	GCCACCAAGAGGCTCACAATTCTCGGGAAAGATGCAGGTGCATTGACCGAAGACCCAGAT	*2040*
**PB2 246038 Method E**	*1981*	GCCACCAAGAGGCTCACAATTCTCGGGAAAGATGCAGGTGCATTGACCGAAGACCCAGAT	*2040*
**PB2 246038 Method K**	*1981*	GCCACCAAGAGGCTCACAATTCTCGGGAAAGATGCAGGTGCATTGACCGAAGACCCAGAT	*2040*
**PB2 246038 Method N**	*1981*	GCCACCAAGAGGCTCACAATTCTCGGGAAAGATGCAGGTGCATTGACCGAAGACCCAGAT	*2040*

**PB2 246038 MiSeq**	*2041*	GAAGGCACAGCTGGAGTGGAGTCTGCTGTTTTAAGAGGATTCCTCATTTTGGGCAAAGAA	*2100*
**PB2 246038 Method A**	*2041*	GAAGGCACAGCTGGAGTGGAGTCTGCTGTTTTAAGAGGATTCCTCATTTTGGGCAAAGAA	*2100*
**PB2 246038 Method S**	*2041*	GAAGGCACAGCTGGAGTGGAGTCTGCTGTTTTAAGAGGATTCCTCATTTTGGGCAAAGAA	*2100*
**PB2 246038 Method E**	*2041*	GAAGGCACAGCTGGAGTGGAGTCTGCTGTTTTAAGAGGATTCCTCATTTTGGGCAAAGAA	*2100*
**PB2 246038 Method K**	*2041*	GAAGGCACAGCTGGAGTGGAGTCTGCTGTTTTAAGAGGATTCCTCATTTTGGGCAAAGAA	*2100*
**PB2 246038 Method N**	*2041*	GAAGGCACAGCTGGAGTGGAGTCTGCTGTTTTAAGAGGATTCCTCATTTTGGGCAAAGAA	*2100*
**PB2 246038 MiSeq**	*2101*	GACAAGAGATATGGCCCAGCACTGAGCATCAATGAGCTGAGCAATTTGGCAAAGGGAGAG	*2160*
**PB2 246038 Method A**	*2101*	GACAAGAGATATGGCCCAGCACTGAGCATCAATGAGCTGAGCAATTTGGCAAAGGGAGAG	*2160*
**PB2 246038 Method S**	*2101*	GACAAGAGATATGGCCCAGCACTGAGCATCAATGAGCTGAGCAATTTGGCAAAGGGAGAG	*2160*
**PB2 246038 Method E**	*2101*	GACAAGAGATATGGCCCAGCACTGAGCATCAATGAGCTGAGCAATTTGGCAAAGGGAGAG	*2160*
**PB2 246038 Method K**	*2101*	GACAAGAGATATGGCCCAGCACTGAGCATCAATGAGCTGAGCAATTTGGCAAAGGGAGAG	*2160*
**PB2 246038 Method N**	*2101*	GACAAGAGATATGGCCCAGCACTGAGCATCAATGAGCTGAGCAATTTGGCAAAGGGAGAG	*2160*
**PB2 246038 MiSeq**	*2161*	AAGGCTAATGTGCTAATTGGGCAAGGAGACGTGGTGTTGGTGATGAAACGGAAACGGGAC	*2220*
**PB2 246038 Method A**	*2161*	AAGGCTAATGTGCTAATTGGGCAAGGAGACGTGGTGTTGGTGATGAAACGGAAACGGGAC	*2220*
**PB2 246038 Method S**	*2161*	AAGGCTAATGTGCTAATTGGGCAAGGAGACGTGGTGTTGGTGATGAAACGGAAACGGGAC	*2220*
**PB2 246038 Method E**	*2161*	AAGGCTAATGTGCTAATTGGGCAAGGAGACGTGGTGTTGGTGATGAAACGGAAACGGGAC	*2220*
**PB2 246038 Method K**	*2161*	AAGGCTAATGTGCTAATTGGGCAAGGAGACGTGGTGTTGGTGATGAAACGGAAACGGGAC	*2220*
**PB2 246038 Method N**	*2161*	AAGGCTAATGTGCTAATTGGGCAAGGAGACGTGGTGTTGGTGATGAAACGGAAACGGGAC	*2220*
**PB2 246038 MiSeq**	*2221*	TCTAGCATACTTACTGACAGCCAGACAGCGACCAAAAGAATTCGGATGGCCATCAATTAG	*2280*
**PB2 246038 Method A**	*2221*	TCTAGCATACTTACTGACAGCCAGACAGCGACCAAAAGAATTCGGATGGCCATCAATTAG	*2280*
**PB2 246038 Method S**	*2221*	TCTAGCATACTTACTGACAGCCAGACAGCGACCAAAAGAATTCGGATGGCCATCAATTAG	*2280*
**PB2 246038 Method E**	*2221*	TCTAGCATACTTACTGACAGCCAGACAGCGACCAAAAGAATTCGGATGGCCATCAATTAG	*2280*
**PB2 246038 Method K**	*2221*	TCTAGCATACTTACTGACAGCCAGACAGCGACCAAAAGAATTCGGATGGCCATCAATTAG	*2280*
**PB2 246038 Method N**	*2221*	TCTAGCATACTTACTGACAGCCAGACAGCGACCAAAAGAATTCGGATGGCCATCAATTAG	*2280*
