# Supplementary material for: An Evaluation of Avian Influenza Virus Whole-Genome Sequencing Approaches Using Nanopore Technology
Source: Microorganisms. 2023 Feb 19;11(2):529. doi: 10.3390/microorganisms11020529 (PMC9967579; doi:10.3390/microorganisms11020529)
Supplement: Supplementary file 1 [file microorganisms-11-00529-s001.zip › manuscript.v8 230219 Suppl Figures and Tables/Supplementary Figures S1a-h 245467/Supplementary Figure S1b PB1.pdf]

Formatted Alignments

**PB1 245467 MiSeq**	*1*	ATGGATGTCAATCCGACTTTACTCTTCTTGAAAGTTCCAGCGCAAAATGCCATAAGCACC	*60*
**PB1 245467 Method A**	*1*	ATGGATGTCAATCCGACTTTACTCTTCTTGAAAGTTCCAGCGCAAAATGCCATAAGCACC	*60*
**PB1 245467 Method S**	*1*	ATGGATGTCAATCCGACTTTACTCTTCTTGAAAGTTCCAGCGCAAAATGCCATAAGCACC	*60*
**PB1 245467 Method E**	*1*	ATGGATGTCAATCCGACTTTACTCTTCTTGAAAGTTCCAGCGCAAAATGCCATAAGCACC	*60*
**PB1 245467 Method K**	*1*	ATGGATGTCAATCCGACTTTACTCTTCTTGAAAGTTCCAGCGCAAAATGCCATAAGCACC	*60*
**PB1 245467 Method N**	*1*	ATGGATGTCAATCCGACTTTACTCTTCTTGAAAGTTCCAGCGCAAAATGCCATAAGCACC	*60*
**PB1 245467 MiSeq**	*61*	ACATTCCCGTATACTGGAGATCCTCCATACAGCCATGGAACAGGAACAGGATATACCATG	*120*
**PB1 245467 Method A**	*61*	ACATTCCCGTATACTGGAGATCCTCCATACAGCCATGGAACAGGAACAGGATATACCATG	*120*
**PB1 245467 Method S**	*61*	ACATTCCCGTATACTGGAGATCCTCCATACAGCCATGGAACAGGAACAGGATATACCATG	*120*
**PB1 245467 Method E**	*61*	ACATTCCCGTATACTGGAGATCCTCCATACAGCCATGGAACAGGAACAGGATATACCATG	*120*
**PB1 245467 Method K**	*61*	ACATTCCCGTATACTGGAGATCCTCCATACAGCCATGGAACAGGAACAGGATATACCATG	*120*
**PB1 245467 Method N**	*61*	ACATTCCCGTATACTGGAGATCCTCCATACAGCCATGGAACAGGAACAGGATATACCATG	*120*
**PB1 245467 MiSeq**	*121*	GACACAGTTAACAGAACACATCAATATTCAGAAAAAGGGAAATGGACAACAAACTCAGAA	*180*
**PB1 245467 Method A**	*121*	GACACAGTTAACAGAACACATCAATATTCAGAAAAAGGGAAATGGACAACAAACTCAGAA	*180*
**PB1 245467 Method S**	*121*	GACAC**G**GTTAACAGAACACATCAATATTCAGAAAAAGGGAAATGGACAACAAACTCAGAA	*180*
**PB1 245467 Method E**	*121*	GACACAGTTAACAGAACACATCAATATTCAGAAAAAGGGAAATGGACAACAAACTCAGAA	*180*
**PB1 245467 Method K**	*121*	GACACAGTTAACAGAACACATCAATATTCAGAAAAAGGGAAATGGACAACAAACTCAGAA	*180*
**PB1 245467 Method N**	*121*	GACACAGTTAACAGAACACATCAATATTCAGAAAAAGGGAAATGGACAACAAACTCAGAA	*180*
**PB1 245467 MiSeq**	*181*	ACTGGAGCACCTCAACTCAATCCAATTGATGGACCTTTGCCTGAGGACAATGAGCCAAGT	*240*
**PB1 245467 Method A**	*181*	ACTGGAGCACCTCAACTCAATCCAATTGATGGACCTTTGCCTGAGGACAATGAGCCAAGT	*240*
**PB1 245467 Method S**	*181*	ACTGGAGCACCTCAACTCAATCCAATTGATGGACC**A**TTGCCTGAGGACAATGAGCCAAGT	*240*
**PB1 245467 Method E**	*181*	ACTGGAGCACCTCAACTCAATCCAATTGATGGACCTTTGCCTGAGGACAATGAGCCAAGT	*240*
**PB1 245467 Method K**	*181*	ACTGGAGCACCTCAACTCAATCCAATTGATGGACCTTTGCCTGAGGACAATGAGCCAAGT	*240*
**PB1 245467 Method N**	*181*	ACTGGAGCACCTCAACTCAATCCAATTGATGGACCTTTGCCTGAGGACAATGAGCCAAGT	*240*

**PB1 245467 MiSeq**	*241*	GGATATGCACAAACGGACTGTGTCCTTGAAGCAATGGCTTTCCTTGAAGAGTCCCACCCA	*300*
**PB1 245467 Method A**	*241*	GGATATGCACAAACGGACTGTGTCCTTGAAGCAATGGCTTTCCTTGAAGAGTCCCACCCA	*300*
**PB1 245467 Method S**	*241*	GGATATGCACAAACGGACTG**C**GTCCTTGAAGCAATGGCTTTCCTTGAAGAGTCCCACCCA	*300*
**PB1 245467 Method E**	*241*	GGATATGCACAAACGGACTGTGTCCTTGAAGCAATGGCTTTCCTTGAAGAGTCCCACCCA	*300*
**PB1 245467 Method K**	*241*	GGATATGCACAAACGGACTGTGTCCTTGAAGCAATGGCTTTCCTTGAAGAGTCCCACCCA	*300*
**PB1 245467 Method N**	*241*	GGATATGCACAAACGGACTGTGTCCTTGAAGCAATGGCTTTCCTTGAAGAGTCCCACCCA	*300*

**PB1 245467 MiSeq**	*301*	GGAATCTTTGAAAACTCGTGTCTTGAAACAATGGAAGTTGTTCAACAAACAAGAGTGGAC	*360*
**PB1 245467 Method A**	*301*	GGAATCTTTGAAAACTCGTGTCTTGAAACAATGGAAGTTGTTCAACAAACAAGAGTGGAC	*360*
**PB1 245467 Method S**	*301*	GGAATCTTTGAAAACTCGTGTCTTGAAAC**G**ATGGAAGTTGTTCAACAAACAAGAGTGGAC	*360*
**PB1 245467 Method E**	*301*	GGAATCTTTGAAAACTCGTGTCTTGAAACAATGGAAGTTGTTCAACAAACAAGAGTGGAC	*360*
**PB1 245467 Method K**	*301*	GGAATCTTTGAAAACTCGTGTCTTGAAACAATGGAAGTTGTTCAACAAACAAGAGTGGAC	*360*
**PB1 245467 Method N**	*301*	GGAATCTTTGAAAACTCGTGTCTTGAAACAATGGAAGTTGTTCAACAAACAAGAGTGGAC	*360*

**PB1 245467 MiSeq**	*361*	AAGTTGACCCAAGGCCGTCAGACTTATGATTGGACATTAAACAGAAATCAGCCGGCTGCA	*420*
**PB1 245467 Method A**	*361*	AAGTTGACCCAAGGCCGTCAGACTTATGATTGGACATTAAACAGAAATCAGCCGGCTGCA	*420*
**PB1 245467 Method S**	*361*	AA**A**TTGACCCAAGGCCGTCAGACTTATGATTGGACATTAAACAGAAATCAGCCGGCTGCA	*420*
**PB1 245467 Method E**	*361*	AAGTTGACCCAAGGCCGTCAGACTTATGATTGGACATTAAACAGAAATCAGCCGGCTGCA	*420*
**PB1 245467 Method K**	*361*	AAGTTGACCCAAGGCCGTCAGACTTATGATTGGACATTAAACAGAAATCAGCCGGCTGCA	*420*
**PB1 245467 Method N**	*361*	AAGTTGACCCAAGGCCGTCAGACTTATGATTGGACATTAAACAGAAATCAGCCGGCTGCA	*420*

**PB1 245467 MiSeq**	*421*	ACTGCATTAGCTAATACTATAGAGGTCTTCAGATCGAACGGTCTTACAGCTAATGAATCA	*480*
**PB1 245467 Method A**	*421*	ACTGCATTAGCTAATACTATAGAGGTCTTCAGATCGAACGGTCTTACAGCTAATGAATCA	*480*
**PB1 245467 Method S**	*421*	ACTGCATTAGCTAATACTATAGAGGTCTTCAGATCGAACGGTCTTACAGCTAATGAATCA	*480*
**PB1 245467 Method E**	*421*	ACTGCATTAGCTAATACTATAGAGGTCTTCAGATCGAACGGTCTTACAGCTAATGAATCA	*480*
**PB1 245467 Method K**	*421*	ACTGCATTAGCTAATACTATAGAGGTCTTCAGATCGAACGGTCTTACAGCTAATGAATCA	*480*
**PB1 245467 Method N**	*421*	ACTGCATTAGCTAATACTATAGAGGTCTTCAGATCGAACGGTCTTACAGCTAATGAATCA	*480*

**PB1 245467 MiSeq**	*481*	GGAAGGCTAATTGATTTTCTCAAGGATGTGATGGAATCAATGGATAAAGAGGAAATAGAA	*540*
**PB1 245467 Method A**	*481*	GGAAGGCTAATTGATTTTCTCAAGGATGTGATGGAATCAATGGATAAAGAGGAAATAGAA	*540*
**PB1 245467 Method S**	*481*	GGAAGGCTAAT**A**GATTTTCTCAAGGATGTGATGGAATCAATGGATAAAGAGGAAATAGAA	*540*
**PB1 245467 Method E**	*481*	GGAAGGCTAATTGATTTTCTCAAGGATGTGATGGAATCAATGGATAAAGAGGAAATAGAA	*540*
**PB1 245467 Method K**	*481*	GGAAGGCTAATTGATTTTCTCAAGGATGTGATGGAATCAATGGATAAAGAGGAAATAGAA	*540*
**PB1 245467 Method N**	*481*	GGAAGGCTAATTGATTTTCTCAAGGATGTGATGGAATCAATGGATAAAGAGGAAATAGAA	*540*

**PB1 245467 MiSeq**	*541*	ATAACAACACATTTCCAAAGGAAAAGAAGAGTGAGAGACAACATGACCAAGAAAATGGTC	*600*
**PB1 245467 Method A**	*541*	ATAACAACACATTTCCAAAGGAAAAGAAGAGTGAGAGACAACATGACCAAGAAAATGGTC	*600*
**PB1 245467 Method S**	*541*	ATAACAACGCATTTCCAAAGGAAAAGAAGAGTGAGAGACAACATGACCAAGAAAATGGTC	*600*
**PB1 245467 Method E**	*541*	ATAACAACACATTTCCAAAGGAAAAGAAGAGTGAGAGACAACATGACCAAGAAAATGGTC	*600*
**PB1 245467 Method K**	*541*	ATAACAACACATTTCCAAAGGAAAAGAAGAGTGAGAGACAACATGACCAAGAAAATGGTC	*600*
**PB1 245467 Method N**	*541*	ATAACAACACATTTCCAAAGGAAAAGAAGAGTGAGAGACAACATGACCAAGAAAATGGTC	*600*

**PB1 245467 MiSeq**	*601*	ACACAAAGGACAATAGGAAAGAAGAAACAAAGATTAAACAAAAGGAGCTATCTGATAAGA	*660*
**PB1 245467 Method A**	*601*	ACACAAAGGACAATAGGAAAGAAGAAACAAAGATTAAACAAAAGGAGCTATCTGATAAGA	*660*
**PB1 245467 Method S**	*601*	ACACAAAGGACAATAGGAAAGAAGAAACAAAGGTTAAACAAAAGGAGCTATCTGATAAGA	*660*
**PB1 245467 Method E**	*601*	ACACAAAGGACAATAGGAAAGAAGAAACAAAGATTAAACAAAAGGAGCTATCTGATAAGA	*660*
**PB1 245467 Method K**	*601*	ACACAAAGGACAATAGGAAAGAAGAAACAAAGATTAAACAAAAGGAGCTATCTGATAAGA	*660*
**PB1 245467 Method N**	*601*	ACACAAAGGACAATAGGAAAGAAGAAACAAAGATTAAACAAAAGGAGCTATCTGATAAGA	*660*

**PB1 245467 MiSeq**	*661*	GCATTGACATTGAACACAATGACAAAAGACGCCGAAAGAGGCAAATTAAAGAGAAGGGCA	*720*
**PB1 245467 Method A**	*661*	GCATTGACATTGAACACAATGACAAAAGACGCCGAAAGAGGCAAATTAAAGAGAAGGGCA	*720*
**PB1 245467 Method S**	*661*	GCATTGACACTGAACACAATGACAAAAGACGCCGAAAGAGGCAAATTAAAGAGAAGGGCA	*720*
**PB1 245467 Method E**	*661*	GCATTGACATTGAACACAATGACAAAAGACGCCGAAAGAGGCAAATTAAAGAGAAGGGCA	*720*
**PB1 245467 Method K**	*661*	GCATTGACATTGAACACAATGACAAAAGACGCCGAAAGAGGCAAATTAAAGAGAAGGGCA	*720*
**PB1 245467 Method N**	*661*	GCATTGACATTGAACACAATGACAAAAGACGCCGAAAGAGGCAAATTAAAGAGAAGGGCA	*720*

**PB1 245467 MiSeq**	*721*	ATTGCAACACCCGGGATGCAAATCAGAGGGTTTGTGTACTTTGTTGAAACATTAGCAAGG	*780*
**PB1 245467 Method A**	*721*	ATTGCAACACCCGGGATGCAAATCAGAGGGTTTGTGTACTTTGTTGAAACATTAGCAAGG	*780*
**PB1 245467 Method S**	*721*	ATTGCAACACCCGGGATGCAAATCAGAGGGTTTGTGTACTTTGTTGAAACATTAGCAAGG	*780*
**PB1 245467 Method E**	*721*	ATTGCAACACCCGGGATGCAAATCAGAGGGTTTGTGTACTTTGTTGAAACATTAGCAAGG	*780*
**PB1 245467 Method K**	*721*	ATTGCAACACCCGGGATGCAAATCAGAGGGTTTGTGTACTTTGTTGAAACATTAGCAAGG	*780*
**PB1 245467 Method N**	*721*	ATTGCAACACCCGGGATGCAAATCAGAGGGTTTGTGTACTTTGTTGAAACATTAGCAAGG	*780*

**PB1 245467 MiSeq**	*781*	GGCATTTGTGAGAAACTCGAACAATCTGGACTCCCAGTTGGAGGCAATGAAAAAAAGGCT	*840*
**PB1 245467 Method A**	*781*	GGCATTTGTGAGAAACTCGAACAATCTGGACTCCCAGTTGGAGGCAATGAAAAAAAGGCT	*840*
**PB1 245467 Method S**	*781*	AGCATTTGTGAGAAACTTGAACAATCTGGACTCCCAGTTGGAGGCAATGAAAAGAAGGCT	*840*
**PB1 245467 Method E**	*781*	GGCATTTGTGAGAAACTCGAACAATCTGGACTCCCAGTTGGAGGCAATGAAAAAAAGGCT	*840*
**PB1 245467 Method K**	*781*	GGCATTTGTGAGAAACTCGAACAATCTGGACTCCCAGTTGGAGGCAATGAAAAAAAGGCT	*840*
**PB1 245467 Method N**	*781*	GGCATTTGTGAGAAACTCGAACAATCTGGACTCCCAGTTGGAGGCAATGAAAAAAAGGCT	*840*

**PB1 245467 MiSeq**	*841*	AAACTAGCAAATGTCGTGAGAAAGATGATGACTAATTCGCAAGACACAGAGCTCTCTTTC	*900*
**PB1 245467 Method A**	*841*	AAACTAGCAAATGTCGTGAGAAAGATGATGACTAATTCGCAAGACACAGAGCTCTCTTTC	*900*
**PB1 245467 Method S**	*841*	AAACTAGCAAATGTTGTGAGAAAGATGATGACTAATTCGCAAGACACAGAGCTCTCTTTC	*900*
**PB1 245467 Method E**	*841*	AAACTAGCAAATGTCGTGAGAAAGATGATGACTAATTCGCAAGACACAGAGCTCTCTTTC	*900*
**PB1 245467 Method K**	*841*	AAACTAGCAAATGTCGTGAGAAAGATGATGACTAATTCGCAAGACACAGAGCTCTCTTTC	*900*
**PB1 245467 Method N**	*841*	AAACTAGCAAATGTCGTGAGAAAGATGATGACTAATTCGCAAGACACAGAGCTCTCTTTC	*900*
**PB1 245467 MiSeq**	*901*	ACAATCACGGGAGACAACACCAAATGGAATGAGAACCAGAATCCTAGGATGTTTCTGGCA	*960*
**PB1 245467 Method A**	*901*	ACAATCACGGGAGACAACACCAAATGGAATGAGAACCAGAATCCTAGGATGTTTCTGGCA	*960*
**PB1 245467 Method S**	*901*	ACAATCACGGGAGACAACACCAAATGGAATGAAAATCAGAATCCTAGGATGTTTCTGGCA	*960*
**PB1 245467 Method E**	*901*	ACAATCACGGGAGACAACACCAAATGGAATGAGAACCAGAATCCTAGGATGTTTCTGGCA	*960*
**PB1 245467 Method K**	*901*	ACAATCACGGGAGACAACACCAAATGGAATGAGAACCAGAATCCTAGGATGTTTCTGGCA	*960*
**PB1 245467 Method N**	*901*	ACAATCACGGGAGACAACACCAAATGGAATGAGAACCAGAATCCTAGGATGTTTCTGGCA	*960*
**PB1 245467 MiSeq**	*961*	ATGATAACATATATAACAAGGAACCAACCTGAATGGTTCAGGAATGTCTTGAGCATTGCA	*1020*
**PB1 245467 Method A**	*961*	ATGATAACATATATAACAAGGAACCAACCTGAATGGTTCAGGAATGTCTTGAGCATTGCA	*1020*
**PB1 245467 Method S**	*961*	ATGATAACATATATAACAAGGAACCAACCTGAATGGTTCAGGAATGTCTTGAGCATTGCA	*1020*
**PB1 245467 Method E**	*961*	ATGATAACATATATAACAAGGAACCAACCTGAATGGTTCAGGAATGTCTTGAGCATTGCA	*1020*
**PB1 245467 Method K**	*961*	ATGATAACATATATAACAAGGAACCAACCTGAATGGTTCAGGAATGTCTTGAGCATTGCA	*1020*
**PB1 245467 Method N**	*961*	ATGATAACATATATAACAAGGAACCAACCTGAATGGTTCAGGAATGTCTTGAGCATTGCA	*1020*
**PB1 245467 MiSeq**	*1021*	CCTATAATGTTCTCAAATAAAATGGCAAGACTAGGGAAAGGATACATGTTCGAAAGTAAG	*1080*
**PB1 245467 Method A**	*1021*	CCTATAATGTTCTCAAATAAAATGGCAAGACTAGGGAAAGGATACATGTTCGAAAGTAAG	*1080*
**PB1 245467 Method S**	*1021*	CCTATAATGTTCTCAAATAAAATGGCAAGACTAGGGAAAGGATACATGTTCGAAAGTAAG	*1080*
**PB1 245467 Method E**	*1021*	CCTATAATGTTCTCAAATAAAATGGCAAGACTAGGGAAAGGATACATGTTCGAAAGTAAG	*1080*
**PB1 245467 Method K**	*1021*	CCTATAATGTTCTCAAATAAAATGGCAAGACTAGGGAAAGGATACATGTTCGAAAGTAAG	*1080*
**PB1 245467 Method N**	*1021*	CCTATAATGTTCTCAAATAAAATGGCAAGACTAGGGAAAGGATACATGTTCGAAAGTAAG	*1080*
**PB1 245467 MiSeq**	*1081*	AGCATGAAGCTTCGAACACAAATACCGGCAGAAATGCTAGCAAGCATCGATCTGAAGTAC	*1140*
**PB1 245467 Method A**	*1081*	AGCATGAAGCTTCGAACACAAATACCGGCAGAAATGCTAGCAAGCATCGATCTGAAGTAC	*1140*
**PB1 245467 Method S**	*1081*	AGCATGAAGCTTCGAACACAAATACCGGCAGAAATGCTAGCAAGCATTGATCTGAAGTAT	*1140*
**PB1 245467 Method E**	*1081*	AGCATGAAGCTTCGAACACAAATACCGGCAGAAATGCTAGCAAGCATCGATCTGAAGTAC	*1140*
**PB1 245467 Method K**	*1081*	AGCATGAAGCTTCGAACACAAATACCGGCAGAAATGCTAGCAAGCATCGATCTGAAGTAC	*1140*
**PB1 245467 Method N**	*1081*	AGCATGAAGCTTCGAACACAAATACCGGCAGAAATGCTAGCAAGCATCGATCTGAAGTAC	*1140*

**PB1 245467 MiSeq**	*1141*	TTCAATGAGTCAACAAAAAAGAAAATAGAGAAGACAAGACCTCTTCTAATAGATGGCACG	*1200*
**PB1 245467 Method A**	*1141*	TTCAATGAGTCAACAAAAAAGAAAATAGAGAAGACAAGACCTCTTCTAATAGATGGCACG	*1200*
**PB1 245467 Method S**	*1141*	TTCAATGAGTCAACAAGAAAGAAAATAGAGAAGATAAGACCTCTTCTAATAGATGGTACG	*1200*
**PB1 245467 Method E**	*1141*	TTCAATGAGTCAACAAAAAAGAAAATAGAGAAGACAAGACCTCTTCTAATAGATGGCACG	*1200*
**PB1 245467 Method K**	*1141*	TTCAATGAGTCAACAAAAAAGAAAATAGAGAAGACAAGACCTCTTCTAATAGATGGCACG	*1200*
**PB1 245467 Method N**	*1141*	TTCAATGAGTCAACAAAAAAGAAAATAGAGAAGACAAGACCTCTTCTAATAGATGGCACG	*1200*

**PB1 245467 MiSeq**	*1201*	GCCTCATTAAGCCCTGGAATGATGATGGGCATGTTCAACATGCTGAGTACAGTTTTGGGA	*1260*
**PB1 245467 Method A**	*1201*	GCCTCATTAAGCCCTGGAATGATGATGGGCATGTTCAACATGCTGAGTACAGTTTTGGGA	*1260*
**PB1 245467 Method S**	*1201*	GCCTCATTAAGCCCTGGAATGATGATGGGCATGTTCAACATGCTGAGTACAGTTCTGGGA	*1260*
**PB1 245467 Method E**	*1201*	GCCTCATTAAGCCCTGGAATGATGATGGGCATGTTCAACATGCTGAGTACAGTTTTGGGA	*1260*
**PB1 245467 Method K**	*1201*	GCCTCATTAAGCCCTGGAATGATGATGGGCATGTTCAACATGCTGAGTACAGTTTTGGGA	*1260*
**PB1 245467 Method N**	*1201*	GCCTCATTAAGCCCTGGAATGATGATGGGCATGTTCAACATGCTGAGTACAGTTTTGGGA	*1260*

**PB1 245467 MiSeq**	*1261*	GTTTCGATTTTGAATCTAGGGCAAAAGAGGCACACCAAAACAACATACTGGTGGGATGGA	*1320*
**PB1 245467 Method A**	*1261*	GTTTCGATTTTGAATCTAGGGCAAAAGAGGCACACCAAAACAACATACTGGTGGGATGGA	*1320*
**PB1 245467 Method S**	*1261*	GTTTCGATTCTAAATCTAGGGCAAAAGAGGTACACCAAAACAACATACTGGTGGGATGGA	*1320*
**PB1 245467 Method E**	*1261*	GTTTCGATTTTGAATCTAGGGCAAAAGAGGCACACCAAAACAACATACTGGTGGGATGGA	*1320*
**PB1 245467 Method K**	*1261*	GTTTCGATTTTGAATCTAGGGCAAAAGAGGCACACCAAAACAACATACTGGTGGGATGGA	*1320*
**PB1 245467 Method N**	*1261*	GTTTCGATTTTGAATCTAGGGCAAAAGAGGCACACCAAAACAACATACTGGTGGGATGGA	*1320*

**PB1 245467 MiSeq**	*1321*	CTACAATCCTCTGATGACTTTGCTCTCATAGTGAATGCTCCGAATCATGAGGGAATACAA	*1380*
**PB1 245467 Method A**	*1321*	CTACAATCCTCTGATGACTTTGCTCTCATAGTGAATGCTCCGAATCATGAGGGAATACAA	*1380*
**PB1 245467 Method S**	*1321*	CTACAATCCTCTGATGACTTTGCTCTCATAGTGAATGCTCCGAATCATGAGGGAATACAA	*1380*
**PB1 245467 Method E**	*1321*	CTACAATCCTCTGATGACTTTGCTCTCATAGTGAATGCTCCGAATCATGAGGGAATACAA	*1380*
**PB1 245467 Method K**	*1321*	CTACAATCCTCTGATGACTTTGCTCTCATAGTGAATGCTCCGAATCATGAGGGAATACAA	*1380*
**PB1 245467 Method N**	*1321*	CTACAATCCTCTGATGACTTTGCTCTCATAGTGAATGCTCCGAATCATGAGGGAATACAA	*1380*

**PB1 245467 MiSeq**	*1381*	GCAGGGGTAGACAGATTCTATAGAACCTGCAAGCTGGTCGGAATCAACATGAGCAAAAAG	*1440*
**PB1 245467 Method A**	*1381*	GCAGGGGTAGACAGATTCTATAGAACCTGCAAGCTGGTCGGAATCAACATGAGCAAAAAG	*1440*
**PB1 245467 Method S**	*1381*	GCAGGAGTAGACAGATTCTATAGAACCTGCAAGCTGGTCGGAATCAACATGAGCAAAAAG	*1440*
**PB1 245467 Method E**	*1381*	GCAGGGGTAGACAGATTCTATAGAACCTGCAAGCTGGTCGGAATCAACATGAGCAAAAAG	*1440*
**PB1 245467 Method K**	*1381*	GCAGGGGTAGACAGATTCTATAGAACCTGCAAGCTGGTCGGAATCAACATGAGCAAAAAG	*1440*
**PB1 245467 Method N**	*1381*	GCAGGGGTAGACAGATTCTATAGAACCTGCAAGCTGGTCGGAATCAACATGAGCAAAAAG	*1440*

**PB1 245467 MiSeq**	*1441*	AAGTCCTACATAAACAGGACAGGAACATTTGAATTCACAAGTTTTTTCTACCGCTATGGA	*1500*
**PB1 245467 Method A**	*1441*	AAGTCCTACATAAACAGGACAGGAACATTTGAATTCACAAGTTTTTTCTACCGCTATGGA	*1500*
**PB1 245467 Method S**	*1441*	AAGTCCTACATAAACAGGACAGGAACATTTGAATTCACAAGTTTTTTCTACCGCTATGGA	*1500*
**PB1 245467 Method E**	*1441*	AAGTCCTACATAAACAGGACAGGAACATTTGAATTCACAAGTTTTTTCTACCGCTATGGA	*1500*
**PB1 245467 Method K**	*1441*	AAGTCCTACATAAACAGGACAGGAACATTTGAATTCACAAGTTTTTTCTACCGCTATGGA	*1500*
**PB1 245467 Method N**	*1441*	AAGTCCTACATAAACAGGACAGGAACATTTGAATTCACAAGTTTTTTCTACCGCTATGGA	*1500*
**PB1 245467 MiSeq**	*1501*	TTTGTAGCCAACTTCAGCATGGAATTGCCCAGCTTTGGAGTGTCTGGGATCAATGAATCT	*1560*
**PB1 245467 Method A**	*1501*	TTTGTAGCCAACTTCAGCATGGAATTGCCCAGCTTTGGAGTGTCTGGGATCAATGAATCT	*1560*
**PB1 245467 Method S**	*1501*	TTTGTAGCCAACTTCAGCATGGAGTTGCCCAGCTTTGGAGTGTCTGGGATCAATGAATCT	*1560*
**PB1 245467 Method E**	*1501*	TTTGTAGCCAACTTCAGCATGGAATTGCCCAGCTTTGGAGTGTCTGGGATCAATGAATCT	*1560*
**PB1 245467 Method K**	*1501*	TTTGTAGCCAACTTCAGCATGGAATTGCCCAGCTTTGGAGTGTCTGGGATCAATGAATCT	*1560*
**PB1 245467 Method N**	*1501*	TTTGTAGCCAACTTCAGCATGGAATTGCCCAGCTTTGGAGTGTCTGGGATCAATGAATCT	*1560*
**PB1 245467 MiSeq**	*1561*	GCGGACATGAGTATTGGAGTAACAGTGATAAAGAACAACATGATCAACAATGATCTTGGA	*1620*
**PB1 245467 Method A**	*1561*	GCGGACATGAGTATTGGAGTAACAGTGATAAAGAACAACATGATCAACAATGATCTTGGA	*1620*
**PB1 245467 Method S**	*1561*	GCAGACATGAGCATTGGAGTAACAGTGATAAAGAACAACATGATCAACAATGATCTTGGA	*1620*
**PB1 245467 Method E**	*1561*	GCGGACATGAGTATTGGAGTAACAGTGATAAAGAACAACATGATCAACAATGATCTTGGA	*1620*
**PB1 245467 Method K**	*1561*	GCGGACATGAGTATTGGAGTAACAGTGATAAAGAACAACATGATCAACAATGATCTTGGA	*1620*
**PB1 245467 Method N**	*1561*	GCGGACATGAGTATTGGAGTAACAGTGATAAAGAACAACATGATCAACAATGATCTTGGA	*1620*
**PB1 245467 MiSeq**	*1621*	CCAGCAACAGCCCAAATGGCTCTACAGCTATTCATCAAGGATTACAGATACACATATCGA	*1680*
**PB1 245467 Method A**	*1621*	CCAGCAACAGCCCAAATGGCTCTACAGCTATTCATCAAGGATTACAGATACACATATCGA	*1680*
**PB1 245467 Method S**	*1621*	CCAGCAACAGCCCAAATGGCTCTACAGCTATTCATCAAGGATTACAGATACACATATCGA	*1680*
**PB1 245467 Method E**	*1621*	CCAGCAACAGCCCAAATGGCTCTACAGCTATTCATCAAGGATTACAGATACACATATCGA	*1680*
**PB1 245467 Method K**	*1621*	CCAGCAACAGCCCAAATGGCTCTACAGCTATTCATCAAGGATTACAGATACACATATCGA	*1680*
**PB1 245467 Method N**	*1621*	CCAGCAACAGCCCAAATGGCTCTACAGCTATTCATCAAGGATTACAGATACACATATCGA	*1680*
**PB1 245467 MiSeq**	*1681*	TGTCACAGAGGAGACACACAAATTCAAACAAGGAGGTCATTTGAGCTGAAGAAGTTATGG	*1740*
**PB1 245467 Method A**	*1681*	TGTCACAGAGGAGACACACAAATTCAAACAAGGAGGTCATTTGAGCTGAAGAAGTTATGG	*1740*
**PB1 245467 Method S**	*1681*	TGTCACAGAGGAGACACACAAATTCAAACAAGGAGGTCATTCGAGCTGAAGAAGTTATGG	*1740*
**PB1 245467 Method E**	*1681*	TGTCACAGAGGAGACACACAAATTCAAACAAGGAGGTCATTTGAGCTGAAGAAGTTATGG	*1740*
**PB1 245467 Method K**	*1681*	TGTCACAGAGGAGACACACAAATTCAAACAAGGAGGTCATTTGAGCTGAAGAAGTTATGG	*1740*
**PB1 245467 Method N**	*1681*	TGTCACAGAGGAGACACACAAATTCAAACAAGGAGGTCATTTGAGCTGAAGAAGTTATGG	*1740*

**PB1 245467 MiSeq**	*1741*	GAACAAACCCGCTCAAAAGCAGGACTGCTGGTCTCAGATGGAGGACCAAATCTATACAAT	*1800*
**PB1 245467 Method A**	*1741*	GAACAAACCCGCTCAAAAGCAGGACTGCTGGTCTCAGATGGAGGACCAAATCTATACAAT	*1800*
**PB1 245467 Method S**	*1741*	GAACAAACCCGCTCAAAAGCAGGACTGCTGGTCTCAGATGGAGGACCAAATCTATACAAT	*1800*
**PB1 245467 Method E**	*1741*	GAACAAACCCGCTCAAAAGCAGGACTGCTGGTCTCAGATGGAGGACCAAATCTATACAAT	*1800*
**PB1 245467 Method K**	*1741*	GAACAAACCCGCTCAAAAGCAGGACTGCTGGTCTCAGATGGAGGACCAAATCTATACAAT	*1800*
**PB1 245467 Method N**	*1741*	GAACAAACCCGCTCAAAAGCAGGACTGCTGGTCTCAGATGGAGGACCAAATCTATACAAT	*1800*
**PB1 245467 MiSeq**	*1801*	ATCCGGAATCTCCACATTCCGGAAGTCTGTTTAAAATGGGAACTAATGGATGAAGACTAT	*1860*
**PB1 245467 Method A**	*1801*	ATCCGGAATCTCCACATTCCGGAAGTCTGTTTAAAATGGGAACTAATGGATGAAGACTAT	*1860*
**PB1 245467 Method S**	*1801*	ATCCGGAATCTCCACATTCCGGAAGTCTGCTTAAAATGGGAGCTAATGGACGAAGACTAT	*1860*
**PB1 245467 Method E**	*1801*	ATCCGGAATCTCCACATTCCGGAAGTCTGTTTAAAATGGGAACTAATGGATGAAGACTAT	*1860*
**PB1 245467 Method K**	*1801*	ATCCGGAATCTCCACATTCCGGAAGTCTGTTTAAAATGGGAACTAATGGATGAAGACTAT	*1860*
**PB1 245467 Method N**	*1801*	ATCCGGAATCTCCACATTCCGGAAGTCTGTTTAAAATGGGAACTAATGGATGAAGACTAT	*1860*
**PB1 245467 MiSeq**	*1861*	CAGGGAAGGCTTTGTAATCCTCTGAATCCGTTTGTCAGCCACAAAGAAATAGAGTCTGTG	*1920*
**PB1 245467 Method A**	*1861*	CAGGGAAGGCTTTGTAATCCTCTGAATCCGTTTGTCAGCCACAAAGAAATAGAGTCTGTG	*1920*
**PB1 245467 Method S**	*1861*	CAGGGAAGGCTTTGTAATCCCCTGAATCCGTTTGTCAGCCACAAAGAAATAGAGTCTGTG	*1920*
**PB1 245467 Method E**	*1861*	CAGGGAAGGCTTTGTAATCCTCTGAATCCGTTTGTCAGCCACAAAGAAATAGAGTCTGTG	*1920*
**PB1 245467 Method K**	*1861*	CAGGGAAGGCTTTGTAATCCTCTGAATCCGTTTGTCAGCCACAAAGAAATAGAGTCTGTG	*1920*
**PB1 245467 Method N**	*1861*	CAGGGAAGGCTTTGTAATCCTCTGAATCCGTTTGTCAGCCACAAAGAAATAGAGTCTGTG	*1920*
**PB1 245467 MiSeq**	*1921*	AACAATGCTGTGGTGATGCCAGCGCATGGCCCAGCCAAGAGTATGGAATATGATGCTGTT	*1980*
**PB1 245467 Method A**	*1921*	AACAATGCTGTGGTGATGCCAGCGCATGGCCCAGCCAAGAGTATGGAATATGATGCTGTT	*1980*
**PB1 245467 Method S**	*1921*	AACAATGCTGTGGTGATGCCAGCTCATGGCCCAGCCAAGAGTATGGAATATGATGCTGTT	*1980*
**PB1 245467 Method E**	*1921*	AACAATGCTGTGGTGATGCCAGCGCATGGCCCAGCCAAGAGTATGGAATATGATGCTGTT	*1980*
**PB1 245467 Method K**	*1921*	AACAATGCTGTGGTGATGCCAGCGCATGGCCCAGCCAAGAGTATGGAATATGATGCTGTT	*1980*
**PB1 245467 Method N**	*1921*	AACAATGCTGTGGTGATGCCAGCGCATGGCCCAGCCAAGAGTATGGAATATGATGCTGTT	*1980*
**PB1 245467 MiSeq**	*1981*	GCCACCACTCATTCCTGGATTCCTAAGAGGAACCGCTCCATTCTCAATACAAGCCAAAGG	*2040*
**PB1 245467 Method A**	*1981*	GCCACCACTCATTCCTGGATTCCTAAGAGGAACCGCTCCATTCTCAATACAAGCCAAAGG	*2040*
**PB1 245467 Method S**	*1981*	GCCACCACTCACTCCTGGATCCCTAAGAGGAACCGCTCCATTCTCAATACAAGCCAAAGG	*2040*
**PB1 245467 Method E**	*1981*	GCCACCACTCATTCCTGGATTCCTAAGAGGAACCGCTCCATTCTCAATACAAGCCAAAGG	*2040*
**PB1 245467 Method K**	*1981*	GCCACCACTCATTCCTGGATTCCTAAGAGGAACCGCTCCATTCTCAATACAAGCCAAAGG	*2040*
**PB1 245467 Method N**	*1981*	GCCACCACTCATTCCTGGATTCCTAAGAGGAACCGCTCCATTCTCAATACAAGCCAAAGG	*2040*

**PB1 245467 MiSeq**	*2041*	GGAATCCTTGAAGACGAACAGATGTATCAAAAGTGCTGCAATCTGTTTGAAAAATTCTTC	*2100*
**PB1 245467 Method A**	*2041*	GGAATCCTTGAAGACGAACAGATGTATCAAAAGTGCTGCAATCTGTTTGAAAAATTCTTC	*2100*
**PB1 245467 Method S**	*2041*	GGAATCCTTGAAGACGAACAGATGTATCAAAAGTGCTGCAATCTATTTGAAAAATTCTTC	*2100*
**PB1 245467 Method E**	*2041*	GGAATCCTTGAAGACGAACAGATGTATCAAAAGTGCTGCAATCTGTTTGAAAAATTCTTC	*2100*
**PB1 245467 Method K**	*2041*	GGAATCCTTGAAGACGAACAGATGTATCAAAAGTGCTGCAATCTGTTTGAAAAATTCTTC	*2100*
**PB1 245467 Method N**	*2041*	GGAATCCTTGAAGACGAACAGATGTATCAAAAGTGCTGCAATCTGTTTGAAAAATTCTTC	*2100*
**PB1 245467 MiSeq**	*2101*	CCTAGCAGTTCATACAGGAGGCCGGTTGGAATTTCCAGCATGGTGGAGGCCATGGTTTCT	*2160*
**PB1 245467 Method A**	*2101*	CCTAGCAGTTCATACAGGAGGCCGGTTGGAATTTCCAGCATGGTGGAGGCCATGGTTTCT	*2160*
**PB1 245467 Method S**	*2101*	CCTAGCAGTTCATACAGGAGGCCGGTTGGAATTTCCAGCATGGTGGAGGCCATGGTTTCT	*2160*
**PB1 245467 Method E**	*2101*	CCTAGCAGTTCATACAGGAGGCCGGTTGGAATTTCCAGCATGGTGGAGGCCATGGTTTCT	*2160*
**PB1 245467 Method K**	*2101*	CCTAGCAGTTCATACAGGAGGCCGGTTGGAATTTCCAGCATGGTGGAGGCCATGGTTTCT	*2160*
**PB1 245467 Method N**	*2101*	CCTAGCAGTTCATACAGGAGGCCGGTTGGAATTTCCAGCATGGTGGAGGCCATGGTTTCT	*2160*
**PB1 245467 MiSeq**	*2161*	AGGGCCCGAATTGATGCGCGAATTGACTTCGAGTCTGGACGGATTAAGAAGGAGGAGTTT	*2220*
**PB1 245467 Method A**	*2161*	AGGGCCCGAATTGATGCGCGAATTGACTTCGAGTCTGGACGGATTAAGAAGGAGGAGTTT	*2220*
**PB1 245467 Method S**	*2161*	AGGGCCCGAATTGATGCGCGAATTGACTTCGAATCTGGACGGATTAAGAAGGAGGAGTTT	*2220*
**PB1 245467 Method E**	*2161*	AGGGCCCGAATTGATGCGCGAATTGACTTCGAGTCTGGACGGATTAAGAAGGAGGAGTTT	*2220*
**PB1 245467 Method K**	*2161*	AGGGCCCGAATTGATGCGCGAATTGACTTCGAGTCTGGACGGATTAAGAAGGAGGAGTTT	*2220*
**PB1 245467 Method N**	*2161*	AGGGCCCGAATTGATGCGCGAATTGACTTCGAGTCTGGACGGATTAAGAAGGAGGAGTTT	*2220*
**PB1 245467 MiSeq**	*2221*	GCTGAGATCATGAAGACCTGTTCCACCATTGAAGAGCTCAGACGGCAGAAATAG	*2274*
**PB1 245467 Method A**	*2221*	GCTGAGATCATGAAGACCTGTTCCACCATTGAAGAGCTCAGACGGCAGAAATAG	*2274*
**PB1 245467 Method S**	*2221*	GCTGAGATCATGAAGATCTGTTCCACCATTGAAGAGCTCAGACGGCAGAAATAG	*2274*
**PB1 245467 Method E**	*2221*	GCTGAGATCATGAAGACCTGTTCCACCATTGAAGAGCTCAGACGGCAGAAATAG	*2274*
**PB1 245467 Method K**	*2221*	GCTGAGATCATGAAGACCTGTTCCACCATTGAAGAGCTCAGACGGCAGAAATAG	*2274*
**PB1 245467 Method N**	*2221*	GCTGAGATCATGAAGACCTGTTCCACCATTGAAGAGCTCAGACGGCAGAAATAG	*2274*
