# Supplementary material for: An Evaluation of Avian Influenza Virus Whole-Genome Sequencing Approaches Using Nanopore Technology
Source: Microorganisms. 2023 Feb 19;11(2):529. doi: 10.3390/microorganisms11020529 (PMC9967579; doi:10.3390/microorganisms11020529)
Supplement: Supplementary file 1 [file microorganisms-11-00529-s001.zip › manuscript.v8 230219 Suppl Figures and Tables/Supplementary Figures S1a-h 245467/Supplementary Figure S1c PA.pdf]

# Formatted Alignments

| | | | |
|---|---|---|---|
| **PA 245467 MiSeq** | *1* | ATGGAAGACTTTATGCGACAATGCTTCAATCCAATGATTGTCGAGCTTGCGGAAAAAGCA | *60* |
| **PA 245467 Method A** | *1* | ATGGAAGACTTTATGCGACAATGCTTCAATCCAATGATTGTCGAGCTTGCGGAAAAAGCA | *60* |
| **PA 245467 Method S** | *1* | ATGGAAGACTTTGTGCGACAATGCTTCAATCCAATGATTGTCGAGCTTGCGGAAAAAGCA | *60* |
| **PA 245467 Method E** | *1* | ATGGAAGACTTTATGCGACAATGCTTCAATCCAATGATTGTCGAGCTTGCGGAAAAAGCA | *60* |
| **PA 245467 Method K** | *1* | ATGGAAGACTTTATGCGACAATGCTTCAATCCAATGATTGTCGAGCTTGCGGAAAAAGCA | *60* |
| **PA 245467 Method N** | *1* | ATGGAAGACTTTATGCGACAATGCTTCAATCCAATGATTGTCGAGCTTGCGGAAAAAGCA | *60* |
| **PA 245467 MiSeq** | *61* | ATGAAAGAATATGGGGAAGATCCGAAAATCGAGACAAACAAATTTGCCGCAATATGCACA | *120* |
| **PA 245467 Method A** | *61* | ATGAAAGAATATGGGGAAGATCCGAAAATCGAGACAAACAAATTTGCCGCAATATGCACA | *120* |
| **PA 245467 Method S** | *61* | ATGAAAGAATATGGGGAAGATCCGAAAATCGAGACAAACAAATTTGCCGCAATATGCACA | *120* |
| **PA 245467 Method E** | *61* | ATGAAAGAATATGGGGAAGATCCGAAAATCGAGACAAACAAATTTGCCGCAATATGCACA | *120* |
| **PA 245467 Method K** | *61* | ATGAAAGAATATGGGGAAGATCCGAAAATCGAGACAAACAAATTTGCCGCAATATGCACA | *120* |
| **PA 245467 Method N** | *61* | ATGAAAGAATATGGGGAAGATCCGAAAATCGAGACAAACAAATTTGCCGCAATATGCACA | *120* |
| **PA 245467 MiSeq** | *121* | CACTTAGAAGTCTGTTTCATGTATTCGGATTTCCATTTTATTGATGAACGAGGCGAATCA | *180* |
| **PA 245467 Method A** | *121* | CACTTAGAAGTCTGTTTCATGTATTCGGATTTCCATTTTATTGATGAACGAGGCGAATCA | *180* |
| **PA 245467 Method S** | *121* | CACTTAGAAGTCTGTTTCATGTATTCGGATTTCCATTTTATTGATGAACGAGGCGAATCA | *180* |
| **PA 245467 Method E** | *121* | CACTTAGAAGTCTGTTTCATGTATTCGGATTTCCATTTTATTGATGAACGAGGCGAATCA | *180* |
| **PA 245467 Method K** | *121* | CACTTAGAAGTCTGTTTCATGTATTCGGATTTCCATTTTATTGATGAACGAGGCGAATCA | *180* |
| **PA 245467 Method N** | *121* | CACTTAGAAGTCTGTTTCATGTATTCGGATTTCCATTTTATTGATGAACGAGGCGAATCA | *180* |
| **PA 245467 MiSeq** | *181* | ATGATTGTAGAATCTGGCGATCCAAATGCATTATTGAAACACCGATTTGAGATAATCGAA | *240* |
| **PA 245467 Method A** | *181* | ATGATTGTAGAATCTGGCGATCCAAATGCATTATTGAAACACCGATTTGAGATAATCGAA | *240* |
| **PA 245467 Method S** | *181* | ATGATTGTAGAATCTGGCGATCCAAATGCATTATTGAAACACCGATTTGAGATAATCGAA | *240* |
| **PA 245467 Method E** | *181* | ATGATTGTAGAATCTGGCGATCCAAATGCATTATTGAAACACCGATTTGAGATAATCGAA | *240* |
| **PA 245467 Method K** | *181* | ATGATTGTAGAATCTGGCGATCCAAATGCATTATTGAAACACCGATTTGAGATAATCGAA | *240* |
| **PA 245467 Method N** | *181* | ATGATTGTAGAATCTGGCGATCCAAATGCATTATTGAAACACCGATTTGAGATAATCGAA | *240* |

| | | | |
|---|---|---|---|
| **PA 245467 MiSeq** | *241* | GGGAGAGACCGAGCAATGGCCTGGACAGTGGTGAATAGTATCTGCAACACCACAGGGGTC | *300* |
| **PA 245467 Method A** | *241* | GGGAGAGACCGAGCAATGGCCTGGACAGTGGTGAATAGTATCTGCAACACCACAGGGGTC | *300* |
| **PA 245467 Method S** | *241* | GGGAGAGACCGAGCAATGGCCTGGACAGTGGTGAATAGTATCTGCAACACCACAGGAGTC | *300* |
| **PA 245467 Method E** | *241* | GGGAGAGACCGAGCAATGGCCTGGACAGTGGTGAATAGTATCTGCAACACCACAGGGGTC | *300* |
| **PA 245467 Method K** | *241* | GGGAGAGACCGAGCAATGGCCTGGACAGTGGTGAATAGTATCTGCAACACCACAGGGGTC | *300* |
| **PA 245467 Method N** | *241* | GGGAGAGACCGAGCAATGGCCTGGACAGTGGTGAATAGTATCTGCAACACCACAGGGGTC | *300* |

| | | | |
|---|---|---|---|
| **PA 245467 MiSeq** | *301* | GAAAAGCCCAAATTCCTCCCTGATTTGTATGACTACAAAGAGAACCGATTCATTGAAATT | *360* |
| **PA 245467 Method A** | *301* | GAAAAGCCCAAATTCCTCCCTGATTTGTATGACTACAAAGAGAACCGATTCATTGAAATT | *360* |
| **PA 245467 Method S** | *301* | GAAAAGCCCAAATTCCTCCCTGATTTGTATGACTACAAAGAGAACCGATTCATTGAAATT | *360* |
| **PA 245467 Method E** | *301* | GAAAAGCCCAAATTCCTCCCTGATTTGTATGACTACAAAGAGAACCGATTCATTGAAATT | *360* |
| **PA 245467 Method K** | *301* | GAAAAGCCCAAATTCCTCCCTGATTTGTATGACTACAAAGAGAACCGATTCATTGAAATT | *360* |
| **PA 245467 Method N** | *301* | GAAAAGCCCAAATTCCTCCCTGATTTGTATGACTACAAAGAGAACCGATTCATTGAAATT | *360* |

| | | | |
|---|---|---|---|
| **PA 245467 MiSeq** | *361* | GGAGTAACGCGAAGGGAAGTTCACATATACTATTTAGAAAAAGCCAACAAGATAAAATCA | *420* |
| **PA 245467 Method A** | *361* | GGAGTAACGCGAAGGGAAGTTCACATATACTATTTAGAAAAAGCCAACAAGATAAAATCA | *420* |
| **PA 245467 Method S** | *361* | GGAGTAACGCGAAGGGAAGTTCACATATACTATTTAGAAAAAGCCAACAAGATAAAATCA | *420* |
| **PA 245467 Method E** | *361* | GGAGTAACGCGAAGGGAAGTTCACATATACTATTTAGAAAAAGCCAACAAGATAAAATCA | *420* |
| **PA 245467 Method K** | *361* | GGAGTAACGCGAAGGGAAGTTCACATATACTATTTAGAAAAAGCCAACAAGATAAAATCA | *420* |
| **PA 245467 Method N** | *361* | GGAGTAACGCGAAGGGAAGTTCACATATACTATTTAGAAAAAGCCAACAAGATAAAATCA | *420* |

| | | | |
|---|---|---|---|
| **PA 245467 MiSeq** | *421* | GAGAAAACACATATTCACATATTCTCATTCACTGGAGAGGAAATGGCCACCAAGGCGGAC | *480* |
| **PA 245467 Method A** | *421* | GAGAAAACACATATTCACATATTCTCATTCACTGGAGAGGAAATGGCCACCAAGGCGGAC | *480* |
| **PA 245467 Method S** | *421* | GAGAAAACACACATTCACATATTCTCATTCACTGGAGAGGAAATGGCCACCAAGGCGGAC | *480* |
| **PA 245467 Method E** | *421* | GAGAAAACACATATTCACATATTCTCATTCACTGGAGAGGAAATGGCCACCAAGGCGGAC | *480* |
| **PA 245467 Method K** | *421* | GAGAAAACACATATTCACATATTCTCATTCACTGGAGAGGAAATGGCCACCAAGGCGGAC | *480* |
| **PA 245467 Method N** | *421* | GAGAAAACACATATTCACATATTCTCATTCACTGGAGAGGAAATGGCCACCAAGGCGGAC | *480* |

| | | | |
|---|---|---|---|
| **PA 245467 MiSeq** | *481* | TACACCCTTGATGAAGAGAGCAGGGCAAGAATAAAAACCAGACTGTTCACTATAAGACAA | *540* |
| **PA 245467 Method A** | *481* | TACACCCTTGATGAAGAGAGCAGGGCAAGAATAAAAACCAGACTGTTCACTATAAGACAA | *540* |
| **PA 245467 Method S** | *481* | TACACCCTTGATGAAGA**A**AGCAG**A**GCAAGAATAAAAACCAGACTGTTCACTATAAGACAA | *540* |
| **PA 245467 Method E** | *481* | TACACCCTTGATGAAGAGAGCAGGGCAAGAATAAAAACCAGACTGTTCACTATAAGACAA | *540* |
| **PA 245467 Method K** | *481* | TACACCCTTGATGAAGAGAGCAGGGCAAGAATAAAAACCAGACTGTTCACTATAAGACAA | *540* |
| **PA 245467 Method N** | *481* | TACACCCTTGATGAAGAGAGCAGGGCAAGAATAAAAACCAGACTGTTCACTATAAGACAA | *540* |

| | | | |
|---|---|---|---|
| **PA 245467 MiSeq** | *541* | GAAATGGCCAGTAGAGGTCTATGGGATTCCTTTCGTCAGTCCGAGAGAGGCGAAGAGACA | *600* |
| **PA 245467 Method A** | *541* | GAAATGGCCAGTAGAGGTCTATGGGATTCCTTTCGTCAGTCCGAGAGAGGCGAAGAGACA | *600* |
| **PA 245467 Method S** | *541* | GAAATGGC**T**AGTAGAGGTCTATGGGATTCCTTTCGTCAGTCCGAGAGAGGCGAAGAGACA | *600* |
| **PA 245467 Method E** | *541* | GAAATGGCCAGTAGAGGTCTATGGGATTCCTTTCGTCAGTCCGAGAGAGGCGAAGAGACA | *600* |
| **PA 245467 Method K** | *541* | GAAATGGCCAGTAGAGGTCTATGGGATTCCTTTCGTCAGTCCGAGAGAGGCGAAGAGACA | *600* |
| **PA 245467 Method N** | *541* | GAAATGGCCAGTAGAGGTCTATGGGATTCCTTTCGTCAGTCCGAGAGAGGCGAAGAGACA | *600* |

| | | | |
|---|---|---|---|
| **PA 245467 MiSeq** | *601* | ATTGAAGAAAGATTTGAAATCACAGGAACCATGCGCAGGCTTGCCGACCAAAGCCTCCCA | *660* |
| **PA 245467 Method A** | *601* | ATTGAAGAAAGATTTGAAATCACAGGAACCATGCGCAGGCTTGCCGACCAAAGCCTCCCA | *660* |
| **PA 245467 Method S** | *601* | ATTGAAGAAAGATTTGAAATCACAGGAACCATGCGCAGGCTTGCCGACCAAAG**T**CTCCCA | *660* |
| **PA 245467 Method E** | *601* | ATTGAAGAAAGATTTGAAATCACAGGAACCATGCGCAGGCTTGCCGACCAAAGCCTCCCA | *660* |
| **PA 245467 Method K** | *601* | ATTGAAGAAAGATTTGAAATCACAGGAACCATGCGCAGGCTTGCCGACCAAAGCCTCCCA | *660* |
| **PA 245467 Method N** | *601* | ATTGAAGAAAGATTTGAAATCACAGGAACCATGCGCAGGCTTGCCGACCAAAGCCTCCCA | *660* |

| | | | |
|---|---|---|---|
| **PA 245467 MiSeq** | *661* | CCGAACTTCTCCAGCCTTGAAAACTTTAGAGCCTATGTGGATGGATTCGAACCGAACGGC | *720* |
| **PA 245467 Method A** | *661* | CCGAACTTCTCCAGCCTTGAAAACTTTAGAGCCTATGTGGATGGATTCGAACCGAACGGC | *720* |
| **PA 245467 Method S** | *661* | CCGAACTTCTCCAGCCTTGAAAACTTTAGAGCCTATGTGGATGGATTCGAACCGAACGGC | *720* |
| **PA 245467 Method E** | *661* | CCGAACTTCTCCAGCCTTGAAAACTTTAGAGCCTATGTGGATGGATTCGAACCGAACGGC | *720* |
| **PA 245467 Method K** | *661* | CCGAACTTCTCCAGCCTTGAAAACTTTAGAGCCTATGTGGATGGATTCGAACCGAACGGC | *720* |
| **PA 245467 Method N** | *661* | CCGAACTTCTCCAGCCTTGAAAACTTTAGAGCCTATGTGGATGGATTCGAACCGAACGGC | *720* |

| | | | |
|---|---|---|---|
| **PA 245467 MiSeq** | *721* | TGCATTGAGGGCAAACTTTCTCAAATGTCAAAAGAAGTGAACGCCAGAATTGAGCCATTT | *780* |
| **PA 245467 Method A** | *721* | TGCATTGAGGGCAAACTTTCTCAAATGTCAAAAGAAGTGAACGCCAGAATTGAGCCATTT | *780* |
| **PA 245467 Method S** | *721* | TGCATTGAGGGCAAGCTTTCTCAAATGTCAAAAGAAGTGAACGCCAGAATTGAGCCATTT | *780* |
| **PA 245467 Method E** | *721* | TGCATTGAGGGCAAACTTTCTCAAATGTCAAAAGAAGTGAACGCCAGAATTGAGCCATTT | *780* |
| **PA 245467 Method K** | *721* | TGCATTGAGGGCAAACTTTCTCAAATGTCAAAAGAAGTGAACGCCAGAATTGAGCCATTT | *780* |
| **PA 245467 Method N** | *721* | TGCATTGAGGGCAAACTTTCTCAAATGTCAAAAGAAGTGAACGCCAGAATTGAGCCATTT | *780* |

| | | | |
|---|---|---|---|
| **PA 245467 MiSeq** | *781* | CTGAAGACAACACCACGCCCTCTCAGATTACCTGATGGGCCTCTCTGTTCTCAGCGGTCG | *840* |
| **PA 245467 Method A** | *781* | CTGAAGACAACACCACGCCCTCTCAGATTACCTGATGGGCCTCTCTGTTCTCAGCGGTCG | *840* |
| **PA 245467 Method S** | *781* | CTGAAGACAACACCACGCCCTCTCAGATTACCTGATGGGCCTCCCTGTTCTCAGCGGTCG | *840* |
| **PA 245467 Method E** | *781* | CTGAAGACAACACCACGCCCTCTCAGATTACCTGATGGGCCTCTCTGTTCTCAGCGGTCG | *840* |
| **PA 245467 Method K** | *781* | CTGAAGACAACACCACGCCCTCTCAGATTACCTGATGGGCCTCTCTGTTCTCAGCGGTCG | *840* |
| **PA 245467 Method N** | *781* | CTGAAGACAACACCACGCCCTCTCAGATTACCTGATGGGCCTCTCTGTTCTCAGCGGTCG | *840* |

| | | | |
|---|---|---|---|
| **PA 245467 MiSeq** | *841* | AAGTTCTTGCTGATGGATGCCCTTAAGTTGAGCATCGAAGACCCTAGCCATGAGGGGGAG | *900* |
| **PA 245467 Method A** | *841* | AAGTTCTTGCTGATGGATGCCCTTAAGTTGAGCATCGAAGACCCTAGCCATGAGGGGGAG | *900* |
| **PA 245467 Method S** | *841* | AAGTTCTTACTGATGGATGCCCTTAAGTTGAGCATCGAAGACCCTAGCCATGAGGGAGAG | *900* |
| **PA 245467 Method E** | *841* | AAGTTCTTGCTGATGGATGCCCTTAAGTTGAGCATCGAAGACCCTAGCCATGAGGGGGAG | *900* |
| **PA 245467 Method K** | *841* | AAGTTCTTGCTGATGGATGCCCTTAAGTTGAGCATCGAAGACCCTAGCCATGAGGGGGAG | *900* |
| **PA 245467 Method N** | *841* | AAGTTCTTGCTGATGGATGCCCTTAAGTTGAGCATCGAAGACCCTAGCCATGAGGGGGAG | *900* |

| | | | |
|---|---|---|---|
| **PA 245467 MiSeq** | *901* | GGCATACCGCTGTATGATGCAATCAAATGCATGAAGACATTTTTTGGCTGGAAAGAGCCC | *960* |
| **PA 245467 Method A** | *901* | GGCATACCGCTGTATGATGCAATCAAATGCATGAAGACATTTTTTGGCTGGAAAGAGCCC | *960* |
| **PA 245467 Method S** | *901* | GGCATACCGCTGTATGATGCAATCAAATGCATGAAGACATTTTTTGGCTGGAAAGAGCCC | *960* |
| **PA 245467 Method E** | *901* | GGCATACCGCTGTATGATGCAATCAAATGCATGAAGACATTTTTTGGCTGGAAAGAGCCC | *960* |
| **PA 245467 Method K** | *901* | GGCATACCGCTGTATGATGCAATCAAATGCATGAAGACATTTTTTGGCTGGAAAGAGCCC | *960* |
| **PA 245467 Method N** | *901* | GGCATACCGCTGTATGATGCAATCAAATGCATGAAGACATTTTTTGGCTGGAAAGAGCCC | *960* |

| | | | |
|---|---|---|---|
| **PA 245467 MiSeq** | *961* | AACATCGTAAAGCCGCATGAGAAAGGCATAAACCCTAATTACCTCCTGGCTTGGAAGCAG | *1020* |
| **PA 245467 Method A** | *961* | AACATCGTAAAGCCGCATGAGAAAGGCATAAACCCTAATTACCTCCTGGCTTGGAAGCAG | *1020* |
| **PA 245467 Method S** | *961* | AACATCGTAAAGCCGCATGAGAAAGGCATAAACCCTAATTACCTCCTGGCTTGGAAGCAG | *1020* |
| **PA 245467 Method E** | *961* | AACATCGTAAAGCCGCATGAGAAAGGCATAAACCCTAATTACCTCCTGGCTTGGAAGCAG | *1020* |
| **PA 245467 Method K** | *961* | AACATCGTAAAGCCGCATGAGAAAGGCATAAACCCTAATTACCTCCTGGCTTGGAAGCAG | *1020* |
| **PA 245467 Method N** | *961* | AACATCGTAAAGCCGCATGAGAAAGGCATAAACCCTAATTACCTCCTGGCTTGGAAGCAG | *1020* |

| | | | |
|---|---|---|---|
| **PA 245467 MiSeq** | *1021* | GTGCTGGCAGAACTTCAAGACATTGAAAATGAGGAGAAAATTCCAAAAACAAAGAACATG | *1080* |
| **PA 245467 Method A** | *1021* | GTGCTGGCAGAACTTCAAGACATTGAAAATGAGGAGAAAATTCCAAAAACAAAGAACATG | *1080* |
| **PA 245467 Method S** | *1021* | GTGCTGGCAGAACTTCAAGATGTTGAAAATGAGGAGAAAATTCCAAAAACAAAGAACATG | *1080* |
| **PA 245467 Method E** | *1021* | GTGCTGGCAGAACTTCAAGACATTGAAAATGAGGAGAAAATTCCAAAAACAAAGAACATG | *1080* |
| **PA 245467 Method K** | *1021* | GTGCTGGCAGAACTTCAAGACATTGAAAATGAGGAGAAAATTCCAAAAACAAAGAACATG | *1080* |
| **PA 245467 Method N** | *1021* | GTGCTGGCAGAACTTCAAGACATTGAAAATGAGGAGAAAATTCCAAAAACAAAGAACATG | *1080* |

| | | | |
|---|---|---|---|
| **PA 245467 MiSeq** | *1081* | AAGAAAACAAGCCAATTGAAGTGGGCACTTGGTGAGAACATGGCTCCAGAAAAAGTGGAC | *1140* |
| **PA 245467 Method A** | *1081* | AAGAAAACAAGCCAATTGAAGTGGGCACTTGGTGAGAACATGGCTCCAGAAAAAGTGGAC | *1140* |
| **PA 245467 Method S** | *1081* | AAGAAAACAAGCCAATTGAAGTGGGCACTTGGTGAGAACATAGCGCCAGAAAAAGTGGAC | *1140* |
| **PA 245467 Method E** | *1081* | AAGAAAACAAGCCAATTGAAGTGGGCACTTGGTGAGAACATGGCTCCAGAAAAAGTGGAC | *1140* |
| **PA 245467 Method K** | *1081* | AAGAAAACAAGCCAATTGAAGTGGGCACTTGGTGAGAACATGGCTCCAGAAAAAGTGGAC | *1140* |
| **PA 245467 Method N** | *1081* | AAGAAAACAAGCCAATTGAAGTGGGCACTTGGTGAGAACATGGCTCCAGAAAAAGTGGAC | *1140* |

| | | | |
|---|---|---|---|
| **PA 245467 MiSeq** | *1141* | TTTGAGGACTGCAAAGATGTTAGCGATCTAAGACAGTACGACAGTGACGAACCAGAGTCT | *1200* |
| **PA 245467 Method A** | *1141* | TTTGAGGACTGCAAAGATGTTAGCGATCTAAGACAGTACGACAGTGACGAACCAGAGTCT | *1200* |
| **PA 245467 Method S** | *1141* | TTTGAGGACTGCAAAGATGTTAGCGATCTAAGACAATACGACAGTGACGAACCAGAGTCT | *1200* |
| **PA 245467 Method E** | *1141* | TTTGAGGACTGCAAAGATGTTAGCGATCTAAGACAGTACGACAGTGACGAACCAGAGTCT | *1200* |
| **PA 245467 Method K** | *1141* | TTTGAGGACTGCAAAGATGTTAGCGATCTAAGACAGTACGACAGTGACGAACCAGAGTCT | *1200* |
| **PA 245467 Method N** | *1141* | TTTGAGGACTGCAAAGATGTTAGCGATCTAAGACAGTACGACAGTGACGAACCAGAGTCT | *1200* |

| | | | |
|---|---|---|---|
| **PA 245467 MiSeq** | *1201* | AGATCACTAGCAAGCTGGATTCAGAGTGAATTCAACAAGGCATGCGAACTGACAGATTCG | *1260* |
| **PA 245467 Method A** | *1201* | AGATCACTAGCAAGCTGGATTCAGAGTGAATTCAACAAGGCATGCGAACTGACAGATTCG | *1260* |
| **PA 245467 Method S** | *1201* | AGATCACTAGCAAGCTGGATTCAGAGTGAATTCAACAAGGCATGCGAATTGACAGATTCG | *1260* |
| **PA 245467 Method E** | *1201* | AGATCACTAGCAAGCTGGATTCAGAGTGAATTCAACAAGGCATGCGAACTGACAGATTCG | *1260* |
| **PA 245467 Method K** | *1201* | AGATCACTAGCAAGCTGGATTCAGAGTGAATTCAACAAGGCATGCGAACTGACAGATTCG | *1260* |
| **PA 245467 Method N** | *1201* | AGATCACTAGCAAGCTGGATTCAGAGTGAATTCAACAAGGCATGCGAACTGACAGATTCG | *1260* |
| **PA 245467 MiSeq** | *1261* | AGTTGGATTGAACTTGATGAGATAGGGGAAGACGTTGCTCCAATCGAACACATTGCGAGT | *1320* |
| **PA 245467 Method A** | *1261* | AGTTGGATTGAACTTGATGAGATAGGGGAAGACGTTGCTCCAATCGAACACATTGCGAGT | *1320* |
| **PA 245467 Method S** | *1261* | AGTTGGATTGAACTTGATGAGATAGGGGAAGACGTTGCTCCAATCGAACACATTGCGAGT | *1320* |
| **PA 245467 Method E** | *1261* | AGTTGGATTGAACTTGATGAGATAGGGGAAGACGTTGCTCCAATCGAACACATTGCGAGT | *1320* |
| **PA 245467 Method K** | *1261* | AGTTGGATTGAACTTGATGAGATAGGGGAAGACGTTGCTCCAATCGAACACATTGCGAGT | *1320* |
| **PA 245467 Method N** | *1261* | AGTTGGATTGAACTTGATGAGATAGGGGAAGACGTTGCTCCAATCGAACACATTGCGAGT | *1320* |
| **PA 245467 MiSeq** | *1321* | GTGAGGAGGAACTATTTCACAGCGGAGGTATCCCATTGCAGGGCCACTGAATACATAATG | *1380* |
| **PA 245467 Method A** | *1321* | GTGAGGAGGAACTATTTCACAGCGGAGGTATCCCATTGCAGGGCCACTGAATACATAATG | *1380* |
| **PA 245467 Method S** | *1321* | ATGAGGAGGAACTATTTCACAGCGGAGGTATCCCATTGCAGGGCCACTGAATACATAATG | *1380* |
| **PA 245467 Method E** | *1321* | GTGAGGAGGAACTATTTCACAGCGGAGGTATCCCATTGCAGGGCCACTGAATACATAATG | *1380* |
| **PA 245467 Method K** | *1321* | GTGAGGAGGAACTATTTCACAGCGGAGGTATCCCATTGCAGGGCCACTGAATACATAATG | *1380* |
| **PA 245467 Method N** | *1321* | GTGAGGAGGAACTATTTCACAGCGGAGGTATCCCATTGCAGGGCCACTGAATACATAATG | *1380* |
| **PA 245467 MiSeq** | *1381* | AAGGGAGTATACATAAACACAGCCCTATTGAATGCATCCTGTGCAGCCATGGATGACTTC | *1440* |
| **PA 245467 Method A** | *1381* | AAGGGAGTATACATAAACACAGCCCTATTGAATGCATCCTGTGCAGCCATGGATGACTTC | *1440* |
| **PA 245467 Method S** | *1381* | AAGGGAGTATACATAAACACAGCCCTATTGAATGCATCCTGTGCAGCCATGGATGACTTC | *1440* |
| **PA 245467 Method E** | *1381* | AAGGGAGTATACATAAACACAGCCCTATTGAATGCATCCTGTGCAGCCATGGATGACTTC | *1440* |
| **PA 245467 Method K** | *1381* | AAGGGAGTATACATAAACACAGCCCTATTGAATGCATCCTGTGCAGCCATGGATGACTTC | *1440* |
| **PA 245467 Method N** | *1381* | AAGGGAGTATACATAAACACAGCCCTATTGAATGCATCCTGTGCAGCCATGGATGACTTC | *1440* |

| | | | |
|---|---|---|---|
| **PA 245467 MiSeq** | *1441* | CAACTGATTCCAATGATAAGTAAGTGCAGAACTAAGGAAGGAAGACGGAAGACAAATCTG | *1500* |
| **PA 245467 Method A** | *1441* | CAACTGATTCCAATGATAAGTAAGTGCAGAACTAAGGAAGGAAGACGGAAGACAAATCTG | *1500* |
| **PA 245467 Method S** | *1441* | CAACTGATTCCAATGATAAG**C**AAGTGCAGAAC**C**AA**A**GAAGGAAGACGGAAGACAAATCTG | *1500* |
| **PA 245467 Method E** | *1441* | CAACTGATTCCAATGATAAGTAAGTGCAGAACTAAGGAAGGAAGACGGAAGACAAATCTG | *1500* |
| **PA 245467 Method K** | *1441* | CAACTGATTCCAATGATAAGTAAGTGCAGAACTAAGGAAGGAAGACGGAAGACAAATCTG | *1500* |
| **PA 245467 Method N** | *1441* | CAACTGATTCCAATGATAAGTAAGTGCAGAACTAAGGAAGGAAGACGGAAGACAAATCTG | *1500* |

| | | | |
|---|---|---|---|
| **PA 245467 MiSeq** | *1501* | TATGGATTCATTATAAAAGGAAGATCCCATTTGAGGAATGACACCGATGTGGTAAACTTT | *1560* |
| **PA 245467 Method A** | *1501* | TATGGATTCATTATAAAAGGAAGATCCCATTTGAGGAATGACACCGATGTGGTAAACTTT | *1560* |
| **PA 245467 Method S** | *1501* | TATGGATTCATTATAAAAGGAAGATCCCATTTGAGGAATGACACCGATGTGGTAAACTTT | *1560* |
| **PA 245467 Method E** | *1501* | TATGGATTCATTATAAAAGGAAGATCCCATTTGAGGAATGACACCGATGTGGTAAACTTT | *1560* |
| **PA 245467 Method K** | *1501* | TATGGATTCATTATAAAAGGAAGATCCCATTTGAGGAATGACACCGATGTGGTAAACTTT | *1560* |
| **PA 245467 Method N** | *1501* | TATGGATTCATTATAAAAGGAAGATCCCATTTGAGGAATGACACCGATGTGGTAAACTTT | *1560* |

| | | | |
|---|---|---|---|
| **PA 245467 MiSeq** | *1561* | GTGAGCATGGAATTCTCTCTAACTGACCCGAGGCTAGAGCCACACAAATGGGAAAAGTAC | *1620* |
| **PA 245467 Method A** | *1561* | GTGAGCATGGAATTCTCTCTAACTGACCCGAGGCTAGAGCCACACAAATGGGAAAAGTAC | *1620* |
| **PA 245467 Method S** | *1561* | GTGAGCATGGAATTCTCTCTAACTGACCCGAGGCTAGAGCCACACAAATGGGAAAAGTAC | *1620* |
| **PA 245467 Method E** | *1561* | GTGAGCATGGAATTCTCTCTAACTGACCCGAGGCTAGAGCCACACAAATGGGAAAAGTAC | *1620* |
| **PA 245467 Method K** | *1561* | GTGAGCATGGAATTCTCTCTAACTGACCCGAGGCTAGAGCCACACAAATGGGAAAAGTAC | *1620* |
| **PA 245467 Method N** | *1561* | GTGAGCATGGAATTCTCTCTAACTGACCCGAGGCTAGAGCCACACAAATGGGAAAAGTAC | *1620* |

| | | | |
|---|---|---|---|
| **PA 245467 MiSeq** | *1621* | TGTGTTCTTGAGATAGGAGACATGCTCCTACGGACTGCAATAGGCCAAGTGTCGAGGCCC | *1680* |
| **PA 245467 Method A** | *1621* | TGTGTTCTTGAGATAGGAGACATGCTCCTACGGACTGCAATAGGCCAAGTGTCGAGGCCC | *1680* |
| **PA 245467 Method S** | *1621* | TGTGTTCTTGAGATAGGAGACATGCTCCTACGGACTGCAATAGGCCAAGTGTCGAGGCCC | *1680* |
| **PA 245467 Method E** | *1621* | TGTGTTCTTGAGATAGGAGACATGCTCCTACGGACTGCAATAGGCCAAGTGTCGAGGCCC | *1680* |
| **PA 245467 Method K** | *1621* | TGTGTTCTTGAGATAGGAGACATGCTCCTACGGACTGCAATAGGCCAAGTGTCGAGGCCC | *1680* |
| **PA 245467 Method N** | *1621* | TGTGTTCTTGAGATAGGAGACATGCTCCTACGGACTGCAATAGGCCAAGTGTCGAGGCCC | *1680* |

| | | | |
|---|---|---|---|
| **PA 245467 MiSeq** | *1681* | ATGTTCCTGTATGTGAGAACCAATGGGACTTCCAAGATCAAAATGAAATGGGGCATGGAG | *1740* |
| **PA 245467 Method A** | *1681* | ATGTTCCTGTATGTGAGAACCAATGGGACTTCCAAGATCAAAATGAAATGGGGCATGGAG | *1740* |
| **PA 245467 Method S** | *1681* | ATGTTCCTGTATGTGAGAACCAATGGGACTTCCAAGATCAAAATGAAATGGGGCATGGAG | *1740* |
| **PA 245467 Method E** | *1681* | ATGTTCCTGTATGTGAGAACCAATGGGACTTCCAAGATCAAAATGAAATGGGGCATGGAG | *1740* |
| **PA 245467 Method K** | *1681* | ATGTTCCTGTATGTGAGAACCAATGGGACTTCCAAGATCAAAATGAAATGGGGCATGGAG | *1740* |
| **PA 245467 Method N** | *1681* | ATGTTCCTGTATGTGAGAACCAATGGGACTTCCAAGATCAAAATGAAATGGGGCATGGAG | *1740* |

| | | | |
|---|---|---|---|
| **PA 245467 MiSeq** | *1741* | ATGAGGCGATGCCTTCTTCAGTCCCTTCAACAAATTGAGAGCATGATTGAGGCCGAATCT | *1800* |
| **PA 245467 Method A** | *1741* | ATGAGGCGATGCCTTCTTCAGTCCCTTCAACAAATTGAGAGCATGATTGAGGCCGAATCT | *1800* |
| **PA 245467 Method S** | *1741* | ATGAGACGATGCCTTCTTCAGTCCCTTCAACAAATTGAGAGCACGATTGAGGCCGAATCT | *1800* |
| **PA 245467 Method E** | *1741* | ATGAGGCGATGCCTTCTTCAGTCCCTTCAACAAATTGAGAGCATGATTGAGGCCGAATCT | *1800* |
| **PA 245467 Method K** | *1741* | ATGAGGCGATGCCTTCTTCAGTCCCTTCAACAAATTGAGAGCATGATTGAGGCCGAATCT | *1800* |
| **PA 245467 Method N** | *1741* | ATGAGGCGATGCCTTCTTCAGTCCCTTCAACAAATTGAGAGCATGATTGAGGCCGAATCT | *1800* |

| | | | |
|---|---|---|---|
| **PA 245467 MiSeq** | *1801* | TCTGTCAAAGAGAAGGACATGTCCAAGGAATTCTTTGAAAACAAATCAGAAACATGGCCA | *1860* |
| **PA 245467 Method A** | *1801* | TCTGTCAAAGAGAAGGACATGTCCAAGGAATTCTTTGAAAACAAATCAGAAACATGGCCA | *1860* |
| **PA 245467 Method S** | *1801* | TCTGTCAAAGAGAAGGACATGACCAAGGAATTCTTTGAAAACAAATCAGAAACATGGCCA | *1860* |
| **PA 245467 Method E** | *1801* | TCTGTCAAAGAGAAGGACATGTCCAAGGAATTCTTTGAAAACAAATCAGAAACATGGCCA | *1860* |
| **PA 245467 Method K** | *1801* | TCTGTCAAAGAGAAGGACATGTCCAAGGAATTCTTTGAAAACAAATCAGAAACATGGCCA | *1860* |
| **PA 245467 Method N** | *1801* | TCTGTCAAAGAGAAGGACATGTCCAAGGAATTCTTTGAAAACAAATCAGAAACATGGCCA | *1860* |

| | | | |
|---|---|---|---|
| **PA 245467 MiSeq** | *1861* | ATTGGAGAATCACCCAAAGGGGTGGAGGAAGGCTCTATTGGGAAAGTATGCAGAACATTG | *1920* |
| **PA 245467 Method A** | *1861* | ATTGGAGAATCACCCAAAGGGGTGGAGGAAGGCTCTATTGGGAAAGTATGCAGAACATTG | *1920* |
| **PA 245467 Method S** | *1861* | ATTGGAGAATCACCCAAAGGGGTGGAGGAAGGCTCTATTGGGAAAGTATGCAGAACATTG | *1920* |
| **PA 245467 Method E** | *1861* | ATTGGAGAATCACCCAAAGGGGTGGAGGAAGGCTCTATTGGGAAAGTATGCAGAACATTG | *1920* |
| **PA 245467 Method K** | *1861* | ATTGGAGAATCACCCAAAGGGGTGGAGGAAGGCTCTATTGGGAAAGTATGCAGAACATTG | *1920* |
| **PA 245467 Method N** | *1861* | ATTGGAGAATCACCCAAAGGGGTGGAGGAAGGCTCTATTGGGAAAGTATGCAGAACATTG | *1920* |

| | | | |
|---|---|---|---|
| **PA 245467 MiSeq** | *1921* | CTAGCAAAGTCTGTGTTCAACAGCCTATATGCATCTCCACAACTCGAGGGGTTTTCAGCT | *1980* |
| **PA 245467 Method A** | *1921* | CTAGCAAAGTCTGTGTTCAACAGCCTATATGCATCTCCACAACTCGAGGGGTTTTCAGCT | *1980* |
| **PA 245467 Method S** | *1921* | CTAGCAAAGTCTGTGTTCAACAGCCTATATGCATCTCCACAACTCGAGGGGTTTTCAGCT | *1980* |
| **PA 245467 Method E** | *1921* | CTAGCAAAGTCTGTGTTCAACAGCCTATATGCATCTCCACAACTCGAGGGGTTTTCAGCT | *1980* |
| **PA 245467 Method K** | *1921* | CTAGCAAAGTCTGTGTTCAACAGCCTATATGCATCTCCACAACTCGAGGGGTTTTCAGCT | *1980* |
| **PA 245467 Method N** | *1921* | CTAGCAAAGTCTGTGTTCAACAGCCTATATGCATCTCCACAACTCGAGGGGTTTTCAGCT | *1980* |

| | | | |
|---|---|---|---|
| **PA 245467 MiSeq** | *1981* | GAATCAAGAAAATTGCTTCTCATTGTTCAGGCACTTAGGGACAACCTGGAACCTGGAACC | *2040* |
| **PA 245467 Method A** | *1981* | GAATCAAGAAAATTGCTTCTCATTGTTCAGGCACTTAGGGACAACCTGGAACCTGGAACC | *2040* |
| **PA 245467 Method S** | *1981* | GAATCAAGAAAATTGCTTCTCATTGTTCAGGCACTTAGGGACAACCTGGAACCTGGAACC | *2040* |
| **PA 245467 Method E** | *1981* | GAATCAAGAAAATTGCTTCTCATTGTTCAGGCACTTAGGGACAACCTGGAACCTGGAACC | *2040* |
| **PA 245467 Method K** | *1981* | GAATCAAGAAAATTGCTTCTCATTGTTCAGGCACTTAGGGACAACCTGGAACCTGGAACC | *2040* |
| **PA 245467 Method N** | *1981* | GAATCAAGAAAATTGCTTCTCATTGTTCAGGCACTTAGGGACAACCTGGAACCTGGAACC | *2040* |

| | | | |
|---|---|---|---|
| **PA 245467 MiSeq** | *2041* | TTCGATCTTGGGGGGCTATATGAAGCAATTGAGGAGTGCCTGATTAACGATCCCTGGGTT | *2100* |
| **PA 245467 Method A** | *2041* | TTCGATCTTGGGGGGCTATATGAAGCAATTGAGGAGTGCCTGATTAACGATCCCTGGGTT | *2100* |
| **PA 245467 Method S** | *2041* | TTCGATCTTGGGGGGCTATATGAAGCAATTGAGGAGTGCCTGATTAACGATCCCTGGGTT | *2100* |
| **PA 245467 Method E** | *2041* | TTCGATCTTGGGGGGCTATATGAAGCAATTGAGGAGTGCCTGATTAACGATCCCTGGGTT | *2100* |
| **PA 245467 Method K** | *2041* | TTCGATCTTGGGGGGCTATATGAAGCAATTGAGGAGTGCCTGATTAACGATCCCTGGGTT | *2100* |
| **PA 245467 Method N** | *2041* | TTCGATCTTGGGGGGCTATATGAAGCAATTGAGGAGTGCCTGATTAACGATCCCTGGGTT | *2100* |

| | | | |
|---|---|---|---|
| **PA 245467 MiSeq** | *2101* | TTGCTTAATGCGTCTTGGTTCAACTCCTTCCTCACACATGCACTGAAATAG | *2151* |
| **PA 245467 Method A** | *2101* | TTGCTTAATGCGTCTTGGTTCAACTCCTTCCTCACACATGCACTGAAATAG | *2151* |
| **PA 245467 Method S** | *2101* | TTGCTTAATGCGTCTTGGTTCAACTCCTTCCTCACACATGCACTGAAATAG | *2151* |
| **PA 245467 Method E** | *2101* | TTGCTTAATGCGTCTTGGTTCAACTCCTTCCTCACACATGCACTGAAATAG | *2151* |
| **PA 245467 Method K** | *2101* | TTGCTTAATGCGTCTTGGTTCAACTCCTTCCTCACACATGCACTGAAATAG | *2151* |
| **PA 245467 Method N** | *2101* | TTGCTTAATGCGTCTTGGTTCAACTCCTTCCTCACACATGCACTGAAATAG | *2151* |
