# Supplementary material for: An Evaluation of Avian Influenza Virus Whole-Genome Sequencing Approaches Using Nanopore Technology
Source: Microorganisms. 2023 Feb 19;11(2):529. doi: 10.3390/microorganisms11020529 (PMC9967579; doi:10.3390/microorganisms11020529)
Supplement: Supplementary file 1 [file microorganisms-11-00529-s001.zip › manuscript.v8 230219 Suppl Figures and Tables/Supplementary Figures S1a-h 245467/Supplementary Figure S1e NP.pdf]

Formatted Alignments

**NP 245467 MiSeq**	*1*	ATGGCTTCTCAAGGCACCAAACGATCCTATGAACAAATGGAAACTGGTGGGGAACGCCAG	*60*
**NP 245467 Method A**	*1*	ATGGCTTCTCAAGGCACCAAACGATCCTATGAACAAATGGAAACTGGTGGGGAACGCCAG	*60*
**NP 245467 Method S**	*1*	ATGGCGTCTCAAGGCACCAAACGATCCTATGAACAAATGGAAACTGGTGGGGAGCGCCAG	*60*
**NP 245467 Method E**	*1*	ATGGCTTCTCAAGGCACCAAACGATCCTATGAACAAATGGAAACTGGTGGGGAACGCCAG	*60*
**NP 245467 method K**	*1*	ATGGCTTCTCAAGGCACCAAACGATCCTATGAACAAATGGAAACTGGTGGGGAACGCCAG	*60*
**NP 245467 Method N**	*1*	ATGGCTTCTCAAGGCACCAAACGATCCTATGAACAAATGGAAACTGGTGGGGAACGCCAG	*60*

**NP 245467 MiSeq**	*61*	AATGCCACTGAAATCAGAGCATCTGTTGGAAGAATGGTTGGCGGAATCGGGAGATTCTAC	*120*
**NP 245467 Method A**	*61*	AATGCCACTGAAATCAGAGCATCTGTTGGAAGAATGGTTGGCGGAATCGGGAGATTCTAC	*120*
**NP 245467 Method S**	*61*	AATGCCACTGAAATCAGAGCATCTGTTGGAAGAATGGTTGGCGGAATCGGGAGATTCTAC	*120*
**NP 245467 Method E**	*61*	AATGCCACTGAAATCAGAGCATCTGTTGGAAGAATGGTTGGCGGAATCGGGAGATTCTAC	*120*
**NP 245467 method K**	*61*	AATGCCACTGAAATCAGAGCATCTGTTGGAAGAATGGTTGGCGGAATCGGGAGATTCTAC	*120*
**NP 245467 Method N**	*61*	AATGCCACTGAAATCAGAGCATCTGTTGGAAGAATGGTTGGCGGAATCGGGAGATTCTAC	*120*

**NP 245467 MiSeq**	*121*	ATACAGATGTGCACTGAGCTCAAACTCAGTGATTACGAAGGGAGGCTGATCCAAAACAGC	*180*
**NP 245467 Method A**	*121*	ATACAGATGTGCACTGAGCTCAAACTCAGTGATTACGAAGGGAGGCTGATCCAAAACAGC	*180*
**NP 245467 Method S**	*121*	ATACAAATGTGCACTGAGCTCAAACTCAGTGATTACGAAGGGAGGCTGATCCAAAACAGC	*180*
**NP 245467 Method E**	*121*	ATACAGATGTGCACTGAGCTCAAACTCAGTGATTACGAAGGGAGGCTGATCCAAAACAGC	*180*
**NP 245467 method K**	*121*	ATACAGATGTGCACTGAGCTCAAACTCAGTGATTACGAAGGGAGGCTGATCCAAAACAGC	*180*
**NP 245467 Method N**	*121*	ATACAGATGTGCACTGAGCTCAAACTCAGTGATTACGAAGGGAGGCTGATCCAAAACAGC	*180*

**NP 245467 MiSeq**	*181*	ATAACCATAGAAAGGATGGTTCTCTCGGCATTTGATGAGAGGAGGAACAAGTATCTGGAG	*240*
**NP 245467 Method A**	*181*	ATAACCATAGAAAGGATGGTTCTCTCGGCATTTGATGAGAGGAGGAACAAGTATCTGGAG	*240*
**NP 245467 Method S**	*181*	ATAACCATAGAAAGGATGGTTCTCTCGGCATTTGATGAGAGGAGGAACAAGTATCTGGAG	*240*
**NP 245467 Method E**	*181*	ATAACCATAGAAAGGATGGTTCTCTCGGCATTTGATGAGAGGAGGAACAAGTATCTGGAG	*240*
**NP 245467 method K**	*181*	ATAACCATAGAAAGGATGGTTCTCTCGGCATTTGATGAGAGGAGGAACAAGTATCTGGAG	*240*
**NP 245467 Method N**	*181*	ATAACCATAGAAAGGATGGTTCTCTCGGCATTTGATGAGAGGAGGAACAAGTATCTGGAG	*240*

**NP 245467 MiSeq**	*241*	GAACATCCCAGTGCTGGGAAGGATCCCAAGAAGACTGGAGGTCCAATCTACAGGAGGAGA	*300*
**NP 245467 Method A**	*241*	GAACATCCCAGTGCTGGGAAGGATCCCAAGAAGACTGGAGGTCCAATCTACAGGAGGAGA	*300*
**NP 245467 Method S**	*241*	GAACATCCCAGTGCTGGGAAGGATCCCAAGAAGACTGGAGGTCCAATCTACAGGAGGAGA	*300*
**NP 245467 Method E**	*241*	GAACATCCCAGTGCTGGGAAGGATCCCAAGAAGACTGGAGGTCCAATCTACAGGAGGAGA	*300*
**NP 245467 method K**	*241*	GAACATCCCAGTGCTGGGAAGGATCCCAAGAAGACTGGAGGTCCAATCTACAGGAGGAGA	*300*
**NP 245467 Method N**	*241*	GAACATCCCAGTGCTGGGAAGGATCCCAAGAAGACTGGAGGTCCAATCTACAGGAGGAGA	*300*

**NP 245467 MiSeq**	*301*	GATGGCAAATGGATGAGAGAGTTGATCCTCTACGACAAAGAAGAGATCAGAAGAATTTGG	*360*
**NP 245467 Method A**	*301*	GATGGCAAATGGATGAGAGAGTTGATCCTCTACGACAAAGAAGAGATCAGAAGAATTTGG	*360*
**NP 245467 Method S**	*301*	GATGGCAAATGGATGAGAGAGTTGATCCTCTACGACAAAGAAGAGATCAGAAGAATTTGG	*360*
**NP 245467 Method E**	*301*	GATGGCAAATGGATGAGAGAGTTGATCCTCTACGACAAAGAAGAGATCAGAAGAATTTGG	*360*
**NP 245467 method K**	*301*	GATGGCAAATGGATGAGAGAGTTGATCCTCTACGACAAAGAAGAGATCAGAAGAATTTGG	*360*
**NP 245467 Method N**	*301*	GATGGCAAATGGATGAGAGAGTTGATCCTCTACGACAAAGAAGAGATCAGAAGAATTTGG	*360*

**NP 245467 MiSeq**	*361*	CGTCAAGCTAATAATGGAGAGGATGCAACTGCTGGTCTCACTCATTTGATGATTTGGCAT	*420*
**NP 245467 Method A**	*361*	CGTCAAGCTAATAATGGAGAGGATGCAACTGCTGGTCTCACTCATTTGATGATTTGGCAT	*420*
**NP 245467 Method S**	*361*	CGTCAAGCTAATAATGGAGAGGATGCAACTGCTGGTCTCACTCATTTGATGATTTGGCAT	*420*
**NP 245467 Method E**	*361*	CGTCAAGCTAATAATGGAGAGGATGCAACTGCTGGTCTCACTCATTTGATGATTTGGCAT	*420*
**NP 245467 method K**	*361*	CGTCAAGCTAATAATGGAGAGGATGCAACTGCTGGTCTCACTCATTTGATGATTTGGCAT	*420*
**NP 245467 Method N**	*361*	CGTCAAGCTAATAATGGAGAGGATGCAACTGCTGGTCTCACTCATTTGATGATTTGGCAT	*420*

**NP 245467 MiSeq**	*421*	TCCAATTTGAATGATGCCACATACCAGAGAACAAGGGCACTTGTGCGTACTGGAATGGAC	*480*
**NP 245467 Method A**	*421*	TCCAATTTGAATGATGCCACATACCAGAGAACAAGGGCACTTGTGCGTACTGGAATGGAC	*480*
**NP 245467 Method S**	*421*	TCCAATCTGAATGATGCCACATACCAGAGAACAAGGGCACTTGTGCGTACTGGAATGGAT	*480*
**NP 245467 Method E**	*421*	TCCAATTTGAATGATGCCACATACCAGAGAACAAGGGCACTTGTGCGTACTGGAATGGAC	*480*
**NP 245467 method K**	*421*	TCCAATTTGAATGATGCCACATACCAGAGAACAAGGGCACTTGTGCGTACTGGAATGGAC	*480*
**NP 245467 Method N**	*421*	TCCAATTTGAATGATGCCACATACCAGAGAACAAGGGCACTTGTGCGTACTGGAATGGAC	*480*

**NP 245467 MiSeq**	*481*	CCTAGGATGTGCTCTCTGATGCAAGGCTCAACCCTCCCTAGGAGATCCGGGGCTGCTGGA	*540*
**NP 245467 Method A**	*481*	CCTAGGATGTGCTCTCTGATGCAAGGCTCAACCCTCCCTAGGAGATCCGGGGCTGCTGGA	*540*
**NP 245467 Method S**	*481*	CCTAGGATGTGCTCTCTGATGCAAGGCTCAACCCTCCCTAGGAGATCCGGGGCTGCTGGA	*540*
**NP 245467 Method E**	*481*	CCTAGGATGTGCTCTCTGATGCAAGGCTCAACCCTCCCTAGGAGATCCGGGGCTGCTGGA	*540*
**NP 245467 method K**	*481*	CCTAGGATGTGCTCTCTGATGCAAGGCTCAACCCTCCCTAGGAGATCCGGGGCTGCTGGA	*540*
**NP 245467 Method N**	*481*	CCTAGGATGTGCTCTCTGATGCAAGGCTCAACCCTCCCTAGGAGATCCGGGGCTGCTGGA	*540*

**NP 245467 MiSeq**	*541*	GCAGCCGTGAAAGGAGTTGGAACAATGGTGATGGAATTGATTCGGATGATCAAACGAGGG	*600*
**NP 245467 Method A**	*541*	GCAGCCGTGAAAGGAGTTGGAACAATGGTGATGGAATTGATTCGGATGATCAAACGAGGG	*600*
**NP 245467 Method S**	*541*	GCAGCAGTGAAAGGAGTTGGAACAATGGTGATGGAATTGATTCGGATGATCAAACGAGGG	*600*
**NP 245467 Method E**	*541*	GCAGCCGTGAAAGGAGTTGGAACAATGGTGATGGAATTGATTCGGATGATCAAACGAGGG	*600*
**NP 245467 method K**	*541*	GCAGCCGTGAAAGGAGTTGGAACAATGGTGATGGAATTGATTCGGATGATCAAACGAGGG	*600*
**NP 245467 Method N**	*541*	GCAGCCGTGAAAGGAGTTGGAACAATGGTGATGGAATTGATTCGGATGATCAAACGAGGG	*600*

**NP 245467 MiSeq**	*601*	ATCAATGATCGGAATTTCTGGAGAGGCGAAAATGGACGGAGAACCAGGATTGCCTACGAG	*660*
**NP 245467 Method A**	*601*	ATCAATGATCGGAATTTCTGGAGAGGCGAAAATGGACGGAGAACCAGGATTGCCTACGAG	*660*
**NP 245467 Method S**	*601*	ATCAATGATCGGAATTTCTGGAGAGGCGAAAACGGACGGAGAACCAGGATTGCCTACGAG	*660*
**NP 245467 Method E**	*601*	ATCAATGATCGGAATTTCTGGAGAGGCGAAAATGGACGGAGAACCAGGATTGCCTACGAG	*660*
**NP 245467 method K**	*601*	ATCAATGATCGGAATTTCTGGAGAGGCGAAAATGGACGGAGAACCAGGATTGCCTACGAG	*660*
**NP 245467 Method N**	*601*	ATCAATGATCGGAATTTCTGGAGAGGCGAAAATGGACGGAGAACCAGGATTGCCTACGAG	*660*

**NP 245467 MiSeq**	*661*	AGAATGTGCAACATTCTCAAGGGAAAGTTCCAAACAGCAGCACAACGAGCAATGATGGAC	*720*
**NP 245467 Method A**	*661*	AGAATGTGCAACATTCTCAAGGGAAAGTTCCAAACAGCAGCACAACGAGCAATGATGGAC	*720*
**NP 245467 Method S**	*661*	AGAATGTGCAACATCCTCAAGGGAAAGTTCCAAACAGCAGCACAACGAGCAATGATGGAC	*720*
**NP 245467 Method E**	*661*	AGAATGTGCAACATTCTCAAGGGAAAGTTCCAAACAGCAGCACAACGAGCAATGATGGAC	*720*
**NP 245467 method K**	*661*	AGAATGTGCAACATTCTCAAGGGAAAGTTCCAAACAGCAGCACAACGAGCAATGATGGAC	*720*
**NP 245467 Method N**	*661*	AGAATGTGCAACATTCTCAAGGGAAAGTTCCAAACAGCAGCACAACGAGCAATGATGGAC	*720*

**NP 245467 MiSeq**	*721*	CAAGTGAGGGAAAGCCGGAATCCTGGGAATGCTGAGATTGAAGATCTCATCTTTCTCGCA	*780*
**NP 245467 Method A**	*721*	CAAGTGAGGGAAAGCCGGAATCCTGGGAATGCTGAGATTGAAGATCTCATCTTTCTCGCA	*780*
**NP 245467 Method S**	*721*	CAAGTGAGGGAAAGCCGGAATCCTGGGAATGCTGAAATTGAAGATCTCATCTTTCTCGCA	*780*
**NP 245467 Method E**	*721*	CAAGTGAGGGAAAGCCGGAATCCTGGGAATGCTGAGATTGAAGATCTCATCTTTCTCGCA	*780*
**NP 245467 method K**	*721*	CAAGTGAGGGAAAGCCGGAATCCTGGGAATGCTGAGATTGAAGATCTCATCTTTCTCGCA	*780*
**NP 245467 Method N**	*721*	CAAGTGAGGGAAAGCCGGAATCCTGGGAATGCTGAGATTGAAGATCTCATCTTTCTCGCA	*780*

**NP 245467 MiSeq**	*781*	CGATCTGCTCTCATTCTGAGGGGATCAGTGGCTCACAAGTCCTGTCTGCCTGCTTGCGTG	*840*
**NP 245467 Method A**	*781*	CGATCTGCTCTCATTCTGAGGGGATCAGTGGCTCACAAGTCCTGTCTGCCTGCTTGCGTG	*840*
**NP 245467 Method S**	*781*	CGATCTGCTCTAATCCTGAGGGGATCAGTGGCTCATAAGTCCTGTCTGCCTGCTTGCGTG	*840*
**NP 245467 Method E**	*781*	CGATCTGCTCTCATTCTGAGGGGATCAGTGGCTCACAAGTCCTGTCTGCCTGCTTGCGTG	*840*
**NP 245467 method K**	*781*	CGATCTGCTCTCATTCTGAGGGGATCAGTGGCTCACAAGTCCTGTCTGCCTGCTTGCGTG	*840*
**NP 245467 Method N**	*781*	CGATCTGCTCTCATTCTGAGGGGATCAGTGGCTCACAAGTCCTGTCTGCCTGCTTGCGTG	*840*

**NP 245467 MiSeq**	*841*	TATGGACTTGCTGTAGCCAGTGGATATGACTTTGAAAGAGAAGGATACTCTCTAGTCGGG	*900*
**NP 245467 Method A**	*841*	TATGGACTTGCTGTAGCCAGTGGATATGACTTTGAAAGAGAAGGATACTCTCTAGTCGGG	*900*
**NP 245467 Method S**	*841*	TATGGACTTGCTGTAGCCAGTGGATATGACTTTGAAAGGGAGGGATACTCTCTAGTCGGA	*900*
**NP 245467 Method E**	*841*	TATGGACTTGCTGTAGCCAGTGGATATGACTTTGAAAGAGAAGGATACTCTCTAGTCGGG	*900*
**NP 245467 method K**	*841*	TATGGACTTGCTGTAGCCAGTGGATATGACTTTGAAAGAGAAGGATACTCTCTAGTCGGG	*900*
**NP 245467 Method N**	*841*	TATGGACTTGCTGTAGCCAGTGGATATGACTTTGAAAGAGAAGGATACTCTCTAGTCGGG	*900*

**NP 245467 MiSeq**	*901*	ATTGATCCTTTCCGTCTACTCCAAAACAGTCAAGTCTTCAGTCTCATCAGACCAAACGAA	*960*
**NP 245467 Method A**	*901*	ATTGATCCTTTCCGTCTACTCCAAAACAGTCAAGTCTTCAGTCTCATCAGACCAAACGAA	*960*
**NP 245467 Method S**	*901*	ATTGATCCTTTCCGTCTGCTCCAAAACAGTCAAGTCTTCAGTCTCATCAGACCGAACGAA	*960*
**NP 245467 Method E**	*901*	ATTGATCCTTTCCGTCTACTCCAAAACAGTCAAGTCTTCAGTCTCATCAGACCAAACGAA	*960*
**NP 245467 method K**	*901*	ATTGATCCTTTCCGTCTACTCCAAAACAGTCAAGTCTTCAGTCTCATCAGACCAAACGAA	*960*
**NP 245467 Method N**	*901*	ATTGATCCTTTCCGTCTACTCCAAAACAGTCAAGTCTTCAGTCTCATCAGACCAAACGAA	*960*

**NP 245467 MiSeq**	*961*	AATCCAGCTCATAAAAGTCAGCTGGTATGGATGGCATGCCACTCTGCAGCATTCGAGGAT	*1020*
**NP 245467 Method A**	*961*	AATCCAGCTCATAAAAGTCAGCTGGTATGGATGGCATGCCACTCTGCAGCATTCGAGGAT	*1020*
**NP 245467 Method S**	*961*	AATCCAGCTCATAAAAGTCAGCTGGTATGGATGGCATGCCACTCTGCGGCATTTGAGGAT	*1020*
**NP 245467 Method E**	*961*	AATCCAGCTCATAAAAGTCAGCTGGTATGGATGGCATGCCACTCTGCAGCATTCGAGGAT	*1020*
**NP 245467 method K**	*961*	AATCCAGCTCATAAAAGTCAGCTGGTATGGATGGCATGCCACTCTGCAGCATTCGAGGAT	*1020*
**NP 245467 Method N**	*961*	AATCCAGCTCATAAAAGTCAGCTGGTATGGATGGCATGCCACTCTGCAGCATTCGAGGAT	*1020*

**NP 245467 MiSeq**	*1021*	CTGAGAGTGTCAAGCTTCATCAGAGGGACAAGAGTAGTCCCAAGAGGACAACTGTCCACC	*1080*
**NP 245467 Method A**	*1021*	CTGAGAGTGTCAAGCTTCATCAGAGGGACAAGAGTAGTCCCAAGAGGACAACTGTCCACC	*1080*
**NP 245467 Method S**	*1021*	CTGAGAGTGTCAAGCTTCATCAGAGGGACAAGAGTAGTCCCAAGAGGACAACTGTCCACC	*1080*
**NP 245467 Method E**	*1021*	CTGAGAGTGTCAAGCTTCATCAGAGGGACAAGAGTAGTCCCAAGAGGACAACTGTCCACC	*1080*
**NP 245467 method K**	*1021*	CTGAGAGTGTCAAGCTTCATCAGAGGGACAAGAGTAGTCCCAAGAGGACAACTGTCCACC	*1080*
**NP 245467 Method N**	*1021*	CTGAGAGTGTCAAGCTTCATCAGAGGGACAAGAGTAGTCCCAAGAGGACAACTGTCCACC	*1080*

**NP 245467 MiSeq**	*1081*	AGAGGAGTTCAGATTGCTTCAAATGAAAACATGGATACGATGGACTCCAATACTCTTGAA	*1140*
**NP 245467 Method A**	*1081*	AGAGGAGTTCAGATTGCTTCAAATGAAAACATGGATACGATGGACTCCAATACTCTTGAA	*1140*
**NP 245467 Method S**	*1081*	AGAGGAGTTCAGATTGCTTCAAATGAAAACATGGAGACAATGGACTCCAGTACTCTTGAA	*1140*
**NP 245467 Method E**	*1081*	AGAGGAGTTCAGATTGCTTCAAATGAAAACATGGATACGATGGACTCCAATACTCTTGAA	*1140*
**NP 245467 method K**	*1081*	AGAGGAGTTCAGATTGCTTCAAATGAAAACATGGATACGATGGACTCCAATACTCTTGAA	*1140*
**NP 245467 Method N**	*1081*	AGAGGAGTTCAGATTGCTTCAAATGAAAACATGGATACGATGGACTCCAATACTCTTGAA	*1140*

**NP 245467 MiSeq**	*1141*	CTGAGGAGCAGATACTGGGCTATAAGAACAAGAAGTGGAGGAAACACTAACCAACAGAGA	*1200*
**NP 245467 Method A**	*1141*	CTGAGGAGCAGATACTGGGCTATAAGAACAAGAAGTGGAGGAAACACTAACCAACAGAGA	*1200*
**NP 245467 Method S**	*1141*	CTGAGGAGCAGATACTGGGCTATAAGAACAAGAAGTGGAGGAAACACTAACCAACAGAGA	*1200*
**NP 245467 Method E**	*1141*	CTGAGGAGCAGATACTGGGCTATAAGAACAAGAAGTGGAGGAAACACTAACCAACAGAGA	*1200*
**NP 245467 method K**	*1141*	CTGAGGAGCAGATACTGGGCTATAAGAACAAGAAGTGGAGGAAACACTAACCAACAGAGA	*1200*
**NP 245467 Method N**	*1141*	CTGAGGAGCAGATACTGGGCTATAAGAACAAGAAGTGGAGGAAACACTAACCAACAGAGA	*1200*

NP 245467 MiSeq	1201	GCATCTGCAGGACAAATCAGCGTACAGCCCACATTCTCTGTGCAGAGAAACCTCCCATTC	1260
NP 245467 Method A	1201	GCATCTGCAGGACAAATCAGCGTACAGCCCACATTCTCTGTGCAGAGAAACCTCCCATTC	1260
NP 245467 Method S	1201	GCATCTGCAGGACAAATCAGCGTACAGCCCACATTCTCTGTGCAGAGAAACCTCCCATTC	1260
NP 245467 Method E	1201	GCATCTGCAGGACAAATCAGCGTACAGCCCACATTCTCTGTGCAGAGAAACCTCCCATTC	1260
NP 245467 method K	1201	GCATCTGCAGGACAAATCAGCGTACAGCCCACATTCTCTGTGCAGAGAAACCTCCCATTC	1260
NP 245467 Method N	1201	GCATCTGCAGGACAAATCAGCGTACAGCCCACATTCTCTGTGCAGAGAAACCTCCCATTC	1260

NP 245467 MiSeq	1261	GAGAGAGCAACCATCATGGCAGCATTTACGGGAAACACTGAAGGCAGAACTTCAGACATG	1320
NP 245467 Method A	1261	GAGAGAGCAACCATCATGGCAGCATTTACGGGAAACACTGAAGGCAGAACTTCAGACATG	1320
NP 245467 Method S	1261	GAGAGAGCAACCATCATGGCAGCATTTACGGGAAACACTGAAGGCAGAACTTCAGACATG	1320
NP 245467 Method E	1261	GAGAGAGCAACCATCATGGCAGCATTTACGGGAAACACTGAAGGCAGAACTTCAGACATG	1320
NP 245467 method K	1261	GAGAGAGCAACCATCATGGCAGCATTTACGGGAAACACTGAAGGCAGAACTTCAGACATG	1320
NP 245467 Method N	1261	GAGAGAGCAACCATCATGGCAGCATTTACGGGAAACACTGAAGGCAGAACTTCAGACATG	1320

NP 245467 MiSeq	1321	AGAACTGAGATCATAAGGATGATGGAAAATGCCAGACCTGAAGATGTGTCTTTCCAGGGG	1380
NP 245467 Method A	1321	AGAACTGAGATCATAAGGATGATGGAAAATGCCAGACCTGAAGATGTGTCTTTCCAGGGG	1380
NP 245467 Method S	1321	AGAACTGAGATCATAAGGATGATGGAAAATGCCAGACCTGAAGATGTGTCTTTCCAGGGG	1380
NP 245467 Method E	1321	AGAACTGAGATCATAAGGATGATGGAAAATGCCAGACCTGAAGATGTGTCTTTCCAGGGG	1380
NP 245467 method K	1321	AGAACTGAGATCATAAGGATGATGGAAAATGCCAGACCTGAAGATGTGTCTTTCCAGGGG	1380
NP 245467 Method N	1321	AGAACTGAGATCATAAGGATGATGGAAAATGCCAGACCTGAAGATGTGTCTTTCCAGGGG	1380

NP 245467 MiSeq	1381	CGGGGAGTCTTCGAGCTCTCGGACGAAAAGGCAACGAACCCGATCGTGCCTTCCTTTGAC	1440
NP 245467 Method A	1381	CGGGGAGTCTTCGAGCTCTCGGACGAAAAGGCAACGAACCCGATCGTGCCTTCCTTTGAC	1440
NP 245467 Method S	1381	CGGGGAGTCTTCGAGCTCTCGGACGAAAAGGCAACGAACCCGATCGTGCCTTCCTTTGAC	1440
NP 245467 Method E	1381	CGGGGAGTCTTCGAGCTCTCGGACGAAAAGGCAACGAACCCGATCGTGCCTTCCTTTGAC	1440
NP 245467 method K	1381	CGGGGAGTCTTCGAGCTCTCGGACGAAAAGGCAACGAACCCGATCGTGCCTTCCTTTGAC	1440
NP 245467 Method N	1381	CGGGGAGTCTTCGAGCTCTCGGACGAAAAGGCAACGAACCCGATCGTGCCTTCCTTTGAC	1440

**NP 245467 MiSeq**	*1441*	ATGAGCAATGAAGGATCTTATTTCTTCGGAGACAATGCAGAGGAGTATGACAATTAA	*1497*
**NP 245467 Method A**	*1441*	ATGAGCAATGAAGGATCTTATTTCTTCGGAGACAATGCAGAGGAGTATGACAATTAA	*1497*
**NP 245467 Method S**	*1441*	ATGAGCAATGAAGGATCTTATTTCTTCGGAGACAATGCAGAGGAGTATGACAATTAA	*1497*
**NP 245467 Method E**	*1441*	ATGAGCAATGAAGGATCTTATTTCTTCGGAGACAATGCAGAGGAGTATGACAATTAA	*1497*
**NP 245467 method K**	*1441*	ATGAGCAATGAAGGATCTTATTTCTTCGGAGACAATGCAGAGGAGTATGACAATTAA	*1497*
**NP 245467 Method N**	*1441*	ATGAGCAATGAAGGATCTTATTTCTTCGGAGACAATGCAGAGGAGTATGACAATTAA	*1497*
