# Supplementary material for: An Evaluation of Avian Influenza Virus Whole-Genome Sequencing Approaches Using Nanopore Technology
Source: Microorganisms. 2023 Feb 19;11(2):529. doi: 10.3390/microorganisms11020529 (PMC9967579; doi:10.3390/microorganisms11020529)
Supplement: Supplementary file 1 [file microorganisms-11-00529-s001.zip › manuscript.v8 230219 Suppl Figures and Tables/Supplementary Figures S1a-h 245467/Supplementary Figure S1a PB2.pdf]

Formatted Alignments

**PB2 245467 MiSeq**	*1*	ATGGAGAGAATAAGAGAACTAAGAGATCTAATGTCACAGTCTCGCACTCGCGAGATACTC	*60*
**PB2 245467 Method A**	*1*	ATGGAGAGAATAAGAGAACTAAGAGATCTAATGTCACAGTCTCGCACTCGCGAGATACTC	*60*
**PB2 245467 Method S**	*1*	ATGGAGAGAATAA**A**AGAACTAAGAGATCTAATGTCACAGTCTCGCACTCGCGAGATACTC	*60*
**PB2 245467 Method E**	*1*	ATGGAGAGAATAAGAGAACTAAGAGATCTAATGTCACAGTCTCGCACTCGCGAGATACTC	*60*
**PB2 245467 Method K**	*1*	ATGGAGAGAATAAGAGAACTAAGAGATCTAATGTCACAGTCTCGCACTCGCGAGATACTC	*60*
**PB2 245467 Method N**	*1*	ATGGAGAGAATAAGAGAACTAAGAGATCTAATGTCACAGTCTCGCACTCGCGAGATACTC	*60*

**PB2 245467 MiSeq**	*61*	ACCAAAACTACTGTGGACCACATGGCCATAATCAAAAAATACACATCGGGAAGGCAAGAG	*120*
**PB2 245467 Method A**	*61*	ACCAAAACTACTGTGGACCACATGGCCATAATCAAAAAATACACATCGGGAAGGCAAGAG	*120*
**PB2 245467 Method S**	*61*	ACCAAAAC**C**ACTGTGGACCACATGGCCATAATCAAAAAATACACATCGGGAAGGCAAGAG	*120*
**PB2 245467 Method E**	*61*	ACCAAAACTACTGTGGACCACATGGCCATAATCAAAAAATACACATCGGGAAGGCAAGAG	*120*
**PB2 245467 Method K**	*61*	ACCAAAACTACTGTGGACCACATGGCCATAATCAAAAAATACACATCGGGAAGGCAAGAG	*120*
**PB2 245467 Method N**	*61*	ACCAAAACTACTGTGGACCACATGGCCATAATCAAAAAATACACATCGGGAAGGCAAGAG	*120*

**PB2 245467 MiSeq**	*121*	AAGAACCCCGCGCTCAGGATGAAATGGATGATGGCAATGAAATATCCAATCACAGCAGAC	*180*
**PB2 245467 Method A**	*121*	AAGAACCCCGCGCTCAGGATGAAATGGATGATGGCAATGAAATATCCAATCACAGCAGAC	*180*
**PB2 245467 Method S**	*121*	AAGAACCCCGC**A**CTCAGGATGAAATGGATGATGGCAATGAAATA**C**CCAATCACAGCAGAC	*180*
**PB2 245467 Method E**	*121*	AAGAACCCCGCGCTCAGGATGAAATGGATGATGGCAATGAAATATCCAATCACAGCAGAC	*180*
**PB2 245467 Method K**	*121*	AAGAACCCCGCGCTCAGGATGAAATGGATGATGGCAATGAAATATCCAATCACAGCAGAC	*180*
**PB2 245467 Method N**	*121*	AAGAACCCCGCGCTCAGGATGAAATGGATGATGGCAATGAAATATCCAATCACAGCAGAC	*180*

**PB2 245467 MiSeq**	*181*	AAGAGAATAATGGAAATGATTCCTGAAAGAAATGAACAAGGACAAACCCTTTGGAGCAAA	*240*
**PB2 245467 Method A**	*181*	AAGAGAATAATGGAAATGATTCCTGAAAGAAATGAACAAGGACAAACCCTTTGGAGCAAA	*240*
**PB2 245467 Method S**	*181*	AAGAGAATAATGGAAATGATTCCTGAAAGAAATGAACAAGG**G**CAAACCCT**C**TGGAGCAAA	*240*
**PB2 245467 Method E**	*181*	AAGAGAATAATGGAAATGATTCCTGAAAGAAATGAACAAGGACAAACCCTTTGGAGCAAA	*240*
**PB2 245467 Method K**	*181*	AAGAGAATAATGGAAATGATTCCTGAAAGAAATGAACAAGGACAAACCCTTTGGAGCAAA	*240*
**PB2 245467 Method N**	*181*	AAGAGAATAATGGAAATGATTCCTGAAAGAAATGAACAAGGACAAACCCTTTGGAGCAAA	*240*

**PB2 245467 MiSeq**	*241*	ACGAACGATGCCGGGTCTGACCGAGTGATGGTATCACCTCTGGCCGTGACATGGTGGAAT	*300*
**PB2 245467 Method A**	*241*	ACGAACGATGCCGGGTCTGACCGAGTGATGGTATCACCTCTGGCCGTGACATGGTGGAAT	*300*
**PB2 245467 Method S**	*241*	ACGAACGATGCCGGGTCAGACCGAGTGATGGTATCACCTCTGGCTGTGACATGGTGGAAT	*300*
**PB2 245467 Method E**	*241*	ACGAACGATGCCGGGTCTGACCGAGTGATGGTATCACCTCTGGCCGTGACATGGTGGAAT	*300*
**PB2 245467 Method K**	*241*	ACGAACGATGCCGGGTCTGACCGAGTGATGGTATCACCTCTGGCCGTGACATGGTGGAAT	*300*
**PB2 245467 Method N**	*241*	ACGAACGATGCCGGGTCTGACCGAGTGATGGTATCACCTCTGGCCGTGACATGGTGGAAT	*300*

**PB2 245467 MiSeq**	*301*	AGGAACGGACCAACAACAAGTACGGTCCACTACCCAAAGGTATACAAAACGTATTTCGAA	*360*
**PB2 245467 Method A**	*301*	AGGAACGGACCAACAACAAGTACGGTCCACTACCCAAAGGTATACAAAACGTATTTCGAA	*360*
**PB2 245467 Method S**	*301*	AGGAATGGACCAACAACAAGTACAGTCCACTACCCAAAGGTATACAAAACTTATTTCGAA	*360*
**PB2 245467 Method E**	*301*	AGGAACGGACCAACAACAAGTACGGTCCACTACCCAAAGGTATACAAAACGTATTTCGAA	*360*
**PB2 245467 Method K**	*301*	AGGAACGGACCAACAACAAGTACGGTCCACTACCCAAAGGTATACAAAACGTATTTCGAA	*360*
**PB2 245467 Method N**	*301*	AGGAACGGACCAACAACAAGTACGGTCCACTACCCAAAGGTATACAAAACGTATTTCGAA	*360*

**PB2 245467 MiSeq**	*361*	AAAGTTGAAAGATTGAAACATGGGACCTTTGGTCCTGTTCACTTCAGAAATCAAGTTAAG	*420*
**PB2 245467 Method A**	*361*	AAAGTTGAAAGATTGAAACATGGGACCTTTGGTCCTGTTCACTTCAGAAATCAAGTTAAG	*420*
**PB2 245467 Method S**	*361*	AAAGTTGAAAGGTTGAAACACGGGACCTTTGGCCCTGTCCACTTCAGAAATCAAGTTAAG	*420*
**PB2 245467 Method E**	*361*	AAAGTTGAAAGATTGAAACATGGGACCTTTGGTCCTGTTCACTTCAGAAATCAAGTTAAG	*420*
**PB2 245467 Method K**	*361*	AAAGTTGAAAGATTGAAACATGGGACCTTTGGTCCTGTTCACTTCAGAAATCAAGTTAAG	*420*
**PB2 245467 Method N**	*361*	AAAGTTGAAAGATTGAAACATGGGACCTTTGGTCCTGTTCACTTCAGAAATCAAGTTAAG	*420*

**PB2 245467 MiSeq**	*421*	ATAAGACGGAGAGTCGACATAAACCCAGGCCATGCAGACCTCAGTGCCAAAGAGGCGCAG	*480*
**PB2 245467 Method A**	*421*	ATAAGACGGAGAGTCGACATAAACCCAGGCCATGCAGACCTCAGTGCCAAAGAGGCGCAG	*480*
**PB2 245467 Method S**	*421*	ATAAGACGGAGGGTCGACATAAACCCAGGCCATGCAGACCTCAGTGCCAAAGAGGCGCAG	*480*
**PB2 245467 Method E**	*421*	ATAAGACGGAGAGTCGACATAAACCCAGGCCATGCAGACCTCAGTGCCAAAGAGGCGCAG	*480*
**PB2 245467 Method K**	*421*	ATAAGACGGAGAGTCGACATAAACCCAGGCCATGCAGACCTCAGTGCCAAAGAGGCGCAG	*480*
**PB2 245467 Method N**	*421*	ATAAGACGGAGAGTCGACATAAACCCAGGCCATGCAGACCTCAGTGCCAAAGAGGCGCAG	*480*

**PB2 245467 MiSeq**	*481*	GATGTAATCATGGAAGTTGTCTTCCCAAATGAAGTGGGAGCGAGAATACTAACATCGGAG	*540*
**PB2 245467 Method A**	*481*	GATGTAATCATGGAAGTTGTCTTCCCAAATGAAGTGGGAGCGAGAATACTAACATCGGAG	*540*
**PB2 245467 Method S**	*481*	GATGTAATCATGGAAGTTGTCTTCCCAAATGAAGTGGGAGCGAGAATACTAACATCGGAG	*540*
**PB2 245467 Method E**	*481*	GATGTAATCATGGAAGTTGTCTTCCCAAATGAAGTGGGAGCGAGAATACTAACATCGGAG	*540*
**PB2 245467 Method K**	*481*	GATGTAATCATGGAAGTTGTCTTCCCAAATGAAGTGGGAGCGAGAATACTAACATCGGAG	*540*
**PB2 245467 Method N**	*481*	GATGTAATCATGGAAGTTGTCTTCCCAAATGAAGTGGGAGCGAGAATACTAACATCGGAG	*540*

**PB2 245467 MiSeq**	*541*	TCACAACTGACGATAACAAAGGAGAAGAAGGAAGAACTCCAAGACTGCAAAATTGCACCT	*600*
**PB2 245467 Method A**	*541*	TCACAACTGACGATAACAAAGGAGAAGAAGGAAGAACTCCAAGACTGCAAAATTGCACCT	*600*
**PB2 245467 Method S**	*541*	TCACAACTGACGATAACAAAGGAGAAGAAGGAAGAACTCCAGGACTGCAAAATCGCCCCT	*600*
**PB2 245467 Method E**	*541*	TCACAACTGACGATAACAAAGGAGAAGAAGGAAGAACTCCAAGACTGCAAAATTGCACCT	*600*
**PB2 245467 Method K**	*541*	TCACAACTGACGATAACAAAGGAGAAGAAGGAAGAACTCCAAGACTGCAAAATTGCACCT	*600*
**PB2 245467 Method N**	*541*	TCACAACTGACGATAACAAAGGAGAAGAAGGAAGAACTCCAAGACTGCAAAATTGCACCT	*600*
**PB2 245467 MiSeq**	*601*	TTGATGGTCGCATACATGCTAGAAAGAGAGTTAGTCCGCAAGACGAGGTTCCTCCCAGTG	*660*
**PB2 245467 Method A**	*601*	TTGATGGTCGCATACATGCTAGAAAGAGAGTTAGTCCGCAAGACGAGGTTCCTCCCAGTG	*660*
**PB2 245467 Method S**	*601*	TTGATGGTCGCATACATGCTAGAAAGAGAGTTAGTCCGCAAGACGAGGTTCCTCCCAGTG	*660*
**PB2 245467 Method E**	*601*	TTGATGGTCGCATACATGCTAGAAAGAGAGTTAGTCCGCAAGACGAGGTTCCTCCCAGTG	*660*
**PB2 245467 Method K**	*601*	TTGATGGTCGCATACATGCTAGAAAGAGAGTTAGTCCGCAAGACGAGGTTCCTCCCAGTG	*660*
**PB2 245467 Method N**	*601*	TTGATGGTCGCATACATGCTAGAAAGAGAGTTAGTCCGCAAGACGAGGTTCCTCCCAGTG	*660*
**PB2 245467 MiSeq**	*661*	GCTGGTGGAACAAGCAGTGTCTATATTGAGGTGCTGCATTTAACCCAGGGGACATGCTGG	*720*
**PB2 245467 Method A**	*661*	GCTGGTGGAACAAGCAGTGTCTATATTGAGGTGCTGCATTTAACCCAGGGGACATGCTGG	*720*
**PB2 245467 Method S**	*661*	GCTGGTGGAACAAGCAGTGTCTATATTGAGGTGCTGCATTTAACCCAGGGGACATGCTGG	*720*
**PB2 245467 Method E**	*661*	GCTGGTGGAACAAGCAGTGTCTATATTGAGGTGCTGCATTTAACCCAGGGGACATGCTGG	*720*
**PB2 245467 Method K**	*661*	GCTGGTGGAACAAGCAGTGTCTATATTGAGGTGCTGCATTTAACCCAGGGGACATGCTGG	*720*
**PB2 245467 Method N**	*661*	GCTGGTGGAACAAGCAGTGTCTATATTGAGGTGCTGCATTTAACCCAGGGGACATGCTGG	*720*
**PB2 245467 MiSeq**	*721*	GAGCAGATGTACACTCCAGGAGGAGAAGTGAGAAATGATGATGTAGACCAGAGTTTGATT	*780*
**PB2 245467 Method A**	*721*	GAGCAGATGTACACTCCAGGAGGAGAAGTGAGAAATGATGATGTAGACCAGAGTTTGATT	*780*
**PB2 245467 Method S**	*721*	GAGCAGATGTACACTCCAGGAGGAGAAGTGAGAAATGATGATGTAGACCAGAGTTTGATT	*780*
**PB2 245467 Method E**	*721*	GAGCAGATGTACACTCCAGGAGGAGAAGTGAGAAATGATGATGTAGACCAGAGTTTGATT	*780*
**PB2 245467 Method K**	*721*	GAGCAGATGTACACTCCAGGAGGAGAAGTGAGAAATGATGATGTAGACCAGAGTTTGATT	*780*
**PB2 245467 Method N**	*721*	GAGCAGATGTACACTCCAGGAGGAGAAGTGAGAAATGATGATGTAGACCAGAGTTTGATT	*780*
**PB2 245467 MiSeq**	*781*	ATCGCCGCCAGGAACATAGTAAGAAGAGCAACAGTATCAGCAGATCCATTAGCATCTCTA	*840*
**PB2 245467 Method A**	*781*	ATCGCCGCCAGGAACATAGTAAGAAGAGCAACAGTATCAGCAGATCCATTAGCATCTCTA	*840*
**PB2 245467 Method S**	*781*	ATCGCTGCCAGAAACATAGTAAGAAGAGCAACAGTATCAGCAGACCCACTAGCATCTCTA	*840*
**PB2 245467 Method E**	*781*	ATCGCCGCCAGGAACATAGTAAGAAGAGCAACAGTATCAGCAGATCCATTAGCATCTCTA	*840*
**PB2 245467 Method K**	*781*	ATCGCCGCCAGGAACATAGTAAGAAGAGCAACAGTATCAGCAGATCCATTAGCATCTCTA	*840*
**PB2 245467 Method N**	*781*	ATCGCCGCCAGGAACATAGTAAGAAGAGCAACAGTATCAGCAGATCCATTAGCATCTCTA	*840*

**PB2 245467 MiSeq**	*841*	TTGGAGATGTGCCACAGCACACAGATTGGGGGAATAAGGATGGTCGACATTCTTCGACAA	*900*
**PB2 245467 Method A**	*841*	TTGGAGATGTGCCACAGCACACAGATTGGGGGAATAAGGATGGTCGACATTCTTCGACAA	*900*
**PB2 245467 Method S**	*841*	TTGGAGATGTGCCACAGCACACAGATTGGGGGAATAAGGATGGTCGACATCCTTCGGCAA	*900*
**PB2 245467 Method E**	*841*	TTGGAGATGTGCCACAGCACACAGATTGGGGGAATAAGGATGGTCGACATTCTTCGACAA	*900*
**PB2 245467 Method K**	*841*	TTGGAGATGTGCCACAGCACACAGATTGGGGGAATAAGGATGGTCGACATTCTTCGACAA	*900*
**PB2 245467 Method N**	*841*	TTGGAGATGTGCCACAGCACACAGATTGGGGGAATAAGGATGGTCGACATTCTTCGACAA	*900*

**PB2 245467 MiSeq**	*901*	AATCCAACAGAGGAACAAGCCGTGGATATATGCAAGGCAGCAATGGGCTTGAGGATTAGC	*960*
**PB2 245467 Method A**	*901*	AATCCAACAGAGGAACAAGCCGTGGATATATGCAAGGCAGCAATGGGCTTGAGGATTAGC	*960*
**PB2 245467 Method S**	*901*	AATCCAACAGAGGAACAAGCCGTGGACATATGCAAGGCAGCAATGGGCTTGAGGATTAGC	*960*
**PB2 245467 Method E**	*901*	AATCCAACAGAGGAACAAGCCGTGGATATATGCAAGGCAGCAATGGGCTTGAGGATTAGC	*960*
**PB2 245467 Method K**	*901*	AATCCAACAGAGGAACAAGCCGTGGATATATGCAAGGCAGCAATGGGCTTGAGGATTAGC	*960*
**PB2 245467 Method N**	*901*	AATCCAACAGAGGAACAAGCCGTGGATATATGCAAGGCAGCAATGGGCTTGAGGATTAGC	*960*

**PB2 245467 MiSeq**	*961*	TCATCTTTCAGCTTTGGTGGATTCACTTTCAAAAGAACAAGTGGATCATCAGTCAAAAGA	*1020*
**PB2 245467 Method A**	*961*	TCATCTTTCAGCTTTGGTGGATTCACTTTCAAAAGAACAAGTGGATCATCAGTCAAAAGA	*1020*
**PB2 245467 Method S**	*961*	TCATCTTTCAGCTTTGGTGGATTCACTTTTAAAAGAACAAGTGGATCATCAGTCAAAAGA	*1020*
**PB2 245467 Method E**	*961*	TCATCTTTCAGCTTTGGTGGATTCACTTTCAAAAGAACAAGTGGATCATCAGTCAAAAGA	*1020*
**PB2 245467 Method K**	*961*	TCATCTTTCAGCTTTGGTGGATTCACTTTCAAAAGAACAAGTGGATCATCAGTCAAAAGA	*1020*
**PB2 245467 Method N**	*961*	TCATCTTTCAGCTTTGGTGGATTCACTTTCAAAAGAACAAGTGGATCATCAGTCAAAAGA	*1020*

**PB2 245467 MiSeq**	*1021*	GAAGAAGAAGTGCTTACGGGCAACCTTCAAACACTGAAAATAAGAGTACATGAGGGGTAT	*1080*
**PB2 245467 Method A**	*1021*	GAAGAAGAAGTGCTTACGGGCAACCTTCAAACACTGAAAATAAGAGTACATGAGGGGTAT	*1080*
**PB2 245467 Method S**	*1021*	GAAGAAGAAGTGCTTACGGGCAACCTTCAAACATTGAAAATAAGAGTACATGAGGGGTAT	*1080*
**PB2 245467 Method E**	*1021*	GAAGAAGAAGTGCTTACGGGCAACCTTCAAACACTGAAAATAAGAGTACATGAGGGGTAT	*1080*
**PB2 245467 Method K**	*1021*	GAAGAAGAAGTGCTTACGGGCAACCTTCAAACACTGAAAATAAGAGTACATGAGGGGTAT	*1080*
**PB2 245467 Method N**	*1021*	GAAGAAGAAGTGCTTACGGGCAACCTTCAAACACTGAAAATAAGAGTACATGAGGGGTAT	*1080*

**PB2 245467 MiSeq**	*1081*	GAAGAGTTCACGATGGTTGGAAGAAGAGCAACGGCTATTCTCAGGAAGGCAACCAGAAGG	*1140*
**PB2 245467 Method A**	*1081*	GAAGAGTTCACGATGGTTGGAAGAAGAGCAACGGCTATTCTCAGGAAGGCAACCAGAAGG	*1140*
**PB2 245467 Method S**	*1081*	GAAGAGTTCACAATGGTTGGGAGAAGAGCAACGGCTATTCTCAGGAAGGCAACCAGAAGG	*1140*
**PB2 245467 Method E**	*1081*	GAAGAGTTCACGATGGTTGGAAGAAGAGCAACGGCTATTCTCAGGAAGGCAACCAGAAGG	*1140*
**PB2 245467 Method K**	*1081*	GAAGAGTTCACGATGGTTGGAAGAAGAGCAACGGCTATTCTCAGGAAGGCAACCAGAAGG	*1140*
**PB2 245467 Method N**	*1081*	GAAGAGTTCACGATGGTTGGAAGAAGAGCAACGGCTATTCTCAGGAAGGCAACCAGAAGG	*1140*

**PB2 245467 MiSeq**	*1141*	TTGATCCAGCTAATAGTAAGTGGAAGAGACGAGCAGTCAATTGCTGAAGCAATAATTGTA	*1200*
**PB2 245467 Method A**	*1141*	TTGATCCAGCTAATAGTAAGTGGAAGAGACGAGCAGTCAATTGCTGAAGCAATAATTGTA	*1200*
**PB2 245467 Method S**	*1141*	TTGATCCAGCTAATAGTAAGTGGAAGAGACGAGCAGTCAATTGCTGAAGCAATAATTGTG	*1200*
**PB2 245467 Method E**	*1141*	TTGATCCAGCTAATAGTAAGTGGAAGAGACGAGCAGTCAATTGCTGAAGCAATAATTGTA	*1200*
**PB2 245467 Method K**	*1141*	TTGATCCAGCTAATAGTAAGTGGAAGAGACGAGCAGTCAATTGCTGAAGCAATAATTGTA	*1200*
**PB2 245467 Method N**	*1141*	TTGATCCAGCTAATAGTAAGTGGAAGAGACGAGCAGTCAATTGCTGAAGCAATAATTGTA	*1200*
**PB2 245467 MiSeq**	*1201*	GCCATGGTATTCTCACAGGAAGACTGCATGATCAAGGCAGTTCGAGGTGATCTGAATTTT	*1260*
**PB2 245467 Method A**	*1201*	GCCATGGTATTCTCACAGGAAGACTGCATGATCAAGGCAGTTCGAGGTGATCTGAATTTT	*1260*
**PB2 245467 Method S**	*1201*	GCCATGGTATTCTCGCAAGAAGACTGCATGATCAAGGCAGTTCGAGGTGATCTGAATTTT	*1260*
**PB2 245467 Method E**	*1201*	GCCATGGTATTCTCACAGGAAGACTGCATGATCAAGGCAGTTCGAGGTGATCTGAATTTT	*1260*
**PB2 245467 Method K**	*1201*	GCCATGGTATTCTCACAGGAAGACTGCATGATCAAGGCAGTTCGAGGTGATCTGAATTTT	*1260*
**PB2 245467 Method N**	*1201*	GCCATGGTATTCTCACAGGAAGACTGCATGATCAAGGCAGTTCGAGGTGATCTGAATTTT	*1260*
**PB2 245467 MiSeq**	*1261*	GTCAATAGGGCGAACCAGCGGCTGAATCCAATGCATCAACTCTTGAGACACTTCCAAAAG	*1320*
**PB2 245467 Method A**	*1261*	GTCAATAGGGCGAACCAGCGGCTGAATCCAATGCATCAACTCTTGAGACACTTCCAAAAG	*1320*
**PB2 245467 Method S**	*1261*	GTCAATAGGGCGAACCAGCGGCTGAATCCAATGCATCAACTCTTGAGACACTTCCAAAAG	*1320*
**PB2 245467 Method E**	*1261*	GTCAATAGGGCGAACCAGCGGCTGAATCCAATGCATCAACTCTTGAGACACTTCCAAAAG	*1320*
**PB2 245467 Method K**	*1261*	GTCAATAGGGCGAACCAGCGGCTGAATCCAATGCATCAACTCTTGAGACACTTCCAAAAG	*1320*
**PB2 245467 Method N**	*1261*	GTCAATAGGGCGAACCAGCGGCTGAATCCAATGCATCAACTCTTGAGACACTTCCAAAAG	*1320*
**PB2 245467 MiSeq**	*1321*	GATGCTAAAGTGCTTTTCCAAAACTGGGGAATTGAATCCATTGACAACGTGATGGGGATG	*1380*
**PB2 245467 Method A**	*1321*	GATGCTAAAGTGCTTTTCCAAAACTGGGGAATTGAATCCATTGACAACGTGATGGGGATG	*1380*
**PB2 245467 Method S**	*1321*	GATGCTAAAGTGCTTTTCCAAAACTGGGGAATTGAACCCATTGACAATGTGATGGGGATG	*1380*
**PB2 245467 Method E**	*1321*	GATGCTAAAGTGCTTTTCCAAAACTGGGGAATTGAATCCATTGACAACGTGATGGGGATG	*1380*
**PB2 245467 Method K**	*1321*	GATGCTAAAGTGCTTTTCCAAAACTGGGGAATTGAATCCATTGACAACGTGATGGGGATG	*1380*
**PB2 245467 Method N**	*1321*	GATGCTAAAGTGCTTTTCCAAAACTGGGGAATTGAATCCATTGACAACGTGATGGGGATG	*1380*
**PB2 245467 MiSeq**	*1381*	ATTGGGATATTGCCCGACATGACCCCAAGCACTGAGATGTCACTGAGGGGAATAAGAGTC	*1440*
**PB2 245467 Method A**	*1381*	ATTGGGATATTGCCCGACATGACCCCAAGCACTGAGATGTCACTGAGGGGAATAAGAGTC	*1440*
**PB2 245467 Method S**	*1381*	ATCGGGATATTGCCCGACATGACCCCAAGCACTGAGATGTCACTGAGGGGGATAAGAGTC	*1440*
**PB2 245467 Method E**	*1381*	ATTGGGATATTGCCCGACATGACCCCAAGCACTGAGATGTCACTGAGGGGAATAAGAGTC	*1440*
**PB2 245467 Method K**	*1381*	ATTGGGATATTGCCCGACATGACCCCAAGCACTGAGATGTCACTGAGGGGAATAAGAGTC	*1440*
**PB2 245467 Method N**	*1381*	ATTGGGATATTGCCCGACATGACCCCAAGCACTGAGATGTCACTGAGGGGAATAAGAGTC	*1440*

**PB2 245467 MiSeq**	*1441*	AGCAAGATGGGAGTAGATGAATACTCCAGTACAGAGAGGGTGGTGGTGAGCATCGACCGA	*1500*
**PB2 245467 Method A**	*1441*	AGCAAGATGGGAGTAGATGAATACTCCAGTACAGAGAGGGTGGTGGTGAGCATCGACCGA	*1500*
**PB2 245467 Method S**	*1441*	AG**T**AAGATGGGAGTAGATGAATACTCCAGTACAGAGAGGGTGGT**A**GTGAGCATCGACCGA	*1500*
**PB2 245467 Method E**	*1441*	AGCAAGATGGGAGTAGATGAATACTCCAGTACAGAGAGGGTGGTGGTGAGCATCGACCGA	*1500*
**PB2 245467 Method K**	*1441*	AGCAAGATGGGAGTAGATGAATACTCCAGTACAGAGAGGGTGGTGGTGAGCATCGACCGA	*1500*
**PB2 245467 Method N**	*1441*	AGCAAGATGGGAGTAGATGAATACTCCAGTACAGAGAGGGTGGTGGTGAGCATCGACCGA	*1500*

**PB2 245467 MiSeq**	*1501*	TTTTTAAGAGTCCGGGACCAACGAGGGAATGTACTATTGTCACCAGAAGAAGTCAGCGAG	*1560*
**PB2 245467 Method A**	*1501*	TTTTTAAGAGTCCGGGACCAACGAGGGAATGTACTATTGTCACCAGAAGAAGTCAGCGAG	*1560*
**PB2 245467 Method S**	*1501*	TTTTTAAGAGT**T**CGGGACCAACGAGGGAA**C**GTACTATTGTCACC**T**GAAGAAGTCAGCGAG	*1560*
**PB2 245467 Method E**	*1501*	TTTTTAAGAGTCCGGGACCAACGAGGGAATGTACTATTGTCACCAGAAGAAGTCAGCGAG	*1560*
**PB2 245467 Method K**	*1501*	TTTTTAAGAGTCCGGGACCAACGAGGGAATGTACTATTGTCACCAGAAGAAGTCAGCGAG	*1560*
**PB2 245467 Method N**	*1501*	TTTTTAAGAGTCCGGGACCAACGAGGGAATGTACTATTGTCACCAGAAGAAGTCAGCGAG	*1560*

**PB2 245467 MiSeq**	*1561*	ACACAAGGAACAGAGAAATTGACAATCACTTATTCATCATCAATGATGTGGGAGATCAAT	*1620*
**PB2 245467 Method A**	*1561*	ACACAAGGAACAGAGAAATTGACAATCACTTATTCATCATCAATGATGTGGGAGATCAAT	*1620*
**PB2 245467 Method S**	*1561*	ACACAAGG**G**ACAGAGAAATTGACAATCACTTATTC**G**TCATCAATGATGTGGGAGATCAAT	*1620*
**PB2 245467 Method E**	*1561*	ACACAAGGAACAGAGAAATTGACAATCACTTATTCATCATCAATGATGTGGGAGATCAAT	*1620*
**PB2 245467 Method K**	*1561*	ACACAAGGAACAGAGAAATTGACAATCACTTATTCATCATCAATGATGTGGGAGATCAAT	*1620*
**PB2 245467 Method N**	*1561*	ACACAAGGAACAGAGAAATTGACAATCACTTATTCATCATCAATGATGTGGGAGATCAAT	*1620*

**PB2 245467 MiSeq**	*1621*	GGACCCGAATCGGTGTTGGTCAACACTTATCAGTGGATCATCAGGAACTGGGAAACTGTG	*1680*
**PB2 245467 Method A**	*1621*	GGACCCGAATCGGTGTTGGTCAACACTTATCAGTGGATCATCAGGAACTGGGAAACTGTG	*1680*
**PB2 245467 Method S**	*1621*	GGACC**T**GA**G**TCGGTGTTGGTCAACACTTATCAGTGGATCATCAG**A**AA**T**TGGGAAACTGTG	*1680*
**PB2 245467 Method E**	*1621*	GGACCCGAATCGGTGTTGGTCAACACTTATCAGTGGATCATCAGGAACTGGGAAACTGTG	*1680*
**PB2 245467 Method K**	*1621*	GGACCCGAATCGGTGTTGGTCAACACTTATCAGTGGATCATCAGGAACTGGGAAACTGTG	*1680*
**PB2 245467 Method N**	*1621*	GGACCCGAATCGGTGTTGGTCAACACTTATCAGTGGATCATCAGGAACTGGGAAACTGTG	*1680*

**PB2 245467 MiSeq**	*1681*	AAAATTCAATGGTCACAGGATCCCACAATGTTATATAATAAGATGGAATTCGAGCCATTC	*1740*
**PB2 245467 Method A**	*1681*	AAAATTCAATGGTCACAGGATCCCACAATGTTATATAATAAGATGGAATTCGAGCCATTC	*1740*
**PB2 245467 Method S**	*1681*	AAAATTCAATGGTCACAGGATCCCACAATGTTATATAATAAGATGGAATTCGAGCCATTC	*1740*
**PB2 245467 Method E**	*1681*	AAAATTCAATGGTCACAGGATCCCACAATGTTATATAATAAGATGGAATTCGAGCCATTC	*1740*
**PB2 245467 Method K**	*1681*	AAAATTCAATGGTCACAGGATCCCACAATGTTATATAATAAGATGGAATTCGAGCCATTC	*1740*
**PB2 245467 Method N**	*1681*	AAAATTCAATGGTCACAGGATCCCACAATGTTATATAATAAGATGGAATTCGAGCCATTC	*1740*

**PB2 245467 MiSeq**	*1741*	CAGTCTCTGATACCTAAAGCAGCCAGAGGTCAATACAGTGGATTCGTGAGGACACTATTC	*1800*
**PB2 245467 Method A**	*1741*	CAGTCTCTGATACCTAAAGCAGCCAGAGGTCAATACAGTGGATTCGTGAGGACACTATTC	*1800*
**PB2 245467 Method S**	*1741*	CAGTCTCTGGTACCTAAAGCAGCCAGAGGTCAATACAGTGGATTCGTGAGGACACTATTC	*1800*
**PB2 245467 Method E**	*1741*	CAGTCTCTGATACCTAAAGCAGCCAGAGGTCAATACAGTGGATTCGTGAGGACACTATTC	*1800*
**PB2 245467 Method K**	*1741*	CAGTCTCTGATACCTAAAGCAGCCAGAGGTCAATACAGTGGATTCGTGAGGACACTATTC	*1800*
**PB2 245467 Method N**	*1741*	CAGTCTCTGATACCTAAAGCAGCCAGAGGTCAATACAGTGGATTCGTGAGGACACTATTC	*1800*

**PB2 245467 MiSeq**	*1801*	CAGCAGATGCGAGATGTGCTCGGAACATTTGACACTGTCCAAATAATAAAACTCCTCCCC	*1860*
**PB2 245467 Method A**	*1801*	CAGCAGATGCGAGATGTGCTCGGAACATTTGACACTGTCCAAATAATAAAACTCCTCCCC	*1860*
**PB2 245467 Method S**	*1801*	CAGCAGATGCGAGATGTGCTCGGAACATTTGACACTGTTCAAATAATAAAACTCCTCCCC	*1860*
**PB2 245467 Method E**	*1801*	CAGCAGATGCGAGATGTGCTCGGAACATTTGACACTGTCCAAATAATAAAACTCCTCCCC	*1860*
**PB2 245467 Method K**	*1801*	CAGCAGATGCGAGATGTGCTCGGAACATTTGACACTGTCCAAATAATAAAACTCCTCCCC	*1860*
**PB2 245467 Method N**	*1801*	CAGCAGATGCGAGATGTGCTCGGAACATTTGACACTGTCCAAATAATAAAACTCCTCCCC	*1860*

**PB2 245467 MiSeq**	*1861*	TTTGCTGCTGCCCCACCAGAACAAAGTAGAATGCAATTCTCCTCCCTGACTGTAAATGTG	*1920*
**PB2 245467 Method A**	*1861*	TTTGCTGCTGCCCCACCAGAACAAAGTAGAATGCAATTCTCCTCCCTGACTGTAAATGTG	*1920*
**PB2 245467 Method S**	*1861*	TTTGCTGCTGCCCCACCAGAACAAAGTAGGATGCAATTCTCCTCCCTGACTGTAAATGTG	*1920*
**PB2 245467 Method E**	*1861*	TTTGCTGCTGCCCCACCAGAACAAAGTAGAATGCAATTCTCCTCCCTGACTGTAAATGTG	*1920*
**PB2 245467 Method K**	*1861*	TTTGCTGCTGCCCCACCAGAACAAAGTAGAATGCAATTCTCCTCCCTGACTGTAAATGTG	*1920*
**PB2 245467 Method N**	*1861*	TTTGCTGCTGCCCCACCAGAACAAAGTAGAATGCAATTCTCCTCCCTGACTGTAAATGTG	*1920*

**PB2 245467 MiSeq**	*1921*	AGAGGATCAGGAATGAGAATACTGGTTAGAGGCAATTCCCCAGTGTTCAATTACAACAAG	*1980*
**PB2 245467 Method A**	*1921*	AGAGGATCAGGAATGAGAATACTGGTTAGAGGCAATTCCCCAGTGTTCAATTACAACAAG	*1980*
**PB2 245467 Method S**	*1921*	AGAGGATCAGGAATGAGAATACTGGTAAGAGGCAATTCCCCAGTGTTCAATTACAACAAG	*1980*
**PB2 245467 Method E**	*1921*	AGAGGATCAGGAATGAGAATACTGGTTAGAGGCAATTCCCCAGTGTTCAATTACAACAAG	*1980*
**PB2 245467 Method K**	*1921*	AGAGGATCAGGAATGAGAATACTGGTTAGAGGCAATTCCCCAGTGTTCAATTACAACAAG	*1980*
**PB2 245467 Method N**	*1921*	AGAGGATCAGGAATGAGAATACTGGTTAGAGGCAATTCCCCAGTGTTCAATTACAACAAG	*1980*

**PB2 245467 MiSeq**	*1981*	GCCACCAAGAGGCTCACAGTTCTCGGGAAAGATGCAGGTGCATTGACCGAAGATCCAGAT	*2040*
**PB2 245467 Method A**	*1981*	GCCACCAAGAGGCTCACAGTTCTCGGGAAAGATGCAGGTGCATTGACCGAAGATCCAGAT	*2040*
**PB2 245467 Method S**	*1981*	GCCACCAAGAGGCTCACAGTTCTCGGGAAAGATGCAGGTGCATTGACCGAAGATCCAGAT	*2040*
**PB2 245467 Method E**	*1981*	GCCACCAAGAGGCTCACAGTTCTCGGGAAAGATGCAGGTGCATTGACCGAAGATCCAGAT	*2040*
**PB2 245467 Method K**	*1981*	GCCACCAAGAGGCTCACAGTTCTCGGGAAAGATGCAGGTGCATTGACCGAAGATCCAGAT	*2040*
**PB2 245467 Method N**	*1981*	GCCACCAAGAGGCTCACAGTTCTCGGGAAAGATGCAGGTGCATTGACCGAAGATCCAGAT	*2040*

**PB2 245467 MiSeq**	*2041*	GAAGGCACATCTGGAGTAGAGTCTGCTGTTCTAAGAGGATTCCTCATTTTGGGCAAAGAA	*2100*
**PB2 245467 Method A**	*2041*	GAAGGCACATCTGGAGTAGAGTCTGCTGTTCTAAGAGGATTCCTCATTTTGGGCAAAGAA	*2100*
**PB2 245467 Method S**	*2041*	GAAGGCACAGCTGGAGTAGAGTCTGCTGTTCTACGAGGATTCCTCATTTTGGGCAAAGAA	*2100*
**PB2 245467 Method E**	*2041*	GAAGGCACATCTGGAGTAGAGTCTGCTGTTCTAAGAGGATTCCTCATTTTGGGCAAAGAA	*2100*
**PB2 245467 Method K**	*2041*	GAAGGCACATCTGGAGTAGAGTCTGCTGTTCTAAGAGGATTCCTCATTTTGGGCAAAGAA	*2100*
**PB2 245467 Method N**	*2041*	GAAGGCACATCTGGAGTAGAGTCTGCTGTTCTAAGAGGATTCCTCATTTTGGGCAAAGAA	*2100*

**PB2 245467 MiSeq**	*2101*	GACAAGAGATATGGCCCAGCATTGAGCATCAATGAGCTGAGCAATCTTGCAAAGGGAGAG	*2160*
**PB2 245467 Method A**	*2101*	GACAAGAGATATGGCCCAGCATTGAGCATCAATGAGCTGAGCAATCTTGCAAAGGGAGAG	*2160*
**PB2 245467 Method S**	*2101*	GACAAGAGATATGGACCAGCATTGAGCATCAATGAGCTGAGCAATCTTGCAAAGGGAGAG	*2160*
**PB2 245467 Method E**	*2101*	GACAAGAGATATGGCCCAGCATTGAGCATCAATGAGCTGAGCAATCTTGCAAAGGGAGAG	*2160*
**PB2 245467 Method K**	*2101*	GACAAGAGATATGGCCCAGCATTGAGCATCAATGAGCTGAGCAATCTTGCAAAGGGAGAG	*2160*
**PB2 245467 Method N**	*2101*	GACAAGAGATATGGCCCAGCATTGAGCATCAATGAGCTGAGCAATCTTGCAAAGGGAGAG	*2160*

**PB2 245467 MiSeq**	*2161*	AAGGCTAATGTGCTAATTGGGCAAGGAGACGTGGTGTTGGTAATGAAACGGAAACGGGAC	*2220*
**PB2 245467 Method A**	*2161*	AAGGCTAATGTGCTAATTGGGCAAGGAGACGTGGTGTTGGTAATGAAACGGAAACGGGAC	*2220*
**PB2 245467 Method S**	*2161*	AAGGCTAATGTGCTAATTGGGCAAGGAGACGTGGTGTTGGTAATGAAACGGAAACGGGAC	*2220*
**PB2 245467 Method E**	*2161*	AAGGCTAATGTGCTAATTGGGCAAGGAGACGTGGTGTTGGTAATGAAACGGAAACGGGAC	*2220*
**PB2 245467 Method K**	*2161*	AAGGCTAATGTGCTAATTGGGCAAGGAGACGTGGTGTTGGTAATGAAACGGAAACGGGAC	*2220*
**PB2 245467 Method N**	*2161*	AAGGCTAATG-------TGGGCAAGGAGACGTGGTGTTGGTAA------------------	*2196*

**PB2 245467 MiSeq**	*2221*	TCTAGCATACTTACTGACAGCCAGACAGCGACCAAAAGGATTCGGATGGCCATCAATTAG	*2280*
**PB2 245467 Method A**	*2221*	TCTAGCATACTTACTGACAGCCAGACAGCGACCAAAAGGATTCGGATGGCCATCAATTAG	*2280*
**PB2 245467 Method S**	*2221*	TCTAGCATACTTACTGACAGCCAGACAGCGACCAAAAGGATTCGGATGGCCATCAATTAG	*2280*
**PB2 245467 Method E**	*2221*	TCTAGCATACTTACTGACAGCCAGACAGCGACCAAAAGGATTCGGATGGCCATCAATTAG	*2280*
**PB2 245467 Method K**	*2221*	TCTAGCATACTTACTGACAGCCAGACAGCGACCAAAAGGATTCGGATGGCCATCAATTAG	*2280*
**PB2 245467 Method N**	*2197*		*2196*
