# Supplementary material for: An Evaluation of Avian Influenza Virus Whole-Genome Sequencing Approaches Using Nanopore Technology
Source: Microorganisms. 2023 Feb 19;11(2):529. doi: 10.3390/microorganisms11020529 (PMC9967579; doi:10.3390/microorganisms11020529)
Supplement: Supplementary file 1 [file microorganisms-11-00529-s001.zip › manuscript.v8 230219 Suppl Figures and Tables/Supplementary Figures S1a-h 245467/Supplementary Figure S1f N1.pdf]

Formatted Alignments

**N1 245467 MiSeq**	*1*	ATGAATCCAAATCAAAAGATAACAACCATTGGATCAATCTGTATGGTAATTGGTATAGTC	*60*
**N1 245467 Method A**	*1*	ATGAATCCAAATCAAAAGATAACAACCATTGGATCAATCTGTATGGTAATTGGTATAGTC	*60*
**N1 245467 Method S**	*1*	ATGAATCCAAATCAAAAGATAACAACCATTGGATCAATCTGTATGGTAATTGGGATAGTC	*60*
**N1 245467 Method E**	*1*	ATGAATCCAAATCAAAAGATAACAACCATTGGATCAATCTGTATGGTAATTGGTATAGTC	*60*
**N1 245467 Method K**	*1*	ATGAATCCAAATCAAAAGATAACAACCATTGGATCAATCTGTATGGTAATTGGTATAGTC	*60*
**N1 245467 Method N**	*1*	ATGAATCCAAATCAAAAGATAACAACCATTGGATCAATCTGTATGGTAATTGGTATAGTC	*60*
**N1 245467 MiSeq**	*61*	AGCTTGATGCTGCAAATTGGGAACATAATCTCAATATGGGTTAGCCATTCAATCCAAACA	*120*
**N1 245467 Method A**	*61*	AGCTTGATGCTGCAAATTGGGAACATAATCTCAATATGGGTTAGCCATTCAATCCAAACA	*120*
**N1 245467 Method S**	*61*	AGCTTGATGCTGCAAATTGGGAATATAATCTCAATATGGGTTAGCCATTCAATCCAAACA	*120*
**N1 245467 Method E**	*61*	AGCTTGATGCTGCAAATTGGGAACATAATCTCAATATGGGTTAGCCATTCAATCCAAACA	*120*
**N1 245467 Method K**	*61*	AGCTTGATGCTGCAAATTGGGAACATAATCTCAATATGGGTTAGCCATTCAATCCAAACA	*120*
**N1 245467 Method N**	*61*	AGCTTGATGCTGCAAATTGGGAACATAATCTCAATATGGGTTAGCCATTCAATCCAAACA	*120*
**N1 245467 MiSeq**	*121*	GGGAATCAATACCAGCCTGAACCATGCAATCAAAGCATCATTACCTATGAGAACAACACC	*180*
**N1 245467 Method A**	*121*	GGGAATCAATACCAGCCTGAACCATGCAATCAAAGCATCATTACCTATGAGAACAACACC	*180*
**N1 245467 Method S**	*121*	GGGAATCAATACCAGCCTGAACCATGCAATCAAAGCATCATTACCTATGAGAACAACACC	*180*
**N1 245467 Method E**	*121*	GGGAATCAATACCAGCCTGAACCATGCAATCAAAGCATCATTACCTATGAGAACAACACC	*180*
**N1 245467 Method K**	*121*	GGGAATCAATACCAGCCTGAACCATGCAATCAAAGCATCATTACCTATGAGAACAACACC	*180*
**N1 245467 Method N**	*121*	GGGAATCAATACCAGCCTGAACCATGCAATCAAAGCATCATTACCTATGAGAACAACACC	*180*
**N1 245467 MiSeq**	*181*	TGGGTAAATCAGACGTATGTCAACATCAGCAATACCAATTTTCTTGCTGAGCAGGCTGTT	*240*
**N1 245467 Method A**	*181*	TGGGTAAATCAGACGTATGTCAACATCAGCAATACCAATTTTCTTGCTGAGCAGGCTGTT	*240*
**N1 245467 Method S**	*181*	TGGGTAAATCAGACGTATGTCAACATCAGCAATACCAATTTTCTTGCTGAGCAGGCTGTT	*240*
**N1 245467 Method E**	*181*	TGGGTAAATCAGACGTATGTCAACATCAGCAATACCAATTTTCTTGCTGAGCAGGCTGTT	*240*
**N1 245467 Method K**	*181*	TGGGTAAATCAGACGTATGTCAACATCAGCAATACCAATTTTCTTGCTGAGCAGGCTGTT	*240*
**N1 245467 Method N**	*181*	TGGGTAAATCAGACGTATGTCAACATCAGCAATACCAATTTTCTTGCTGAGCAGGCTGTT	*240*

**N1 245467 MiSeq**	*241*	ACTTCGGTAACATTAGCGGGCAATTCATCTCTTTGCCCTATTAGTGGGTGGGCAATATAC	*300*
**N1 245467 Method A**	*241*	ACTTCGGTAACATTAGCGGGCAATTCATCTCTTTGCCCTATTAGTGGGTGGGCAATATAC	*300*
**N1 245467 Method S**	*241*	ACTTCGGTAACATTAGCGGGCAATTCATCTCTTTGCCCTATTAGTGGGTGGGCAATATAC	*300*
**N1 245467 Method E**	*241*	ACTTCGGTAACATTAGCGGGCAATTCATCTCTTTGCCCTATTAGTGGGTGGGCAATATAC	*300*
**N1 245467 Method K**	*241*	ACTTCGGTAACATTAGCGGGCAATTCATCTCTTTGCCCTATTAGTGGGTGGGCAATATAC	*300*
**N1 245467 Method N**	*241*	ACTTCGGTAACATTAGCGGGCAATTCATCTCTTTGCCCTATTAGTGGGTGGGCAATATAC	*300*

**N1 245467 MiSeq**	*301*	AGTAAGGACAACGGTATAAGGATTGGGTCCAAGGGGGATGTGTTTGTTATAAGAGAACCG	*360*
**N1 245467 Method A**	*301*	AGTAAGGACAACGGTATAAGGATTGGGTCCAAGGGGGATGTGTTTGTTATAAGAGAACCG	*360*
**N1 245467 Method S**	*301*	AGTAAGGACAACGGTATAAGAATTGGGTCCAAGGGGGATGTGTTTGTTATAAGAGAACCG	*360*
**N1 245467 Method E**	*301*	AGTAAGGACAACGGTATAAGGATTGGGTCCAAGGGGGATGTGTTTGTTATAAGAGAACCG	*360*
**N1 245467 Method K**	*301*	AGTAAGGACAACGGTATAAGGATTGGGTCCAAGGGGGATGTGTTTGTTATAAGAGAACCG	*360*
**N1 245467 Method N**	*301*	AGTAAGGACAACGGTATAAGGATTGGGTCCAAGGGGGATGTGTTTGTTATAAGAGAACCG	*360*

**N1 245467 MiSeq**	*361*	TTCATCTCATGCTCCCACTTGGAATGCAGAACCTTTTTCCTGACCCAGGGAGCTCTGCTG	*420*
**N1 245467 Method A**	*361*	TTCATCTCATGCTCCCACTTGGAATGCAGAACCTTTTTCCTGACCCAGGGAGCTCTGCTG	*420*
**N1 245467 Method S**	*361*	TTCATCTCATGCTCCCACTTGGAATGCAGAACCTTTTTCCTGACCCAGGGAGCTCTGCTG	*420*
**N1 245467 Method E**	*361*	TTCATCTCATGCTCCCACTTGGAATGCAGAACCTTTTTCCTGACCCAGGGAGCTCTGCTG	*420*
**N1 245467 Method K**	*361*	TTCATCTCATGCTCCCACTTGGAATGCAGAACCTTTTTCCTGACCCAGGGAGCTCTGCTG	*420*
**N1 245467 Method N**	*361*	TTCATCTCATGCTCCCACTTGGAATGCAGAACCTTTTTCCTGACCCAGGGAGCTCTGCTG	*420*

**N1 245467 MiSeq**	*421*	AATGACAAACATTCTAATGGGACCGTTAAGGATAGAAGCCCTTATAGAACTTTGATGAGT	*480*
**N1 245467 Method A**	*421*	AATGACAAACATTCTAATGGGACCGTTAAGGATAGAAGCCCTTATAGAACTTTGATGAGT	*480*
**N1 245467 Method S**	*421*	AATGACAAACATTCTAATGGGACCGTTAAGGATAGAAGCCCCTATAGAACTTTGATGAGT	*480*
**N1 245467 Method E**	*421*	AATGACAAACATTCTAATGGGACCGTTAAGGATAGAAGCCCTTATAGAACTTTGATGAGT	*480*
**N1 245467 Method K**	*421*	AATGACAAACATTCTAATGGGACCGTTAAGGATAGAAGCCCTTATAGAACTTTGATGAGT	*480*
**N1 245467 Method N**	*421*	AATGACAAACATTCTAATGGGACCGTTAAGGATAGAAGCCCTTATAGAACTTTGATGAGT	*480*

**N1 245467 MiSeq**	*481*	TGTCCCGTGGGTGAGGCTCCTTCCCCGTACAATTCAAGATTTGAGTCTGTTGCTTGGTCG	*540*
**N1 245467 Method A**	*481*	TGTCCCGTGGGTGAGGCTCCTTCCCCGTACAATTCAAGATTTGAGTCTGTTGCTTGGTCG	*540*
**N1 245467 Method S**	*481*	TGTCCCGTGGGTGAGGCTCCTTCCCCGTACAATTCAAGATTTGAGTCTGTTGCTTGGTCG	*540*
**N1 245467 Method E**	*481*	TGTCCCGTGGGTGAGGCTCCTTCCCCGTACAATTCAAGATTTGAGTCTGTTGCTTGGTCG	*540*
**N1 245467 Method K**	*481*	TGTCCCGTGGGTGAGGCTCCTTCCCCGTACAATTCAAGATTTGAGTCTGTTGCTTGGTCG	*540*
**N1 245467 Method N**	*481*	TGTCCCGTGGGTGAGGCTCCTTCCCCGTACAATTCAAGATTTGAGTCTGTTGCTTGGTCG	*540*

**N1 245467 MiSeq**	*541*	GCAAGTGCTTGTCATGATGGCATCAGTTGGTTGACAATCGGTATTTCTGGTCCAGACAAT	*600*
**N1 245467 Method A**	*541*	GCAAGTGCTTGTCATGATGGCATCAGTTGGTTGACAATCGGTATTTCTGGTCCAGACAAT	*600*
**N1 245467 Method S**	*541*	GCAAGTGCTTGTCATGATGGCATTAGTTGGTTGACAATCGGTATTTCTGGTCCAGACAAT	*600*
**N1 245467 Method E**	*541*	GCAAGTGCTTGTCATGATGGCATCAGTTGGTTGACAATCGGTATTTCTGGTCCAGACAAT	*600*
**N1 245467 Method K**	*541*	GCAAGTGCTTGTCATGATGGCATCAGTTGGTTGACAATCGGTATTTCTGGTCCAGACAAT	*600*
**N1 245467 Method N**	*541*	GCAAGTGCTTGTCATGATGGCATCAGTTGGTTGACAATCGGTATTTCTGGTCCAGACAAT	*600*

**N1 245467 MiSeq**	*601*	GGAGCTGTGGCTGTATTGAAGTACAATGGCATAATAACGGATACTATCAAGAGTTGGAGA	*660*
**N1 245467 Method A**	*601*	GGAGCTGTGGCTGTATTGAAGTACAATGGCATAATAACGGATACTATCAAGAGTTGGAGA	*660*
**N1 245467 Method S**	*601*	GGAGCTGTGGCTGTATTGAAGTACAATGGCATAATAACGGATACTATCAAGAGTTGGAGA	*660*
**N1 245467 Method E**	*601*	GGAGCTGTGGCTGTATTGAAGTACAATGGCATAATAACGGATACTATCAAGAGTTGGAGA	*660*
**N1 245467 Method K**	*601*	GGAGCTGTGGCTGTATTGAAGTACAATGGCATAATAACGGATACTATCAAGAGTTGGAGA	*660*
**N1 245467 Method N**	*601*	GGAGCTGTGGCTGTATTGAAGTACAATGGCATAATAACGGATACTATCAAGAGTTGGAGA	*660*

**N1 245467 MiSeq**	*661*	AACAACATTTTGAGAACTCAAGAATCTGAATGTGCGTGCGTAAATGGCTCTTGCTTCACC	*720*
**N1 245467 Method A**	*661*	AACAACATTTTGAGAACTCAAGAATCTGAATGTGCGTGCGTAAATGGCTCTTGCTTCACC	*720*
**N1 245467 Method S**	*661*	AACAACATTTTGAGAACTCAAGAATCTGAATGTGCGTGCGTAAATGGCTCTTGCTTCACT	*720*
**N1 245467 Method E**	*661*	AACAACATTTTGAGAACTCAAGAATCTGAATGTGCGTGCGTAAATGGCTCTTGCTTCACC	*720*
**N1 245467 Method K**	*661*	AACAACATTTTGAGAACTCAAGAATCTGAATGTGCGTGCGTAAATGGCTCTTGCTTCACC	*720*
**N1 245467 Method N**	*661*	AACAACATTTTGAGAACTCAAGAATCTGAATGTGCGTGCGTAAATGGCTCTTGCTTCACC	*720*

```
N1 245467 MiSeq     721 GTAATGACTGATGGGCCAAGCAATGGGCAGGCCTCATATAAAATCTTCAAGATAGAGAAA 780
N1 245467 Method A  721 GTAATGACTGATGGGCCAAGCAATGGGCAGGCCTCATATAAAATCTTCAAGATAGAGAAA 780
N1 245467 Method S  721 GTAATGACTGATGGACCAAGCAATGGGCAGGCCTCATATAAAATCTTCAAGATAGAGAAA 780
N1 245467 Method E  721 GTAATGACTGATGGGCCAAGCAATGGGCAGGCCTCATATAAAATCTTCAAGATAGAGAAA 780
N1 245467 Method K  721 GTAATGACTGATGGGCCAAGCAATGGGCAGGCCTCATATAAAATCTTCAAGATAGAGAAA 780
N1 245467 Method N  721 GTAATGACTGATGGGCCAAGCAATGGGCAGGCCTCATATAAAATCTTCAAGATAGAGAAA 780

N1 245467 MiSeq     781 GGGAAAGTTGTCAAATCAGTTGAATTGAATGCCCCTAATTACCACTACGAGGAATGCTCC 840
N1 245467 Method A  781 GGGAAAGTTGTCAAATCAGTTGAATTGAATGCCCCTAATTACCACTACGAGGAATGCTCC 840
N1 245467 Method S  781 GGGAAAGTTGTCAAATCAGTTGAATTGAATGCCCCTAATTACCACTACGAGGAATGCTCC 840
N1 245467 Method E  781 GGGAAAGTTGTCAAATCAGTTGAATTGAATGCCCCTAATTACCACTACGAGGAATGCTCC 840
N1 245467 Method K  781 GGGAAAGTTGTCAAATCAGTTGAATTGAATGCCCCTAATTACCACTACGAGGAATGCTCC 840
N1 245467 Method N  781 GGGAAAGTTGTCAAATCAGTTGAATTGAATGCCCCTAATTACCACTACGAGGAATGCTCC 840

N1 245467 MiSeq     841 TGTTATCCTAATGCGGGTGATATTATGTGTGTGTGCAGGGACAATTGGCATGGCTCAAAC 900
N1 245467 Method A  841 TGTTATCCTAATGCGGGTGATATTATGTGTGTGTGCAGGGACAATTGGCATGGCTCAAAC 900
N1 245467 Method S  841 TGTTATCCTGATGCGGGTGATATTATGTGTGTGTGCAGGGACAATTGGCATGGCTCAAAC 900
N1 245467 Method E  841 TGTTATCCTAATGCGGGTGATATTATGTGTGTGTGCAGGGACAATTGGCATGGCTCAAAC 900
N1 245467 Method K  841 TGTTATCCTAATGCGGGTGATATTATGTGTGTGTGCAGGGACAATTGGCATGGCTCAAAC 900
N1 245467 Method N  841 TGTTATCCTAATGCGGGTGATATTATGTGTGTGTGCAGGGACAATTGGCATGGCTCAAAC 900

N1 245467 MiSeq     901 CGGCCGTGGGTATCTTTTAATCAAAATCTGGAGTATCAAATAGGATATATATGCAGTGGG 960
N1 245467 Method A  901 CGGCCGTGGGTATCTTTTAATCAAAATCTGGAGTATCAAATAGGATATATATGCAGTGGG 960
N1 245467 Method S  901 CGGCCGTGGGTATCTTTTAATCAAAATCTGGAGTATCAAATAGGATATATATGCAGTGGG 960
N1 245467 Method E  901 CGGCCGTGGGTATCTTTTAATCAAAATCTGGAGTATCAAATAGGATATATATGCAGTGGG 960
N1 245467 Method K  901 CGGCCGTGGGTATCTTTTAATCAAAATCTGGAGTATCAAATAGGATATATATGCAGTGGG 960
N1 245467 Method N  901 CGGCCGTGGGTATCTTTTAATCAAAATCTGGAGTATCAAATAGGATATATATGCAGTGGG 960
```

**N1 245467 MiSeq**	*961*	GTTTTCGGGGACAATCCCCGCCCCAATGATGGAACAGGCAGTTGCAGTCCAATGTCCTCT	*1020*
**N1 245467 Method A**	*961*	GTTTTCGGGGACAATCCCCGCCCCAATGATGGAACAGGCAGTTGCAGTCCAATGTCCTCT	*1020*
**N1 245467 Method S**	*961*	GTTTTCGGGGACAATCCCCGCCCCAATGATGGAACAGGCAGTTGCAGTCCAATGTCCTCT	*1020*
**N1 245467 Method E**	*961*	GTTTTCGGGGACAATCCCCGCCCCAATGATGGAACAGGCAGTTGCAGTCCAATGTCCTCT	*1020*
**N1 245467 Method K**	*961*	GTTTTCGGGGACAATCCCCGCCCCAATGATGGAACAGGCAGTTGCAGTCCAATGTCCTCT	*1020*
**N1 245467 Method N**	*961*	GTTTTCGGGGACAATCCCCGCCCCAATGATGGAACAGGCAGTTGCAGTCCAATGTCCTCT	*1020*

**N1 245467 MiSeq**	*1021*	AATGGGGCATATGGGGTAAAAGGGTTTTCATTTAAGTACGGTAATGGGGTTTGGATCGGA	*1080*
**N1 245467 Method A**	*1021*	AATGGGGCATATGGGGTAAAAGGGTTTTCATTTAAGTACGGTAATGGGGTTTGGATCGGA	*1080*
**N1 245467 Method S**	*1021*	AACGGGGCATATGGGGTAAAAGGGTTTTCATTTAAGTACGGTAATGGGGTTTGGATCGGA	*1080*
**N1 245467 Method E**	*1021*	AATGGGGCATATGGGGTAAAAGGGTTTTCATTTAAGTACGGTAATGGGGTTTGGATCGGA	*1080*
**N1 245467 Method K**	*1021*	AATGGGGCATATGGGGTAAAAGGGTTTTCATTTAAGTACGGTAATGGGGTTTGGATCGGA	*1080*
**N1 245467 Method N**	*1021*	AATGGGGCATATGGGGTAAAAGGGTTTTCATTTAAGTACGGTAATGGGGTTTGGATCGGA	*1080*

**N1 245467 MiSeq**	*1081*	AGAACAAAAAGCACTAGTTCCAGAAGCGGCTTTGAGATGATTTGGGATCCGAATGGGTGG	*1140*
**N1 245467 Method A**	*1081*	AGAACAAAAAGCACTAGTTCCAGAAGCGGCTTTGAGATGATTTGGGATCCGAATGGGTGG	*1140*
**N1 245467 Method S**	*1081*	AGAACAAAAAGCACTAGTTCCAGAAGCGGCTTTGAGATGATTTGGGATCCGAATGGGTGG	*1140*
**N1 245467 Method E**	*1081*	AGAACAAAAAGCACTAGTTCCAGAAGCGGCTTTGAGATGATTTGGGATCCGAATGGGTGG	*1140*
**N1 245467 Method K**	*1081*	AGAACAAAAAGCACTAGTTCCAGAAGCGGCTTTGAGATGATTTGGGATCCGAATGGGTGG	*1140*
**N1 245467 Method N**	*1081*	AGAACAAAAAGCACTAGTTCCAGAAGCGGCTTTGAGATGATTTGGGATCCGAATGGGTGG	*1140*

**N1 245467 MiSeq**	*1141*	ACTGAGACGGACAGTAGTTTCTCAGTGAAGCAAGACATTGTAGAAATAACTGACTGGTCA	*1200*
**N1 245467 Method A**	*1141*	ACTGAGACGGACAGTAGTTTCTCAGTGAAGCAAGACATTGTAGAAATAACTGACTGGTCA	*1200*
**N1 245467 Method S**	*1141*	ACTGAGACGGACAGTAGTTTCTCAGTGAAGCAAGACATTGTAGAAATAACTGACTGGTCA	*1200*
**N1 245467 Method E**	*1141*	ACTGAGACGGACAGTAGTTTCTCAGTGAAGCAAGACATTGTAGAAATAACTGACTGGTCA	*1200*
**N1 245467 Method K**	*1141*	ACTGAGACGGACAGTAGTTTCTCAGTGAAGCAAGACATTGTAGAAATAACTGACTGGTCA	*1200*
**N1 245467 Method N**	*1141*	ACTGAGACGGACAGTAGTTTCTCAGTGAAGCAAGACATTGTAGAAATAACTGACTGGTCA	*1200*

**N1 245467 MiSeq**	*1201*	GGATATAGTGGGAGTTTTGTCCAGCATCCAGAACTGACAGGATTAGATTGCATGAGGCCT	*1260*
**N1 245467 Method A**	*1201*	GGATATAGTGGGAGTTTTGTCCAGCATCCAGAACTGACAGGATTAGATTGCATGAGGCCT	*1260*
**N1 245467 Method S**	*1201*	GGATATAGTGGAAGTTTTGTCCAGCATCCAGAACTGACAGGATTAGATTGCATGAGGCCT	*1260*
**N1 245467 Method E**	*1201*	GGATATAGTGGGAGTTTTGTCCAGCATCCAGAACTGACAGGATTAGATTGCATGAGGCCT	*1260*
**N1 245467 Method K**	*1201*	GGATATAGTGGGAGTTTTGTCCAGCATCCAGAACTGACAGGATTAGATTGCATGAGGCCT	*1260*
**N1 245467 Method N**	*1201*	GGATATAGTGGGAGTTTTGTCCAGCATCCAGAACTGACAGGATTAGATTGCATGAGGCCT	*1260*

**N1 245467 MiSeq**	*1261*	TGTTTCTGGGTTGAGCTAATTAGAGGGAGGCCCAAAGAGAACACAATTTGGACTAGCGGG	*1320*
**N1 245467 Method A**	*1261*	TGTTTCTGGGTTGAGCTAATTAGAGGGAGGCCCAAAGAGAACACAATTTGGACTAGCGGG	*1320*
**N1 245467 Method S**	*1261*	TGTTTCTGGGTTGAGCTAATTAGAGGGAGGCCCAAAGAGAACACAATTTGGACTAGCGGG	*1320*
**N1 245467 Method E**	*1261*	TGTTTCTGGGTTGAGCTAATTAGAGGGAGGCCCAAAGAGAACACAATTTGGACTAGCGGG	*1320*
**N1 245467 Method K**	*1261*	TGTTTCTGGGTTGAGCTAATTAGAGGGAGGCCCAAAGAGAACACAATTTGGACTAGCGGG	*1320*
**N1 245467 Method N**	*1261*	TGTTTCTGGGTTGAGCTAATTAGAGGGAGGCCCAAAGAGAACACAATTTGGACTAGCGGG	*1320*

**N1 245467 MiSeq**	*1321*	AGCAGCATATCCTTTTGTGGTGTAAATAGTGACACTGTGGGTTGGTCTTGGCCAGACGGT	*1380*
**N1 245467 Method A**	*1321*	AGCAGCATATCCTTTTGTGGTGTAAATAGTGACACTGTGGGTTGGTCTTGGCCAGACGGT	*1380*
**N1 245467 Method S**	*1321*	AGCAGCATATCCTTTTGTGGTGTAAATAGTGACACTGTGGGTTGGTCTTGGCCAGACGGT	*1380*
**N1 245467 Method E**	*1321*	AGCAGCATATCCTTTTGTGGTGTAAATAGTGACACTGTGGGTTGGTCTTGGCCAGACGGT	*1380*
**N1 245467 Method K**	*1321*	AGCAGCATATCCTTTTGTGGTGTAAATAGTGACACTGTGGGTTGGTCTTGGCCAGACGGT	*1380*
**N1 245467 Method N**	*1321*	AGCAGCATATCCTTTTGTGGTGTAAATAGTGACACTGTGGGTTGGTCTTGGCCAGACGGT	*1380*

**N1 245467 MiSeq**	*1381*	GCTGAGTTGCCATTCACCATTGACAAGTAG	*1410*
**N1 245467 Method A**	*1381*	GCTGAGTTGCCATTCACCATTGACAAGTAG	*1410*
**N1 245467 Method S**	*1381*	GCTGAGTTGCCATTCACCATTGACAAGTAG	*1410*
**N1 245467 Method E**	*1381*	GCTGAGTTGCCATTCACCATTGACAAGTAG	*1410*
**N1 245467 Method K**	*1381*	GCTGAGTTGCCATTCACCATTGACAAGTAG	*1410*
**N1 245467 Method N**	*1381*	GCTGAGTTGCCATTCACCATTGACAAGTAG	*1410*
