# Supplementary material for: An Evaluation of Avian Influenza Virus Whole-Genome Sequencing Approaches Using Nanopore Technology
Source: Microorganisms. 2023 Feb 19;11(2):529. doi: 10.3390/microorganisms11020529 (PMC9967579; doi:10.3390/microorganisms11020529)
Supplement: Supplementary file 1 [file microorganisms-11-00529-s001.zip › manuscript.v8 230219 Suppl Figures and Tables/Supplementary Figures S1a-h 245467/Supplementary Figure S1h NS.pdf]

Formatted Alignments

Sequence	Start	Alignment	End
**NS 245467 MiSeq**	*1*	ATGGATTCCAACACTGTGTCAAGCTTTCAGGTAGACTGCTTTCTTTGGCATGTCCGCAAA	*60*
**NS 245467 Method A**	*1*	ATGGATTCCAACACTGTGTCAAGCTTTCAGGTAGACTGCTTTCTTTGGCATGTCCGCAAA	*60*
**NS 245467 Method S**	*1*	ATGGATTCCAACACTGTGTCAAGCTTTCAGGTAGACTGCTTTCTTTGGCATGTCCGCAAA	*60*
**NS 245467 Method E**	*1*	ATGGATTCCAACACTGTGTCAAGCTTTCAGGTAGACTGCTTTCTTTGGCATGTCCGCAAA	*60*
**NS 245467 Method K**	*1*	ATGGATTCCAACACTGTGTCAAGCTTTCAGGTAGACTGCTTTCTTTGGCATGTCCGCAAA	*60*
**NS 245467 Method N**	*1*	ATGGATTCCAACACTGTGTCAAGCTTTCAGGTAGACTGCTTTCTTTGGCATGTCCGCAAA	*60*
**NS 245467 MiSeq**	*61*	CGGTTTGCAGACCAAGAACTGGGTGATGCCCCATTCCTTGACCGGATTCGCCGAGATCAG	*120*
**NS 245467 Method A**	*61*	CGGTTTGCAGACCAAGAACTGGGTGATGCCCCATTCCTTGACCGGATTCGCCGAGATCAG	*120*
**NS 245467 Method S**	*61*	CGATTTGCAGACCAAGAACTGGGTGATGCCCCATTCCTTGACCGGCTTCGCCGAGATCAG	*120*
**NS 245467 Method E**	*61*	CGGTTTGCAGACCAAGAACTGGGTGATGCCCCATTCCTTGACCGGATTCGCCGAGATCAG	*120*
**NS 245467 Method K**	*61*	CGGTTTGCAGACCAAGAACTGGGTGATGCCCCATTCCTTGACCGGATTCGCCGAGATCAG	*120*
**NS 245467 Method N**	*61*	CGGTTTGCAGACCAAGAACTGGGTGATGCCCCATTCCTTGACCGGATTCGCCGAGATCAG	*120*
**NS 245467 MiSeq**	*121*	AAATCCCTGAGAGGAAGAGGCAGCACTCTTGGTCTGGGCATCGAAACAGCCACCCGTGCA	*180*
**NS 245467 Method A**	*121*	AAATCCCTGAGAGGAAGAGGCAGCACTCTTGGTCTGGGCATCGAAACAGCCACCCGTGCA	*180*
**NS 245467 Method S**	*121*	AAATCCCTGAGAGGAAGAGGCAGCACTCTTGGTCTGGACATCGAAACAGCCACCCGTGCG	*180*
**NS 245467 Method E**	*121*	AAATCCCTGAGAGGAAGAGGCAGCACTCTTGGTCTGGGCATCGAAACAGCCACCCGTGCA	*180*
**NS 245467 Method K**	*121*	AAATCCCTGAGAGGAAGAGGCAGCACTCTTGGTCTGGGCATCGAAACAGCCACCCGTGCA	*180*
**NS 245467 Method N**	*121*	AAATCCCTGAGAGGAAGAGGCAGCACTCTTGGTCTGGGCATCGAAACAGCCACCCGTGCA	*180*
**NS 245467 MiSeq**	*181*	GGAAAGCAGATAGTGGAGCGGATTCTGGAAGAAGAATCTGATGAGACACTTAAAATGACT	*240*
**NS 245467 Method A**	*181*	GGAAAGCAGATAGTGGAGCGGATTCTGGAAGAAGAATCTGATGAGACACTTAAAATGACT	*240*
**NS 245467 Method S**	*181*	GGAAAGCAGATAGTGGAGCGGATTCTGGAAGAAGAATCTAATGAGGCACTTAAAATGACT	*240*
**NS 245467 Method E**	*181*	GGAAAGCAGATAGTGGAGCGGATTCTGGAAGAAGAATCTGATGAGACACTTAAAATGACT	*240*
**NS 245467 Method K**	*181*	GGAAAGCAGATAGTGGAGCGGATTCTGGAAGAAGAATCTGATGAGACACTTAAAATGACT	*240*
**NS 245467 Method N**	*181*	GGAAAGCAGATAGTGGAGCGGATTCTGGAAGAAGAATCTGATGAGACACTTAAAATGACT	*240*

**NS 245467 MiSeq**	*241*	ATTGCCCCCGTGCCAGCTTCACGCTACCTAACTGACATGACTCTTGAGGAGATGTCAAGG	*300*
**NS 245467 Method A**	*241*	ATTGCCCCCGTGCCAGCTTCACGCTACCTAACTGACATGACTCTTGAGGAGATGTCAAGG	*300*
**NS 245467 Method S**	*241*	ATTGCCCCCGTGCCAGCTTCACGCTACCTAACTGACATGACTCTTGAGGAGATGTCAAGG	*300*
**NS 245467 Method E**	*241*	ATTGCCCCCGTGCCAGCTTCACGCTACCTAACTGACATGACTCTTGAGGAGATGTCAAGG	*300*
**NS 245467 Method K**	*241*	ATTGCCCCCGTGCCAGCTTCACGCTACCTAACTGACATGACTCTTGAGGAGATGTCAAGG	*300*
**NS 245467 Method N**	*241*	ATTGCCCCCGTGCCAGCTTCACGCTACCTAACTGACATGACTCTTGAGGAGATGTCAAGG	*300*

**NS 245467 MiSeq**	*301*	GACTGGTTCATGCTCATGCCCAAACAGAAAATGGCAGGTTCCCTTTGCATCAGAATGGAC	*360*
**NS 245467 Method A**	*301*	GACTGGTTCATGCTCATGCCCAAACAGAAAATGGCAGGTTCCCTTTGCATCAGAATGGAC	*360*
**NS 245467 Method S**	*301*	GACTGGTTCATGCTCATGCCCAAACAGAAAGTGGCAGGTTCCCTTTGCATCAGAATGGAC	*360*
**NS 245467 Method E**	*301*	GACTGGTTCATGCTCATGCCCAAACAGAAAATGGCAGGTTCCCTTTGCATCAGAATGGAC	*360*
**NS 245467 Method K**	*301*	GACTGGTTCATGCTCATGCCCAAACAGAAAATGGCAGGTTCCCTTTGCATCAGAATGGAC	*360*
**NS 245467 Method N**	*301*	GACTGGTTCATGCTCATGCCCAAACAGAAAATGGCAGGTTCCCTTTGCATCAGAATGGAC	*360*

**NS 245467 MiSeq**	*361*	CAGGCAATAATGGATAAAAACATCATATTGAAAGCAAACTTCAGTGTGATTTTTGACCGG	*420*
**NS 245467 Method A**	*361*	CAGGCAATAATGGATAAAAACATCATATTGAAAGCAAACTTCAGTGTGATTTTTGACCGG	*420*
**NS 245467 Method S**	*361*	CAGGCAATAATGGATAAAAACATCATATTGAAAGCAAACTTCAGTGTGATTTTTGACCGG	*420*
**NS 245467 Method E**	*361*	CAGGCAATAATGGATAAAAACATCATATTGAAAGCAAACTTCAGTGTGATTTTTGACCGG	*420*
**NS 245467 Method K**	*361*	CAGGCAATAATGGATAAAAACATCATATTGAAAGCAAACTTCAGTGTGATTTTTGACCGG	*420*
**NS 245467 Method N**	*361*	CAGGCAATAATGGATAAAAACATCATATTGAAAGCAAACTTCAGTGTGATTTTTGACCGG	*420*

**NS 245467 MiSeq**	*421*	CTGGAAACCCTAATACTACTTAGAGCTTTCACAGAAGAAGGAGCAATTGTGGGAGAAATC	*480*
**NS 245467 Method A**	*421*	CTGGAAACCCTAATACTACTTAGAGCTTTCACAGAAGAAGGAGCAATTGTGGGAGAAATC	*480*
**NS 245467 Method S**	*421*	CTGGAAACCCTAATACTACTTAGAGCTTTCACAGAAGAAGGAGCAATTGTGGGAGAAATC	*480*
**NS 245467 Method E**	*421*	CTGGAAACCCTAATACTACTTAGAGCTTTCACAGAAGAAGGAGCAATTGTGGGAGAAATC	*480*
**NS 245467 Method K**	*421*	CTGGAAACCCTAATACTACTTAGAGCTTTCACAGAAGAAGGAGCAATTGTGGGAGAAATC	*480*
**NS 245467 Method N**	*421*	CTGGAAACCCTAATACTACTTAGAGCTTTCACAGAAGAAGGAGCAATTGTGGGAGAAATC	*480*

**NS 245467 MiSeq**	*481*	TCACCATTACCTTCTCTTCCAGGACATACTGATGAGGATGTCAAAAATGCAATTGGGGTC	*540*
**NS 245467 Method A**	*481*	TCACCATTACCTTCTCTTCCAGGACATACTGATGAGGATGTCAAAAATGCAATTGGGGTC	*540*
**NS 245467 Method S**	*481*	TCACCATTACCTTCTCTTCCAGGACATACTGATGAGGATGTCAAAAATGCAATTGGGGTC	*540*
**NS 245467 Method E**	*481*	TCACCATTACCTTCTCTTCCAGGACATACTGATGAGGATGTCAAAAATGCAATTGGGGTC	*540*
**NS 245467 Method K**	*481*	TCACCATTACCTTCTCTTCCAGGACATACTGATGAGGATGTCAAAAATGCAATTGGGGTC	*540*
**NS 245467 Method N**	*481*	TCACCATTACCTTCTCTTCCAGGACATACTGATGAGGATGTCAAAAATGCAATTGGGGTC	*540*

**NS 245467 MiSeq**	*541*	CTCATCGGAGGACTTGAATGGAATGATAACACAGTTCGAGTCTCTGAAACTTTACAGAGA	*600*
**NS 245467 Method A**	*541*	CTCATCGGAGGACTTGAATGGAATGATAACACAGTTCGAGTCTCTGAAACTTTACAGAGA	*600*
**NS 245467 Method S**	*541*	CTCATCGGAGGACTTGAATGGAATGATAACACAGTTCGAGTCTCTGAAACT**C**TACAGAGA	*600*
**NS 245467 Method E**	*541*	CTCATCGGAGGACTTGAATGGAATGATAACACAGTTCGAGTCTCTGAAACTTTACAGAGA	*600*
**NS 245467 Method K**	*541*	CTCATCGGAGGACTTGAATGGAATGATAACACAGTTCGAGTCTCTGAAACTTTACAGAGA	*600*
**NS 245467 Method N**	*541*	CTCATCGGAGGACTTGAATGGAATGATAACACAGTTCGAGTCTCTGAAACTTTACAGAGA	*600*

**NS 245467 MiSeq**	*601*	TTCGCTTGGAGAAGCAGTAATGAGGATGGGAGACCTCCACTCCCTCCAAAGCAGAAACGG	*660*
**NS 245467 Method A**	*601*	TTCGCTTGGAGAAGCAGTAATGAGGATGGGAGACCTCCACTCCCTCCAAAGCAGAAACGG	*660*
**NS 245467 Method S**	*601*	TTCGCTTGGAGAAGCAGTAATGAGGATGGGAGACCTCCACTCCCTCCAAAGCAGAAACGG	*660*
**NS 245467 Method E**	*601*	TTCGCTTGGAGAAGCAGTAATGAGGATGGGAGACCTCCACTCCCTCCAAAGCAGAAACGG	*660*
**NS 245467 Method K**	*601*	TTCGCTTGGAGAAGCAGTAATGAGGATGGGAGACCTCCACTCCCTCCAAAGCAGAAACGG	*660*
**NS 245467 Method N**	*601*	TTCGCTTGGAGAAGCAGTAATGAGGATGGGAGACCTCCACTCCCTCCAAAGCAGAAACGG	*660*

**NS 245467 MiSeq**	*661*	AAAATGGCGAGAACAATTGAGTCAGAAGTTTGAAGAAATAAGATGGCTGATTGAAGGAGT	*720*
**NS 245467 Method A**	*661*	AAAATGGCGAGAACAATTGAGTCAGAAGTTTGAAGAAATAAGATGGCTGATTGAAGGAGT	*720*
**NS 245467 Method S**	*661*	AAAATGGCG**T**GAACAATTGAGTCAGAAGTTTGAAGAAATAAGATGGCTGATTGAAG**A**AGT	*720*
**NS 245467 Method E**	*661*	AAAATGGCGAGAACAATTGAGTCAGAAGTTTGAAGAAATAAGATGGCTGATTGAAGGAGT	*720*
**NS 245467 Method K**	*661*	AAAATGGCGAGAACAATTGAGTCAGAAGTTTGAAGAAATAAGATGGCTGATTGAAGGAGT	*720*
**NS 245467 Method N**	*661*	AAAATGGCGAGAACAATTGAGTCAGAAGTTTGAAGAAATAAGATGGCTGATTGAAGGAGT	*720*

**NS 245467 MiSeq**	*721*	GCGGCACAGATTGAAGATTACAGAGAACAGTTTCGAACAGATAACTTTTATGCAAGCCTT	*780*
**NS 245467 Method A**	*721*	GCGGCACAGATTGAAGATTACAGAGAACAGTTTCGAACAGATAACTTTTATGCAAGCCTT	*780*
**NS 245467 Method S**	*721*	GCGGCACAGATTGAAGATTACAGAGAACAGTTTCGAACAGATAACTTTTATGCAAGCCTT	*780*
**NS 245467 Method E**	*721*	GCGGCACAGATTGAAGATTACAGAGAACAGTTTCGAACAGATAACTTTTATGCAAGCCTT	*780*
**NS 245467 Method K**	*721*	GCGGCACAGATTGAAGATTACAGAGAACAGTTTCGAACAGATAACTTTTATGCAAGCCTT	*780*
**NS 245467 Method N**	*721*	GCGGCACAGATTGAAGATTACAGAGAACAGTTTCGAACAGATAACTTTTATGCAAGCCTT	*780*

**NS 245467 MiSeq**	*781*	ACAACTATTGCTTGAAGTGGAGCAAGAGATAAGAACTTTCTCGTTTCAGCTTATTTAA	*838*
**NS 245467 Method A**	*781*	ACAACTATTGCTTGAAGTGGAGCAAGAGATAAGAACTTTCTCGTTTCAGCTTATTTAA	*838*
**NS 245467 Method S**	*781*	ACAACTATTGCTTGAAGTGGAGCAAGAGATAAGAACTTTCTCGTTTCAGCTTATTTAA	*838*
**NS 245467 Method E**	*781*	ACAACTATTGCTTGAAGTGGAGCAAGAGATAAGAACTTTCTCGTTTCAGCTTATTTAA	*838*
**NS 245467 Method K**	*781*	ACAACTATTGCTTGAAGTGGAGCAAGAGATAAGAACTTTCTCGTTTCAGCTTATTTAA	*838*
**NS 245467 Method N**	*781*	ACAACTATTGCTTGAAGTGGAGCAAGAGATAAGAACTTTCTCGTTTCAGCTTATTTAA	*838*
