# Supplementary material for: An Evaluation of Avian Influenza Virus Whole-Genome Sequencing Approaches Using Nanopore Technology
Source: Microorganisms. 2023 Feb 19;11(2):529. doi: 10.3390/microorganisms11020529 (PMC9967579; doi:10.3390/microorganisms11020529)
Supplement: Supplementary file 1 [file microorganisms-11-00529-s001.zip › manuscript.v8 230219 Suppl Figures and Tables/Supplementary Figures S1a-h 245467/Supplementary Figure S1d H5.pdf]

Formatted Alignments

```
H5 245467 MiSeq       1  ATGGAGAACATAGTACTACTTCTTGCAATAGTTAGCCTTGTTAAAAGTGATCAGATTTGC  60
H5 245467 Method A    1  ATGGAGAACATAGTACTACTTCTTGCAATAGTTAGCCTTGTTAAAAGTGATCAGATTTGC  60
H5 245467 Method S    1  ATGGAGAACATAGTACTTCTTCTTGCAATAGTTAGCCTTGTTAAAAGTGATCAGATTTGC  60
H5 245467 Method E    1  ATGGAGAACATAGTACTACTTCTTGCAATAGTTAGCCTTGTTAAAAGTGATCAGATTTGC  60
H5 245467 Method K    1  ATGGAGAACATAGTACTACTTCTTGCAATAGTTAGCCTTGTTAAAAGTGATCAGATTTGC  60
H5 245467 Method N    1  ATGGAGAACATAGTACTACTTCTTGCAATAGTTAGCCTTGTTAAAAGTGATCAGATTTGC  60

H5 245467 MiSeq      61  ATTGGTTACCATGCAAACAATTCGACAGAGCAAGTTGACACGATAATGGAAAAGAATGTT  120
H5 245467 Method A   61  ATTGGTTACCATGCAAACAATTCGACAGAGCAAGTTGACACGATAATGGAAAAGAATGTT  120
H5 245467 Method S   61  ATTGGTTACCATGCAAACAATTCGACAGAGCAAGTTGACACGATAATGGAAAAGAACGTC  120
H5 245467 Method E   61  ATTGGTTACCATGCAAACAATTCGACAGAGCAAGTTGACACGATAATGGAAAAGAATGTT  120
H5 245467 Method K   61  ATTGGTTACCATGCAAACAATTCGACAGAGCAAGTTGACACGATAATGGAAAAGAATGTT  120
H5 245467 Method N   61  ATTGGTTACCATGCAAACAATTCGACAGAGCAAGTTGACACGATAATGGAAAAGAATGTT  120

H5 245467 MiSeq     121  ACTGTTACACATGCCCAAGACATACTGGAAAAAACACACAACGGGAAGCTCTGTGATCTA  180
H5 245467 Method A  121  ACTGTTACACATGCCCAAGACATACTGGAAAAAACACACAACGGGAAGCTCTGTGATCTA  180
H5 245467 Method S  121  ACTGTTACACATGCCCAAGACATACTGGAAAAAACACACAACGGGAAGCTCTGTGATCTA  180
H5 245467 Method E  121  ACTGTTACACATGCCCAAGACATACTGGAAAAAACACACAACGGGAAGCTCTGTGATCTA  180
H5 245467 Method K  121  ACTGTTACACATGCCCAAGACATACTGGAAAAAACACACAACGGGAAGCTCTGTGATCTA  180
H5 245467 Method N  121  ACTGTTACACATGCCCAAGACATACTGGAAAAAACACACAACGGGAAGCTCTGTGATCTA  180

H5 245467 MiSeq     181  AATGGGGTGAAGCCTCTGATTTTAAAGGATTGTAGTGTAGCTGGATGGCTCCTCGGAAAC  240
H5 245467 Method A  181  AATGGGGTGAAGCCTCTGATTTTAAAGGATTGTAGTGTAGCTGGATGGCTCCTCGGAAAC  240
H5 245467 Method S  181  AATGGGGTGAAGCCTCTGATTTTAAAGGATTGTAGTGTAGCTGGATGGCTCCTCGGAAAC  240
H5 245467 Method E  181  AATGGGGTGAAGCCTCTGATTTTAAAGGATTGTAGTGTAGCTGGATGGCTCCTCGGAAAC  240
H5 245467 Method K  181  AATGGGGTGAAGCCTCTGATTTTAAAGGATTGTAGTGTAGCTGGATGGCTCCTCGGAAAC  240
H5 245467 Method N  181  AATGGGGTGAAGCCTCTGATTTTAAAGGATTGTAGTGTAGCTGGATGGCTCCTCGGAAAC  240
```

**H5 245467 MiSeq**	*241*	CCAATGTGCGACGAATTCATCAGAGTGCCTGAATGGTCATACATAGTGGAGCGGGCTAAC	*300*
**H5 245467 Method A**	*241*	CCAATGTGCGACGAATTCATCAGAGTGCCTGAATGGTCATACATAGTGGAGCGGGCTAAC	*300*
**H5 245467 Method S**	*241*	CCAATGTGCGACGAATTCATCAGAGTGCCGGAATGGTCCTACATAGTGGAGCGGGCTAAT	*300*
**H5 245467 Method E**	*241*	CCAATGTGCGACGAATTCATCAGAGTGCCTGAATGGTCATACATAGTGGAGCGGGCTAAC	*300*
**H5 245467 Method K**	*241*	CCAATGTGCGACGAATTCATCAGAGTGCCTGAATGGTCATACATAGTGGAGCGGGCTAAC	*300*
**H5 245467 Method N**	*241*	CCAATGTGCGACGAATTCATCAGAGTGCCTGAATGGTCATACATAGTGGAGCGGGCTAAC	*300*

**H5 245467 MiSeq**	*301*	CCAGCTAATGACCTCTGTTACCCAGGGAGCCTCAATGACTATGAAGAACTGAAACACATG	*360*
**H5 245467 Method A**	*301*	CCAGCTAATGACCTCTGTTACCCAGGGAGCCTCAATGACTATGAAGAACTGAAACACATG	*360*
**H5 245467 Method S**	*301*	CCAGCTAATGACCTCTGTTACCCAGGGAGTCTCAATGACTATGAAGAACTGAAACACCTG	*360*
**H5 245467 Method E**	*301*	CCAGCTAATGACCTCTGTTACCCAGGGAGCCTCAATGACTATGAAGAACTGAAACACATG	*360*
**H5 245467 Method K**	*301*	CCAGCTAATGACCTCTGTTACCCAGGGAGCCTCAATGACTATGAAGAACTGAAACACATG	*360*
**H5 245467 Method N**	*301*	CCAGCTAATGACCTCTGTTACCCAGGGAGCCTCAATGACTATGAAGAACTGAAACACATG	*360*

**H5 245467 MiSeq**	*361*	TTGAGCAGAATAAATCATTTTGAGAAGATTCTGATCATCCCCAAGAGTTCCTGGCCAAAT	*420*
**H5 245467 Method A**	*361*	TTGAGCAGAATAAATCATTTTGAGAAGATTCTGATCATCCCCAAGAGTTCCTGGCCAAAT	*420*
**H5 245467 Method S**	*361*	TTGAGCAGAATAAATCATTTTGAGAAGATTCTGATCATCCCCAAGAGTTCCTGGCCAAAT	*420*
**H5 245467 Method E**	*361*	TTGAGCAGAATAAATCATTTTGAGAAGATTCTGATCATCCCCAAGAGTTCCTGGCCAAAT	*420*
**H5 245467 Method K**	*361*	TTGAGCAGAATAAATCATTTTGAGAAGATTCTGATCATCCCCAAGAGTTCCTGGCCAAAT	*420*
**H5 245467 Method N**	*361*	TTGAGCAGAATAAATCATTTTGAGAAGATTCTGATCATCCCCAAGAGTTCCTGGCCAAAT	*420*

**H5 245467 MiSeq**	*421*	CATGAAACATCACTAGGGGTGAGCGCAGCTTGTCCATACCAGGGAGCGCCCTCCTTTTTC	*480*
**H5 245467 Method A**	*421*	CATGAAACATCACTAGGGGTGAGCGCAGCTTGTCCATACCAGGGAGCGCCCTCCTTTTTC	*480*
**H5 245467 Method S**	*421*	CATGAAACATCACTAGGGGTGAGCGCAGCTTGTCCATACCAGGGAGCGCCCTCCTTTTTC	*480*
**H5 245467 Method E**	*421*	CATGAAACATCACTAGGGGTGAGCGCAGCTTGTCCATACCAGGGAGCGCCCTCCTTTTTC	*480*
**H5 245467 Method K**	*421*	CATGAAACATCACTAGGGGTGAGCGCAGCTTGTCCATACCAGGGAGCGCCCTCCTTTTTC	*480*
**H5 245467 Method N**	*421*	CATGAAACATCACTAGGGGTGAGCGCAGCTTGTCCATACCAGGGAGCGCCCTCCTTTTTC	*480*

**H5 245467 MiSeq**	*481*	AGAAATGTGGTGTGGCTTATCAAAAAGAACGATGCATACCCAACAATAAAGATAAGCTAC	*540*
**H5 245467 Method A**	*481*	AGAAATGTGGTGTGGCTTATCAAAAAGAACGATGCATACCCAACAATAAAGATAAGCTAC	*540*
**H5 245467 Method S**	*481*	AGAAATGTGGTGTGGCTTATCAAAAAGAACGATGCATACCCAACAATAAAGATAAGCTAC	*540*
**H5 245467 Method E**	*481*	AGAAATGTGGTGTGGCTTATCAAAAAGAACGATGCATACCCAACAATAAAGATAAGCTAC	*540*
**H5 245467 Method K**	*481*	AGAAATGTGGTGTGGCTTATCAAAAAGAACGATGCATACCCAACAATAAAGATAAGCTAC	*540*
**H5 245467 Method N**	*481*	AGAAATGTGGTGTGGCTTATCAAAAAGAACGATGCATACCCAACAATAAAGATAAGCTAC	*540*

**H5 245467 MiSeq**	*541*	AATAATACCAATCGGGAAGATCTCTTGATACTGTGGGGGATTCATCATTCCAACAATGCA	*600*
**H5 245467 Method A**	*541*	AATAATACCAATCGGGAAGATCTCTTGATACTGTGGGGGATTCATCATTCCAACAATGCA	*600*
**H5 245467 Method S**	*541*	AATAATACCAATC**A**GGAAGATCTCTTGATACTGTGGGGGATTCATCATTCCAACAATGCA	*600*
**H5 245467 Method E**	*541*	AATAATACCAATCGGGAAGATCTCTTGATACTGTGGGGGATTCATCATTCCAACAATGCA	*600*
**H5 245467 Method K**	*541*	AATAATACCAATCGGGAAGATCTCTTGATACTGTGGGGGATTCATCATTCCAACAATGCA	*600*
**H5 245467 Method N**	*541*	AATAATACCAATCGGGAAGATCTCTTGATACTGTGGGGGATTCATCATTCCAACAATGCA	*600*

**H5 245467 MiSeq**	*601*	GAAGAGCAGACAAATCTCTACAAAAACCCAACCACCTACATTTCAGTTGGAACATCAACT	*660*
**H5 245467 Method A**	*601*	GAAGAGCAGACAAATCTCTACAAAAACCCAACCACCTACATTTCAGTTGGAACATCAACT	*660*
**H5 245467 Method S**	*601*	GAAGAGCAGACAAATCTCTA**T**AAAAACCCAACCACCTACATTTCAGTTGGAACATCAACT	*660*
**H5 245467 Method E**	*601*	GAAGAGCAGACAAATCTCTACAAAAACCCAACCACCTACATTTCAGTTGGAACATCAACT	*660*
**H5 245467 Method K**	*601*	GAAGAGCAGACAAATCTCTACAAAAACCCAACCACCTACATTTCAGTTGGAACATCAACT	*660*
**H5 245467 Method N**	*601*	GAAGAGCAGACAAATCTCTACAAAAACCCAACCACCTACATTTCAGTTGGAACATCAACT	*660*

**H5 245467 MiSeq**	*661*	TTAAACCAGAGGTTGGCACCAAAAATAGCTACTAGATCCCAAGTAAACGGGCAACGTGGA	*720*
**H5 245467 Method A**	*661*	TTAAACCAGAGGTTGGCACCAAAAATAGCTACTAGATCCCAAGTAAACGGGCAACGTGGA	*720*
**H5 245467 Method S**	*661*	TTAAACCAGAGGTTGG**T**ACCAAAAATAGCTACTAGATCCCAAGTAAACGGGCAACGTGGA	*720*
**H5 245467 Method E**	*661*	TTAAACCAGAGGTTGGCACCAAAAATAGCTACTAGATCCCAAGTAAACGGGCAACGTGGA	*720*
**H5 245467 Method K**	*661*	TTAAACCAGAGGTTGGCACCAAAAATAGCTACTAGATCCCAAGTAAACGGGCAACGTGGA	*720*
**H5 245467 Method N**	*661*	TTAAACCAGAGGTTGGCACCAAAAATAGCTACTAGATCCCAAGTAAACGGGCAACGTGGA	*720*

**H5 245467 MiSeq**	*721*	AGAATGGACTTCTTCTGGACAATCTTAAAACCAGATGATGCAATCCATTTCGAGAGTAAT	*780*
**H5 245467 Method A**	*721*	AGAATGGACTTCTTCTGGACAATCTTAAAACCAGATGATGCAATCCATTTCGAGAGTAAT	*780*
**H5 245467 Method S**	*721*	AGAATGGACTTCTTCTGGACAATTTTAAAACCGGATGATGCAATCCATTTCGAGAGTAAT	*780*
**H5 245467 Method E**	*721*	AGAATGGACTTCTTCTGGACAATCTTAAAACCAGATGATGCAATCCATTTCGAGAGTAAT	*780*
**H5 245467 Method K**	*721*	AGAATGGACTTCTTCTGGACAATCTTAAAACCAGATGATGCAATCCATTTCGAGAGTAAT	*780*
**H5 245467 Method N**	*721*	AGAATGGACTTCTTCTGGACAATCTTAAAACCAGATGATGCAATCCATTTCGAGAGTAAT	*780*

**H5 245467 MiSeq**	*781*	GGAAATTTCATTGCTCCAGAATATGCATACAAAATTGTCAAGAAAGGGGACTCAACAATT	*840*
**H5 245467 Method A**	*781*	GGAAATTTCATTGCTCCAGAATATGCATACAAAATTGTCAAGAAAGGGGACTCAACAATT	*840*
**H5 245467 Method S**	*781*	GGAAATTTCATTGCTCCAGAATATGCATACAAAATTGTCAAGAAAGGGGACTCAACAATT	*840*
**H5 245467 Method E**	*781*	GGAAATTTCATTGCTCCAGAATATGCATACAAAATTGTCAAGAAAGGGGACTCAACAATT	*840*
**H5 245467 Method K**	*781*	GGAAATTTCATTGCTCCAGAATATGCATACAAAATTGTCAAGAAAGGGGACTCAACAATT	*840*
**H5 245467 Method N**	*781*	GGAAATTTCATTGCTCCAGAATATGCATACAAAATTGTCAAGAAAGGGGACTCAACAATT	*840*

**H5 245467 MiSeq**	*841*	ATGAAAAGTGGAGTGGAATATGGCCACTGCAACACCAAATGTCAAACCCCAGTAGGTGCG	*900*
**H5 245467 Method A**	*841*	ATGAAAAGTGGAGTGGAATATGGCCACTGCAACACCAAATGTCAAACCCCAGTAGGTGCG	*900*
**H5 245467 Method S**	*841*	ATGAAAAGTGGAGTGGAATATGGCCACTGCAACACCAAATGTCAAACCCCAGTAGGAGCG	*900*
**H5 245467 Method E**	*841*	ATGAAAAGTGGAGTGGAATATGGCCACTGCAACACCAAATGTCAAACCCCAGTAGGTGCG	*900*
**H5 245467 Method K**	*841*	ATGAAAAGTGGAGTGGAATATGGCCACTGCAACACCAAATGTCAAACCCCAGTAGGTGCG	*900*
**H5 245467 Method N**	*841*	ATGAAAAGTGGAGTGGAATATGGCCACTGCAACACCAAATGTCAAACCCCAGTAGGTGCG	*900*

**H5 245467 MiSeq**	*901*	ATAAATTCTAGTATGCCATTCCACAACATACATCCTCTCACCATTGGGGAATGCCCCAAA	*960*
**H5 245467 Method A**	*901*	ATAAATTCTAGTATGCCATTCCACAACATACATCCTCTCACCATTGGGGAATGCCCCAAA	*960*
**H5 245467 Method S**	*901*	ATAAATTCTAGTATGCCATTCCACAACATACATCCTCTCACCATTGGGGAATGCCCCAAA	*960*
**H5 245467 Method E**	*901*	ATAAATTCTAGTATGCCATTCCACAACATACATCCTCTCACCATTGGGGAATGCCCCAAA	*960*
**H5 245467 Method K**	*901*	ATAAATTCTAGTATGCCATTCCACAACATACATCCTCTCACCATTGGGGAATGCCCCAAA	*960*
**H5 245467 Method N**	*901*	ATAAATTCTAGTATGCCATTCCACAACATACATCCTCTCACCATTGGGGAATGCCCCAAA	*960*

**H5 245467 MiSeq**	*961*	TACGTGAAGTCAAACAAGTTGGTCCTTGCGACTGGGCTCAGAAATAGTCCTCTAAGAGAA	*1020*
**H5 245467 Method A**	*961*	TACGTGAAGTCAAACAAGTTGGTCCTTGCGACTGGGCTCAGAAATAGTCCTCTAAGAGAA	*1020*
**H5 245467 Method S**	*961*	TACGTGAAGTCAAACAAGTTGGTCCTTGCGACTGGGCTCAGAAATAGTCCTCTAAGAGAA	*1020*
**H5 245467 Method E**	*961*	TACGTGAAGTCAAACAAGTTGGTCCTTGCGACTGGGCTCAGAAATAGTCCTCTAAGAGAA	*1020*
**H5 245467 Method K**	*961*	TACGTGAAGTCAAACAAGTTGGTCCTTGCGACTGGGCTCAGAAATAGTCCTCTAAGAGAA	*1020*
**H5 245467 Method N**	*961*	TACGTGAAGTCAAACAAGTTGGTCCTTGCGACTGGGCTCAGAAATAGTCCTCTAAGAGAA	*1020*

**H5 245467 MiSeq**	*1021*	AAGAGAAGAAAAAGAGGCCTGTTTGGGGCGATAGCAGGGTTTATAGAGGGAGGATGGCAG	*1080*
**H5 245467 Method A**	*1021*	AAGAGAAGAAAAAGAGGCCTGTTTGGGGCGATAGCAGGGTTTATAGAGGGAGGATGGCAG	*1080*
**H5 245467 Method S**	*1021*	AAGAGAAGAAAAAGAGGCCTGTTTGGGGCGATAGCAGGGTTTATAGAGGGAGGATGGCAG	*1080*
**H5 245467 Method E**	*1021*	AAGAGAAGAAAAAGAGGCCTGTTTGGGGCGATAGCAGGGTTTATAGAGGGAGGATGGCAG	*1080*
**H5 245467 Method K**	*1021*	AAGAGAAGAAAAAGAGGCCTGTTTGGGGCGATAGCAGGGTTTATAGAGGGAGGATGGCAG	*1080*
**H5 245467 Method N**	*1021*	AAGAGAAGAAAAAGAGGCCTGTTTGGGGCGATAGCAGGGTTTATAGAGGGAGGATGGCAG	*1080*

**H5 245467 MiSeq**	*1081*	GGAATGGTTGATGGTTGGTATGGGTACCATCATAGCAATGAGCAGGGGAGTGGGTACGCT	*1140*
**H5 245467 Method A**	*1081*	GGAATGGTTGATGGTTGGTATGGGTACCATCATAGCAATGAGCAGGGGAGTGGGTACGCT	*1140*
**H5 245467 Method S**	*1081*	GGAATGGTTGATGGTTGGTATGGGTACCATCATAGCAATGAGCAGGGGAGTGGGTACGCT	*1140*
**H5 245467 Method E**	*1081*	GGAATGGTTGATGGTTGGTATGGGTACCATCATAGCAATGAGCAGGGGAGTGGGTACGCT	*1140*
**H5 245467 Method K**	*1081*	GGAATGGTTGATGGTTGGTATGGGTACCATCATAGCAATGAGCAGGGGAGTGGGTACGCT	*1140*
**H5 245467 Method N**	*1081*	GGAATGGTTGATGGTTGGTATGGGTACCATCATAGCAATGAGCAGGGGAGTGGGTACGCT	*1140*

**H5 245467 MiSeq**	*1141*	GCGGACAAAGAATCCACCCAAAAGGCAATAGATGGAGTTACCAATAAGGTCAACTCAATT	*1200*
**H5 245467 Method A**	*1141*	GCGGACAAAGAATCCACCCAAAAGGCAATAGATGGAGTTACCAATAAGGTCAACTCAATT	*1200*
**H5 245467 Method S**	*1141*	GCAGACAAAGAATCCACCCAAAAGGCAATAGATGGAGTTACCAATAAGGTCAACTCAATC	*1200*
**H5 245467 Method E**	*1141*	GCGGACAAAGAATCCACCCAAAAGGCAATAGATGGAGTTACCAATAAGGTCAACTCAATT	*1200*
**H5 245467 Method K**	*1141*	GCGGACAAAGAATCCACCCAAAAGGCAATAGATGGAGTTACCAATAAGGTCAACTCAATT	*1200*
**H5 245467 Method N**	*1141*	GCGGACAAAGAATCCACCCAAAAGGCAATAGATGGAGTTACCAATAAGGTCAACTCAATT	*1200*

**H5 245467 MiSeq**	*1201*	ATTGACAAAATGAACACTCAATTTGAGGCAGTTGGAAGGGAGTTTAATAACTTAGAAAGG	*1260*
**H5 245467 Method A**	*1201*	ATTGACAAAATGAACACTCAATTTGAGGCAGTTGGAAGGGAGTTTAATAACTTAGAAAGG	*1260*
**H5 245467 Method S**	*1201*	ATTGACAAAATGAACACTCAATTTGAGGCAGTTGGAAGGGAGTTTAATAACTTAGAAAGG	*1260*
**H5 245467 Method E**	*1201*	ATTGACAAAATGAACACTCAATTTGAGGCAGTTGGAAGGGAGTTTAATAACTTAGAAAGG	*1260*
**H5 245467 Method K**	*1201*	ATTGACAAAATGAACACTCAATTTGAGGCAGTTGGAAGGGAGTTTAATAACTTAGAAAGG	*1260*
**H5 245467 Method N**	*1201*	ATTGACAAAATGAACACTCAATTTGAGGCAGTTGGAAGGGAGTTTAATAACTTAGAAAGG	*1260*

**H5 245467 MiSeq**	*1261*	AGGATAGAGAATTTGAACAAGAAAATGGAAGACGGATTCCTAGATGTCTGGACCTATAAT	*1320*
**H5 245467 Method A**	*1261*	AGGATAGAGAATTTGAACAAGAAAATGGAAGACGGATTCCTAGATGTCTGGACCTATAAT	*1320*
**H5 245467 Method S**	*1261*	AGGATAGAGAATTTGAACAAGAAAATGGAAGACGGATTCCTAGATGTCTGGACCTATAAT	*1320*
**H5 245467 Method E**	*1261*	AGGATAGAGAATTTGAACAAGAAAATGGAAGACGGATTCCTAGATGTCTGGACCTATAAT	*1320*
**H5 245467 Method K**	*1261*	AGGATAGAGAATTTGAACAAGAAAATGGAAGACGGATTCCTAGATGTCTGGACCTATAAT	*1320*
**H5 245467 Method N**	*1261*	AGGATAGAGAATTTGAACAAGAAAATGGAAGACGGATTCCTAGATGTCTGGACCTATAAT	*1320*

**H5 245467 MiSeq**	*1321*	GCTGAACTTCTAGTTCTCATGGAAAACGAGAGGACTCTAGATTTCCATGATTCAAATGTC	*1380*
**H5 245467 Method A**	*1321*	GCTGAACTTCTAGTTCTCATGGAAAACGAGAGGACTCTAGATTTCCATGATTCAAATGTC	*1380*
**H5 245467 Method S**	*1321*	GCTGAACTTCTAGTTCTCATGGAAAACGAGAGGACTCTAGATTTCCATGATTCAAATGTC	*1380*
**H5 245467 Method E**	*1321*	GCTGAACTTCTAGTTCTCATGGAAAACGAGAGGACTCTAGATTTCCATGATTCAAATGTC	*1380*
**H5 245467 Method K**	*1321*	GCTGAACTTCTAGTTCTCATGGAAAACGAGAGGACTCTAGATTTCCATGATTCAAATGTC	*1380*
**H5 245467 Method N**	*1321*	GCTGAACTTCTAGTTCTCATGGAAAACGAGAGGACTCTAGATTTCCATGATTCAAATGTC	*1380*

**H5 245467 MiSeq**	*1381*	AAGAACCTTTACGACAAAGTCAGATTACAGCTTAGGGATAATGCAAAGGAGCTGGGTAAC	*1440*
**H5 245467 Method A**	*1381*	AAGAACCTTTACGACAAAGTCAGATTACAGCTTAGGGATAATGCAAAGGAGCTGGGTAAC	*1440*
**H5 245467 Method S**	*1381*	AAGAACCTTTACGACAAAGTCAGACTACAGCTTAGGGATAATGCAAAGGAGCTGGGTAAC	*1440*
**H5 245467 Method E**	*1381*	AAGAACCTTTACGACAAAGTCAGATTACAGCTTAGGGATAATGCAAAGGAGCTGGGTAAC	*1440*
**H5 245467 Method K**	*1381*	AAGAACCTTTACGACAAAGTCAGATTACAGCTTAGGGATAATGCAAAGGAGCTGGGTAAC	*1440*
**H5 245467 Method N**	*1381*	AAGAACCTTTACGACAAAGTCAGATTACAGCTTAGGGATAATGCAAAGGAGCTGGGTAAC	*1440*

**H5 245467 MiSeq**	*1441*	GGCTGTTTCGAATTCTATCACAAATGTGATAATGAATGTATGGAAAGTGTGAGAAATGGG	*1500*
**H5 245467 Method A**	*1441*	GGCTGTTTCGAATTCTATCACAAATGTGATAATGAATGTATGGAAAGTGTGAGAAATGGG	*1500*
**H5 245467 Method S**	*1441*	GGCTGTTTCGAATTCTATCACAAATGCGATAATGAATGTATGGAAAGTGTGAGAAATGGG	*1500*
**H5 245467 Method E**	*1441*	GGCTGTTTCGAATTCTATCACAAATGTGATAATGAATGTATGGAAAGTGTGAGAAATGGG	*1500*
**H5 245467 Method K**	*1441*	GGCTGTTTCGAATTCTATCACAAATGTGATAATGAATGTATGGAAAGTGTGAGAAATGGG	*1500*
**H5 245467 Method N**	*1441*	GGCTGTTTCGAATTCTATCACAAATGTGATAATGAATGTATGGAAAGTGTGAGAAATGGG	*1500*

**H5 245467 MiSeq**	*1501*	ACATATGACTACCCTCAGTATTCAGAAGAAGCAAGATTAAAAAGAGAAGAAATAAGCGGA	*1560*
**H5 245467 Method A**	*1501*	ACATATGACTACCCTCAGTATTCAGAAGAAGCAAGATTAAAAAGAGAAGAAATAAGCGGA	*1560*
**H5 245467 Method S**	*1501*	ACGTATGACTACCCTCAGTATTCAGAAGAAGCAAGATTAAAAAGAGAAGAAATAAGCGGA	*1560*
**H5 245467 Method E**	*1501*	ACATATGACTACCCTCAGTATTCAGAAGAAGCAAGATTAAAAAGAGAAGAAATAAGCGGA	*1560*
**H5 245467 Method K**	*1501*	ACATATGACTACCCTCAGTATTCAGAAGAAGCAAGATTAAAAAGAGAAGAAATAAGCGGA	*1560*
**H5 245467 Method N**	*1501*	ACATATGACTACCCTCAGTATTCAGAAGAAGCAAGATTAAAAAGAGAAGAAATAAGCGGA	*1560*

**H5 245467 MiSeq**	*1561*	GTGAAATTAGAATCAGTAGGAACTTACCAGATACTGTCAATTTATTCAACAGCGGCAAGT	*1620*
**H5 245467 Method A**	*1561*	GTGAAATTAGAATCAGTAGGAACTTACCAGATACTGTCAATTTATTCAACAGCGGCAAGT	*1620*
**H5 245467 Method S**	*1561*	GTGAAATTAGAATCAATAGGAACTTACCAGATACTGTCAATTTATTCAACAGCGGCGAGT	*1620*
**H5 245467 Method E**	*1561*	GTGAAATTAGAATCAGTAGGAACTTACCAGATACTGTCAATTTATTCAACAGCGGCAAGT	*1620*
**H5 245467 Method K**	*1561*	GTGAAATTAGAATCAGTAGGAACTTACCAGATACTGTCAATTTATTCAACAGCGGCAAGT	*1620*
**H5 245467 Method N**	*1561*	GTGAAATTAGAATCAGTAGGAACTTACCAGATACTGTCAATTTATTCAACAGCGGCAAGT	*1620*

**H5 245467 MiSeq**	*1621*	TCCCTAGCACTGGCAATCATGATGGCTGGTCTATCTTTATGGATGTGCTCCAATGGGTCG	*1680*
**H5 245467 Method A**	*1621*	TCCCTAGCACTGGCAATCATGATGGCTGGTCTATCTTTATGGATGTGCTCCAATGGGTCG	*1680*
**H5 245467 Method S**	*1621*	TCCCTAGCACTGGCAATCATGATGGCTGGTCTATCTTTATGGATGTGCTCCAATGGGTCG	*1680*
**H5 245467 Method E**	*1621*	TCCCTAGCACTGGCAATCATGATGGCTGGTCTATCTTTATGGATGTGCTCCAATGGGTCG	*1680*
**H5 245467 Method K**	*1621*	TCCCTAGCACTGGCAATCATGATGGCTGGTCTATCTTTATGGATGTGCTCCAATGGGTCG	*1680*
**H5 245467 Method N**	*1621*	TCCCTAGCACTGGCAATCATGATGGCTGGTCTATCTTTATGGATGTGCTCCAATGGGTCG	*1680*

**H5 245467 MiSeq**	*1681*	TTACAGTGCAGAATTTGCATTTAG	*1704*
**H5 245467 Method A**	*1681*	TTACAGTGCAGAATTTGCATTTAG	*1704*
**H5 245467 Method S**	*1681*	TTACAGTGCAGAATTTGCATTTAA	*1704*
**H5 245467 Method E**	*1681*	TTACAGTGCAGAATTTGCATTTAG	*1704*
**H5 245467 Method K**	*1681*	TTACAGTGCAGAATTTGCATTTAG	*1704*
**H5 245467 Method N**	*1681*	TTACAGTGCAGAATTTGCATTTAG	*1704*
