# Supplementary material for: An Evaluation of Avian Influenza Virus Whole-Genome Sequencing Approaches Using Nanopore Technology
Source: Microorganisms. 2023 Feb 19;11(2):529. doi: 10.3390/microorganisms11020529 (PMC9967579; doi:10.3390/microorganisms11020529)
Supplement: Supplementary file 1 [file microorganisms-11-00529-s001.zip › manuscript.v8 230219 Suppl Figures and Tables/Supplementary Figures S1a-h 245467/Supplementary Figure S1g MA.pdf]

Formatted Alignments

MA 245467 MiSeq	1	ATGAGTCTTCTAACCGAGGTCGAAACGTACGTTCTCTCTATCGTCCCGTCGGGCCCCCTC	60
MA 245467 Method A	1	ATGAGTCTTCTAACCGAGGTCGAAACGTACGTTCTCTCTATCGTCCCGTCGGGCCCCCTC	60
MA 245467 Method S	1	ATGAGTCTTCTAACCGAGGTCGAAACGTACGTTCTCTCTATCGTCCCGTCGGGCCCCCTC	60
MA 245467 Method E	1	ATGAGTCTTCTAACCGAGGTCGAAACGTACGTTCTCTCTATCGTCCCGTCGGGCCCCCTC	60
MA 245467 Method K	1	ATGAGTCTTCTAACCGAGGTCGAAACGTACGTTCTCTCTATCGTCCCGTCGGGCCCCCTC	60
MA 245467 Method N	1	ATGAGTCTTCTAACCGAGGTCGAAACGTACGTTCTCTCTATCGTCCCGTCGGGCCCCCTC	60
MA 245467 MiSeq	61	AAAGCCGAGATCGCGCAGAGACTTGAAGATGTCTTTGCAGGGAAGAACACCGATCTTGAG	120
MA 245467 Method A	61	AAAGCCGAGATCGCGCAGAGACTTGAAGATGTCTTTGCAGGGAAGAACACCGATCTTGAG	120
MA 245467 Method S	61	AAAGCCGAGATCGCGCAGAGACTTGAAGATGTCTTTGCAGGGAAGAACACCGATCTTGAG	120
MA 245467 Method E	61	AAAGCCGAGATCGCGCAGAGACTTGAAGATGTCTTTGCAGGGAAGAACACCGATCTTGAG	120
MA 245467 Method K	61	AAAGCCGAGATCGCGCAGAGACTTGAAGATGTCTTTGCAGGGAAGAACACCGATCTTGAG	120
MA 245467 Method N	61	AAAGCCGAGATCGCGCAGAGACTTGAAGATGTCTTTGCAGGGAAGAACACCGATCTTGAG	120
MA 245467 MiSeq	121	GCTCTCATGGAATGGCTAAAGACAAGACCAATCCTGTCACCTCTGACTAAGGGGATTTTG	180
MA 245467 Method A	121	GCTCTCATGGAATGGCTAAAGACAAGACCAATCCTGTCACCTCTGACTAAGGGGATTTTG	180
MA 245467 Method S	121	GCTCTCATGGAATGGCTAAAGACAAGACCAATCCTGTCACCTCTGACTAAGGGGATTTTG	180
MA 245467 Method E	121	GCTCTCATGGAATGGCTAAAGACAAGACCAATCCTGTCACCTCTGACTAAGGGGATTTTG	180
MA 245467 Method K	121	GCTCTCATGGAATGGCTAAAGACAAGACCAATCCTGTCACCTCTGACTAAGGGGATTTTG	180
MA 245467 Method N	121	GCTCTCATGGAATGGCTAAAGACAAGACCAATCCTGTCACCTCTGACTAAGGGGATTTTG	180
MA 245467 MiSeq	181	GGATTTGTGTTCACGCTCACCGTGCCCAGTGAGCGAGGACTGCAGCGTAGACGCTTTGTC	240
MA 245467 Method A	181	GGATTTGTGTTCACGCTCACCGTGCCCAGTGAGCGAGGACTGCAGCGTAGACGCTTTGTC	240
MA 245467 Method S	181	GGATTTGTGTTCACGCTCACCGTGCCCAGTGAGCGAGGACTGCAGCGTAGACGCTTTGTC	240
MA 245467 Method E	181	GGATTTGTGTTCACGCTCACCGTGCCCAGTGAGCGAGGACTGCAGCGTAGACGCTTTGTC	240
MA 245467 Method K	181	GGATTTGTGTTCACGCTCACCGTGCCCAGTGAGCGAGGACTGCAGCGTAGACGCTTTGTC	240
MA 245467 Method N	181	GGATTTGTGTTCACGCTCACCGTGCCCAGTGAGCGAGGACTGCAGCGTAGACGCTTTGTC	240

**MA 245467 MiSeq**	*241*	CAAAATGCTCTAAATGGAAATGGAGACCCAAACAACATGGACAGGGCAGTCAAGTTGTAC	*300*
**MA 245467 Method A**	*241*	CAAAATGCTCTAAATGGAAATGGAGACCCAAACAACATGGACAGGGCAGTCAAGTTGTAC	*300*
**MA 245467 Method S**	*241*	CAAAATGCTCTAAATGGAAATGGAGACCCAAACAACATGGACAGGGCAGTCAAACTGTAC	*300*
**MA 245467 Method E**	*241*	CAAAATGCTCTAAATGGAAATGGAGACCCAAACAACATGGACAGGGCAGTCAAGTTGTAC	*300*
**MA 245467 Method K**	*241*	CAAAATGCTCTAAATGGAAATGGAGACCCAAACAACATGGACAGGGCAGTCAAGTTGTAC	*300*
**MA 245467 Method N**	*241*	CAAAATGCTCTAAATGGAAATGGAGACCCAAACAACATGGACAGGGCAGTCAAGTTGTAC	*300*

**MA 245467 MiSeq**	*301*	AGGAAATTGAAGAGAGAGATAACATTCCATGGGGCTAAAGAAGTTGCACTCAGTTACTCA	*360*
**MA 245467 Method A**	*301*	AGGAAATTGAAGAGAGAGATAACATTCCATGGGGCTAAAGAAGTTGCACTCAGTTACTCA	*360*
**MA 245467 Method S**	*301*	AGGAAATTGAAGAGAGAGATAACATTCCATGGGGCTAAAGAAGTTGCACTCAGTTACTCA	*360*
**MA 245467 Method E**	*301*	AGGAAATTGAAGAGAGAGATAACATTCCATGGGGCTAAAGAAGTTGCACTCAGTTACTCA	*360*
**MA 245467 Method K**	*301*	AGGAAATTGAAGAGAGAGATAACATTCCATGGGGCTAAAGAAGTTGCACTCAGTTACTCA	*360*
**MA 245467 Method N**	*301*	AGGAAATTGAAGAGAGAGATAACATTCCATGGGGCTAAAGAAGTTGCACTCAGTTACTCA	*360*

**MA 245467 MiSeq**	*361*	ACCGGTGCACTTGCCAGTTGTATGGGTCTCATATACAACAGGATGGGGACGGTGACCGCA	*420*
**MA 245467 Method A**	*361*	ACCGGTGCACTTGCCAGTTGTATGGGTCTCATATACAACAGGATGGGGACGGTGACCGCA	*420*
**MA 245467 Method S**	*361*	ACCGGTGCACTTGCCAGTTGTATGGGTCTCATATACAACAGGATGGGGACGGTGACCGCA	*420*
**MA 245467 Method E**	*361*	ACCGGTGCACTTGCCAGTTGTATGGGTCTCATATACAACAGGATGGGGACGGTGACCGCA	*420*
**MA 245467 Method K**	*361*	ACCGGTGCACTTGCCAGTTGTATGGGTCTCATATACAACAGGATGGGGACGGTGACCGCA	*420*
**MA 245467 Method N**	*361*	ACCGGTGCACTTGCCAGTTGTATGGGTCTCATATACAACAGGATGGGGACGGTGACCGCA	*420*

**MA 245467 MiSeq**	*421*	GAAGTGGCATTGGGCCTAGTGTGTGCCACCTGTGAGCAGATTGCTGATTCACAGCATCGG	*480*
**MA 245467 Method A**	*421*	GAAGTGGCATTGGGCCTAGTGTGTGCCACCTGTGAGCAGATTGCTGATTCACAGCATCGG	*480*
**MA 245467 Method S**	*421*	GAAGTGGCATTGGGCCTAGTGTGTGCCACCTGTGAGCAGATTGCTGATTCACAGCATCGG	*480*
**MA 245467 Method E**	*421*	GAAGTGGCATTGGGCCTAGTGTGTGCCACCTGTGAGCAGATTGCTGATTCACAGCATCGG	*480*
**MA 245467 Method K**	*421*	GAAGTGGCATTGGGCCTAGTGTGTGCCACCTGTGAGCAGATTGCTGATTCACAGCATCGG	*480*
**MA 245467 Method N**	*421*	GAAGTGGCATTGGGCCTAGTGTGTGCCACCTGTGAGCAGATTGCTGATTCACAGCATCGG	*480*

MA 245467 MiSeq	481	TCTCACAGACAGATAGCTACCACCACCAACCCACTGATCAGACATGAAAACAGAATGGTG	540
MA 245467 Method A	481	TCTCACAGACAGATAGCTACCACCACCAACCCACTGATCAGACATGAAAACAGAATGGTG	540
MA 245467 Method S	481	TCTCACAGACAGATAGCTACCACCACCAACCCACTAATCAGACATGAAAACAGAATGGTG	540
MA 245467 Method E	481	TCTCACAGACAGATAGCTACCACCACCAACCCACTGATCAGACATGAAAACAGAATGGTG	540
MA 245467 Method K	481	TCTCACAGACAGATAGCTACCACCACCAACCCACTGATCAGACATGAAAACAGAATGGTG	540
MA 245467 Method N	481	TCTCACAGACAGATAGCTACCACCACCAACCCACTGATCAGACATGAAAACAGAATGGTG	540
MA 245467 MiSeq	541	TTGGCCAGTACTACAGCTAAGGCTATGGAGCAGATGGCTGGATCGAGTGAGCAAGCAGTG	600
MA 245467 Method A	541	TTGGCCAGTACTACAGCTAAGGCTATGGAGCAGATGGCTGGATCGAGTGAGCAAGCAGTG	600
MA 245467 Method S	541	TTGGCCAGTACTACAGCTAAGGCTATGGAGCAGATGGCTGGATCGAGTGAGCAAGCAGCG	600
MA 245467 Method E	541	TTGGCCAGTACTACAGCTAAGGCTATGGAGCAGATGGCTGGATCGAGTGAGCAAGCAGTG	600
MA 245467 Method K	541	TTGGCCAGTACTACAGCTAAGGCTATGGAGCAGATGGCTGGATCGAGTGAGCAAGCAGTG	600
MA 245467 Method N	541	TTGGCCAGTACTACAGCTAAGGCTATGGAGCAGATGGCTGGATCGAGTGAGCAAGCAGTG	600
MA 245467 MiSeq	601	GAGGCCATGGAGGTTGCTAGTCAGGCTAGGCAGATGGTGCAGGCGATGAGGACCATTGGA	660
MA 245467 Method A	601	GAGGCCATGGAGGTTGCTAGTCAGGCTAGGCAGATGGTGCAGGCGATGAGGACCATTGGA	660
MA 245467 Method S	601	GAAGCCATGGAGGTTGCTAGTCAGGCTAGGCAGATGGTGCAGGCGATGAGGACCATTGGA	660
MA 245467 Method E	601	GAGGCCATGGAGGTTGCTAGTCAGGCTAGGCAGATGGTGCAGGCGATGAGGACCATTGGA	660
MA 245467 Method K	601	GAGGCCATGGAGGTTGCTAGTCAGGCTAGGCAGATGGTGCAGGCGATGAGGACCATTGGA	660
MA 245467 Method N	601	GAGGCCATGGAGGTTGCTAGTCAGGCTAGGCAGATGGTGCAGGCGATGAGGACCATTGGA	660
MA 245467 MiSeq	661	ACTCATCCTAGCTCCAGTGCCGGTCTGAGAGATGATCTCCTTGAAAATTTGCAGGCCTAC	720
MA 245467 Method A	661	ACTCATCCTAGCTCCAGTGCCGGTCTGAGAGATGATCTCCTTGAAAATTTGCAGGCCTAC	720
MA 245467 Method S	661	ACTCATCCTAGCTCCAGTGCCGGTCTGAGAGATGATCTCCTTGAAAATTTGCAGGCCTAC	720
MA 245467 Method E	661	ACTCATCCTAGCTCCAGTGCCGGTCTGAGAGATGATCTCCTTGAAAATTTGCAGGCCTAC	720
MA 245467 Method K	661	ACTCATCCTAGCTCCAGTGCCGGTCTGAGAGATGATCTCCTTGAAAATTTGCAGGCCTAC	720
MA 245467 Method N	661	ACTCATCCTAGCTCCAGTGCCGGTCTGAGAGATGATCTCCTTGAAAATTTGCAGGCCTAC	720

**MA 245467 MiSeq**	*721*	CAAAAACGGATGGGAGTGCAACTGCAGCGATTCAAGTGATCCTCTCGTTATTGCCGCAAG	*780*
**MA 245467 Method A**	*721*	CAAAAACGGATGGGAGTGCAACTGCAGCGATTCAAGTGATCCTCTCGTTATTGCCGCAAG	*780*
**MA 245467 Method S**	*721*	CAAAAACGGATGGGAGTGCAACTGCAGCGATTCAAGTGATCCTCTCGTTATTGCCGCAAG	*780*
**MA 245467 Method E**	*721*	CAAAAACGGATGGGAGTGCAACTGCAGCGATTCAAGTGATCCTCTCGTTATTGCCGCAAG	*780*
**MA 245467 Method K**	*721*	CAAAAACGGATGGGAGTGCAACTGCAGCGATTCAAGTGATCCTCTCGTTATTGCCGCAAG	*780*
**MA 245467 Method N**	*721*	CAAAAACGGATGGGAGTGCAACTGCAGCGATTCAAGTGATCCTCTCGTTATTGCCGCAAG	*780*

**MA 245467 MiSeq**	*781*	TATCATTGGGATCTTGCACTTGATATTGTGGATTCTTGATCGCCTTTTCTTCAAATGCGT	*840*
**MA 245467 Method A**	*781*	TATCATTGGGATCTTGCACTTGATATTGTGGATTCTTGATCGCCTTTTCTTCAAATGCGT	*840*
**MA 245467 Method S**	*781*	TATCATTGGGATCTTGCACTTGATATTGTGGATTCTTGATCGCCTTTTCTTCAAATGCGT	*840*
**MA 245467 Method E**	*781*	TATCATTGGGATCTTGCACTTGATATTGTGGATTCTTGATCGCCTTTTCTTCAAATGCGT	*840*
**MA 245467 Method K**	*781*	TATCATTGGGATCTTGCACTTGATATTGTGGATTCTTGATCGCCTTTTCTTCAAATGCGT	*840*
**MA 245467 Method N**	*781*	TATCATTGGGATCTTGCACTTGATATTGTGGATTCTTGATCGCCTTTTCTTCAAATGCGT	*840*

**MA 245467 MiSeq**	*841*	TTATCGTCGCCTTAAATACGGTTTGAAAGGAGGGCCTTCTACGGAAGGAGTACCTGAGTC	*900*
**MA 245467 Method A**	*841*	TTATCGTCGCCTTAAATACGGTTTGAAAGGAGGGCCTTCTACGGAAGGAGTACCTGAGTC	*900*
**MA 245467 Method S**	*841*	TTATCGTCGCCTTAAATACGGTTTGAAA**A**GAGGGCCTTCTACGGAAGGAGTACCTGAGTC	*900*
**MA 245467 Method E**	*841*	TTATCGTCGCCTTAAATACGGTTTGAAAGGAGGGCCTTCTACGGAAGGAGTACCTGAGTC	*900*
**MA 245467 Method K**	*841*	TTATCGTCGCCTTAAATACGGTTTGAAAGGAGGGCCTTCTACGGAAGGAGTACCTGAGTC	*900*
**MA 245467 Method N**	*841*	TTATCGTCGCCTTAAATACGGTTTGAAAGGAGGGCCTTCTACGGAAGGAGTACCTGAGTC	*900*

**MA 245467 MiSeq**	*901*	CATGAGGGAAGAGTACCGGCAGGAACAGCAGAGTGCTGTGGATGTTGACGATGGTCATTT	*960*
**MA 245467 Method A**	*901*	CATGAGGGAAGAGTACCGGCAGGAACAGCAGAGTGCTGTGGATGTTGACGATGGTCATTT	*960*
**MA 245467 Method S**	*901*	CATGAGGGAAGAGTACCGGCAGGAACAGCAGAGTGCTGTGGATGTTGACGATGGTCATTT	*960*
**MA 245467 Method E**	*901*	CATGAGGGAAGAGTACCGGCAGGAACAGCAGAGTGCTGTGGATGTTGACGATGGTCATTT	*960*
**MA 245467 Method K**	*901*	CATGAGGGAAGAGTACCGGCAGGAACAGCAGAGTGCTGTGGATGTTGACGATGGTCATTT	*960*
**MA 245467 Method N**	*901*	CATGAGGGAAGAGTACCGGCAGGAACAGCAGAGTGCTGTGGATGTTGACGATGGTCATTT	*960*

**MA 245467 MiSeq**	*961*	TGTCAACATAGAGCTGGAGTAA	*982*
**MA 245467 Method A**	*961*	TGTCAACATAGAGCTGGAGTAA	*982*
**MA 245467 Method S**	*961*	TGTCAACATAGAGCTGGAGTAA	*982*
**MA 245467 Method E**	*961*	TGTCAACATAGAGCTGGAGTAA	*982*
**MA 245467 Method K**	*961*	TGTCAACATAGAGCTGGAGTAA	*982*
**MA 245467 Method N**	*961*	TGTCAACATAGAGCTGGAGTAA	*982*
