# Supplementary material for: An Evaluation of Avian Influenza Virus Whole-Genome Sequencing Approaches Using Nanopore Technology
Source: Microorganisms. 2023 Feb 19;11(2):529. doi: 10.3390/microorganisms11020529 (PMC9967579; doi:10.3390/microorganisms11020529)
Supplement: Supplementary file 1 [file microorganisms-11-00529-s001.zip › manuscript.v8 230219 Suppl Figures and Tables/Supplementary Figures S2a-h 245626/Supplementary Figure S2c PA.pdf]

Formatted Alignments

**PA 245625 MiSeq**	*1*	ATGGAAGACTTTGTGCGACAATGCTTCAATCCAATGATTGTCGAGCTTGCGGAGAAAGCA	*60*
**PA 245626 Method A**	*1*	ATGGAAGACTTTGTGCGACAATGCTTCAATCCAATGATTGTCGAGCTTGCGGAGAAAGCA	*60*
**PA 245626 Method K**	*1*	ATGGAAGACTTTGTGCGACAATGCTTCAATCCAATGATTGTCGAGCTTGCGGAGAAAGCA	*60*
**PA 245626 Method N**	*1*	ATGGAAGATTTTGTGCGACAATGCTTCAATCCAATGATTGTCGAGCTTGCGGAGAAAGCA	*60*
**PA 245625 MiSeq**	*61*	ATGAAAGAATATGGGGAAGATCCGAAAATCGAGACAAACAAATTTGCCGCAATATGCACA	*120*
**PA 245626 Method A**	*61*	ATGAAAGAATATGGGGAAGATCCGAAAATCGAGACAAACAAATTTGCCGCAATATGCACA	*120*
**PA 245626 Method K**	*61*	ATGAAAGAATATGGGGAAGATCCGAAAATCGAGACAAACAAATTTGCCGCAATATGCACA	*120*
**PA 245626 Method N**	*61*	ATGAAAGAATATGGGGAAGATCCGAAAATCGAGACAAACAAATTTGCCGCAATATGCACA	*120*
**PA 245625 MiSeq**	*121*	CACTTAGAAGTCTGTTTCATGTATTCGGATTTCCATTTTATTGATGAACGAGGCGAATCA	*180*
**PA 245626 Method A**	*121*	CACTTAGAAGTCTGTTTCATGTATTCGGATTTCCATTTTATTGATGAACGAGGCGAATCA	*180*
**PA 245626 Method K**	*121*	CACTTAGAAGTCTGTTTCATGTATTCGGATTTCCATTTTATTGATGAACGAGGCGAATCA	*180*
**PA 245626 Method N**	*121*	CACTTAGAAGTCTGTTTCATGTATTCGGATTTCCATTTTATTGATGAACGAGGCGAATCA	*180*
**PA 245625 MiSeq**	*181*	ATGATTGTAGAATCTGGCGATCCAAATGCATTATTGAAACACCGATTTGAGATAATCGAA	*240*
**PA 245626 Method A**	*181*	ATGATTGTAGAATCTGGCGATCCAAATGCATTATTGAAACACCGATTTGAGATAATCGAA	*240*
**PA 245626 Method K**	*181*	ATGATTGTAGAATCTGGCGATCCAAATGCATTATTGAAACACCGATTTGAGATAATCGAA	*240*
**PA 245626 Method N**	*181*	ATGATTGTAGAATCTGGCGATCCAAATGCATTATTGAAACACCGATTTGAGATAATCGAA	*240*
**PA 245625 MiSeq**	*241*	GGGAGAGACCGAGCAATGGCCTGGACAGTGGTGAATAGTATCTGCAACACCACAGGGGTC	*300*
**PA 245626 Method A**	*241*	GGGAGAGACCGAGCAATGGCCTGGACAGTGGTGAATAGTATCTGCAACACCACAGGGGTC	*300*
**PA 245626 Method K**	*241*	GGGAGAGACCGAGCAATGGCCTGGACAGTGGTGAATAGTATCTGCAACACCACAGGGGTC	*300*
**PA 245626 Method N**	*241*	GGGAGAGACCGAGCAATGGCCTGGACAGTGGTGAATAGTATCTGCAACACCACAGGGGTC	*300*
**PA 245625 MiSeq**	*301*	GAAAAGCCCAAATTCCTCCCTGATTTGTATGACTACAAAGAGAACCGATTCATTGAAATT	*360*
**PA 245626 Method A**	*301*	GAAAAGCCCAAATTCCTCCCTGATTTGTATGACTACAAAGAGAACCGATTCATTGAAATT	*360*
**PA 245626 Method K**	*301*	GAAAAGCCCAAATTCCTCCCTGATTTGTATGACTACAAAGAGAACCGATTCATTGAAATT	*360*
**PA 245626 Method N**	*301*	GAAAAGCCCAAATTCCTCCCTGATTTGTATGACTACAAAGAGAACCGATTCATTGAAATT	*360*

PA 245625 MiSeq	361	GGAGTAACGCGAAGGGAAGTTCACATATACTATTTAGAAAAAGCCAACAAGATAAAATCA	420
PA 245626 Method A	361	GGAGTAACGCGAAGGGAAGTTCACATATACTATTTAGAAAAAGCCAACAAGATAAAATCA	420
PA 245626 Method K	361	GGAGTAACGCGAAGGGAAGTTCACATATACTATTTAGAAAAAGCCAACAAGATAAAATCA	420
PA 245626 Method N	361	GGAGTAACGCGAAGGGAAGTTCACATATACTATTTAGAAAAAGCCAACAAGATAAAATCA	420
PA 245625 MiSeq	421	GAGAAAACACATATTCACATATTCTCATTCACTGGAGAGGAAATGGCCACCAAGGCGGAC	480
PA 245626 Method A	421	GAGAAAACACATATTCACATATTCTCATTCACTGGAGAGGAAATGGCCACCAAGGCGGAC	480
PA 245626 Method K	421	GAGAAAACACATATTCACATATTCTCATTCACTGGAGAGGAAATGGCCACCAAGGCGGAC	480
PA 245626 Method N	421	GAGAAAACACATATTCACATATTCTCATTCACTGGAGAGGAAATGGCCACCAAGGCGGAC	480
PA 245625 MiSeq	481	TACACCCTTGATGAAGAGAGCAGAGCAAGAATAAAAACCAGACTGTTCACTATAAGACAA	540
PA 245626 Method A	481	TACACCCTTGATGAAGAGAGCAGAGCAAGAATAAAAACCAGACTGTTCACTATAAGACAA	540
PA 245626 Method K	481	TACACCCTTGATGAAGAGAGCAGAGCAAGAATAAAAACCAGACTGTTCACTATAAGACAA	540
PA 245626 Method N	481	TACACCCTTGATGAAGAGAGCAGAGCAAGAATAAAAACCAGACTGTTCACTATAAGACAA	540
PA 245625 MiSeq	541	GAAATAGCCAGCAGAGGTCTATGGGATTCCTTTCGTCAGTCCGAGAGAGGCGAAGAGACA	600
PA 245626 Method A	541	GAAATAGCCAGCAGAGGTCTATGGGATTCCTTTCGTCAGTCCGAGAGAGGCGAAGAGACA	600
PA 245626 Method K	541	GAAATAGCCAGCAGAGGTCTATGGGATTCCTTTCGTCAGTCCGAGAGAGGCGAAGAGACA	600
PA 245626 Method N	541	GAAATAGCCAGCAGAGGTCTATGGGATTCCTTTCGTCAGTCCGAGAGAGGCGAAGAGACA	600
PA 245625 MiSeq	601	ATTGAAGAAAGATTTGAAATCACAGGAACCATGCGCAGGCTTGCCGACCAAAGTCTTCCA	660
PA 245626 Method A	601	ATTGAAGAAAGATTTGAAATCACAGGAACCATGCGCAGGCTTGCCGACCAAAGTCTTCCA	660
PA 245626 Method K	601	ATTGAAGAAAGATTTGAAATCACAGGAACCATGCGCAGGCTTGCCGACCAAAGTCTTCCA	660
PA 245626 Method N	601	ATTGAAGAAAGATTTGAAATCACAGGAACCATGCGCAGGCTTGCCGACCAAAGTCTTCCA	660
PA 245625 MiSeq	661	CCGAACTTCTCCAGCCTTGAAAACTTTAGAGCCTATGTGGATGGATTCGAACCGAACGGC	720
PA 245626 Method A	661	CCGAACTTCTCCAGCCTTGAAAACTTTAGAGCCTATGTGGATGGATTCGAACCGAACGGC	720
PA 245626 Method K	661	CCGAACTTCTCCAGCCTTGAAAACTTTAGAGCCTATGTGGATGGATTCGAACCGAACGGC	720
PA 245626 Method N	661	CCGAACTTCTCCAGCCTTGAAAACTTTAGAGCCTATGTGGATGGATTCGAACCGAACGGC	720

```
PA 245625 MiSeq     721  TGCATTGAGGGCAAACTTTCTCAAATGTCAAAAGAAGTGAACGCCAGAATTGAGCCATTT  780
PA 245626 Method A  721  TGCATTGAGGGCAAACTTTCTCAAATGTCAAAAGAAGTGAACGCCAGAATTGAGCCATTT  780
PA 245626 Method K  721  TGCATTGAGGGCAAACTTTCTCAAATGTCAAAAGAAGTGAACGCCAGAATTGAGCCATTT  780
PA 245626 Method N  721  TGCATTGAGGGCAAACTTTCTCAAATGTCAAAAGAAGTGAACGCCAGAATTGAGCCATTT  780

PA 245625 MiSeq     781  CTGAAGACAACACCACGCCCTCTCAGATTACCTGATGGGCCTCCCTGTTCTCAGCGGTCG  840
PA 245626 Method A  781  CTGAAGACAACACCACGCCCTCTCAGATTACCTGATGGGCCTCCCTGTTCTCAGCGGTCG  840
PA 245626 Method K  781  CTGAAGACAACACCACGCCCTCTCAGATTACCTGATGGGCCTCCCTGTTCTCAGCGGTCG  840
PA 245626 Method N  781  CTGAAGACAACACCACGCCCTCTCAGATTACCTGATGGGCCTCCCTGTTCTCAGCGGTCG  840

PA 245625 MiSeq     841  AAGTTCTTGCTGATGGATGCCCTTAAGTTGAGCATCGAAGACCCTAGCCATGAGGGGGAG  900
PA 245626 Method A  841  AAGTTCTTGCTGATGGATGCCCTTAAGTTGAGCATCGAAGACCCTAGCCATGAGGGG[AG]G  900
PA 245626 Method K  841  AAGTTCTTGCTGATGGATGCCCTTAAGTTGAGCATCGAAGACCCTAGCCATGAGGGGGAG  900
PA 245626 Method N  841  AAGTTCTTGCTGATGGATGCCCTTAAGTTGAGCATCGAAGACCCTAGCCATGAGGGGGAG  900

PA 245625 MiSeq     901  GGCATACCGCTGTATGATGCAATCAAATGCATGAAGACATTTTTTGGCTGGAAAGAGCCC  960
PA 245626 Method A  901  GGCATACCGCTGTATGATGCAATCAAATGCATGAAGACATTTTTTGGCTGGAAAGAGCCC  960
PA 245626 Method K  901  GGCATACCGCTGTATGATGCAATCAAATGCATGAAGACATTTTTTGGCTGGAAAGAGCCC  960
PA 245626 Method N  901  GGCATACCGCTGTATGATGCAATCAAATGCATGAAGACATTTTTTGGCTGGAAAGAGCCC  960

PA 245625 MiSeq     961  AACATCGTAAAGCCGCATGAGAAAGGCATAAACCCTAATTACCTCCTGGCTTGGAAGCAG  1020
PA 245626 Method A  961  AACATCGTAAAGCCGCATGAGAAAGGCATAAACCCTAATTACCTCCTGGCTTGGAAGCAG  1020
PA 245626 Method K  961  AACATCGTAAAGCCGCATGAGAAAGGCATAAACCCTAATTACCTCCTGGCTTGGAAGCAG  1020
PA 245626 Method N  961  AACATCGTAAAGCCGCATGAGAAAGGCATAAACCCTAATTACCTCCTGGCTTGGAAGCAG  1020

PA 245625 MiSeq     1021 GTGCTGGCAGAACTTCAAGACATTGAAAATGAGGAGAAAATTCCAAAAACAAAGAACATG  1080
PA 245626 Method A  1021 GTGCTGGCAGAACTTCAAGACATTGAAAATGAGGAGAAAATTCCAAAAACAAAGAACATG  1080
PA 245626 Method K  1021 GTGCTGGCAGAACTTCAAGACATTGAAAATGAGGAGAAAATTCCAAAAACAAAGAACATG  1080
PA 245626 Method N  1021 GTGCTGGCAGAACTTCAAGACATTGAAAATGAGGAGAAAATTCCAAAAACAAAGAACATG  1080
```

**PA 245625 MiSeq**	*1081*	AAGAAAACAAGCCAATTGAAGTGGGCACTTGGTGAGAACATGGCTCCAGAAAAAGTGGAC	*1140*
**PA 245626 Method A**	*1081*	AAGAAAACAAGCCAATTGAAGTGGGCACTTGGTGAGAACATGGCTCCAGAAAAAGTGGAC	*1140*
**PA 245626 Method K**	*1081*	AAGAAAACAAGCCAATTGAAGTGGGCACTTGGTGAGAACATGGCTCCAGAAAAAGTGGAC	*1140*
**PA 245626 Method N**	*1081*	AAGAAAACAAGCCAATTGAAGTGGGCACTTGGTGAGAACATGGCTCCAGAAAAAGTGGAC	*1140*
**PA 245625 MiSeq**	*1141*	TTTGAGGACTGCAAAGATGTTAGCGATCTAAGACAGTACGACAGTGACGAACCAGAGTCT	*1200*
**PA 245626 Method A**	*1141*	TTTGAGGACTGCAAAGATGTTAGCGATCTAAGACAGTACGACAGTGACGAACCAGAGTCT	*1200*
**PA 245626 Method K**	*1141*	TTTGAGGACTGCAAAGATGTTAGCGATCTAAGACAGTACGACAGTGACGAACCAGAGTCT	*1200*
**PA 245626 Method N**	*1141*	TTTGAGGACTGCAAAGATGTTAGCGATCTAAGACAGTACGACAGTGACGAACCAGAGTCT	*1200*
**PA 245625 MiSeq**	*1201*	AGATCACTAGCAAGCTGGATTCAGAGTGAATTCAACAAGGCATGCGAACTGACAGATTCG	*1260*
**PA 245626 Method A**	*1201*	AGATCACTAGCAAGCTGGATTCAGAGTGAATTCAACAAGGCATGCGAACTGACAGATTCG	*1260*
**PA 245626 Method K**	*1201*	AGATCACTAGCAAGCTGGATTCAGAGTGAATTCAACAAGGCATGCGAACTGACAGATTCG	*1260*
**PA 245626 Method N**	*1201*	AGATCACTAGCAAGCTGGATTCAGAGTGAATTCAACAAGGCATGCGAACTGACAGATTCG	*1260*
**PA 245625 MiSeq**	*1261*	AGTTGGATTGAACTTGATGAGATAGGGGAAGACGTTGCTCCAATCGAACACATTGCGAGT	*1320*
**PA 245626 Method A**	*1261*	AGTTGGATTGAACTTGATGAGATAGGGGAAGACGTTGCTCCAATCGAACACATTGCGAGT	*1320*
**PA 245626 Method K**	*1261*	AGTTGGATTGAACTTGATGAGATAGGGGAAGACGTTGCTCCAATCGAACACATTGCGAGT	*1320*
**PA 245626 Method N**	*1261*	AGTTGGATTGAACTTGATGAGATAGGGGAAGACGTTGCTCCAATCGAACACATTGCGAGT	*1320*
**PA 245625 MiSeq**	*1321*	GTGAGGAGGAACTATTTCACAGCGGAGGTATCCCATTGCAGGGCCACTGAATACATAATG	*1380*
**PA 245626 Method A**	*1321*	GTGAGGAGGAACTATTTCACAGCGGAGGTATCCCATTGCAGGGCCACTGAATACATAATG	*1380*
**PA 245626 Method K**	*1321*	GTGAGGAGGAACTATTTCACAGCGGAGGTATCCCATTGCAGGGCCACTGAATACATAATG	*1380*
**PA 245626 Method N**	*1321*	GTGAGGAGGAACTATTTCACAGCGGAGGTATCCCATTGCAGGGCCACTGAATACATAATG	*1380*
**PA 245625 MiSeq**	*1381*	AAGGGAGTATACATAAACACAGCCCTATTGAATGCATCCTGTGCAGCCATGGATGACTTC	*1440*
**PA 245626 Method A**	*1381*	AAGGGAGTATACATAAACACAGCCCTATTGAATGCATCCTGTGCAGCCATGGATGACTTC	*1440*
**PA 245626 Method K**	*1381*	AAGGGAGTATACATAAACACAGCCCTATTGAATGCATCCTGTGCAGCCATGGATGACTTC	*1440*
**PA 245626 Method N**	*1381*	AAGGGAGTATACATAAACACAGCCCTATTGAATGCATCCTGTGCAGCCATGGATGACTTC	*1440*

**PA 245625 MiSeq**	*1441*	CAACTGATTCCAATGATAAGTAAGTGCAGAACTAAAGAAGGAAGACGGAAGACAAATCTG	*1500*
**PA 245626 Method A**	*1441*	CAACTGATTCCAATGATAAGTAAGTGCAGAACTAAAGAAGGAAGACGGAAGACAAATCTG	*1500*
**PA 245626 Method K**	*1441*	CAACTGATTCCAATGATAAGTAAGTGCAGAACTAAAGAAGGAAGACGGAAGACAAATCTG	*1500*
**PA 245626 Method N**	*1441*	CAACTGATTCCAATGATAAGTAAGTGCAGAACTAAAGAAGGAAGACGGAAGACAAATCTG	*1500*

**PA 245625 MiSeq**	*1501*	TATGGATTCATTATAAAAGGAAGATCCCATTTGAGGAATGACACCGATGTGGTAAACTTT	*1560*
**PA 245626 Method A**	*1501*	TATGGATTCATTATAAAAGGAAGATCCCATTTGAGGAATGACACCGATGTGGTAAACTTT	*1560*
**PA 245626 Method K**	*1501*	TATGGATTCATTATAAAAGGAAGATCCCATTTGAGGAATGACACCGATGTGGTAAACTTT	*1560*
**PA 245626 Method N**	*1501*	TATGGATTCATTATAAAAGGAAGATCCCATTTGAGGAATGACACCGATGTGGTAAACTTT	*1560*

**PA 245625 MiSeq**	*1561*	GTGAGCATGGAATTCTCTCTAACTGACCCGAGGCTAGAGCCACACAAATGGGAAAAGTAC	*1620*
**PA 245626 Method A**	*1561*	GTGAGCATGGAATTCTCTCTAACTGACCCGAGGCTAGAGCCACACAAATGGGAAAAGTAC	*1620*
**PA 245626 Method K**	*1561*	GTGAGCATGGAATTCTCTCTAACTGACCCGAGGCTAGAGCCACACAAATGGGAAAAGTAC	*1620*
**PA 245626 Method N**	*1561*	GTGAGCATGGAATTCTCTCTAACTGACCCGAGGCTAGAGCCACACAAATGGGAAAAGTAC	*1620*

**PA 245625 MiSeq**	*1621*	TGTGTTCTTGAGATAGGAGACATGCTCCTACGGACTGCAATAGGCCAAGTGTCGAGGCCC	*1680*
**PA 245626 Method A**	*1621*	TGTGTTCTTGAGATAGGAGACATGCTCCTACGGACTGCAATAGGCCAAGTGTCGAGGCCC	*1680*
**PA 245626 Method K**	*1621*	TGTGTTCTTGAGATAGGAGACATGCTCCTACGGACTGCAATAGGCCAAGTGTCGAGGCCC	*1680*
**PA 245626 Method N**	*1621*	TGTGTTCTTGAGATAGGAGACATGCTCCTACGGACTGCAATAGGCCAAGTGTCGAGGCCC	*1680*

**PA 245625 MiSeq**	*1681*	ATGTTCCTGTATGTGAGAACCAATGGGACTTCCAAGATCAAAATGAAATGGGGCATGGAG	*1740*
**PA 245626 Method A**	*1681*	ATGTTCCTGTATGTGAGAACCAATGGGACTTCCAAGATCAAAATGAAATGGGGCATGGAG	*1740*
**PA 245626 Method K**	*1681*	ATGTTCCTGTATGTGAGAACCAATGGGACTTCCAAGATCAAAATGAAATGGGGCATGGAG	*1740*
**PA 245626 Method N**	*1681*	ATGTTCCTGTATGTGAGAACCAATGGGACTTCCAAGATCAAAATGAAATGGGGCATGGAG	*1740*

**PA 245625 MiSeq**	*1741*	ATGAGGCGATGCCTTCTTCAGTCCCTTCAACAAATTGAGAGCATGATTGAGGCCGAATCT	*1800*
**PA 245626 Method A**	*1741*	ATGAGGCGATGCCTTCTTCAGTCCCTTCAACAAATTGAGAGCATGATTGAGGCCGAATCT	*1800*
**PA 245626 Method K**	*1741*	ATGAGGCGATGCCTTCTTCAGTCCCTTCAACAAATTGAGAGCATGATTGAGGCCGAATCT	*1800*
**PA 245626 Method N**	*1741*	ATGAGGCGATGCCTTCTTCAGTCCCTTCAACAAATTGAGAGCATGATTGAGGCCGAATCT	*1800*

**PA 245625 MiSeq**	*1801*	TCTGTCAAAGAGAAGGACATGTCCAAGGAATTCTTTGAAAACAAATCAGAAACATGGCCA	*1860*
**PA 245626 Method A**	*1801*	TCTGTCAAAGAGAAGGACATGTCCAAGGAATTCTTTGAAAACAAATCAGAAACATGGCCA	*1860*
**PA 245626 Method K**	*1801*	TCTGTCAAAGAGAAGGACATGTCCAAGGAATTCTTTGAAAACAAATCAGAAACATGGCCA	*1860*
**PA 245626 Method N**	*1801*	TCTGTCAAAGAGAAGGACATGTCCAAGGAATTCTTTGAAAACAAATCAGAAACATGGCCA	*1860*
**PA 245625 MiSeq**	*1861*	ATTGGAGAATCACCCAAAGGGGTGGAGGAAGGCTCTATTGGGAAAGTATGCAGAACATTG	*1920*
**PA 245626 Method A**	*1861*	ATTGGAGAATCACCCAAAGGGGTGGAGGAAGGCTCTATTGGGAAAGTATGCAGAACATTG	*1920*
**PA 245626 Method K**	*1861*	ATTGGAGAATCACCCAAAGGGGTGGAGGAAGGCTCTATTGGGAAAGTATGCAGAACATTG	*1920*
**PA 245626 Method N**	*1861*	ATTGGAGAATCACCCAAAGGGGTGGAGGAAGGCTCTATTGGGAAAGTATGCAGAACATTG	*1920*
**PA 245625 MiSeq**	*1921*	CTAGCAAAGTCTGTGTTCAACAGCCTATATGCATCTCCACAACTCGAGGGGTTTTCAGCT	*1980*
**PA 245626 Method A**	*1921*	CTAGCAAAGTCTGTGTTCAACAGCCTATATGCATCTCCACAGCTCGAGGGGTTTTCAGCT	*1980*
**PA 245626 Method K**	*1921*	CTAGCAAAGTCTGTGTTCAACAGCCTATATGCATCTCCACAACTCGAGGGGTTTTCAGCT	*1980*
**PA 245626 Method N**	*1921*	CTAGCAAAGTCTGTGTTCAACAGCCTATATGCATCTCCACAACTCGAGGGGTTTTCAGCT	*1980*
**PA 245625 MiSeq**	*1981*	GAATCAAGAAAATTGCTTCTCATTGTTCAGGCACTTAGGGACAACCTGGGACCTGGAACC	*2040*
**PA 245626 Method A**	*1981*	GAATCAAGAAAATTGCTTCTCATTGTTCAGGCACTTAGGGACAACCTGGGACCTGGAACC	*2040*
**PA 245626 Method K**	*1981*	GAATCAAGAAAATTGCTTCTCATTGTTCAGGCACTTAGGGACAACCTGGGACCTGGAACC	*2040*
**PA 245626 Method N**	*1981*	GAATCAAGAAAATTGCTTCTCATTGTTCAGGCACTTAGGGACAACCTGGGACCTGGAACC	*2040*
**PA 245625 MiSeq**	*2041*	TTCGATCTTGGGGGGCTATATGAAGCAATTGAGGAGTGCCTGATTAACGATCCCTGGGTT	*2100*
**PA 245626 Method A**	*2041*	TTCGATCTTGGGGGGCTATATGAAGCAATTGAGGAGTGCCTGATTAACGATCCCTGGGTT	*2100*
**PA 245626 Method K**	*2041*	TTCGATCTTGGGGGGCTATATGAAGCAATTGAGGAGTGCCTGATTAACGATCCCTGGGTT	*2100*
**PA 245626 Method N**	*2041*	TTCGATCTTGGGGGGCTATATGAAGCAATTGAGGAGTGCCTGATTAACGATCCCTGGGTT	*2100*
**PA 245625 MiSeq**	*2101*	TTGCTTAATGCGTCTTGGTTCAACTCCTTCCTCACACATGCACTGAAATAG	*2151*
**PA 245626 Method A**	*2101*	TTGCTTAATGCGTCTTGGTTCAACTCCTTCCTCACACATGCACTGAAATAG	*2151*
**PA 245626 Method K**	*2101*	TTGCTTAATGCGTCTTGGTTCAACTCCTTCCTCACACATGCACTGAAATAG	*2151*
**PA 245626 Method N**	*2101*	TTGCTTAATGCGTCTTGGTTCAACTCCTTCCTCACACATGCACTGAAATAG	*2151*
