# Supplementary material for: An Evaluation of Avian Influenza Virus Whole-Genome Sequencing Approaches Using Nanopore Technology
Source: Microorganisms. 2023 Feb 19;11(2):529. doi: 10.3390/microorganisms11020529 (PMC9967579; doi:10.3390/microorganisms11020529)
Supplement: Supplementary file 1 [file microorganisms-11-00529-s001.zip › manuscript.v8 230219 Suppl Figures and Tables/Supplementary Figures S2a-h 245626/Supplementary Figure S2e NP.pdf]

Formatted Alignments

NP 245625 MiSeq	1	ATGGCGTCTCAAGGCACCAAACGACCCTATGAACAAATGGAAACTGGTGGGGAACGCCAG	60
NP 245626 Method A	1	ATGGCGTCTCAAGGCACCAAACGACCCTATGAACAAATGGAAACTGGTGGGGAACGCCAG	60
NP 245626 Method K	1	ATGGCGTCTCAAGGCACCAAACGACCCTATGAACAAATGGAAACTGGTGGGGAACGCCAG	60
NP 245626 Method N	1	ATGGCGTCTCAAGGCACCAAACGACCCTATGAACAAATGGAAACTGGTGGGGAACGCCAG	60
NP 245625 MiSeq	61	AATGCCACTGAAATCAGAGCATCTGTTGGAAGAATGGTTGGCGGAATCGGGAGATTCTAC	120
NP 245626 Method A	61	AATGCCACTGAAATCAGAGCATCTGTTGGAAGAATGGTTGGCGGAATCGGGAGATTCTAC	120
NP 245626 Method K	61	AATGCCACTGAAATCAGAGCATCTGTTGGAAGAATGGTTGGCGGAATCGGGAGATTCTAC	120
NP 245626 Method N	61	AATGCCACTGAAATCAGAGCATCTGTTGGAAGAATGGTTGGCGGAATCGGGAGATTCTAC	120
NP 245625 MiSeq	121	ATACAGATGTGCACTGAGCTCAAACTCAGTGATTACGAAGGGAGGCTGATCCAAAACAGC	180
NP 245626 Method A	121	ATACAGATGTGCACTGAGCTCAAACTCAGTGATTACGAAGGGAGGCTGATCCAAAACAGC	180
NP 245626 Method K	121	ATACAGATGTGCACTGAGCTCAAACTCAGTGATTACGAAGGGAGGCTGATCCAAAACAGC	180
NP 245626 Method N	121	ATACAGATGTGCACTGAGCTCAAACTCAGTGATTACGAAGGGAGGCTGATCCAAAACAGC	180
NP 245625 MiSeq	181	ATAACCATAGAAAGGATGGTTCTCTCGGCATTTGATGAGAGGAGGAACAAGTACCTGGAG	240
NP 245626 Method A	181	ATAACCATAGAAAGGATGGTTCTCTCGGCATTTGATGAGAGGAGGAACAAGTACCTGGAG	240
NP 245626 Method K	181	ATAACCATAGAAAGGATGGTTCTCTCGGCATTTGATGAGAGGAGGAACAAGTACCTGGAG	240
NP 245626 Method N	181	ATAACCATAGAAAGGATGGTTCTCTCGGCATTTGATGAGAGGAGGAACAAGTACCTGGAG	240
NP 245625 MiSeq	241	GAACATCCCAGTGCCGGGAAGGATCCCAAGAAGACTGGAGGTCCAATCTACAGGAGGAGA	300
NP 245626 Method A	241	GAACATCCCAGTGCCGGGAAGGATCCCAAGAAGACTGGAGGTCCAATCTACAGGAGGAGA	300
NP 245626 Method K	241	GAACATCCCAGTGCCGGGAAGGATCCCAAGAAGACTGGAGGTCCAATCTACAGGAGGAGA	300
NP 245626 Method N	241	GAACATCCCAGTGCCGGGAAGGATCCCAAGAAGACTGGAGGTCCAATCTACAGGAGGAGA	300
NP 245625 MiSeq	301	GATGGCAAATGGGTGAGAGAGTTGATCCTCTACGACAAAGAAGAGATCAGAAGAATTTGG	360
NP 245626 Method A	301	GATGGCAAATGGGTGAGAGAGTTGATCCTCTACGACAAAGAAGAGATCAGAAGAATTTGG	360
NP 245626 Method K	301	GATGGCAAATGGGTGAGAGAGTTGATCCTCTACGACAAAGAAGAGATCAGAAGAATTTGG	360
NP 245626 Method N	301	GATGGCAAATGGGTGAGAGAGTTGATCCTCTACGACAAAGAAGAGATCAGAAGAATTTGG	360

NP 245625 MiSeq	361	CGTCAAGCTAATAATGGAGAGGATGCAACTGCTGGTCTCACTCATTTGATGATTTGGCAT	420
NP 245626 Method A	361	CGTCAAGCTAATAATGGAGAGGATGCAACTGCTGGTCTCACTCATTTGATGATTTGGCAT	420
NP 245626 Method K	361	CGTCAAGCTAATAATGGAGAGGATGCAACTGCTGGTCTCACTCATTTGATGATTTGGCAT	420
NP 245626 Method N	361	CGTCAAGCTAATAATGGAGAGGATGCAACTGCTGGTCTCACTCATTTGATGATTTGGCAT	420
NP 245625 MiSeq	421	TCCAATCTGAATGATGCCACATACCAGAGAACAAGGGCACTTGTGCGTACTGGAATGGAC	480
NP 245626 Method A	421	TCCAATCTGAATGATGCCACATACCAGAGAACAAGGGCACTTGTGCGTACTGGAATGGAC	480
NP 245626 Method K	421	TCCAATCTGAATGATGCCACATACCAGAGAACAAGGGCACTTGTGCGTACTGGAATGGAC	480
NP 245626 Method N	421	TCCAATCTGAATGATGCCACATACCAGAGAACAAGGGCACTTGTGCGTACTGGAATGGAC	480
NP 245625 MiSeq	481	CCTAGAATGTGCTCTCTGATGCAAGGCTCAACCCTCCCTAGGAGATCCGGGGCTGCTGGA	540
NP 245626 Method A	481	CCTAGAATGTGCTCTCTGATGCAAGGCTCAACCCTCCCTAGGAGATCCGGGGCTGCTGGA	540
NP 245626 Method K	481	CCTAGAATGTGCTCTCTGATGCAAGGCTCAACCCTCCCTAGGAGATCCGGGGCTGCTGGA	540
NP 245626 Method N	481	CCTAGAATGTGCTCTCTGATGCAAGGCTCAACCCTCCCTAGGAGATCCGGGGCTGCTGGA	540
NP 245625 MiSeq	541	GCAGCAGTGAAAGGAGTTGGAACAATGGTGATGGAACTAATTCGGATGATCAAACGAGGG	600
NP 245626 Method A	541	GCAGCAGTGAAAGGAGTTGGAACAATGGTGATGGAACTAATTCGGATGATCAAACGAGGG	600
NP 245626 Method K	541	GCAGCAGTGAAAGGAGTTGGAACAATGGTGATGGAACTAATTCGGATGATCAAACGAGGG	600
NP 245626 Method N	541	GCAGCAGTGAAAGGAGTTGGAACAATGGTGATGGAACTAATTCGGATGATCAAACGAGGG	600
NP 245625 MiSeq	601	ATCAATGATCGGAATTTCTGGAGAGGCGAAAACGGTCGGAGAACCAGGATTGCCTACGAG	660
NP 245626 Method A	601	ATCAATGATCGGAATTTCTGGAGAGGCGAAAACGGTCGGAGAACCAGGATTGCCTACGAG	660
NP 245626 Method K	601	ATCAATGATCGGAATTTCTGGAGAGGCGAAAACGGTCGGAGAACCAGGATTGCCTACGAG	660
NP 245626 Method N	601	ATCAATGATCGGAATTTCTGGAGAGGCGAAAACGGTCGGAGAACCAGGATTGCCTACGAG	660
NP 245625 MiSeq	661	AGAATGTGCAACGTCCTCAAGGGAAAGTTCCAAACAGCAGCACAACGGGCAATGATGGAC	720
NP 245626 Method A	661	AGAATGTGCAACGTCCTCAAGGGAAAGTTCCAAACAGCAGCACAACGGGCAATGATGGAC	720
NP 245626 Method K	661	AGAATGTGCAACGTCCTCAAGGGAAAGTTCCAAACAGCAGCACAACGGGCAATGATGGAC	720
NP 245626 Method N	661	AGAATGTGCAACGTCCTCAAGGGAAAGTTCCAAACAGCAGCACAACGGGCAATGATGGAC	720

**NP 245625 MiSeq**	*721*	CAAGTGAGAGAAAGCCGGAATCCTGGGAATGCTGAAATTGAAGATCTCATCTTTCTCGCC	*780*
**NP 245626 Method A**	*721*	CAAGTGAGAGAAAGCCGGAATCCTGGGAATGCTGAAATTGAAGATCTCATCTTTCTCGCC	*780*
**NP 245626 Method K**	*721*	CAAGTGAGAGAAAGCCGGAATCCTGGGAATGCTGAAATTGAAGATCTCATCTTTCTCGCC	*780*
**NP 245626 Method N**	*721*	CAAGTGAGAGAAAGCCGGAATCCTGGGAATGCTGAAATTGAAGATCTCATCTTTCTCGCC	*780*

**NP 245625 MiSeq**	*781*	CGATCTGCTCTCATCCTGAGGGGATCAGTGGCTCATAAGTCCTGTCTGCCTGCTTGCGTA	*840*
**NP 245626 Method A**	*781*	CGATCTGCTCTCATCCTGAGGGGATCAGTGGCTCATAAGTCCTGTCTGCCTGCTTGCGTA	*840*
**NP 245626 Method K**	*781*	CGATCTGCTCTCATCCTGAGGGGATCAGTGGCTCATAAGTCCTGTCTGCCTGCTTGCGTA	*840*
**NP 245626 Method N**	*781*	CGATCTGCTCTCATCCTGAGGGGATCAGTGGCTCATAAGTCCTGTCTGCCTGCTTGCGTA	*840*

**NP 245625 MiSeq**	*841*	TATGGACTTGCTGTAGCCAGTGGATATGACTTTGAAAGAGAGGGATACTCTCTAGTCGGA	*900*
**NP 245626 Method A**	*841*	TATGGACTTGCTGTAGCCAGTGGATATGACTTTGAAAGAGAGGGATACTCTCTAGTCGGA	*900*
**NP 245626 Method K**	*841*	TATGGACTTGCTGTAGCCAGTGGATATGACTTTGAAAGAGAGGGATACTCTCTAGTCGGA	*900*
**NP 245626 Method N**	*841*	TATGGACTTGCTGTAGCCAGTGGATATGACTTTGAAAGAGAGGGATACTCTCTAGTCGGA	*900*

**NP 245625 MiSeq**	*901*	ATTGATCCTTTCCGTCTGCTCCAAAACAGCCAAGTCTTCAGTCTCATCAGACCGAACGAA	*960*
**NP 245626 Method A**	*901*	ATTGATCCTTTCCGTCTGCTCCAAAACAGCCAAGTCTTCAGTCTCATCAGACCGAACGAA	*960*
**NP 245626 Method K**	*901*	ATTGATCCTTTCCGTCTGCTCCAAAACAGCCAAGTCTTCAGTCTCATCAGACCGAACGAA	*960*
**NP 245626 Method N**	*901*	ATTGATCCTTTCCGTCTGCTCCAAAACAGCCAAGTCTTCAGTCTCATCAGACCGAACGAA	*960*

**NP 245625 MiSeq**	*961*	AATCCAGCTCATAAAAGTCAGCTGGTATGGATGGCATGCCACTCTGCGGCATTTGAGGAT	*1020*
**NP 245626 Method A**	*961*	AATCCAGCTCATAAAAGTCAGCTGGTATGGATGGCATGCCACTCTGCGGCATTTGAGGAT	*1020*
**NP 245626 Method K**	*961*	AATCCAGCTCATAAAAGTCAGCTGGTATGGATGGCATGCCACTCTGCGGCATTTGAGGAT	*1020*
**NP 245626 Method N**	*961*	AATCCAGCTCATAAAAGTCAGCTGGTATGGATGGCATGCCACTCTGCGGCATTTGAGGAT	*1020*

**NP 245625 MiSeq**	*1021*	CTGAGAGTGTCAAGCTTCATCAGAGGGACAAGAGTAGTCCCAAGAGGACAACTGTCCACC	*1080*
**NP 245626 Method A**	*1021*	CTGAGAGTGTCAAGCTTCATCAGAGGGACAAGAGTAGTCCCAAGAGGACAACTGTCCACC	*1080*
**NP 245626 Method K**	*1021*	CTGAGAGTGTCAAGCTTCATCAGAGGGACAAGAGTAGTCCCAAGAGGACAACTGTCCACC	*1080*
**NP 245626 Method N**	*1021*	CTGAGAGTGTCAAGCTTCATCAGAGGGACAAGAGTAGTCCCAAGAGGACAACTGTCCACC	*1080*

**NP 245625 MiSeq**	*1081*	AGAGGAGTTCAGATTGCTTCAAATGAAAACATGGAGACAATGGACTCCAGTACTCTTGAA	*1140*
**NP 245626 Method A**	*1081*	AGAGGAGTTCAGATTGCTTCAAATGAAAACATGGAGACAATGGACTCCAGTACTCTTGAA	*1140*
**NP 245626 Method K**	*1081*	AGAGGAGTTCAGATTGCTTCAAATGAAAACATGGAGACAATGGACTCCAGTACTCTTGAA	*1140*
**NP 245626 Method N**	*1081*	AGAGGAGTTCAGATTGCTTCAAATGAAAACATGGAGACAATGGACTCCAGTACTCTTGAA	*1140*
**NP 245625 MiSeq**	*1141*	CTGAGGAGCAGATACTGGGCTATAAGAACAAGAAGTGGAGGAAACACTAACCAACAGAGA	*1200*
**NP 245626 Method A**	*1141*	CTGAGGAGCAGATACTGGGCTATAAGAACAAGAAGTGGAGGAAACACTAACCAACAGAGA	*1200*
**NP 245626 Method K**	*1141*	CTGAGGAGCAGATACTGGGCTATAAGAACAAGAAGTGGAGGAAACACTAACCAACAGAGA	*1200*
**NP 245626 Method N**	*1141*	CTGAGGAGCAGATACTGGGCTATAAGAACAAGAAGTGGAGGAAACACTAACCAACAGAGA	*1200*
**NP 245625 MiSeq**	*1201*	GCATCTGCAGGACAAATCAGCGTACAGCCCACATTCTCTGTGCAAAGAAACCTCCCATTC	*1260*
**NP 245626 Method A**	*1201*	GCATCTGCAGGACAAATCAGCGTACAGCCCACATTCTCTGTGCAAAGAAACCTCCCATTC	*1260*
**NP 245626 Method K**	*1201*	GCATCTGCAGGACAAATCAGCGTACAGCCCACATTCTCTGTGCAAAGAAACCTCCCATTC	*1260*
**NP 245626 Method N**	*1201*	GCATCTGCAGGACAAATCAGCGTACAGCCCACATTCTCTGTGCAAAGAAACCTCCCATTC	*1260*
**NP 245625 MiSeq**	*1261*	GAGAGAGCAACCATCATGGCAGCATTTACGGGAAACACTGAAGGCAGAACTTCAGACATG	*1320*
**NP 245626 Method A**	*1261*	GAGAGAGCAACCATCATGGCAGCATTTACGGGAAACACTGAAGGCAGAACTTCAGACATG	*1320*
**NP 245626 Method K**	*1261*	GAGAGAGCAACCATCATGGCAGCATTTACGGGAAACACTGAAGGCAGAACTTCAGACATG	*1320*
**NP 245626 Method N**	*1261*	GAGAGAGCAACCATCATGGCAGCATTTACGGGAAACACTGAAGGCAGAACTTCAGACATG	*1320*
**NP 245625 MiSeq**	*1321*	AGAACTGAGATCATAAGGATGATGGAAAATGCCAGACCTGAAGATGTGTCTTTCCAGGGG	*1380*
**NP 245626 Method A**	*1321*	AGAACTGAGATCATAAGGATGATGGAAAATGCCAGACCTGAAGATGTGTCTTTCCAGGGG	*1380*
**NP 245626 Method K**	*1321*	AGAACTGAGATCATAAGGATGATGGAAAATGCCAGACCTGAAGATGTGTCTTTCCAGGGG	*1380*
**NP 245626 Method N**	*1321*	AGAACTGAGATCATAAGGATGATGGAAAATGCCAGACCTGAAGATGTGTCTTTCCAGGGG	*1380*
**NP 245625 MiSeq**	*1381*	CGGGGAGTCTTCGAGCTCTCGGACGAAAAGGCAACGAACCCGATCGTGCCTTCCTTTGAC	*1440*
**NP 245626 Method A**	*1381*	CGGGGAGTCTTCGAGCTCTCGGACGAAAAGGCAACGAACCCGATCGTGCCTTCCTTTGAC	*1440*
**NP 245626 Method K**	*1381*	CGGGGAGTCTTCGAGCTCTCGGACGAAAAGGCAACGAACCCGATCGTGCCTTCCTTTGAC	*1440*
**NP 245626 Method N**	*1381*	CGGGGAGTCTTCGAGCTCTCGGACGAAAAGGCAACGAACCCGATCGTGCCTTCCTTTGAC	*1440*

**NP 245625 MiSeq**	*1441*	ATGAGTAATGAAGGATCTTATTTCTTCGGAGACAATGCAGAGGAGTATGACAATTAA	*1497*
**NP 245626 Method A**	*1441*	ATGAGTAATGAAGGATCTTATTTCTTCGGAGACAATGCAGAGGAGTATGACAATTAA	*1497*
**NP 245626 Method K**	*1441*	ATGAGTAATGAAGGATCTTATTTCTTCGGAGACAATGCAGAGGAGTATGACAATTAA	*1497*
**NP 245626 Method N**	*1441*	ATGAGTAATGAAGGATCTTATTTCTTCGGAGACAATGCAGAGGAGTATGACAATTAA	*1497*
