# Supplementary material for: An Evaluation of Avian Influenza Virus Whole-Genome Sequencing Approaches Using Nanopore Technology
Source: Microorganisms. 2023 Feb 19;11(2):529. doi: 10.3390/microorganisms11020529 (PMC9967579; doi:10.3390/microorganisms11020529)
Supplement: Supplementary file 1 [file microorganisms-11-00529-s001.zip › manuscript.v8 230219 Suppl Figures and Tables/Supplementary Figures S2a-h 245626/Supplementary Figure S2g MA.pdf]

Formatted Alignments

**MA 245625 MiSeq**	*1*	ATGAGTCTTCTAACCGAGGTCGAAACGTACGTTCTCTCTATCGTCCCGTCAGGCCCCCTC	*60*
**MA 245626 Method A**	*1*	ATGAGTCTTCTAACCGAGGTCGAAACGTACGTTCTCTCTATCGTCCCGTCAGGCCCCCTC	*60*
**MA 245626 Method S**	*1*	ATGAGTCTTCTAACCGAGGTCGAAACGTACGTTCTCTCTATCGTCCCGTCAGGCCCCCTC	*60*
**MA 245626 Method K**	*1*	ATGAGTCTTCTAACCGAGGTCGAAACGTACGTTCTCTCTATCGTCCCGTCAGGCCCCCTC	*60*
**MA 245626 Method N**	*1*	ATGAGTCTTCTAACCGAGGTCGAAACGTACGTTCTCTCTATCGTCCCGTCAGGCCCCCTC	*60*
**MA 245625 MiSeq**	*61*	AAAGCCGAGATCGCGCAGAGACTTGAAGATGTCTTTGCAGGGAAGAACACCGATCTTGAG	*120*
**MA 245626 Method A**	*61*	AAAGCCGAGATCGCGCAGAGACTTGAAGATGTCTTTGCAGGGAAGAACACCGATCTTGAG	*120*
**MA 245626 Method S**	*61*	AAAGCCGAGATCGCGCAGAGACTTGAAGATGTCTTTGCAGGGAAGAACACCGATCTTGAG	*120*
**MA 245626 Method K**	*61*	AAAGCCGAGATCGCGCAGAGACTTGAAGATGTCTTTGCAGGGAAGAACACCGATCTTGAG	*120*
**MA 245626 Method N**	*61*	AAAGCCGAGATCGCGCAGAGACTTGAAGATGTCTTTGCAGGGAAGAACACCGATCTTGAG	*120*
**MA 245625 MiSeq**	*121*	GCTCTCATGGAATGGCTAAAGACAAGACCAATCCTGTCACCTCTGACTAAGGGGATTTTG	*180*
**MA 245626 Method A**	*121*	GCTCTCATGGAATGGCTAAAGACAAGACCAATCCTGTCACCTCTGACTAAGGGGATTTTG	*180*
**MA 245626 Method S**	*121*	GCTCTCATGGAATGGCTAAAGACAAGACCAATCCTGTCACCTCTGACTAAGGGGATTTTG	*180*
**MA 245626 Method K**	*121*	GCTCTCATGGAATGGCTAAAGACAAGACCAATCCTGTCACCTCTGACTAAGGGGATTTTG	*180*
**MA 245626 Method N**	*121*	GCTCTCATGGAATGGCTAAAGACAAGACCAATCCTGTCACCTCTGACTAAGGGGATTTTG	*180*
**MA 245625 MiSeq**	*181*	GGATTTGTGTTCACGCTCACCGTGCCCAGTGAGCGAGGACTGCAGCGTAGACGCTTTGTC	*240*
**MA 245626 Method A**	*181*	GGATTTGTGTTCACGCTCACCGTGCCCAGTGAGCGAGGACTGCAGCGTAGACGCTTTGTC	*240*
**MA 245626 Method S**	*181*	GGATTTGTGTTCACGCTCACCGTGCCCAGTGAGCGAGGACTGCAGCGTAGACGCTTTGTC	*240*
**MA 245626 Method K**	*181*	GGATTTGTGTTCACGCTCACCGTGCCCAGTGAGCGAGGACTGCAGCGTAGACGCTTTGTC	*240*
**MA 245626 Method N**	*181*	GGATTTGTGTTCACGCTCACCGTGCCCAGTGAGCGAGGACTGCAGCGTAGACGCTTTGTC	*240*
**MA 245625 MiSeq**	*241*	CAAAATGCTCTAAATGGAAATGGAGACCCAAACAACATGGACAGGGCAGTCAAGTTGTAC	*300*
**MA 245626 Method A**	*241*	CAAAATGCTCTAAATGGAAATGGAGACCCAAACAACATGGACAGGGCAGTCAAGTTGTAC	*300*
**MA 245626 Method S**	*241*	CAAAATGCTCTAAATGGAAATGGAGACCCAAACAACATGGACAGGGCAGTCAAGTTGTAC	*300*
**MA 245626 Method K**	*241*	CAAAATGCTCTAAATGGAAATGGAGACCCAAACAACATGGACAGGGCAGTCAAGTTGTAC	*300*
**MA 245626 Method N**	*241*	CAAAATGCTCTAAATGGAAATGGAGACCCAAACAACATGGACAGGGCAGTCAAGTTGTAC	*300*

**MA 245625 MiSeq**	*301*	AGGAAACTGAAGAGAGAGATAACATTCCATGGGGCTAAAGAAGTTGCACTCAGTTACTCA	*360*
**MA 245626 Method A**	*301*	AGGAAACTGAAGAGAGAGATAACATTCCATGGGGCTAAAGAAGTTGCACTCAGTTACTCA	*360*
**MA 245626 Method S**	*301*	AGGAAACTGAAGAGAGAGATAACATTCCATGGGGCTAAAGAAGTTGCACTCAGTTACTCA	*360*
**MA 245626 Method K**	*301*	AGGAAACTGAAGAGAGAGATAACATTCCATGGGGCTAAAGAAGTTGCACTCAGTTACTCA	*360*
**MA 245626 Method N**	*301*	AGGAAACTGAAGAGAGAGATAACATTCCATGGGGCTAAAGAAGTTGCACTCAGTTACTCA	*360*
**MA 245625 MiSeq**	*361*	ACCGGTGCACTTGCCAGTTGTATGGGTCTCATATACAACAGGATGGGGACGGTGACCGCA	*420*
**MA 245626 Method A**	*361*	ACCGGTGCACTTGCCAGTTGTATGGGTCTCATATACAACAGGATGGGGACGGTGACCGCA	*420*
**MA 245626 Method S**	*361*	ACCGGTGCACTTGCCAGTTGTATGGGTCTCATATACAACAGGATGGGGACGGTGACCGCA	*420*
**MA 245626 Method K**	*361*	ACCGGTGCACTTGCCAGTTGTATGGGTCTCATATACAACAGGATGGGGACGGTGACCGCA	*420*
**MA 245626 Method N**	*361*	ACCGGTGCACTTGCCAGTTGTATGGGTCTCATATACAACAGGATGGGGACGGTGACCGCA	*420*
**MA 245625 MiSeq**	*421*	GAAGTGGCATTGGGCCTAGTGTGTGCCACCTGTGAGCAGATTGCTGATTCACAGCATCGG	*480*
**MA 245626 Method A**	*421*	GAAGTGGCATTGGGCCTAGTGTGTGCCACCTGTGAGCAGATTGCTGATTCACAGCATCGG	*480*
**MA 245626 Method S**	*421*	GAAGTGGCATTGGGCCTAGTGTGTGCCACCTGTGAGCAGATTGCTGATTCACAGCATCGG	*480*
**MA 245626 Method K**	*421*	GAAGTGGCATTGGGCCTAGTGTGTGCCACCTGTGAGCAGATTGCTGATTCACAGCATCGG	*480*
**MA 245626 Method N**	*421*	GAAGTGGCATTGGGCCTAGTGTGTGCCACCTGTGAGCAGATTGCTGATTCACAGCATCGG	*480*
**MA 245625 MiSeq**	*481*	TCTCACAGACAGATAGCTACCACCACCAACCCACTAATCAGACATGAAAACAGAATGGTG	*540*
**MA 245626 Method A**	*481*	TCTCACAGACAGATAGCTACCACCACCAACCCACTAATCAGACATGAAAACAGAATGGTG	*540*
**MA 245626 Method S**	*481*	TCTCACAGACAGATAGCTACCACCACCAACCCACTAATCAGACATGAAAACAGAATGGTG	*540*
**MA 245626 Method K**	*481*	TCTCACAGACAGATAGCTACCACCACCAACCCACTAATCAGACATGAAAACAGAATGGTG	*540*
**MA 245626 Method N**	*481*	TCTCACAGACAGATAGCTACCACCACCAACCCACTAATCAGACATGAAAACAGAATGGTG	*540*
**MA 245625 MiSeq**	*541*	TTGGCCAGTACTACAGCTAAGGCTATGGAGCAGATGGCTGGATCGAGTGAGCAAGCAGTG	*600*
**MA 245626 Method A**	*541*	TTGGCCAGTACTACAGCTAAGGCTATGGAGCAGATGGCTGGATCGAGTGAGCAAGCAGTG	*600*
**MA 245626 Method S**	*541*	TTGGCCAGTACTACAGCTAAGGCTATGGAGCAGATGGCTGGATCGAGTGAGCAAGCAGTG	*600*
**MA 245626 Method K**	*541*	TTGGCCAGTACTACAGCTAAGGCTATGGAGCAGATGGCTGGATCGAGTGAGCAAGCAGTG	*600*
**MA 245626 Method N**	*541*	TTGGCCAGTACTACAGCTAAGGCTATGGAGCAGATGGCTGGATCGAGTGAGCAAGCAGTG	*600*

**MA 245625 MiSeq**	*601*	GAAGCCATGGAGGTTGCTAGTCAGGCTAGGCAGATGGTGCAGGCGATGAGGACCATTGGA	*660*
**MA 245626 Method A**	*601*	GAAGCCATGGAGGTTGCTAGTCAGGCTAGGCAGATGGTGCAGGCGATGAGGACCATTGGA	*660*
**MA 245626 Method S**	*601*	GAAGCCATGGAGGTTGCTAGTCAGGCTAGGCAGATGGTGCAGGCGATGAGGACCATTGGA	*660*
**MA 245626 Method K**	*601*	GAAGCCATGGAGGTTGCTAGTCAGGCTAGGCAGATGGTGCAGGCGATGAGGACCATTGGA	*660*
**MA 245626 Method N**	*601*	GAAGCCATGGAGGTTGCTAGTCAGGCTAGGCAGATGGTGCAGGCGATGAGGACCATTGGA	*660*

**MA 245625 MiSeq**	*661*	ACTCATCCTAGCTCCAGTGCCGGTCTGAGAGATGATCTCCTTGAAAATTTGCAGGCCTAC	*720*
**MA 245626 Method A**	*661*	ACTCATCCTAGCTCCAGTGCCGGTCTGAGAGATGATCTCCTTGAAAATTTGCAGGCCTAC	*720*
**MA 245626 Method S**	*661*	ACTCATCCTAGCTCCAGTGCCGGTCTGAGAGATGATCTCCTTGAAAATTTGCAGGCCTAC	*720*
**MA 245626 Method K**	*661*	ACTCATCCTAGCTCCAGTGCCGGTCTGAGAGATGATCTCCTTGAAAATTTGCAGGCCTAC	*720*
**MA 245626 Method N**	*661*	ACTCATCCTAGCTCCAGTGCCGGTCTGAGAGATGATCTCCTTGAAAATTTGCAGGCCTAC	*720*

**MA 245625 MiSeq**	*721*	CAAAAACGGATGGGAGTGCAACTGCAGCGATTCAAGTGATCCTCTCGTTATTGCCGCAAG	*780*
**MA 245626 Method A**	*721*	CAAAAACGGATGGGAGTGCAACTGCAGCGATTCAAGTGATCCTCTCGTTATTGCCGCAAG	*780*
**MA 245626 Method S**	*721*	CAAAAACGGATGGGAGTGCAACTGCAGCGATTCAAGTGATCCTCTCGTTATTGCCGCAAG	*780*
**MA 245626 Method K**	*721*	CAAAAACGGATGGGAGTGCAACTGCAGCGATTCAAGTGATCCTCTCGTTATTGCCGCAAG	*780*
**MA 245626 Method N**	*721*	CAAAAACGGATGGGAGTGCAACTGCAGCGATTCAAGTGATCCTCTCGTTATTGCCGCAAG	*780*

**MA 245625 MiSeq**	*781*	TATCGTTGGGATCTTGCACTTGATATTGTGGATTCTTGATCGCCTTTTCTTCAAATGCGT	*840*
**MA 245626 Method A**	*781*	TATCGTTGGGATCTTGCACTTGATATTGTGGATTCTTGATCGCCTTTTCTTCAAATGCGT	*840*
**MA 245626 Method S**	*781*	TATCGTTGGGATCTTGCACTTGATATTGTGGATTCTTGATCGCCTTTTCTTCAAATGCGT	*840*
**MA 245626 Method K**	*781*	TATCGTTGGGATCTTGCACTTGATATTGTGGATTCTTGATCGCCTTTTCTTCAAATGCGT	*840*
**MA 245626 Method N**	*781*	TATCGTTGGGATCTTGCACTTGATATTGTGGATTCTTGATCGCCTTTTCTTCAAATGCGT	*840*

**MA 245625 MiSeq**	*841*	TTATCGTCGCCTTAAATACGGTTTGAAAGGAGGGCCTTCTACGGAAGGAGTACCTGAGTC	*900*
**MA 245626 Method A**	*841*	TTATCGTCGCCTTAAATACGGTTTGAAAGGAGGGCCTTCTACGGAAGGAGTACCTGAGTC	*900*
**MA 245626 Method S**	*841*	TTATCGTCGCCTTAAATACGGTTTGAAAGGAGGGCCTTCTACGGAAGGAGTACCTGAGTC	*900*
**MA 245626 Method K**	*841*	TTATCGTCGCCTTAAATACGGTTTGAAAGGAGGGCCTTCTACGGAAGGAGTACCTGAGTC	*900*
**MA 245626 Method N**	*841*	TTATCGTCGCCTTAAATACGGTTTGAAAGGAGGGCCTTCTACGGAAGGAGTACCTGAGTC	*900*

**MA 245625 MiSeq**	*901*	CATGAGGGAAGAGTACCGGCAGGAACAGCAGAGTGCTGTGGATGTTGACGATGGTCATTT	*960*
**MA 245626 Method A**	*901*	CATGAGGGAAGAGTACCGGCAGGAACAGCAGAGTGCTGTGGATGTTGACGATGGTCATTT	*960*
**MA 245626 Method S**	*901*	CATGAGGGAAGAGTACCGGCAGGAACAGCAGAGTGCTGTGGATGTTGACGATGGTCATTT	*960*
**MA 245626 Method K**	*901*	CATGAGGGAAGAGTACCGGCAGGAACAGCAGAGTGCTGTGGATGTTGACGATGGTCATTT	*960*
**MA 245626 Method N**	*901*	CATGAGGGAAGAGTACCGGCAGGAACAGCAGAGTGCTGTGGATGTTGACGATGGTCATTT	*960*

**MA 245625 MiSeq**	*961*	TGTCAACATAGAGCTGGAGTAA	*982*
**MA 245626 Method A**	*961*	TGTCAACATAGAGCTGGAGTAA	*982*
**MA 245626 Method S**	*961*	TGTCAACATAGAGCTGGAGTAA	*982*
**MA 245626 Method K**	*961*	TGTCAACATAGAGCTGGAGTAA	*982*
**MA 245626 Method N**	*961*	TGTCAACATAGAGCTGGAGTAA	*982*
