# Supplementary material for: An Evaluation of Avian Influenza Virus Whole-Genome Sequencing Approaches Using Nanopore Technology
Source: Microorganisms. 2023 Feb 19;11(2):529. doi: 10.3390/microorganisms11020529 (PMC9967579; doi:10.3390/microorganisms11020529)
Supplement: Supplementary file 1 [file microorganisms-11-00529-s001.zip › manuscript.v8 230219 Suppl Figures and Tables/Supplementary Figures S2a-h 245626/Supplementary Figure S2b PB1.pdf]

Formatted Alignments

**PB1 245625 MiSeq**	*1*	ATGGATGTCAATCCGACTTTACTTTTCTTAAAAGTGCCAGCGCAAAATGCCATAAGTACC	*60*
**PB1 245626 Method A**	*1*	ATGGATGTCAATCCGACTTTACTTTTCTTAAAAGTGCCAGCGCAAAATGCCATAAGTACC	*60*
**PB1 245626 Method E**	*1*	ATGGATGTCAATCCGACTTTACTTTTCTTAAAAGTGCCAGCGCAAAATGCCATAAGTACC	*60*
**PB1 245626 Method K**	*1*	ATGGATGTCAATCCGACTTTACTTTTCTTAAAAGTGCCAGCGCAAAATGCCATAAGTACC	*60*
**PB1 245626 Method N**	*1*	ATGGATGTCAATCCGACTTTACTTTTCTTAAAAGTGCCAGCGCAAAATGCCATAAGTACC	*60*
**PB1 245625 MiSeq**	*61*	ACATTCCCTTATACTGGAGATCCTCCATACAGCCATGGAACAGGGACAGGATACACAATG	*120*
**PB1 245626 Method A**	*61*	ACATTCCCTTATACTGGAGATCCTCCATACAGCCATGGAACAGGGACAGGATACACAATG	*120*
**PB1 245626 Method E**	*61*	ACATTCCCTTATACTGGAGATCCTCCATACAGCCATGGAACAGGGACAGGATACACAATG	*120*
**PB1 245626 Method K**	*61*	ACATTCCCTTATACTGGAGATCCTCCATACAGCCATGGAACAGGGACAGGATACACAATG	*120*
**PB1 245626 Method N**	*61*	ACATTCCCTTATACTGGAGATCCTCCATACAGCCATGGAACAGGGACAGGATACACAATG	*120*
**PB1 245625 MiSeq**	*121*	GACACAGTCAACAGAACACATCAATACTCAGAGAAGGGAAAATGGACAACAAACACAGAA	*180*
**PB1 245626 Method A**	*121*	GACACAGTCAACAGAACACATCAATACTCAGAGAAGGGAAAATGGACAACAAACACAGAA	*180*
**PB1 245626 Method E**	*121*	GACACAGTCAACAGAACACATCAATACTCAGAGAAGGGAAAATGGACAACAAACACAGAA	*180*
**PB1 245626 Method K**	*121*	GACACAGTCAACAGAACACATCAATACTCAGAGAAGGGAAAATGGACAACAAACACAGAA	*180*
**PB1 245626 Method N**	*121*	GACACAGTCAACAGAACACATCAATACTCAGAGAAGGGAAAATGGACAACAAACACAGAA	*180*
**PB1 245625 MiSeq**	*181*	ACCGGAGCACCTCAACTCAACCCAATTGATGGACCACTACCTGAGGACAACGAACCGAGC	*240*
**PB1 245626 Method A**	*181*	ACCGGAGCACCTCAACTCAACCCAATTGATGGACCACTACCTGAGGACAACGAACCGAGC	*240*
**PB1 245626 Method E**	*181*	ACCGGAGCACCTCAACTCAACCCAATTGATGGACCACTACCTGAGGACAACGAACCGAGC	*240*
**PB1 245626 Method K**	*181*	ACCGGAGCACCTCAACTCAACCCAATTGATGGACCACTACCTGAGGACAACGAACCGAGC	*240*
**PB1 245626 Method N**	*181*	ACCGGAGCACCTCAACTCAACCCAATTGATGGACCACTACCTGAGGACAACGAACCGAGC	*240*
**PB1 245625 MiSeq**	*241*	GGATATGCACAAACAGATTGCGTGTTGGAAGCAATGGCTTTCCTTGAAGAGTCCCACCCA	*300*
**PB1 245626 Method A**	*241*	GGATATGCACAAACAGATTGCGTGTTGGAAGCAATGGCTTTCCTTGAAGAGTCCCACCCA	*300*
**PB1 245626 Method E**	*241*	GGATATGCACAAACAGATTGCGTGTTGGAAGCAATGGCTTTCCTTGAAGAGTCCCACCCA	*300*
**PB1 245626 Method K**	*241*	GGATATGCACAAACAGATTGCGTGTTGGAAGCAATGGCTTTCCTTGAAGAGTCCCACCCA	*300*
**PB1 245626 Method N**	*241*	GGATATGCACAAACAGATTGCGTGTTGGAAGCAATGGCTTTCCTTGAAGAGTCCCACCCA	*300*

**PB1 245625 MiSeq**	*301*	GGGATCTTTGGAAACTCTTGTCTTGAAACGATGGAAGTCGTTCAGCAAACAAGAGTGGAC	*360*
**PB1 245626 Method A**	*301*	GGGATCTTTGGAAACTCTTGTCTTGAAACGATGGAAGTCGTTCAGCAAACAAGAGTGGAC	*360*
**PB1 245626 Method E**	*301*	GGGATCTTTGGAAACTCTTGTCTTGAAACGATGGAAGTCGTTCAGCAAACAAGAGTGGAC	*360*
**PB1 245626 Method K**	*301*	GGGATCTTTGGAAACTCTTGTCTTGAAACGATGGAAGTCGTTCAGCAAACAAGAGTGGAC	*360*
**PB1 245626 Method N**	*301*	GGGATCTTTGGAAACTCTTGTCTTGAAACGATGGAAGTCGTTCAGCAAACAAGAGTGGAC	*360*
**PB1 245625 MiSeq**	*361*	AAACTAACTCAAGGTCGCCAGACTTATGACTGGACACTGAATAGAAACCAACCAGCTGCA	*420*
**PB1 245626 Method A**	*361*	AAACTAACTCAAGGTCGCCAGACTTATGACTGGACACTGAATAGAAACCAACCAGCTGCA	*420*
**PB1 245626 Method E**	*361*	AAACTAACTCAAGGTCGCCAGACTTATGACTGGACACTGAATAGAAACCAACCAGCTGCA	*420*
**PB1 245626 Method K**	*361*	AAACTAACTCAAGGTCGCCAGACTTATGACTGGACACTGAATAGAAACCAACCAGCTGCA	*420*
**PB1 245626 Method N**	*361*	AAACTAACTCAAGGTCGCCAGACTTATGACTGGACACTGAATAGAAACCAACCAGCTGCA	*420*
**PB1 245625 MiSeq**	*421*	ACTGCCCTGGCCAACACTATAGAGGTCTTCAGATCAAACGGTCTAACAGCCAATGAATCG	*480*
**PB1 245626 Method A**	*421*	ACTGCCCTGGCCAACACTATAGAGGTCTTCAGATCAAACGGTCTAACAGCCAATGAATCG	*480*
**PB1 245626 Method E**	*421*	ACTGCCCTGGCCAACACTATAGAGGTCTTCAGATCAAACGGTCTAACAGCCAATGAATCG	*480*
**PB1 245626 Method K**	*421*	ACTGCCCTGGCCAACACTATAGAGGTCTTCAGATCAAACGGTCTAACAGCCAATGAATCG	*480*
**PB1 245626 Method N**	*421*	ACTGCCCTGGCCAACACTATAGAGGTCTTCAGATCAAACGGTCTAACAGCCAATGAATCG	*480*
**PB1 245625 MiSeq**	*481*	GGGAGACTAATAGATTTCCTCAAGGACGTGATGGACTCAATGGATACAGAAGAAATGGAA	*540*
**PB1 245626 Method A**	*481*	GGGAGACTAATAGATTTCCTCAAGGACGTGATGGACTCAATGGATACAGAAGAAATGGAA	*540*
**PB1 245626 Method E**	*481*	GGGAGACTAATAGATTTCCTCAAGGACGTGATGGACTCAATGGATACAGAAGAAATGGAA	*540*
**PB1 245626 Method K**	*481*	GGGAGACTAATAGATTTCCTCAAGGACGTGATGGACTCAATGGATACAGAAGAAATGGAA	*540*
**PB1 245626 Method N**	*481*	GGGAGACTAATAGATTTCCTCAAGGACGTGATGGACTCAATGGATACAGAAGAAATGGAA	*540*
**PB1 245625 MiSeq**	*541*	ATAACAACACATTTCCAGAGAAAGAGAAGAGTAAGGGACAACATGACCAAGAAAATGGTC	*600*
**PB1 245626 Method A**	*541*	ATAACAACACATTTCCAGAGAAAGAGAAGAGTAAGGGACAACATGACCAAGAAAATGGTC	*600*
**PB1 245626 Method E**	*541*	ATAACAACACATTTCCAGAGAAAGAGAAGAGTAAGGGACAACATGACCAAGAAAATGGTC	*600*
**PB1 245626 Method K**	*541*	ATAACAACACATTTCCAGAGAAAGAGAAGAGTAAGGGACAACATGACCAAGAAAATGGTC	*600*
**PB1 245626 Method N**	*541*	ATAACAACACATTTCCAGAGAAAGAGAAGAGTAAGGGACAACATGACCAAGAAAATGGTC	*600*

**PB1 245625 MiSeq**	*601*	ACACAAAGAACAATAGGAAAGAAGAAACAAAGGCTAAACAAGAGGAGCTACTTAATAAGA	*660*
**PB1 245626 Method A**	*601*	ACACAAAGAACAATAGGAAAGAAGAAACAAAGGCTAAACAAGAGGAGCTACTTAATAAGA	*660*
**PB1 245626 Method E**	*601*	ACACAAAGAACAATAGGAAAGAAGAAACAAAGGCTAAACAAGAGGAGCTACTTAATAAGA	*660*
**PB1 245626 Method K**	*601*	ACACAAAGAACAATAGGAAAGAAGAAACAAAGGCTAAACAAGAGGAGCTACTTAATAAGA	*660*
**PB1 245626 Method N**	*601*	ACACAAAGAACAATAGGAAAGAAGAAACAAAGGCTAAACAAGAGGAGCTACTTAATAAGA	*660*
**PB1 245625 MiSeq**	*661*	GCACTGACACTGAATACAATGACAAAAGATGCAGAAAGAGGCAAATTGAAGAGACGGGCG	*720*
**PB1 245626 Method A**	*661*	GCACTGACACTGAATACAATGACAAAAGATGCAGAAAGAGGCAAATTGAAGAGACGGGCG	*720*
**PB1 245626 Method E**	*661*	GCACTGACACTGAATACAATGACAAAAGATGCAGAAAGAGGCAAATTGAAGAGACGGGCG	*720*
**PB1 245626 Method K**	*661*	GCACTGACACTGAATACAATGACAAAAGATGCAGAAAGAGGCAAATTGAAGAGACGGGCG	*720*
**PB1 245626 Method N**	*661*	GCACTGACACTGAATACAATGACAAAAGATGCAGAAAGAGGCAAATTGAAGAGACGGGCG	*720*
**PB1 245625 MiSeq**	*721*	ATTGCAACACCAGGGATGCAGATTAGAGGATTTGTGTACTTTGTCGAAACACTGGCAAGG	*780*
**PB1 245626 Method A**	*721*	ATTGCAACACCAGGGATGCAGATTAGAGGATTTGTGTACTTTGTCGAAACACTGGCAAGG	*780*
**PB1 245626 Method E**	*721*	ATTGCAACACCAGGGATGCAGATTAGAGGATTTGTGTACTTTGTCGAAACACTGGCAAGG	*780*
**PB1 245626 Method K**	*721*	ATTGCAACACCAGGGATGCAGATTAGAGGATTTGTGTACTTTGTCGAAACACTGGCAAGG	*780*
**PB1 245626 Method N**	*721*	ATTGCAACACCAGGGATGCAGATTAGAGGATTTGTGTACTTTGTCGAAACACTGGCAAGG	*780*
**PB1 245625 MiSeq**	*781*	AGCATCTGTGAAAAACTTGAGCAATCTGGACTCCCCGTTGGAGGAAATGAGAAGAAGGCT	*840*
**PB1 245626 Method A**	*781*	AGCATCTGTGAAAAACTTGAGCAATCTGGACTCCCCGTTGGAGGAAATGAGAAGAAGGCT	*840*
**PB1 245626 Method E**	*781*	AGCATCTGTGAAAAACTTGAGCAATCTGGACTCCCCGTTGGAGGAAATGAGAAGAAGGCT	*840*
**PB1 245626 Method K**	*781*	AGCATCTGTGAAAAACTTGAGCAATCTGGACTCCCCGTTGGAGGAAATGAGAAGAAGGCT	*840*
**PB1 245626 Method N**	*781*	AGCATCTGTGAAAAACTTGAGCAATCTGGACTCCCCGTTGGAGGAAATGAGAAGAAGGCT	*840*
**PB1 245625 MiSeq**	*841*	AAATTGGCAAATGTCGTGAGGAAAATGATGACTAACTCACAAGATACAGAGCTCTCCTTC	*900*
**PB1 245626 Method A**	*841*	AAATTGGCAAATGTCGTGAGGAAAATGATGACTAACTCACAAGATACAGAGCTCTCCTTC	*900*
**PB1 245626 Method E**	*841*	AAATTGGCAAATGTCGTGAGGAAAATGATGACTAACTCACAAGATACAGAGCTCTCCTTC	*900*
**PB1 245626 Method K**	*841*	AAATTGGCAAATGTCGTGAGGAAAATGATGACTAACTCACAAGATACAGAGCTCTCCTTC	*900*
**PB1 245626 Method N**	*841*	AAATTGGCAAATGTCGTGAGGAAAATGATGACTAACTCACAAGATACAGAGCTCTCCTTC	*900*

**PB1 245625 MiSeq**	*901*	ACAATTACTGGAGATAACACAAAATGGAATGAGAATCAAAATCCTCGGATGTTTCTGGCA	*960*
**PB1 245626 Method A**	*901*	ACAATTACTGGAGATAACACAAAATGGAATGAGAATCAAAATCCTCGGATGTTTCTGGCA	*960*
**PB1 245626 Method E**	*901*	ACAATTACTGGAGATAACACAAAATGGAATGAGAATCAAAATCCTCGGATGTTTCTGGCA	*960*
**PB1 245626 Method K**	*901*	ACAATTACTGGAGATAACACAAAATGGAATGAGAATCAAAATCCTCGGATGTTTCTGGCA	*960*
**PB1 245626 Method N**	*901*	ACAATTACTGGAGATAACACAAAATGGAATGAGAATCAAAATCCTCGGATGTTTCTGGCA	*960*

**PB1 245625 MiSeq**	*961*	ATGATAACGTACATTACAAGAAACCAACCTGAATGGTTTAGAAATGTCTTGAGTATTGCC	*1020*
**PB1 245626 Method A**	*961*	ATGATAACGTACATTACAAGAAACCAACCTGAATGGTTTAGAAATGTCTTGAGTATTGCC	*1020*
**PB1 245626 Method E**	*961*	ATGATAACGTACATTACAAGAAACCAACCTGAATGGTTTAGAAATGTCTTGAGTATTGCC	*1020*
**PB1 245626 Method K**	*961*	ATGATAACGTACATTACAAGAAACCAACCTGAATGGTTTAGAAATGTCTTGAGTATTGCC	*1020*
**PB1 245626 Method N**	*961*	ATGATAACGTACATTACAAGAAACCAACCTGAATGGTTTAGAAATGTCTTGAGTATTGCC	*1020*

**PB1 245625 MiSeq**	*1021*	CCTATAATGTTCTCGAACAAAATGGCGAGATTGGGAAAAGGGTACATGTTTGAAAGTAAG	*1080*
**PB1 245626 Method A**	*1021*	CCTATAATGTTCTCGAACAAAATGGCGAGATTGGGAAAAGGGTACATGTTTGAAAGTAAG	*1080*
**PB1 245626 Method E**	*1021*	CCTATAATGTTCTCGAACAAAATGGCGAGATTGGGAAAAGGGTACATGTTTGAAAGTAAG	*1080*
**PB1 245626 Method K**	*1021*	CCTATAATGTTCTCGAACAAAATGGCGAGATTGGGAAAAGGGTACATGTTTGAAAGTAAG	*1080*
**PB1 245626 Method N**	*1021*	CCTATAATGTTCTCGAACAAAATGGCGAGATTGGGAAAAGGGTACATGTTTGAAAGTAAG	*1080*

**PB1 245625 MiSeq**	*1081*	AGCATGAAGTTACGGACACAAATACCTGCAGAAATACTTGCAAACATTGACTTAAAATAC	*1140*
**PB1 245626 Method A**	*1081*	AGCATGAAGTTACGGACACAAATACCTGCAGAAATACTTGCAAACATTGACTTAAAATAC	*1140*
**PB1 245626 Method E**	*1081*	AGCATGAAGTTACGGACACAAATACCTGCAGAAATACTTGCAAACATTGACTTAAAATAC	*1140*
**PB1 245626 Method K**	*1081*	AGCATGAAGTTACGGACACAAATACCTGCAGAAATACTTGCAAACATTGACTTAAAATAC	*1140*
**PB1 245626 Method N**	*1081*	AGCATGAAGTTACGGACACAAATACCTGCAGAAATACTTGCAAACATTGACTTAAAATAC	*1140*

**PB1 245625 MiSeq**	*1141*	TTCAATGAATCAACAAGAAAGAAAATCGAAAAAATAAGGCCTCTACTAATAGATGGCACT	*1200*
**PB1 245626 Method A**	*1141*	TTCAATGAATCAACAAGAAAGAAAATCGAAAAAATAAGGCCTCTACTAATAGATGGCACT	*1200*
**PB1 245626 Method E**	*1141*	TTCAATGAATCAACAAGAAAGAAAATCGAAAAAATAAGGCCTCTACTAATAGATGGCACT	*1200*
**PB1 245626 Method K**	*1141*	TTCAATGAATCAACAAGAAAGAAAATCGAAAAAATAAGGCCTCTACTAATAGATGGCACT	*1200*
**PB1 245626 Method N**	*1141*	TTCAATGAATCAACAAGAAAGAAAATCGAAAAAATAAGGCCTCTACTAATAGATGGCACT	*1200*

```
PB1 245625 MiSeq     1201 GCCTCATTGAGTCCTGGAATGATGATGGGCATGTTCAATATGCTGAGTACAGTATTAGGA 1260
PB1 245626 Method A  1201 GCCTCATTGAGTCCTGGAATGATGATGGGCATGTTCAATATGCTGAGTACAGTATTAGGA 1260
PB1 245626 Method E  1201 GCCTCATTGAGTCCTGGAATGATGATGGGCATGTTCAATATGCTGAGTACAGTATTAGGA 1260
PB1 245626 Method K  1201 GCCTCATTGAGTCCTGGAATGATGATGGGCATGTTCAATATGCTGAGTACAGTATTAGGA 1260
PB1 245626 Method N  1201 GCCTCATTGAGTCCTGGTATGATGATGGGCATGTTCAATATGCTGAGTACAGTATTAGGA 1260

PB1 245625 MiSeq     1261 GTTTCAATCCTAAATCTTGGGCAAAAGAGGTACACCAAAACCACATACTGGTGGGATGGA 1320
PB1 245626 Method A  1261 GTTTCAATCCTAAATCTTGGGCAAAAGAGGTACACCAAAACCACATACTGGTGGGATGGA 1320
PB1 245626 Method E  1261 GTTTCAATCCTAAATCTTGGGCAAAAGAGGTACACCAAAACCACATACTGGTGGGATGGA 1320
PB1 245626 Method K  1261 GTTTCAATCCTAAATCTTGGGCAAAAGAGGTACACCAAAACCACATACTGGTGGGATGGA 1320
PB1 245626 Method N  1261 GTTTCAATCCTAAATCTTGGGCAAAAGAGGTACACCAAAACCACATACTGGTGGGATGGA 1320

PB1 245625 MiSeq     1321 CTCCAATCCTCTGATGATTTCGCCCTCATAGTAAATGCACCTAATCATGAGGGAATACAA 1380
PB1 245626 Method A  1321 CTCCAATCCTCTGATGATTTCGCCCTCATAGTAAATGCACCTAATCATGAGGGAATACAA 1380
PB1 245626 Method E  1321 CTCCAATCCTCTGATGATTTCGCCCTCATAGTAAATGCACCTAATCATGAGGGAATACAA 1380
PB1 245626 Method K  1321 CTCCAATCCTCTGATGATTTCGCCCTCATAGTAAATGCACCTAATCATGAGGGAATACAA 1380
PB1 245626 Method N  1321 CTCCAATCCTCTGATGATTTCGCCCTCATAGTAAATGCACCTAATCATGAGGGAATACAA 1380

PB1 245625 MiSeq     1381 GCAGGAGTGGATAGGTTCTATAGGACCTGCAAACTGGTCGGGATCAATATGAGCAAAAAG 1440
PB1 245626 Method A  1381 GCAGGAGTGGATAGGTTCTATAGGACCTGCAAACTGGTCGGGATCAATATGAGCAAAAAG 1440
PB1 245626 Method E  1381 GCAGGAGTGGATAGGTTCTATAGGACCTGCAAACTGGTCGGGATCAATATGAGCAAAAAG 1440
PB1 245626 Method K  1381 GCAGGAGTGGATAGGTTCTATAGGACCTGCAAACTGGTCGGGATCAATATGAGCAAAAAG 1440
PB1 245626 Method N  1381 GCAGGAGTGGATAGGTTCTATAGGACCTGCAAACTGGTCGGGATCAATATGAGCAAAAAG 1440

PB1 245625 MiSeq     1441 AAGTCTTACATAAACCGGACTGGAACATTTGAGTTCACAAGCTTTTTCTATCGCTATGGA 1500
PB1 245626 Method A  1441 AAGTCTTACATAAACCGGACTGGAACATTTGAGTTCACAAGCTTTTTCTATCGCTATGGA 1500
PB1 245626 Method E  1441 AAGTCTTACATAAACCGGACTGGAACATTTGAGTTCACAAGCTTTTTCTATCGCTATGGA 1500
PB1 245626 Method K  1441 AAGTCTTACATAAACCGGACTGGAACATTTGAGTTCACAAGCTTTTTCTATCGCTATGGA 1500
PB1 245626 Method N  1441 AAGTCTTACATAAACCGGACTGGAACATTTGAGTTCACAAGCTTTTTCTATCGCTATGGA 1500
```

**PB1 245625 MiSeq**	*1501*	TTTGTGGCTAACTTCAGTATGGAGCTGCCCAGCTTTGGAGTTTCTGGGATCAATGAATCA	*1560*
**PB1 245626 Method A**	*1501*	TTTGTGGCTAACTTCAGTATGGAGCTGCCCAGCTTTGGAGTTTCTGGGATCAATGAATCA	*1560*
**PB1 245626 Method E**	*1501*	TTTGTGGCTAACTTCAGTATGGAGCTGCCCAGCTTTGGAGTTTCTGGGATCAATGAATCA	*1560*
**PB1 245626 Method K**	*1501*	TTTGTGGCTAACTTCAGTATGGAGCTGCCCAGCTTTGGAGTTTCTGGGATCAATGAATCA	*1560*
**PB1 245626 Method N**	*1501*	TTTGTGGCTAACTTCAGTATGGAGCTGCCCAGCTTTGGAGTTTCTGGGATCAATGAATCA	*1560*

**PB1 245625 MiSeq**	*1561*	GCTGACATGAGCATTGGCGTCACAGTGATAAAAAACAACATGATAAACAATGACCTTGGT	*1620*
**PB1 245626 Method A**	*1561*	GCTGACATGAGCATTGGCGTCACAGTGATAAAAAACAACATGATAAACAATGACCTTGGT	*1620*
**PB1 245626 Method E**	*1561*	GCTGACATGAGCATTGGCGTCACAGTGATAAAAAACAACATGATAAACAATGACCTTGGT	*1620*
**PB1 245626 Method K**	*1561*	GCTGACATGAGCATTGGCGTCACAGTGATAAAAAACAACATGATAAACAATGACCTTGGT	*1620*
**PB1 245626 Method N**	*1561*	GCTGACATGAGCATTGGCGTCACAGTGATAAAAAACAACATGATAAACAATGACCTTGGT	*1620*

**PB1 245625 MiSeq**	*1621*	CCAGCAACAGCTCAAATGGCCCTTCAACTATTCATCAAAGATTACAGGTACACGTACCGA	*1680*
**PB1 245626 Method A**	*1621*	CCAGCAACAGCTCAAATGGCCCTTCAACTATTCATCAAAGATTACAGGTACACGTACCGA	*1680*
**PB1 245626 Method E**	*1621*	CCAGCAACAGCTCAAATGGCCCTTCAACTATTCATCAAAGATTACAGGTACACGTACCGA	*1680*
**PB1 245626 Method K**	*1621*	CCAGCAACAGCTCAAATGGCCCTTCAACTATTCATCAAAGATTACAGGTACACGTACCGA	*1680*
**PB1 245626 Method N**	*1621*	CCAGCAACAGCTCAAATGGCCCTTCAACTATTCATCAAAGATTACAGGTACACGTACCGA	*1680*

**PB1 245625 MiSeq**	*1681*	TGCCACAGAGGTGACACACAAATTCAAACGAGGAGATCATTCGAGCTGAAGAAGCTGTGG	*1740*
**PB1 245626 Method A**	*1681*	TGCCACAGAGGTGACACACAAATTCAAACGAGGAGATCATTCGAGCTGAAGAAGCTGTGG	*1740*
**PB1 245626 Method E**	*1681*	TGCCACAGAGGTGACACACAAATTCAAACGAGGAGATCATTCGAGCTGAAGAAGCTGTGG	*1740*
**PB1 245626 Method K**	*1681*	TGCCACAGAGGTGACACACAAATTCAAACGAGGAGATCATTCGAGCTGAAGAAGCTGTGG	*1740*
**PB1 245626 Method N**	*1681*	TGCCACAGAGGTGACACACAAATTCAAACGAGGAGATCATTCGAGCTGAAGAAGCTGTGG	*1740*

**PB1 245625 MiSeq**	*1741*	GAACAGACCCGTTCAAAGGCAGGACTGTTGGTGTCAGATGGAGGACCAAATCTATACAAC	*1800*
**PB1 245626 Method A**	*1741*	GAACAGACCCGTTCAAAGGCAGGACTGTTGGTGTCAGATGGAGGACCAAATCTATACAAC	*1800*
**PB1 245626 Method E**	*1741*	GAACAGACCCGTTCAAAGGCAGGACTGTTGGTGTCAGATGGAGGACCAAATCTATACAAC	*1800*
**PB1 245626 Method K**	*1741*	GAACAGACCCGTTCAAAGGCAGGACTGTTGGTGTCAGATGGAGGACCAAATCTATACAAC	*1800*
**PB1 245626 Method N**	*1741*	GAACAGACCCGTTCAAAGGCAGGACTGTTGGTGTCAGATGGAGGACCAAATCTATACAAC	*1800*

**PB1 245625 MiSeq**	*1801*	ATTCGGAATCTCCATATCCCAGAGGTCTGCCTGAAGTGGGAGCTGATGGACGAAGATTAC	*1860*
**PB1 245626 Method A**	*1801*	ATTCGGAATCTCCATATCCCAGAGGTCTGCCTGAAGTGGGAGCTGATGGACGAAGATTAC	*1860*
**PB1 245626 Method E**	*1801*	ATTCGGAATCTCCATATCCCAGAGGTCTGCCTGAAGTGGGAGCTGATGGACGAAGATTAC	*1860*
**PB1 245626 Method K**	*1801*	ATTCGGAATCTCCATATCCCAGAGGTCTGCCTGAAGTGGGAGCTGATGGACGAAGATTAC	*1860*
**PB1 245626 Method N**	*1801*	ATTCGGAATCTCCATATCCCAGAGGTCTGCCTGAAGTGGGAGCTGATGGACGAAGATTAC	*1860*
**PB1 245625 MiSeq**	*1861*	CAGGGCAGGTTGTGTAATCCTCTGAACCCATTTGTCAGTCATAAAGAAATTGAGTCCGTA	*1920*
**PB1 245626 Method A**	*1861*	CAGGGCAGGTTGTGTAATCCTCTGAACCCATTTGTCAGTCATAAAGAAATTGAGTCCGTA	*1920*
**PB1 245626 Method E**	*1861*	CAGGGCAGGTTGTGTAATCCTCTGAACCCATTTGTCAGTCATAAAGAAATTGAGTCCGTA	*1920*
**PB1 245626 Method K**	*1861*	CAGGGCAGGTTGTGTAATCCTCTGAACCCATTTGTCAGTCATAAAGAAATTGAGTCCGTA	*1920*
**PB1 245626 Method N**	*1861*	CAGGGCAGGTTGTGTAATCTTCTGAACCCATTTGTCAGTCATAAAGAAATTGAGTCCGTA	*1920*
**PB1 245625 MiSeq**	*1921*	AACAATGCTGTGGTGATGCCAGCACACGGTCCAGCCAAAAGCATGGAATATGATGCCATT	*1980*
**PB1 245626 Method A**	*1921*	AACAATGCTGTGGTGATGCCAGCACACGGTCCAGCCAAAAGCATGGAATATGATGCCATT	*1980*
**PB1 245626 Method E**	*1921*	AACAATGCTGTGGTGATGCCAGCACACGGTCCAGCCAAAAGCATGGAATATGATGCCATT	*1980*
**PB1 245626 Method K**	*1921*	AACAATGCTGTGGTGATGCCAGCACACGGTCCAGCCAAAAGCATGGAATATGATGCCATT	*1980*
**PB1 245626 Method N**	*1921*	AACAATGCTGTGGTGATGCCAGCACACGGTCCAGCCAAAAGCATGGAATATGATGCCATT	*1980*
**PB1 245625 MiSeq**	*1981*	GCGACTACACACTCATGGATTCCTAAAAGGAATCGTTCCATTCTCAATACCAGTCAAAGG	*2040*
**PB1 245626 Method A**	*1981*	GCGACTACACACTCATGGATTCCTAAAAGGAATCGTTCCATTCTCAATACCAGTCAAAGG	*2040*
**PB1 245626 Method E**	*1981*	GCGACTACACACTCATGGATTCCTAAAAGGAATCGTTCCATTCTCAATACCAGTCAAAGG	*2040*
**PB1 245626 Method K**	*1981*	GCGACTACACACTCATGGATTCCTAAAAGGAATCGTTCCATTCTCAATACCAGTCAAAGG	*2040*
**PB1 245626 Method N**	*1981*	GCGACTACACACTCATGGATTCCTAAAAGGAATCGTTCCATTCTCAATACCAGTCAAAGG	*2040*
**PB1 245625 MiSeq**	*2041*	GGAATTCTTGAGGATGAACAGATGTACCAGAAATGCTGCAGTCTATTCGAAAAATTTTTT	*2100*
**PB1 245626 Method A**	*2041*	GGAATTCTTGAGGATGAACAGATGTACCAGAAATGCTGCAGTCTATTCGAAAAATTTTTT	*2100*
**PB1 245626 Method E**	*2041*	GGAATTCTTGAGGATGAACAGATGTACCAGAAATGCTGCAGTCTATTCGAAAAATTTTTT	*2100*
**PB1 245626 Method K**	*2041*	GGAATTCTTGAGGATGAACAGATGTACCAGAAATGCTGCAGTCTATTCGAAAAATTTTTT	*2100*
**PB1 245626 Method N**	*2041*	GGAATTCTTGAGGATGAACAGATGTACCAGAAATGCTGCAGTCTATTCGAAAAATTTTTT	*2100*

**PB1 245625 MiSeq**	*2101*	CCCAGTAGTTCATACAGGAGACCAGTTGGAATTTCCAGCATGGTGGAGGCCATGGTGTCT	*2160*
**PB1 245626 Method A**	*2101*	CCCAGTAGTTCATACAGGAGACCAGTTGGAATTTCCAGCATGGTGGAGGCCATGGTGTCT	*2160*
**PB1 245626 Method E**	*2101*	CCCAGTAGTTCATACAGGAGACCAGTTGGAATTTCCAGCATGGTGGAGGCCATGGTGTCT	*2160*
**PB1 245626 Method K**	*2101*	CCCAGTAGTTCATACAGGAGACCAGTTGGAATTTCCAGCATGGTGGAGGCCATGGTGTCT	*2160*
**PB1 245626 Method N**	*2101*	CCCAGTAGTTCATACAGGAGACCAGTTGGAATTTCCAGCATGGTGGAGGCCATGGTGTCT	*2160*

**PB1 245625 MiSeq**	*2161*	AGGGCCCGAATCGATGCACGCATTGATTTCGAATCTGGAAGGATCAAGAAGGGAGAGTTT	*2220*
**PB1 245626 Method A**	*2161*	AGGGCCCGAATCGATGCACGCATTGATTTCGAATCTGGAAGGATCAAGAAGGGAGAGTTT	*2220*
**PB1 245626 Method E**	*2161*	AGGGCCCGAATCGATGCACGCATTGATTTCGAATCTGGAAGGATCAAGAAGGGAGAGTTT	*2220*
**PB1 245626 Method K**	*2161*	AGGGCCCGAATCGATGCACGCATTGATTTCGAATCTGGAAGGATCAAGAAGGGAGAGTTT	*2220*
**PB1 245626 Method N**	*2161*	AGGGCCCGAATCGATGCACGCATTGATTTCGAATCTGGAAGGATCAAGAAGGGAGAGTTT	*2220*

**PB1 245625 MiSeq**	*2221*	TCTGAGATCATGAAGATCTGTTCCACCATTGAAGAGCTCAGACGGCAAAAATAG	*2274*
**PB1 245626 Method A**	*2221*	TCTGAGATCATGAAGATCTGTTCCACCATTGAAGAGCTCAGACGGCAAAAATAG	*2274*
**PB1 245626 Method E**	*2221*	TCTGAGATCATGAAGATCTGTTCCACCATTGAAGAGCTCAGACGGCAAAAATAG	*2274*
**PB1 245626 Method K**	*2221*	TCTGAGATCATGAAGATCTGTTCCACCATTGAAGAGCTCAGACGGCAAAAATAG	*2274*
**PB1 245626 Method N**	*2221*	TCTGAGATCATGAAGATCTGTTCCACCATTGAAGAGCTCAGACGGCAAAAATAG	*2274*
