# Supplementary material for: An Evaluation of Avian Influenza Virus Whole-Genome Sequencing Approaches Using Nanopore Technology
Source: Microorganisms. 2023 Feb 19;11(2):529. doi: 10.3390/microorganisms11020529 (PMC9967579; doi:10.3390/microorganisms11020529)
Supplement: Supplementary file 1 [file microorganisms-11-00529-s001.zip › manuscript.v8 230219 Suppl Figures and Tables/Supplementary Figures S2a-h 245626/Supplementary Figure S2f N1.pdf]

Formatted Alignments

**N1 245625 MiSeq**	*1*	ATGAATCCAAATCAAAAGATAACAACCATTGGATCAATCTGTATGGTAATTGGGATAGTC	*60*
**N1 245626 Method A**	*1*	ATGAATCCAAATCAAAAGATAACAACCATTGGATCAATCTGTATGGTAATTGGGATAGTC	*60*
**N1 245626 Method S**	*1*	ATGAATCCAAATCAAAAGATAACAACCATTGGATCAATCTGTATGGTAATTGGGATAGTC	*60*
**N1 245626 Method K**	*1*	ATGAATCCAAATCAAAAGATAACAACCATTGGATCAATCTGTATGGTAATTGGGATAGTC	*60*
**N1 245626 Method N**	*1*	ATGAATCCAAATCAAAAGATAACAACCATTGGATCAATCTGTATGGTAATTGGGATAGTC	*60*

**N1 245625 MiSeq**	*61*	AGCTTGATGCTGCAAATTGGGAACATAATCTCAATATGGGTTAGCCATTCAATCCAAACA	*120*
**N1 245626 Method A**	*61*	AGCTTGATGCTGCAAATTGGGAACATAATCTCAATATGGGTTAGCCATTCAATCCAAACA	*120*
**N1 245626 Method S**	*61*	AGCTTGATGCTGCAAATTGGGAACATAATCTCAATATGGGTTAGCCATTCAATCCAAACA	*120*
**N1 245626 Method K**	*61*	AGCTTGATGCTGCAAATTGGGAACATAATCTCAATATGGGTTAGCCATTCAATCCAAACA	*120*
**N1 245626 Method N**	*61*	AGCTTGATGCTGCAAATTGGGAACATAATCTCAATATGGGTTAGCCATTCAATCCAAACA	*120*

**N1 245625 MiSeq**	*121*	GGGAATCAATACCAGCCTGAACCATGCAATCAAAGCATCATTACCTATGAGAACAACACC	*180*
**N1 245626 Method A**	*121*	GGGAATCAATACCAGCCTGAACCATGCAATCAAAGCATCATTACCTATGAGAACAACACC	*180*
**N1 245626 Method S**	*121*	GGGAATCAATACCAGCCTGAACCATGCAATCAAAGCATCATTACCTATGAGAACAACACC	*180*
**N1 245626 Method K**	*121*	GGGAATCAATACCAGCCTGAACCATGCAATCAAAGCATCATTACCTATGAGAACAACACC	*180*
**N1 245626 Method N**	*121*	GGGAATCAATACCAGCCTGAACCATGCAATCAAAGCATCATTACCTATGAGAACAACACC	*180*

**N1 245625 MiSeq**	*181*	TGGGTAAATCAGACGTATGTCAACATCAGCAATACCAATTTTCTTGCTGAGCAGGCTGTT	*240*
**N1 245626 Method A**	*181*	TGGGTAAATCAGACGTATGTCAACATCAGCAATACCAATTTTCTTGCTGAGCAGGCTGTT	*240*
**N1 245626 Method S**	*181*	TGGGTAAATCAGACGTATGTCAACATCAGCAATACCAATTTTCTTGCTGAGCAGGCTGTT	*240*
**N1 245626 Method K**	*181*	TGGGTAAATCAGACGTATGTCAACATCAGCAATACCAATTTTCTTGCTGAGCAGGCTGTT	*240*
**N1 245626 Method N**	*181*	TGGGTAAATCAGACGTATGTCAACATCAGCAATACCAATTTTCTTGCTGAGCAGGCTGTT	*240*

**N1 245625 MiSeq**	*241*	ACTTCGGTAACATTAGCGGGCAATTCATCTCTTTGCCCTATTAGTGGGTGGGCAATATAC	*300*
**N1 245626 Method A**	*241*	ACTTCGGTAACATTAGCGGGCAATTCATCTCTTTGCCCTATTAGTGGGTGGGCAATATAC	*300*
**N1 245626 Method S**	*241*	ACTTCGGTAACATTAGCGGGCAATTCATCTCTTTGCCCTATTAGTGGGTGGGCAATATAC	*300*
**N1 245626 Method K**	*241*	ACTTCGGTAACATTAGCGGGCAATTCATCTCTTTGCCCTATTAGTGGGTGGGCAATATAC	*300*
**N1 245626 Method N**	*241*	ACTTCGGTAACATTAGCGGGCAATTCATCTCTTTGCCCTATTAGTGGGTGGGCAATATAC	*300*

**N1 245625 MiSeq**	*301*	AGTAAGGACAACGGTATAAGAATTGGGTCCAAGGGGGATGTGTTTGTTATAAGAGAACCG	*360*
**N1 245626 Method A**	*301*	AGTAAGGACAACGGTATAAGAATTGGGTCCAAGGGGGATGTGTTTGTTATAAGAGAACCG	*360*
**N1 245626 Method S**	*301*	AGTAAGGACAACGGTATAAGAATTGGGTCCAAGGGGGATGTGTTTGTTATAAGAGAACCG	*360*
**N1 245626 Method K**	*301*	AGTAAGGACAACGGTATAAGAATTGGGTCCAAGGGGGATGTGTTTGTTATAAGAGAACCG	*360*
**N1 245626 Method N**	*301*	AGTAAGGACAACGGTATAAGAATTGGGTCCAAGGGGGATGTGTTTGTTATAAGAGAACCG	*360*
**N1 245625 MiSeq**	*361*	TTCATCTCATGCTCCCACTTGGAATGCAGAACCTTTTTCCTGACCCAGGGAGCTCTGCTG	*420*
**N1 245626 Method A**	*361*	TTCATCTCATGCTCCCACTTGGAATGCAGAACCTTTTTCCTGACCCAGGGAGCTCTGCTG	*420*
**N1 245626 Method S**	*361*	TTCATCTCATGCTCCCACTTGGAATGCAGAACCTTTTTCCTGACCCAGGGAGCTCTGCTG	*420*
**N1 245626 Method K**	*361*	TTCATCTCATGCTCCCACTTGGAATGCAGAACCTTTTTCCTGACCCAGGGAGCTCTGCTG	*420*
**N1 245626 Method N**	*361*	TTCATCTCATGCTCCCACTTGGAATGCAGAACCTTTTTCCTGACCCAGGGAGCTCTGCTG	*420*
**N1 245625 MiSeq**	*421*	AATGACAAACATTCTAATGGGACCGTTAAGGATAGAAGCCCTTATAGAACTTTGATGAGT	*480*
**N1 245626 Method A**	*421*	AATGACAAACATTCTAATGGGACCGTTAAGGATAGAAGCCCTTATAGAACTTTGATGAGT	*480*
**N1 245626 Method S**	*421*	AATGACAAACATTCTAATGGGACCGTTAAGGATAGAAGCCCTTATAGAACTTTGATGAGT	*480*
**N1 245626 Method K**	*421*	AATGACAAACATTCTAATGGGACCGTTAAGGATAGAAGCCCTTATAGAACTTTGATGAGT	*480*
**N1 245626 Method N**	*421*	AATGACAAACATTCTAATGGGACCGTTAAGGATAGAAGCCCTTATAGAACTTTGATGAGT	*480*
**N1 245625 MiSeq**	*481*	TGTCCCGTGGGTGAGGCTCCTTCCCCGTACAATTCAAGATTTGAGTCTGTTGCTTGGTCG	*540*
**N1 245626 Method A**	*481*	TGTCCCGTGGGTGAGGCTCCTTCCCCGTACAATTCAAGATTTGAGTCTGTTGCTTGGTCG	*540*
**N1 245626 Method S**	*481*	TGTCCCGTGGGTGAGGCTCCTTCCCCGTACAATTCAAGATTTGAGTCTGTTGCTTGGTCG	*540*
**N1 245626 Method K**	*481*	TGTCCCGTGGGTGAGGCTCCTTCCCCGTACAATTCAAGATTTGAGTCTGTTGCTTGGTCG	*540*
**N1 245626 Method N**	*481*	TGTCCCGTGGGTGAGGCTCCTTCCCCGTACAATTCAAGATTTGAGTCTGTTGCTTGGTCG	*540*
**N1 245625 MiSeq**	*541*	GCAAGTGCTTGTCATGATGGCATCAGTTGGTTGACAATCGGTATTTCTGGTCCAGACAAT	*600*
**N1 245626 Method A**	*541*	GCAAGTGCTTGTCATGATGGCATCAGTTGGTTGACAATCGGTATTTCTGGTCCAGACAAT	*600*
**N1 245626 Method S**	*541*	GCAAGTGCTTGTCATGATGGCATCAGTTGGTTGACAATCGGTATTTCTGGTCCAGACAAT	*600*
**N1 245626 Method K**	*541*	GCAAGTGCTTGTCATGATGGCATCAGTTGGTTGACAATCGGTATTTCTGGTCCAGACAAT	*600*
**N1 245626 Method N**	*541*	GCAAGTGCTTGTCATGATGGCATCAGTTGGTTGACAATCGGTATTTCTGGTCCAGACAAT	*600*

**N1 245625 MiSeq**	*601*	GGAGCTGTGGCTGTATTGAAGTACAATGGCATAATAACGGATACTATCAAGAGTTGGAGA	*660*
**N1 245626 Method A**	*601*	GGAGCTGTGGCTGTATTGAAGTACAATGGCATAATAACGGATACTATCAAGAGTTGGAGA	*660*
**N1 245626 Method S**	*601*	GGAGCTGTGGCTGTATTGAAGTACAATGGCATAATAACGGATACTATCAAGAGTTGGAGA	*660*
**N1 245626 Method K**	*601*	GGAGCTGTGGCTGTATTGAAGTACAATGGCATAATAACGGATACTATCAAGAGTTGGAGA	*660*
**N1 245626 Method N**	*601*	GGAGCTGTGGCTGTATTGAAGTACAATGGCATAATAACGGATACTATCAAGAGTTGGAGA	*660*

**N1 245625 MiSeq**	*661*	AACAACATTTTGAGAACTCAAGAATCTGAATGTGCGTGCGTAAATGGCTCTTGCTTCACC	*720*
**N1 245626 Method A**	*661*	AACAACATTTTGAGAACTCAAGAATCTGAATGTGCGTGCGTAAATGGCTCTTGCTTCACC	*720*
**N1 245626 Method S**	*661*	AACAACATTTTGAGAACTCAAGAATCTGAATGTGCGTGCGTAAATGGCTCTTGCTTCACC	*720*
**N1 245626 Method K**	*661*	AACAACATTTTGAGAACTCAAGAATCTGAATGTGCGTGCGTAAATGGCTCTTGCTTCACC	*720*
**N1 245626 Method N**	*661*	AACAACATTTTGAGAACTCAAGAATCTGAATGTGCGTGCGTAAATGGCTCTTGCTTCACC	*720*

**N1 245625 MiSeq**	*721*	GTAATGACTGATGGACCAAGCAATGGGCAGGCCTCATATAAAATCTTCAAGATAGAGAAA	*780*
**N1 245626 Method A**	*721*	GTAATGACTGATGGACCAAGCAATGGGCAGGCCTCATATAAAATCTTCAAGATAGAGAAA	*780*
**N1 245626 Method S**	*721*	GTAATGACTGATGGACCAAGCAATGGGCAGGCCTCATATAAAATCTTCAAGATAGAGAAA	*780*
**N1 245626 Method K**	*721*	GTAATGACTGATGGACCAAGCAATGGGCAGGCCTCATATAAAATCTTCAAGATAGAGAAA	*780*
**N1 245626 Method N**	*721*	GTAATGACTGATGGACCAAGCAATGGGCAGGCCTCATATAAAATCTTCAAGATAGAGAAA	*780*

**N1 245625 MiSeq**	*781*	GGGAAAGTTGTCAAATCAGTTGAATTGAATGCCCCTAATTACCACTACGAGGAATGCTCC	*840*
**N1 245626 Method A**	*781*	GGGAAAGTTGTCAAATCAGTTGAATTGAATGCCCCTAATTACCACTACGAGGAATGCTCC	*840*
**N1 245626 Method S**	*781*	GGGAAAGTTGTCAAATCAGTTGAATTGAATGCCCCTAATTACCACTACGAGGAATGCTCC	*840*
**N1 245626 Method K**	*781*	GGGAAAGTTGTCAAATCAGTTGAATTGAATGCCCCTAATTACCACTACGAGGAATGCTCC	*840*
**N1 245626 Method N**	*781*	GGGAAAGTTGTCAAATCAGTTGAATTGAATGCCCCTAATTACCACTACGAGGAATGCTCC	*840*

**N1 245625 MiSeq**	*841*	TGTTATCCTGATGCGGGTGATATTATGTGTGTGTGCAGGGACAATTGGCATGGCTCAAAC	*900*
**N1 245626 Method A**	*841*	TGTTATCCTGATGCGGGTGATATTATGTGTGTGTGCAGGGACAATTGGCATGGCTCAAAC	*900*
**N1 245626 Method S**	*841*	TGTTATCCTGATTCGGGTGATATTATGTGTGTGTGCAGGGACAATTGGCATGGCTCAAAC	*900*
**N1 245626 Method K**	*841*	TGTTATCCTGATGCGGGTGATATTATGTGTGTGTGCAGGGACAATTGGCATGGCTCAAAC	*900*
**N1 245626 Method N**	*841*	TGTTATCCTGATGCGGGTGATATTATGTGTGTGTGCAGGGACAATTGGCATGGCTCAAAC	*900*

**N1 245625 MiSeq**	*901*	CGGCCGTGGGTATCTTTTAATCAAAATCTGGAGTATCAAATAGGATATATATGCAGTGGG	*960*
**N1 245626 Method A**	*901*	CGGCCGTGGGTATCTTTTAATCAAAATCTGGAGTATCAAATAGGATATATATGCAGTGGG	*960*
**N1 245626 Method S**	*901*	CGGCCGTGGGTATCTTTTAATCAAAATCTGGAGTATCAAATAGGATATATATGCAGTGGG	*960*
**N1 245626 Method K**	*901*	CGGCCGTGGGTATCTTTTAATCAAAATCTGGAGTATCAAATAGGATATATATGCAGTGGG	*960*
**N1 245626 Method N**	*901*	CGGCCGTGGGTATCTTTTAATCAAAATCTGGAGTATCAAATAGGATATATATGCAGTGGG	*960*
**N1 245625 MiSeq**	*961*	GTTTTCGGGGACAATCCCCGCCCCAATGATGGAACAGGCAGTTGCAGTCCAATGTCCTCT	*1020*
**N1 245626 Method A**	*961*	GTTTTCGGGGACAATCCCCGCCCCAATGATGGAACAGGCAGTTGCAGTCCAATGTCCTCT	*1020*
**N1 245626 Method S**	*961*	GTTTTCGGGGACAATCCCCGCCCCAATGATGGAACAGGCAGTTGCAGTCCAATGTCCTCT	*1020*
**N1 245626 Method K**	*961*	GTTTTCGGGGACAATCCCCGCCCCAATGATGGAACAGGCAGTTGCAGTCCAATGTCCTCT	*1020*
**N1 245626 Method N**	*961*	GTTTTCGGGGACAATCCCCGCCCCAATGATGGAACAGGCAGTTGCAGTCCAATGTCCTCT	*1020*
**N1 245625 MiSeq**	*1021*	AATGGGGCATATGGGGTAAAAGGGTTTTCATTTAAGTACGGTAATGGGGTTTGGATCGGA	*1080*
**N1 245626 Method A**	*1021*	AATGGGGCATATGGGGTAAAAGGGTTTTCATTTAAGTACGGTAATGGGGTTTGGATCGGA	*1080*
**N1 245626 Method S**	*1021*	AATGGGGCATATGGGGTAAAAGGGTTTTCATTTAAGTACGGTAATGGGGTTTGGATCGGA	*1080*
**N1 245626 Method K**	*1021*	AATGGGGCATATGGGGTAAAAGGGTTTTCATTTAAGTACGGTAATGGGGTTTGGATCGGA	*1080*
**N1 245626 Method N**	*1021*	AATGGGGCATATGGGGTAAAAGGGTTTTCATTTAAGTACGGTAATGGGGTTTGGATCGGA	*1080*
**N1 245625 MiSeq**	*1081*	AGAACAAAAAGCACTAGTTCCAGAAGCGGCTTTGAGATGATTTGGGATCCGAATGGGTGG	*1140*
**N1 245626 Method A**	*1081*	AGAACAAAAAGCACTAGTTCCAGAAGCGGCTTTGAGATGATTTGGGATCCGAATGGGTGG	*1140*
**N1 245626 Method S**	*1081*	AGAACAAAAAGCACTAGTTCCAGAAGCGGCTTTGAGATGATTTGGGATCCGAATGGGTGG	*1140*
**N1 245626 Method K**	*1081*	AGAACAAAAAGCACTAGTTCCAGAAGCGGCTTTGAGATGATTTGGGATCCGAATGGGTGG	*1140*
**N1 245626 Method N**	*1081*	AGAACAAAAAGCACTAGTTCCAGAAGCGGCTTTGAGATGATTTGGGATCCGAATGGGTGG	*1140*
**N1 245625 MiSeq**	*1141*	ACTGAGACGGACAGTAGTTTCTCAGTGAAGCAAGACATTGTAGAAATAACTGACTGGTCA	*1200*
**N1 245626 Method A**	*1141*	ACTGAGACGGACAGTAGTTTCTCAGTGAAGCAAGACATTGTAGAAATAACTGACTGGTCA	*1200*
**N1 245626 Method S**	*1141*	ACTGAGACGGACAGTAGTTTCTCAGTGAAGCAAGACATTGTAGAAATAACTGACTGGTCA	*1200*
**N1 245626 Method K**	*1141*	ACTGAGACGGACAGTAGTTTCTCAGTGAAGCAAGACATTGTAGAAATAACTGACTGGTCA	*1200*
**N1 245626 Method N**	*1141*	ACTGAGACGGACAGTAGTTTCTCAGTGAAGCAAGACATTGTAGAAATAACTGACTGGTCA	*1200*

**N1 245625 MiSeq**	*1201*	GGATATAGTGGGAGTTTTGTCCAGCATCCAGAACTGACAGGATTAGATTGCATGAGGCCT	*1260*
**N1 245626 Method A**	*1201*	GGATATAGTGGGAGTTTTGTCCAGCATCCAGAACTGACAGGATTAGATTGCATGAGGCCT	*1260*
**N1 245626 Method S**	*1201*	GGATATAGTGGGAGTTTTGTCCAGCATCCAGAACTGACAGGATTAGATTGCATGAGGCCT	*1260*
**N1 245626 Method K**	*1201*	GGATATAGTGGGAGTTTTGTCCAGCATCCAGAACTGACAGGATTAGATTGCATGAGGCCT	*1260*
**N1 245626 Method N**	*1201*	GGATATAGTGGGAGTTTTGTCCAGCATCCAGAACTGACAGGATTAGATTGCATGAGGCCT	*1260*

**N1 245625 MiSeq**	*1261*	TGTTTCTGGGTTGAGCTAATTAGAGGGAGGCCCAAAGAGAACACAATTTGGACTAGCGGG	*1320*
**N1 245626 Method A**	*1261*	TGTTTCTGGGTTGAGCTAATTAGAGGGAGGCCCAAAGAGAACACAATTTGGACTAGCGGG	*1320*
**N1 245626 Method S**	*1261*	TGTTTCTGGGTTGAGCTAATTAGAGGGAGGCCCAAAGAGAACACAATTTGGACTAGCGGG	*1320*
**N1 245626 Method K**	*1261*	TGTTTCTGGGTTGAGCTAATTAGAGGGAGGCCCAAAGAGAACACAATTTGGACTAGCGGG	*1320*
**N1 245626 Method N**	*1261*	TGTTTCTGGGTTGAGCTAATTAGAGGGAGGCCCAAAGAGAACACAATTTGGACTAGCGGG	*1320*

**N1 245625 MiSeq**	*1321*	AGCAGCATATCCTTTTGTGGTGTAAATAGTGACACTGTGGGTTGGTCTTGGCCAGACGGT	*1380*
**N1 245626 Method A**	*1321*	AGCAGCATATCCTTTTGTGGTGTAAATAGTGACACTGTGGGTTGGTCTTGGCCAGACGGT	*1380*
**N1 245626 Method S**	*1321*	AGCAGCATATCCTTTTGTGGTGTAAATAGTGACACTGTGGGTTGGTCTTGGCCAGACGGT	*1380*
**N1 245626 Method K**	*1321*	AGCAGCATATCCTTTTGTGGTGTAAATAGTGACACTGTGGGTTGGTCTTGGCCAGACGGT	*1380*
**N1 245626 Method N**	*1321*	AGCAGCATATCCTTTTGTGGTGTAAATAGTGACACTGTGGGTTGGTCTTGGCCAGACGGT	*1380*

**N1 245625 MiSeq**	*1381*	GCTGAGTTGCCATTCACCATTGACAAGTAG	*1410*
**N1 245626 Method A**	*1381*	GCTGAGTTGCCATTCACCATTGACAAGTAG	*1410*
**N1 245626 Method S**	*1381*	GCTGAGTTGCCATTCACCATTGACAAGTAG	*1410*
**N1 245626 Method K**	*1381*	GCTGAGTTGCCATTCACCATTGACAAGTAG	*1410*
**N1 245626 Method N**	*1381*	GCTGAGTTGCCATTCACCATTGACAAGTAG	*1410*
