# Supplementary material for: An Evaluation of Avian Influenza Virus Whole-Genome Sequencing Approaches Using Nanopore Technology
Source: Microorganisms. 2023 Feb 19;11(2):529. doi: 10.3390/microorganisms11020529 (PMC9967579; doi:10.3390/microorganisms11020529)
Supplement: Supplementary file 1 [file microorganisms-11-00529-s001.zip › manuscript.v8 230219 Suppl Figures and Tables/Supplementary Figures S2a-h 245626/Supplementary Figure S2h NS.pdf]

Formatted Alignments

NS 245625 MiSeq	1	ATGGATTCCAACACTGTGTCAAGCTTTCAGGTAGACTGCTTTCTTTGGCATGTCCGCAAA	60
NS 245626 Method A	1	ATGGATTCCAACACTGTGTCAAGCTTTCAGGTAGACTGCTTTCTTTGGCATGTCCGCAAA	60
NS 245626 Method S	1	ATGGATTCCAACACTGTGTCAAGCTTTCAGGTAGACTGCTTTCTTTGGCATGTCCGCAAA	60
NS 245626 Method K	1	ATGGATTCCAACACTGTGTCAAGCTTTCAGGTAGACTGCTTTCTTTGGCATGTCCGCAAA	60
NS 245626 Method N	1	ATGGATTCCAACACTGTGTCAAGCTTTCAGGTAGACTGCTTTCTTTGGCATGTCCGCAAA	60
NS 245625 MiSeq	61	CGATTTGCAGACCAAGAACTGGGTGATGCCCCATTCCTTGACCGGCTTCGCCGAGATCAG	120
NS 245626 Method A	61	CGATTTGCAGACCAAGAACTGGGTGATGCCCCATTCCTTGACCGGCTTCGCCGAGATCAG	120
NS 245626 Method S	61	CGATTTGCAGACCAAGAACTGGGTGATGCCCCATTCCTTGACCGGCTTCGCCGAGATCAG	120
NS 245626 Method K	61	CGATTTGCAGACCAAGAACTGGGTGATGCCCCATTCCTTGACCGGCTTCGCCGAGATCAG	120
NS 245626 Method N	61	CGATTTGCAGACCAAGAACTGGGTGATGCCCCATTCCTTGACCGGCTTCGCCGAGATCAG	120
NS 245625 MiSeq	121	AAATCCCTGAGAGGAAGAGGCAGCACTCTTGGTCTGGGCATCGAAACAGCCACCCGTGCA	180
NS 245626 Method A	121	AAATCCCTGAGAGGAAGAGGCAGCACTCTTGGTCTGGGCATCGAAACAGCCACCCGTGCA	180
NS 245626 Method S	121	AAATCCCTGAGAGGAAGAGGCAGCACTCTTGGTCTGGGCATCGAAACAGCCACCCGTGCA	180
NS 245626 Method K	121	AAATCCCTGAGAGGAAGAGGCAGCACTCTTGGTCTGGGCATCGAAACAGCCACCCGTGCA	180
NS 245626 Method N	121	AAATCCCTGAGAGGAAGAGGCAGCACTCTTGGTCTGGGCATCGAAACAGCCACCCGTGCA	180
NS 245625 MiSeq	181	GGAAAGCAGATAGTGGAGCGGATTCTGGAAGAAGAATCTGATGAGACACTTAAAATGACT	240
NS 245626 Method A	181	GGAAAGCAGATAGTGGAGCGGATTCTGGAAGAAGAATCTGATGAGACACTTAAAATGACT	240
NS 245626 Method S	181	GGAAAGCAGATAGTGGAGCGGATTCTGGAAGAAGAATCTGATGAGACACTTAAAATGACT	240
NS 245626 Method K	181	GGAAAGCAGATAGTGGAGCGGATTCTGGAAGAAGAATCTGATGAGACACTTAAAATGACT	240
NS 245626 Method N	181	GGAAAGCAGATAGTGGAGCGGATTCTGGAAGAAGAATCTGATGAGACACTTAAAATGACT	240
NS 245625 MiSeq	241	ATTGCCCCCGTGCCAGCTTCACGCTACCTAACTGACATGACTCTTGAGGAGATGTCAAGG	300
NS 245626 Method A	241	ATTGCCCCCGTGCCAGCTTCACGCTACCTAACTGACATGACTCTTGAGGAGATGTCAAGG	300
NS 245626 Method S	241	ATTGCCCCCGTGCCAGCTTCACGCTACCTAACTGACATGACTCTTGAGGAGATGTCAAGG	300
NS 245626 Method K	241	ATTGCCCCCGTGCCAGCTTCACGCTACCTAACTGACATGACTCTTGAGGAGATGTCAAGG	300
NS 245626 Method N	241	ATTGCCCCCGTGCCAGCTTCACGCTACCTAACTGACATGACTCTTGAGGAGATGTCAAGG	300

**NS 245625 MiSeq**	*301*	GACTGGTTCATGCTCATGCCCAAACAGAAAGTGGCAGGTTCCCTTTGCATCAGAATGGAC	*360*
**NS 245626 Method A**	*301*	GACTGGTTCATGCTCATGCCCAAACAGAAAGTGGCAGGTTCCCTTTGCATCAGAATGGAC	*360*
**NS 245626 Method S**	*301*	GACTGGTTCATGCTCATGCCCAAACAGAAAGTGGCAGGTTCCCTTTGCATCAGAATGGAC	*360*
**NS 245626 Method K**	*301*	GACTGGTTCATGCTCATGCCCAAACAGAAAGTGGCAGGTTCCCTTTGCATCAGAATGGAC	*360*
**NS 245626 Method N**	*301*	GACTGGTTCATGCTCATGCCCAAACAGAAAGTGGCAGGTTCCCTTTGCATCAGAATGGAC	*360*

**NS 245625 MiSeq**	*361*	CAGGCAATAATGGATAAAAACATCATATTGAAAGCAAACTTCAGTGTGATTTTTGACCGG	*420*
**NS 245626 Method A**	*361*	CAGGCAATAATGGATAAAAACATCATATTGAAAGCAAACTTCAGTGTGATTTTTGACCGG	*420*
**NS 245626 Method S**	*361*	CAGGCAATAATGGATAAAAACATCATATTGAAAGCAAACTTCAGTGTGATTTTTGACCGG	*420*
**NS 245626 Method K**	*361*	CAGGCAATAATGGATAAAAACATCATATTGAAAGCAAACTTCAGTGTGATTTTTGACCGG	*420*
**NS 245626 Method N**	*361*	CAGGCAATAATGGATAAAAACATCATATTGAAAGCAAACTTCAGTGTGATTTTTGACCGG	*420*

**NS 245625 MiSeq**	*421*	CTGGAAACCCTAATACTACTTAGAGCTTTCACAGAAGAAGGAGCAATTGTGGGAGAAATC	*480*
**NS 245626 Method A**	*421*	CTGGAAACCCTAATACTACTTAGAGCTTTCACAGAAGAAGGAGCAATTGTGGGAGAAATC	*480*
**NS 245626 Method S**	*421*	CTGGAAACCCTAATACTACTTAGAGCTTTCACAGAAGAAGGAGCAATTGTGGGAGAAATC	*480*
**NS 245626 Method K**	*421*	CTGGAAACCCTAATACTACTTAGAGCTTTCACAGAAGAAGGAGCAATTGTGGGAGAAATC	*480*
**NS 245626 Method N**	*421*	CTGGAAACCCTAATACTACTTAGAGCTTTCACAGAAGAAGGAGCAATTGTGGGAGAAATC	*480*

**NS 245625 MiSeq**	*481*	TCACCATTACCTTCTCTTCCAGGACATACTGATGAGGATGTCAAAAATGCAATTGGGGTC	*540*
**NS 245626 Method A**	*481*	TCACCATTACCTTCTCTTCCAGGACATACTGATGAGGATGTCAAAAATGCAATTGGGGTC	*540*
**NS 245626 Method S**	*481*	TCACCATTACCTTCTCTTCCAGGACATACTGATGAGGATGTCAAAAATGCAATTGGGGTC	*540*
**NS 245626 Method K**	*481*	TCACCATTACCTTCTCTTCCAGGACATACTGATGAGGATGTCAAAAATGCAATTGGGGTC	*540*
**NS 245626 Method N**	*481*	TCACCATTACCTTCTCTTCCAGGACATACTGATGAGGATGTCAAAAATGCAATTGGGGTC	*540*

**NS 245625 MiSeq**	*541*	CTCATCGGAGGACTTGAATGGAATGATAACACAGTTCGAGTCTCTGAAACTTTACAGAGA	*600*
**NS 245626 Method A**	*541*	CTCATCGGAGGACTTGAATGGAATGATAACACAGTTCGAGTCTCTGAAACTTTACAGAGA	*600*
**NS 245626 Method S**	*541*	CTCATCGGAGGACTTGAATGGAATGATAACACAGTTCGAGTCTCTGAAACTTTACAGAGA	*600*
**NS 245626 Method K**	*541*	CTCATCGGAGGACTTGAATGGAATGATAACACAGTTCGAGTCTCTGAAACTTTACAGAGA	*600*
**NS 245626 Method N**	*541*	CTCATCGGAGGACTTGAATGGAATGATAACACAGTTCGAGTCTCTGAAACTTTACAGAGA	*600*

**NS 245625 MiSeq**	*601*	TTCGCTTGGAGAAGCAGTAATGAGGATGGGAGACCTCCACTCCCTCCAAAGCAGAAACGG	*660*
**NS 245626 Method A**	*601*	TTCGCTTGGAGAAGCAGTAATGAGGATGGGAGACCTCCACTCCCTCCAAAGCAGAAACGG	*660*
**NS 245626 Method S**	*601*	TTCGCTTGGAGAAGCAGTAATGAGGATGGGAGACCTCCACTCCCTCCAAAGCAGAAACGG	*660*
**NS 245626 Method K**	*601*	TTCGCTTGGAGAAGCAGTAATGAGGATGGGAGACCTCCACTCCCTCCAAAGCAGAAACGG	*660*
**NS 245626 Method N**	*601*	TTCGCTTGGAGAAGCAGTAATGAGGATGGGAGACCTCCACTCCCTCCAAAGCAGAAACGG	*660*

**NS 245625 MiSeq**	*661*	AAAATGGCGAGAACAATTGAGTCAGAAGTTTGAAGAAATAAGATGGCTGATTGAAGGAGT	*720*
**NS 245626 Method A**	*661*	AAAATGGCGAGAACAATTGAGTCAGAAGTTTGAAGAAATAAGATGGCTGATTGAAGGAGT	*720*
**NS 245626 Method S**	*661*	AAAATGGCGAGAACAATTGAGTCAGAAGTTTGAAGAAATAAGATGGCTGATTGAAGGAGT	*720*
**NS 245626 Method K**	*661*	AAAATGGCGAGAACAATTGAGTCAGAAGTTTGAAGAAATAAGATGGCTGATTGAAGGAGT	*720*
**NS 245626 Method N**	*661*	AAAATGGCGAGAACAATTGAGTCAGAAGTTTGAAGAAATAAGATGGCTGATTGAAGGAGT	*720*

**NS 245625 MiSeq**	*721*	GCGGCACAGATTGAAGATTACAGAGAACAGTTTCGAACAAATAACTTTTATGCAAGCCTT	*780*
**NS 245626 Method A**	*721*	GCGGCACAGATTGAAGATTACAGAGAACAGTTTCGAACAAATAACTTTTATGCAAGCCTT	*780*
**NS 245626 Method S**	*721*	GCGGCACAGATTGAAGATTACAGAGAACAGTTTCGAACAAATAACTTTTATGCAAGCCTT	*780*
**NS 245626 Method K**	*721*	GCGGCACAGATTGAAGATTACAGAGAACAGTTTCGAACAAATAACTTTTATGCAAGCCTT	*780*
**NS 245626 Method N**	*721*	GCGGCACAGATTGAAGATTACAGAGAACAGTTTCGAACAAATAACTTTTATGCAAGCCTT	*780*

**NS 245625 MiSeq**	*781*	ACAACTATTGCTTGAAGTGGAGCAAGAGATAAGAACTTTCTCGTTTCAGCTTATTTAA	*838*
**NS 245626 Method A**	*781*	ACAACTATTGCTTGAAGTGGAGCAAGAGATAAGAACTTTCTCGTTTCAGCTTATTTAA	*838*
**NS 245626 Method S**	*781*	ACAACTATTGCTTGAAGTGGAGCAAGAGATAAGAACTTTCTCGTTTCAGCTTATTTAA	*838*
**NS 245626 Method K**	*781*	ACAACTATTGCTTGAAGTGGAGCAAGAGATAAGAACTTTCTCGTTTCAGCTTATTTAA	*838*
**NS 245626 Method N**	*781*	ACAACTATTGCTTGAAGTGGAGCAAGAGATAAGAACTTTCTCGTTTCAGCTTATTTAA	*838*
