# Supplementary material for: An Evaluation of Avian Influenza Virus Whole-Genome Sequencing Approaches Using Nanopore Technology
Source: Microorganisms. 2023 Feb 19;11(2):529. doi: 10.3390/microorganisms11020529 (PMC9967579; doi:10.3390/microorganisms11020529)
Supplement: Supplementary file 1 [file microorganisms-11-00529-s001.zip › manuscript.v8 230219 Suppl Figures and Tables/Supplementary Figures S2a-h 245626/Supplementary Figure S2a PB2.pdf]

Formatted Alignments

```
PB2 245625 MiSeq       1    ATGGAGAGAATAAAAGAACTAAGAGATCTAATGTCACAGTCTCGCACTCGCGAGATACTG   60
PB2 245626 Method A    1    ATGGAGAGAATAAAAGAAATAAGAGATCTAATGTCACAGTCTCGCACTCGCGAGATACTG   60
PB2 245626 Method E    1    ATGGAGAGAATAAAAGAAATAAGAGATCTAATGTCACAGTCTCGCACTCGCGAGATACTG   60
PB2 245626 Method K    1    ATGGAGAGAATAAAAGAAATAAGAGATCTAATGTCACAGTCTCGCACTCGCGAGATACTG   60
PB2 245626 Method N    1    ATGGAGAGAATAAAAGAAATAAGAGATCTAATGTCACAGTCTCGCACTCGCGAGATACTG   60

PB2 245625 MiSeq       61   ACCAAAACCACTGTTGACCACATGGCCATAATCAAAAAGTACACATCGGGAAGGCAAGAG   120
PB2 245626 Method A    61   ACCAAAACCACTGTTGACCACATGGCCATAATCAAAAAGTACACATCGGGAAGGCAAGAG   120
PB2 245626 Method E    61   ACCAAAACCACTGTTGACCACATGGCCATAATCAAAAAGTACACATCGGGAAGGCAAGAG   120
PB2 245626 Method K    61   ACCAAAACCACTGTTGACCACATGGCCATAATCAAAAAGTACACATCGGGAAGGCAAGAG   120
PB2 245626 Method N    61   ACCAAAACCACTGTTGACCACATGGCCATAATCAAAAAGTACACATCGGGAAGGCAAGAG   120

PB2 245625 MiSeq       121  AAGAACCCCGCACTCAGAATGAAATGGATGATGGCAATGAAATATCCAATTACAGCAGAT   180
PB2 245626 Method A    121  AAGAACCCCGCACTCAGAATGAAATGGATGATGGCAATGAAATATCCAATTACAGCAGAT   180
PB2 245626 Method E    121  AAGAACCCCGCACTCAGAATGAAATGGATGATGGCAATGAAATATCCAATTACAGCAGAT   180
PB2 245626 Method K    121  AAGAACCCCGCACTCAGAATGAAATGGATGATGGCAATGAAATATCCAATTACAGCAGAT   180
PB2 245626 Method N    121  AAGAACCCCGCACTCAGAATGAAATGGATGATGGCAATGAAATATCCAATTACAGCAGAT   180

PB2 245625 MiSeq       181  AAGCGAATAATGGAAATGATCCCTGAGAGGAATGAACAAGGGCAAACTCTCTGGAGCAAA   240
PB2 245626 Method A    181  AAGCGAATAATGGAAATGATCCCTGAGAGGAATGAACAAGGGCAAACTCTCTGGAGCAAA   240
PB2 245626 Method E    181  AAGCGAATAATGGAAATGATCCCTGAGAGGAATGAACAAGGGCAAACTCTCTGGAGCAAA   240
PB2 245626 Method K    181  AAGCGAATAATGGAAATGATCCCTGAGAGGAATGAACAAGGGCAAACTCTCTGGAGCAAA   240
PB2 245626 Method N    181  AAGCGAATAATGGAAATGATCCCTGAGAGGAATGAACAAGGGCAAACTCTCTGGAGCAAA   240

PB2 245625 MiSeq       241  ACAAACGATGCCGGATCAGACCGAGTGATGGTATCACCCCTGGCTGTGACATGGTGGAAC   300
PB2 245626 Method A    241  ACAAACGATGCCGGATCAGACCGAGTGATGGTATCACCCCTGGCTGTGACATGGTGGAAC   300
PB2 245626 Method E    241  ACAAACGATGCCGGATCAGACCGAGTGATGGTATCACCCCTGGCTGTGACATGGTGGAAC   300
PB2 245626 Method K    241  ACAAACGATGCCGGATCAGACCGAGTGATGGTATCACCCCTGGCTGTGACATGGTGGAAC   300
PB2 245626 Method N    241  ACAAACGATGCCGGATCAGACCGAGTGATGGTATCACCCCTGGCTGTGACATGGTGGAAC   300
```

**PB2 245625 MiSeq**	*301*	AGGAACGGACCAACAACAAGTACAGTTCACTACCCAAAGGTATATAAAACTTATTTCGAA	*360*
**PB2 245626 Method A**	*301*	AGGAACGGACCAACAACAAGTACAGTTCACTACCCAAAGGTATATAAAACTTATTTCGAA	*360*
**PB2 245626 Method E**	*301*	AGGAACGGACCAACAACAAGTACAGTTCACTACCCAAAGGTATATAAAACTTATTTCGAA	*360*
**PB2 245626 Method K**	*301*	AGGAACGGACCAACAACAAGTACAGTTCACTACCCAAAGGTATATAAAACTTATTTCGAA	*360*
**PB2 245626 Method N**	*301*	AGGAACGGACCAACAACAAGTACAGTTCACTACCCAAAGGTATATAAAACTTATTTCGAA	*360*

**PB2 245625 MiSeq**	*361*	AAAGTTGAAAGGTTGAAACACGGGACCTTTGGCCCTGTGCACTTCAGAAACCAAGTTAAG	*420*
**PB2 245626 Method A**	*361*	AAAGTTGAAAGGTTGAAACACGGGACCTTTGGCCCTGTGCACTTCAGAAACCAAGTTAAG	*420*
**PB2 245626 Method E**	*361*	AAAGTTGAAAGGTTGAAACACGGGACCTTTGGCCCTGTGCACTTCAGAAACCAAGTTAAG	*420*
**PB2 245626 Method K**	*361*	AAAGTTGAAAGGTTGAAACACGGGACCTTTGGCCCTGTGCACTTCAGAAACCAAGTTAAG	*420*
**PB2 245626 Method N**	*361*	AAAGTTGAAAGGTTGAAACACGGGACCTTTGGCCCTGTGCACTTCAGAAACCAAGTTAAG	*420*

**PB2 245625 MiSeq**	*421*	ATAAGACGGAGGGTCGATATAAACCCGGGCCATGCTGACCTCAGTGCCAGAGAGGCGCAG	*480*
**PB2 245626 Method A**	*421*	ATAAGACGGAGGGTCGATATAAACCCGGGCCATGCTGACCTCAGTGCCAGAGAGGCGCAG	*480*
**PB2 245626 Method E**	*421*	ATAAGACGGAGGGTCGATATAAACCCGGGCCATGCTGACCTCAGTGCCAGAGAGGCGCAG	*480*
**PB2 245626 Method K**	*421*	ATAAGACGGAGGGTCGATATAAACCCGGGCCATGCTGACCTCAGTGCCAGAGAGGCGCAG	*480*
**PB2 245626 Method N**	*421*	ATAAGACGGAGGGTCGATATAAACCCGGGCCATGCTGACCTCAGTGCCAGAGAGGCGCAG	*480*

**PB2 245625 MiSeq**	*481*	GATGTAATCATGGAAGTCGTCTTCCCAAATGAAGTGGGAGCAAGGATATTGACGTCGGAG	*540*
**PB2 245626 Method A**	*481*	GATGTAATCATGGAAGTCGTCTTCCCAAATGAAGTGGGAGCAAGGATATTGACGTCGGAG	*540*
**PB2 245626 Method E**	*481*	GATGTAATCATGGAAGTCGTCTTCCCAAATGAAGTGGGAGCAAGGATATTGACGTCGGAG	*540*
**PB2 245626 Method K**	*481*	GATGTAATCATGGAAGTCGTCTTCCCAAATGAAGTGGGAGCAAGGATATTGACGTCGGAG	*540*
**PB2 245626 Method N**	*481*	GATGTAATCATGGAAGTCGTCTTCCCAAATGAAGTGGGAGCAAGGATATTGACGTCGGAG	*540*

**PB2 245625 MiSeq**	*541*	TCACAACTGACGATAACAAAGGAGAAAAGGGAAGAACTCCAGGACTGCAAAATCGCTCCT	*600*
**PB2 245626 Method A**	*541*	TCACAACTGACGATAACAAAGGAGAAAAGGGAAGAACTCCAGGACTGCAAAATCGCTCCT	*600*
**PB2 245626 Method E**	*541*	TCACAACTGACGATAACAAAGGAGAAAAGGGAAGAACTCCAGGACTGCAAAATCGCTCCT	*600*
**PB2 245626 Method K**	*541*	TCACAACTGACGATAACAAAGGAGAAAAGGGAAGAACTCCAGGACTGCAAAATCGCTCCT	*600*
**PB2 245626 Method N**	*541*	TCACAACTGACGATAACAAAGGAGAAAAGGGAAGAACTCCAGGACTGCAAAATCGCTCCT	*600*

**PB2 245625 MiSeq**	*601*	CTGATGGTTGCATACATGCTAGAAAGAGAGTTGGTTCGCAAGACGAGGTTTCTCCCAGTT	*660*
**PB2 245626 Method A**	*601*	CTGATGGTTGCATACATGCTAGAAAGAGAGTTGGTTCGCAAGACGAGGTTTCTCCCAGTT	*660*
**PB2 245626 Method E**	*601*	CTGATGGTTGCATACATGCTAGAAAGAGAGTTGGTTCGCAAGACGAGGTTTCTCCCAGTT	*660*
**PB2 245626 Method K**	*601*	CTGATGGTTGCATACATGCTAGAAAGAGAGTTGGTTCGCAAGACGAGGTTTCTCCCAGTT	*660*
**PB2 245626 Method N**	*601*	CTGATGGTTGCATACATGCTAGAAAGAGAGTTGGTTCGCAAGACGAGGTTTCTCCCAGTT	*660*
**PB2 245625 MiSeq**	*661*	GCTGGTGGGACAAGCAGTGTCTACATTGAGGTGCTGCATTTGACTCAGGGGACATGCTGG	*720*
**PB2 245626 Method A**	*661*	GCTGGTGGGACAAGCAGTGTCTACATTGAGGTGCTGCATTTGACTCAGGGGACATGCTGG	*720*
**PB2 245626 Method E**	*661*	GCTGGTGGGACAAGCAGTGTCTACATTGAGGTGCTGCATTTGACTCAGGGGACATGCTGG	*720*
**PB2 245626 Method K**	*661*	GCTGGTGGGACAAGCAGTGTCTACATTGAGGTGCTGCATTTGACTCAGGGGACATGCTGG	*720*
**PB2 245626 Method N**	*661*	GCTGGTGGGACAAGCAGTGTCTACATTGAGGTGCTGCATTTGACTCAGGGGACATGCTGG	*720*
**PB2 245625 MiSeq**	*721*	GAGCAGATGTACACTCCGGGAGGAGAAGTGAGGAACGATGATGTAGACCAAAGCTTGATC	*780*
**PB2 245626 Method A**	*721*	GAGCAGATGTACACTCCGGGAGGAGAAGTGAGGAACGATGATGTAGACCAAAGCTTGATC	*780*
**PB2 245626 Method E**	*721*	GAGCAGATGTACACTCCGGGAGGAGAAGTGAGGAACGATGATGTAGACCAAAGCTTGATC	*780*
**PB2 245626 Method K**	*721*	GAGCAGATGTACACTCCGGGAGGAGAAGTGAGGAACGATGATGTAGACCAAAGCTTGATC	*780*
**PB2 245626 Method N**	*721*	GAGCAGATGTACACTCCGGGAGGAGAAGTGAGGAACGATGATGTAGACCAAAGCTTGATC	*780*
**PB2 245625 MiSeq**	*781*	ATTGCTGCCAGGAATATAGTAAGAAGAGCAACGGTATCAGCAGATCCATTAGCATCTCTA	*840*
**PB2 245626 Method A**	*781*	ATTGCTGCCAGGAATATAGTAAGAAGAGCAACGGTATCAGCAGATCCATTAGCATCTCTA	*840*
**PB2 245626 Method E**	*781*	ATTGCTGCCAGGAATATAGTAAGAAGAGCAACGGTATCAGCAGATCCATTAGCATCTCTA	*840*
**PB2 245626 Method K**	*781*	ATTGCTGCCAGGAATATAGTAAGAAGAGCAACGGTATCAGCAGATCCATTAGCATCTCTA	*840*
**PB2 245626 Method N**	*781*	ATTGCTGCCAGGAATATAGTAAGAAGAGCAACGGTATCAGCAGATCCATTAGCATCTCTA	*840*
**PB2 245625 MiSeq**	*841*	TTGGAGATGTGTCACAGCACACAAATTGGGGGAATAAGGATGGTAGACATCCTTCGGCAA	*900*
**PB2 245626 Method A**	*841*	TTGGAGATGTGTCACAGCACACAAATTGGGGGAATAAGGATGGTAGACATCCTTCGGCAA	*900*
**PB2 245626 Method E**	*841*	TTGGAGATGTGTCACAGCACACAAATTGGGGGAATAAGGATGGTAGACATCCTTCGGCAA	*900*
**PB2 245626 Method K**	*841*	TTGGAGATGTGTCACAGCACACAAATTGGGGGAATAAGGATGGTAGACATCCTTCGGCAA	*900*
**PB2 245626 Method N**	*841*	TTGGAGATGTGTCACAGCACACAAATTGGGGGAATAAGGATGGTAGACATCCTTCGGCAA	*900*

**PB2 245625 MiSeq**	*901*	AATCCAACAGAGGAACAAGCCGTGGATATATGCAAGGCAGCAATGGGCTTGAGGATTAGC	*960*
**PB2 245626 Method A**	*901*	AATCCAACAGAGGAACAAGCCGTGGATATATGCAAGGCAGCAATGGGCTTGAGGATTAGC	*960*
**PB2 245626 Method E**	*901*	AATCCAACAGAGGAACAAGCCGTGGATATATGCAAGGCAGCAATGGGCTTGAGGATTAGC	*960*
**PB2 245626 Method K**	*901*	AATCCAACAGAGGAACAAGCCGTGGATATATGCAAGGCAGCAATGGGCTTGAGGATTAGC	*960*
**PB2 245626 Method N**	*901*	AATCCAACAGAGGAACAAGCCGTGGATATATGCAAGGCAGCAATGGGCTTGAGGATTAGC	*960*

**PB2 245625 MiSeq**	*961*	TCATCTTTCAGCTTTGGTGGATTCACTTTTAAAAGAACAAGTGGATCGTCAGTCAAAAAA	*1020*
**PB2 245626 Method A**	*961*	TCATCTTTCAGCTTTGGTGGATTCACTTTTAAAAGAACAAGTGGATCGTCAGTCAAAAAA	*1020*
**PB2 245626 Method E**	*961*	TCATCTTTCAGCTTTGGTGGATTCACTTTTAAAAGAACAAGTGGATCGTCAGTCAAAAAA	*1020*
**PB2 245626 Method K**	*961*	TCATCTTTCAGCTTTGGTGGATTCACTTTTAAAAGAACAAGTGGATCGTCAGTCAAAAAA	*1020*
**PB2 245626 Method N**	*961*	TCATCTTTCAGCTTTGGTGGATTCACTTTTAAAAGAACAAGTGGATCGTCAGTCAAAAAA	*1020*

**PB2 245625 MiSeq**	*1021*	GAAGAAGAAGTGCTAACAGGCAACCTTCAAACATTGAAAATAAGAGTACATGAGGGGTAT	*1080*
**PB2 245626 Method A**	*1021*	GAAGAAGAAGTGCTAACAGGCAACCTTCAAACATTGAAAATAAGAGTACATGAGGGGTAT	*1080*
**PB2 245626 Method E**	*1021*	GAAGAAGAAGTGCTAACAGGCAACCTTCAAACATTGAAAATAAGAGTACATGAGGGGTAT	*1080*
**PB2 245626 Method K**	*1021*	GAAGAAGAAGTGCTAACAGGCAACCTTCAAACATTGAAAATAAGAGTACATGAGGGGTAT	*1080*
**PB2 245626 Method N**	*1021*	GAAGAAGAAGTGCTAACAGGCAACCTTCAAACATTGAAAATAAGAGTACATGAGGGGTAT	*1080*

**PB2 245625 MiSeq**	*1081*	GAAGAGTTCACAATGGTTGGGAGAAGAGCAACGGCCATTCTCAGGAAAGCAACCAGAAGG	*1140*
**PB2 245626 Method A**	*1081*	GAAGAGTTCACAATGGTTGGGAGAAGAGCAACGGCCATTCTCAGGAAAGCAACCAGAAGG	*1140*
**PB2 245626 Method E**	*1081*	GAAGAGTTCACAATGGTTGGGAGAAGAGCAACGGCCATTCTCAGGAAAGCAACCAGAAGG	*1140*
**PB2 245626 Method K**	*1081*	GAAGAGTTCACAATGGTTGGGAGAAGAGCAACGGCCATTCTCAGGAAAGCAACCAGAAGG	*1140*
**PB2 245626 Method N**	*1081*	GAAGAGTTCACAATGGTTGGGAGAAGAGCAACGGCCATTCTCAGGAAAGCAACCAGAAGG	*1140*

**PB2 245625 MiSeq**	*1141*	CTGATCCAGCTAATAGTAAGTGGGAGAGACGAGCAGTCAATTGCTGAAGCAATAATTGTG	*1200*
**PB2 245626 Method A**	*1141*	CTGATCCAGCTAATAGTAAGTGGGAGAGACGAGCAGTCAATTGCTGAAGCAATAATTGTG	*1200*
**PB2 245626 Method E**	*1141*	CTGATCCAGCTAATAGTAAGTGGGAGAGACGAGCAGTCAATTGCTGAAGCAATAATTGTG	*1200*
**PB2 245626 Method K**	*1141*	CTGATCCAGCTAATAGTAAGTGGGAGAGACGAGCAGTCAATTGCTGAAGCAATAATTGTG	*1200*
**PB2 245626 Method N**	*1141*	CTGATCCAGCTAATAGTAAGTGGGAGAGACGAGCAGTCAATTGCTGAAGCAATAATTGTG	*1200*

**PB2 245625 MiSeq**	*1201*	GCAATGGTATTCTCACAAGAGGACTGCATGATTAAAGCAGTTAGAGGTGACCTGAATTTT	*1260*
**PB2 245626 Method A**	*1201*	GCAATGGTATTCTCACAAGAGGACTGCATGATTAAAGCAGTTAGAGGTGACCTGAATTTT	*1260*
**PB2 245626 Method E**	*1201*	GCAATGGTATTCTCACAAGAGGACTGCATGATTAAAGCAGTTAGAGGTGACCTGAATTTT	*1260*
**PB2 245626 Method K**	*1201*	GCAATGGTATTCTCACAAGAGGACTGCATGATTAAAGCAGTTAGAGGTGACCTGAATTTT	*1260*
**PB2 245626 Method N**	*1201*	GCAATGGTATTCTCACAAGAGGACTGCATGATTAAAGCAGTTAGAGGTGACCTGAATTTT	*1260*

**PB2 245625 MiSeq**	*1261*	GTCAATAGGGCGAACCAGCGACTAAACCCAATGCATCAACTCTTGAGACATTTCCAAAAG	*1320*
**PB2 245626 Method A**	*1261*	GTCAATAGGGCGAACCAGCGACTAAACCCAATGCATCAACTCTTGAGACATTTCCAAAAG	*1320*
**PB2 245626 Method E**	*1261*	GTCAATAGGGCGAACCAGCGACTAAACCCAATGCATCAACTCTTGAGACATTTCCAAAAG	*1320*
**PB2 245626 Method K**	*1261*	GTCAATAGGGCGAACCAGCGACTAAACCCAATGCATCAACTCTTGAGACATTTCCAAAAG	*1320*
**PB2 245626 Method N**	*1261*	GTCAATAGGGCGAACCAGCGACTAAACCCAATGCATCAACTCTTGAGACATTTCCAAAAG	*1320*

**PB2 245625 MiSeq**	*1321*	GATGCAAAGGTGCTTTTCCAAAATTGGGGAATTGAACCCATTGACAATGTGATGGGAATG	*1380*
**PB2 245626 Method A**	*1321*	GATGCAAAGGTGCTTTTCCAAAATTGGGGAATTGAACCCATTGACAATGTGATGGGAATG	*1380*
**PB2 245626 Method E**	*1321*	GATGCAAAGGTGCTTTTCCAAAATTGGGGAATTGAACCCATTGACAATGTGATGGGAATG	*1380*
**PB2 245626 Method K**	*1321*	GATGCAAAGGTGCTTTTCCAAAATTGGGGAATTGAACCCATTGACAATGTGATGGGAATG	*1380*
**PB2 245626 Method N**	*1321*	GATGCAAAGGTGCTTTTCCAAAATTGGGGAATTGAACCCATTGACAATGTGATGGGAATG	*1380*

**PB2 245625 MiSeq**	*1381*	ATCGGGATAATGCCCGACATGACCCCAAGTACTGAGATGTCGCTGAGGGGAATAAGAGTC	*1440*
**PB2 245626 Method A**	*1381*	ATCGGGATAATGCCCGACATGACCCCAAGTACTGAGATGTCGCTGAGGGGAATAAGAGTC	*1440*
**PB2 245626 Method E**	*1381*	ATCGGGATAATGCCCGACATGACCCCAAGTACTGAGATGTCGCTGAGGGGAATAAGAGTC	*1440*
**PB2 245626 Method K**	*1381*	ATCGGGATAATGCCCGACATGACCCCAAGTACTGAGATGTCGCTGAGGGGAATAAGAGTC	*1440*
**PB2 245626 Method N**	*1381*	ATCGGGATAATGCCCGACATGACCCCAAGTACTGAGATGTCGCTGAGGGGAATAAGAGTC	*1440*

**PB2 245625 MiSeq**	*1441*	AGTAAGATGGGAGTAGATGAATACTCAAGTACAGAGCGAGTAGTAGTAAGCATCGACCGG	*1500*
**PB2 245626 Method A**	*1441*	AGTAAGATGGGAGTAGATGAATACTCAAGTACAGAGCGAGTAGTAGTAAGCATCGACCGG	*1500*
**PB2 245626 Method E**	*1441*	AGTAAGATGGGAGTAGATGAATACTCAAGTACAGAGCGAGTAGTAGTAAGCATCGACCGG	*1500*
**PB2 245626 Method K**	*1441*	AGTAAGATGGGAGTAGATGAATACTCAAGTACAGAGCGAGTAGTAGTAAGCATCGACCGG	*1500*
**PB2 245626 Method N**	*1441*	AGTAAGATGGGAGTAGATGAATACTCAAGTACAGAGCGAGTAGTAGTAAGCATCGACCGG	*1500*

Sequence	Start	Alignment	End
**PB2 245625 MiSeq**	*1501*	TTTTTAAGAGTTCGAGACCAACGGGGGAACGTACTATTATCACCCGAAGAGGTCAGTGAG	*1560*
**PB2 245626 Method A**	*1501*	TTTTTAAGAGTTCGAGACCAACGGGGGAACGTACTATTATCACCCGAAGAGGTCAGTGAG	*1560*
**PB2 245626 Method E**	*1501*	TTTTTAAGAGTTCGAGACCAACGGGGGAACGTACTATTATCACCCGAAGAGGTCAGTGAG	*1560*
**PB2 245626 Method K**	*1501*	TTTTTAAGAGTTCGAGACCAACGGGGGAACGTACTATTATCACCCGAAGAGGTCAGTGAG	*1560*
**PB2 245626 Method N**	*1501*	TTTTTAAGAGTTCGAGACCAACGGGGGAACGTACTATTATCACCCGAAGAGGTCAGTGAG	*1560*

Sequence	Start	Alignment	End
**PB2 245625 MiSeq**	*1561*	ACACAAGGAACGGAGAAACTGACAATCACTTATTCGTCATCAATGATGTGGGAGATAAAT	*1620*
**PB2 245626 Method A**	*1561*	AAACAAGGAACGGAGAAACTGACAATCACTTATTCGTCATCAATGATGTGGGAGATAAAT	*1620*
**PB2 245626 Method E**	*1561*	ACACAAGGAACGGAGAAACTGACAATCACTTATTCGTCATCAATGATGTGGGAGATAAAT	*1620*
**PB2 245626 Method K**	*1561*	ACACAAGGAACGGAGAAACTGACAATCACTTATTCGTCATCAATGATGTGGGAGATAAAT	*1620*
**PB2 245626 Method N**	*1561*	ACACAAGGAACGGAGAAACTGACAATCACTTATTCGTCATCAATGATGTGGGAGATAAAT	*1620*

Sequence	Start	Alignment	End
**PB2 245625 MiSeq**	*1621*	GGTCCTGAGTCTGTGTTGGTCAATACTTATCAGTGGATAATTAGAAACTGGGAAACTGTA	*1680*
**PB2 245626 Method A**	*1621*	GGTCCTGAGTCTGTGTTGGTCAATACTTATCAGTGGATAATTAGAAACTGGGAAACTGTA	*1680*
**PB2 245626 Method E**	*1621*	GGTCCTGAGTCTGTGTTGGTCAATACTTATCAGTGGATAATTAGAAACTGGGAAACTGTA	*1680*
**PB2 245626 Method K**	*1621*	GGTCCTGAGTCTGTGTTGGTCAATACTTATCAGTGGATAATTAGAAACTGGGAAACTGTA	*1680*
**PB2 245626 Method N**	*1621*	GGTCCTGAGTCTGTGTTGGTCAATACTTATCAGTGGATAATTAGAAACTGGGAAACTGTA	*1680*

Sequence	Start	Alignment	End
**PB2 245625 MiSeq**	*1681*	AAAATTCAATGGTCACAGGATCCCACAATGTTGTATAATAAGATGGAATTCGAGCCATTC	*1740*
**PB2 245626 Method A**	*1681*	AAAATTCAATGGTCACAGGATCCCACAATGTTGTATAATAAGATGGAATTCGAGCCATTC	*1740*
**PB2 245626 Method E**	*1681*	AAAATTCAATGGTCACAGGATCCCACAATGTTGTATAATAAGATGGAATTCGAGCCATTC	*1740*
**PB2 245626 Method K**	*1681*	AAAATTCAATGGTCACAGGATCCCACAATGTTGTATAATAAGATGGAATTCGAGCCATTC	*1740*
**PB2 245626 Method N**	*1681*	AAAATTCAATGGTCACAGGATCCCACAATGTTGTATAATAAGATGGAATTCGAGCCATTC	*1740*

Sequence	Start	Alignment	End
**PB2 245625 MiSeq**	*1741*	CAGTCTCTAGTCCCTAAGGCAGCCAGAGGTCAATACAGTGGGTTTGTGAGAACACTATTC	*1800*
**PB2 245626 Method A**	*1741*	CAGTCTCTAGTCCCTAAGGCAGCCAGAGGTCAATACAGTGGGTTTGTGAGAACACTATTC	*1800*
**PB2 245626 Method E**	*1741*	CAGTCTCTAGTCCCTAAGGCAGCCAGAGGTCAATACAGTGGGTTTGTGAGAACACTATTC	*1800*
**PB2 245626 Method K**	*1741*	CAGTCTCTAGTCCCTAAGGCAGCCAGAGGTCAATACAGTGGGTTTGTGAGAACACTATTC	*1800*
**PB2 245626 Method N**	*1741*	CAGTCTCTAGTCCCTAAGGCAGCCAGAGGTCAATACAGTGGGTTTGTGAGAACACTATTC	*1800*

**PB2 245625 MiSeq**	*1801*	CAGCAAATGCGAGATGTGCTTGGAACATTTGACACTGTTCAGATAATAAAACTCCTCCCC	*1860*
**PB2 245626 Method A**	*1801*	CAGCAAATGCGAGATGTGCTTGGAACATTTGACACTGTTCAGATAATAAAACTCCTCCCC	*1860*
**PB2 245626 Method E**	*1801*	CAGCAAATGCGAGATGTGCTTGGAACATTTGACACTGTTCAGATAATAAAACTCCTCCCC	*1860*
**PB2 245626 Method K**	*1801*	CAGCAAATGCGAGATGTGCTTGGAACATTTGACACTGTTCAGATAATAAAACTCCTCCCC	*1860*
**PB2 245626 Method N**	*1801*	CAGCAAATGCGAGATGTGCTTGGAACATTTGACACTGTTCAGATAATAAAACTCCTCCCC	*1860*
**PB2 245625 MiSeq**	*1861*	TTTGCTACCGCCCCACCGGAACAAAGTAGGATGCAGTTCTCCTCCCTGACTGTGAATGTA	*1920*
**PB2 245626 Method A**	*1861*	TTTGCTACCGCCCCACCGGAACAAAGTAGGATGCAGTTCTCCTCCCTGACTGTGAATGTA	*1920*
**PB2 245626 Method E**	*1861*	TTTGCTACCGCCCCACCGGAACAAAGTAGGATGCAGTTCTCCTCCCTGACTGTGAATGTA	*1920*
**PB2 245626 Method K**	*1861*	TTTGCTACCGCCCCACCGGAACAAAGTAGGATGCAGTTCTCCTCCCTGACTGTGAATGTA	*1920*
**PB2 245626 Method N**	*1861*	TTTGCTACCGCCCCACCGGAACAAAGTAGGATGCAGTTCTCCTCCCTGACTGTGAATGTA	*1920*
**PB2 245625 MiSeq**	*1921*	AGAGGATCAGGAATGAGGATACTGGTAAGAGGCAATTCACCAGTGTTCAATTACAACAAG	*1980*
**PB2 245626 Method A**	*1921*	AGAGGATCAGGAATGAGGATACTGGTAAGAGGCAATTCACCAGTGTTCAATTACAACAAG	*1980*
**PB2 245626 Method E**	*1921*	AGAGGATCAGGAATGAGGATACTGGTAAGAGGCAATTCACCAGTGTTCAATTACAACAAG	*1980*
**PB2 245626 Method K**	*1921*	AGAGGATCAGGAATGAGGATACTGGTAAGAGGCAATTCACCAGTGTTCAATTACAACAAG	*1980*
**PB2 245626 Method N**	*1921*	AGAGGATCAGGAATGAGGATACTGGTAAGAGGCAATTCACCAGTGTTCAATTACAACAAG	*1980*
**PB2 245625 MiSeq**	*1981*	GCCACCAAGAGGCTCACAGTTCTCGGGAAAGATGCAGGTGCATTGACCGAAGATCCAGAT	*2040*
**PB2 245626 Method A**	*1981*	GCCACCAAGAGGCTCACAGTTCTCGGGAAAGATGCAGGTGCATTGACCGAAGATCCAGAT	*2040*
**PB2 245626 Method E**	*1981*	GCCACCAAGAGGCTCACAGTTCTCGGGAAAGATGCAGGTGCATTGACCGAAGATCCAGAT	*2040*
**PB2 245626 Method K**	*1981*	GCCACCAAGAGGCTCACAGTTCTCGGGAAAGATGCAGGTGCATTGACCGAAGATCCAGAT	*2040*
**PB2 245626 Method N**	*1981*	GCCACCAAGAGGCTCACAGTTCTCGGGAAAGATGCAGGTGCATTGACCGAAGATCCAGAT	*2040*
**PB2 245625 MiSeq**	*2041*	GAAGGCACAGCTGGAGTGGAGTCTGCTGTTTTAAGAGGATTCCTCATTTTGGGCAAAGAA	*2100*
**PB2 245626 Method A**	*2041*	GAAGGCACAGCTGGAGTGGAGTCTGCTGTTTTAAGAGGATTCCTCATTTTGGGCAAAGAA	*2100*
**PB2 245626 Method E**	*2041*	GAAGGCACAGCTGGAGTGGAGTCTGCTGTTTTAAGAGGATTCCTCATTTTGGGCAAAGAA	*2100*
**PB2 245626 Method K**	*2041*	GAAGGCACAGCTGGAGTGGAGTCTGCTGTTTTAAGAGGATTCCTCATTTTGGGCAAAGAA	*2100*
**PB2 245626 Method N**	*2041*	GAAGGCACAGCTGGAGTGGAGTCTGCTGTTTTAAGAGGATTCCTCATTTTGGGCAAAGAA	*2100*

```
PB2 245625 MiSeq      2101  GACAAGAGATATGGCCCAGCATTGAGCATCAATGAGCTGAGCAATCTTGCAAAGGGAGAG  2160
PB2 245626 Method A   2101  GACAAGAGATATGGCCCAGCATTGAGCATCAATGAGCTGAGCAATCTTGCAAAGGGAGAG  2160
PB2 245626 Method E   2101  GACAAGAGATATGGCCCAGCATTGAGCATCAATGAGCTGAGCAATCTTGCAAAGGGAGAG  2160
PB2 245626 Method K   2101  GACAAGAGATATGGCCCAGCATTGAGCATCAATGAGCTGAGCAATCTTGCAAAGGGAGAG  2160
PB2 245626 Method N   2101  GACAAGAGATATGGCCCAGCATTGAGCATCAATGAGCTGAGCAATCTTGCAAAGGGAGAG  2160
```

```
PB2 245625 MiSeq      2161  AAGGCTAATGTGCTAATTGGGCAAGGAGACGTAGTGTTGGTGATGAAACGGAAACGGGAC  2220
PB2 245626 Method A   2161  AAGGCTAATGTGCTAATTGGGCAAGGAGACGTAGTGTTGGTGATGAAACGGAAACGGGAC  2220
PB2 245626 Method E   2161  AAGGCTAATGTGCTAATTGGGCAAGGAGACGTAGTGTTGGTGATGAAACGGAAACGGGAC  2220
PB2 245626 Method K   2161  AAGGCTAATGTGCTAATTGGGCAAGGAGACGTAGTGTTGGTGATGAAACGGAAACGGGAC  2220
PB2 245626 Method N   2161  AAGGCTAATGTGCTAATTGGGCAAGGAGACGTAGTGTTGGTGATGAAACGGAAACGGGAC  2220
```

```
PB2 245625 MiSeq      2221  TCTAGCATACTTACTGACAGCCAGACAGCGACCAAAAGAATTCGGATGGCCATCAATTAG  2280
PB2 245626 Method A   2221  TCTAGCATACTTACTGACAGCCAGACAGCGACCAAAAGAATTCGGATGGCCATCAATTAG  2280
PB2 245626 Method E   2221  TCTAGCATACTTACTGACAGCCAGACAGCGACCAAAAGAATTCGGATGGCCATCAATTAG  2280
PB2 245626 Method K   2221  TCTAGCATACTTACTGACAGCCAGACAGCGACCAAAAGAATTCGGATGGCCATCAATTAG  2280
PB2 245626 Method N   2221  TCTAGCATACTTACTGACAGCCAGACAGCGACCAAAAGAATTCGGATGGCCATCAATTAG  2280
```
